# Minor secoiridoid aglycones from the low-polarity part of the traditional Chinese herb: *Swertia mileensis*

**DOI:** 10.1007/s13659-013-0059-y

**Published:** 2013-10-16

**Authors:** Chang-An Geng, Xue-Mei Zhang, Yun-Bao Ma, Xiao-Yan Huang, Ji-Jun Chen

**Affiliations:** State Key Laboratory of Phytochemistry and Plant Resources in West China, Kunming Institute of Botany, Chinese Academy of Sciences, Kunming, 650201 China

**Keywords:** *Swertia mileensis*, swerimilegenins, secoiridoid aglycones, anti-HBV activity

## Abstract

Eleven new secoiridoid aglycones involving unusual C_9_-skeleton: swerimilegenins A-F (**1**–**6**); bis-C9-skeleton: swerimilegenin G (**7**); and C_10_-skeleton: swerimilegenins H-K (**8**–**11**), as well as six known ones, were isolated from the low-polarity part of the traditional Chinese herb medicine *Swertia mileensis*. Their structures were determined by extensive spectroscopic data and X-ray diffraction. Biogenetically, swerimilegenin A (**1**) belonged to 10-nor-secoiridoid, and swerimilegenins B-F (**2**–**6**) were 1-nor-secoiridoids. Erythrocentaurin (**12**) and gentiogenal (**15**) showed moderate anti-HBV activity on HepG 2.2.15 cell line *in vitro*.

**Electronic Supplementary Material:**

Supplementary material is available for this article at 10.1007/s13659-013-0059-y and is accessible for authorized users.

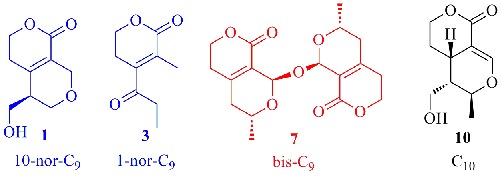

## Introduction

Secoiridoids, are biogenetically derived from the cleavage of the cylcopentane ring of iridiods (generally between C-7 and C-8), and represent a large and still expanding group of monoterpenoids. Secoiridoids are widely present in mainly in Gentianales, Dipsacales, Cornales and Oleaceae.[1,2] They have attracted significant interest from scientists for their diverse bioactivities, and conferred a diverse array of actions such as antiviral, cardiovascular, choleretic, hypoglycemic, anti-inflammatory and purgative activities, and chemotaxonomic significance.[3] Secoiridoids consistently have a hemiacetal hydroxy group at C-1 position, which is unstable and apt to be glycosylated, thus most of the secoiridoids from natural resources are secoiridoid glycosides.[4] According to the carbon numbers involved in the aglycone part, secoiridoids can be classified into three main types: I) the normal skeleton with ten carbons; II) skeletons with more than ten carbons in the aglycone part; and III) skeletons with less than ten carbons. Presently, quite a few cases of secoiridoids other than $C_{10}$ skeleton have been reported.[5–7] Subsequently, the discovery of more secoiridoid aglycones with dissimilar skeletons will help to formulate a greater understanding of these compounds.

Previous investigations of *Swertia mileensis* (‘Qing-Ye-Dan’ in Mandarin), which is a well-known traditional Chinese herb, endemic to the Yunnan province and documented in every edition of the Chinese Pharmacopoeia from 1977–2010, has shown that secoiridoid glycosides, xanthones and triterpenoids were their active constituents.[8–10] As an ongoing search for anti-hepatitis B virus (anti-HBV) active compounds from natural resources, our recent investigation on the active part of *S. mileensis* resulted in a series of novel lactones, swerilactones A–O and swerilactosides A–C.[11–17] From a biosynthetic perspective, swerilactones A and B ($C_{18}$ skeleton) were derived from two $C_9$ secoiridoids, and swerilactones H–K ($C_{29}$ skeleton) were condensed by two $C_{10}$ and one $C_9$ secoiridoids. In addition, swerilactone G and swerilactosides A and B also contained the characteristic $C_9$ secoiridoid moie-ties
in their structures. The above analysis suggests that *S. mileensis* may be rich in secoiridoid aglycones, especially for the unusual $C_9$ skeletons. Secoiridoids without glycosylation should be present in the low-polarity part of *S. mileensis*, however this part was ignored in the previous investigation, for its low-content and less-activity. Therefore, further investigation was focused on secoiridoid aglycones and was performed on the low-polarity part of *S. mileensis*, resulting in eleven new (**1**–**11**), as well as six pre-existing compounds (**12**–**17**). This paper will discuss their isolation, structural elucidation, and anti-HBV properties on HepG 2.2.15 cell line *in vitro*.

## Results and Discussion

Compound **1** had the molecular formula of $C_9H_{12}O_4$, which was determined by negative HRESIMS ($[M-H]^-$, detected at *m*/*z* 183.0686, calcd for 183.0663), indicating four degrees of unsaturation. In the $^{13}C$ NMR (DEPT) spectrum, three quaternary carbons, one methine and five methylenes, were observed. An *α*,*β*-unsaturated *δ*-lactone was proposed from the characteristic carbons at $\delta_C$ 163.0, 125.3, 149.5, 65.9 and 26.8, and HMBC correlations of H-7/C-5 and C-11, and H-6/C-4, which was in accordance with the UV (222 nm) and IR (1701

*To whom correspondence should be addressed. E-mail: chenjj@mail.kib.ac.cn and 1631 $cm^{-1}$) absorptions. Therefore, four residual carbons (three O-bearing methylenes and one methine) required an additional ring to fulfill the unsaturation. The connectivity of O-C(8)-C(9)-C(1)-O was constructed by the $^1$H-$^1$H COSY experiment (H-8/H-9/H-1), of which C-9 was proposed to be attached with C-5 by HMBC correlations from H-1 and H-8 to C-5 (Figure 1). In HMBC spectrum, correlations from $\delta_H$ 4.43 and 4.29 (H-3) to $\delta_C$ 149.5 (C-5), 163.0 (C-11) and 66.5 (C-1) suggested that C-3 was directly linked to C-4 and further cyclized with C-1 by an oxygen atom. The configuration at C-9 was determined as *R*-form based on its optical rotation ($[\alpha]_D^{18}$ − 12.4) and in comparison to the known compounds with the similar chiral center.[18,19] Thus, the structure of compound **1** was determined and named as swerimilegenin A.

Compound **2** was assigned the molecular formula of $C_9H_{14}O_3$ by HREIMS which showed $[M]^+$ at *m/z* 170.0944 (calcd. 170.0943), with three degrees of unsaturation. The existence of hydroxy and carbonyl groups was verified by IR absorptions at 3430 and 1711 $cm^{-1}$. Nine carbons involving three quaternary ones, five methylenes and one methyl were detected in the $^{13}$C NMR (DEPT) spectrum. The characteristic signals at $\delta_C$ 166.2 (s, C-11), 156.8 (s, C-5), 126.2 (s, C-4), 65.5 (t, C-7) and 28.6 (t, C-6) enabled the *α,β*-unsaturated *δ*-lactone moiety. A propyl group was proposed at C-5 based on $^1$H-$^1$H COSY (H-10/H-8/H-9) and HMBC experiments (H-9/C-4, C-6 and H-8/C-5). One remaining oxygenated methylene (C-3) was recognized with a hydroxmethyl group and assigned at C-4 according to the HMBC correlations of H-3/C-4 and C-5. Thus, the structure of compound **2** was constructed.

Compound **3** possessed the molecular formula of $C_9H_{12}O_3$ (four unsaturations) deduced from the pseudo-molecular ion peak of $[M + Na]^+$ (*m/z* 191) in ESIMS and $[M + H]^+$ (*m/z* 169.0866) in HRESIMS. The $^1$H and $^{13}$C NMR spectra of

**Figure 1.** Selected 2D NMR correlations for compounds **1–11**

**Table 1.** $^1$**H and** $^{13}$**C NMR data for compounds 1–5 (*δ* in ppm, *J* in Hz)**

| NO. | 1[a] $\delta_H$ | 1[a] $\delta_C$ | 2[a] $\delta_H$ | 2[a] $\delta_C$ | 3[a] $\delta_H$ | 3[a] $\delta_C$ | 4[b] $\delta_H$ | 4[b] $\delta_C$ | 5[a] $\delta_H$ | 5[a] $\delta_C$ |
|---|---|---|---|---|---|---|---|---|---|---|
| 1 | 4.03, dd, 11.4, 2,7<br>3.74, dd, 11.4, 3.5 | 66.5, t | – | – | – | – | – | – | – | – |
| 3 | 4.43, br. d, 16.2<br>4.29, br. d, 16.2 | 64.4, t | 4.35, s | 57.3, t | 2.00, s | 14.2, q | 5.57, d, 5.0 | 87.7, d | 5.25, s | 94.1, d |
| 4 | – | 125.3, s | – | 126.2, s | – | 127.1, s | – | 125.4, s | – | 123.6, s |
| 5 | – | 149.5, s | – | 156.8, s | – | 147.5, s | – | 153.7, s | – | 153.1, s |
| 6 | 2.60, m<br>2.43, m | 26.8, t | 2.45, t, 6.1 | 28.6, t | 2.61, m | 25.7, t | 2.57, ddd, 17.8, 8.0, 5.9<br>2.35, dt, 17.8, 4.5 | 29.0, t | 2.55, m<br>2.27, dt, 17.9, 4.6 | 28.4, t |
| 7 | 4.42, m | 65.9, t | 4.34, t, 6.1 | 65.5,t | 4.40, t, 6.1 | 65.6, t | 4.35, m | 65.9, t | 4.37, m | 65.1, t |
| 8 | 3.88, m | 62.9, t | 1.53, m | 21.0, t | 2.64, q, 7.2 | 35.1, t | 4.28, m | 61.6, d | 4.20, m | 61.3, d |
| 9 | 2.34, br. s | 40.3, d | 2.34, t, 7.4 | 35.1, t | – | 204.6, s | 2.24, dd, 18.8, 3.6<br>2.12, dd, 18.8, 10.8 | 37.2, t | 2.17, dd, 19.2, 8.7<br>2.14, dd, 19.2, 4.3 | 36.8, t |
| 10 | – | – | 0.94, t, 7.4 | 13.9, q | 1.15, t, 7.2 | 7.4, q | 1.17, d, 6.3 | 21.2, q | 1.29, d, 6.3 | 20.7, q |
| 11 | – | 163.0, s | – | 166.2, s | – | 165.4, s | – | 163.1, s | – | 162.4, s |
| OMe/OH | – | – | – | – | – | – | 5.38, d, 5.0 | – | 3.48, s | 55.7, q |

[a]Measured in $CDCl_3$; [b]Measured in acetone-$d_6$

compound **3** were similar to those of **2**, except that the hydroxymethyl (C-3) and methylene (C-9) in **2** were changed to be methyl and carbonyl in **3**. Two groups of adjacent protons at $\delta_H$ 1.15 (t, $J$ = 7.2 Hz, H-10) and 2.64 (q, $J$ = 7.2 Hz, H-8) in $^1$H NMR spectrum, together with the carbons at $\delta_C$ 204.6 (s), 35.1 (t) and 7.4 (q) in $^{13}$C NMR (DEPT) spectrum suggested a propionyl group which was proposed at C-5 based on the HMBC correlations of H-8/C-5 and H-6/C-9. The remaining singlet methyl was assigned at C-4 by the long range correlations of H-3 with C-4, C-5 and C-11 in HMBC experiment.

Compound **4** was obtained as a hemiacetal analogue with the molecular formula of $C_9H_{12}O_4$ by a positive HRESIMS experiment ($[M + Na]^+$ at *m/z* 207.0624 and $[2M + Na]^+$ at *m/z* 391.1245). The existence of hydroxy (3385 $cm^{-1}$) and carbonyl (1686 $cm^{-1}$) groups was specified in IR spectrum. A characteristic *δ*-lactone fragment was recognized from its $^1$H and $^{13}$C NMR data (Table 1). Besides the *δ*-lactone part, four residual carbons involving one di-oxygenated methine (C-3), one mono-oxygenated methine (C-8), one methylene (C-9), and one methyl (C-10), were proposed to occupy a cyclic pattern in agreement with the unsaturation degree. The proton signals at $\delta_H$ 1.17 (3H, d, $J$ = 6.3 Hz, H-10), 4.28 (1H, m, H-8), 2.24 (1H, dd, $J$ = 18.8, 3.6 Hz, H-9a) and 2.12 (1H, dd, $J$ = 18.8, 10.8 Hz, H-9b) in $^1$H NMR spectrum, as well as the $^1$H-$^1$H COSY correlations of H-10/H-8/H-9 manifested the connectivity of $CH_3$-CH(O-)-$CH_2$-, which was allocated at C-5 by HMBC cross-peaks from H-8 to C-5, and from H-9 to C-4, C-5 and C-6. The hemiacetal methine (C-3) was determined to be directly linked with C-4 and further cyclized with C-8 by an O-bridge based on the HMBC experiment (H-3/C-5, C-8 and C-11; HO-3/C-4; H-8/C-3).

In order to determine the stereochemistry of C-3 and C-8, a ROESY experiment was performed, however the key correlation of H-8 or H-10 with H-3 was not detected. This phenomenon is frequently encountered in secoiridoids whose structure contains a six-member ring fused with a *δ*-lactone fragment. The connection of C(10)-C(8)-O-C(3)-H bears a stable *W* configuration which makes a homolateral H-3 and H-10 departure from each other. Meanwhile, the hydroxyl group at C-3 was apt to form intramolecule hydrogen bond with O=C(11), resulted in OH-3 apart from H-8. Therefore, no correlation of H-3/H-10 or OH-3/H-8 was observed in the ROESY experiment, despite having been located at the same orientation. In this case, the dihedral angles of H-9a/H-8 and H-9b/H-8 are about 56.8° and 176.4° according to the Chem 3D model calculation (Figure 2), which are consistent with the

**Figure 2.** The *W* configuration in the Chem 3D model of compound **4**

coupling constants of 3.6 Hz ($J_{H-9a/H-8}$) and 10.8 Hz ($J_{H-9b/H-8}$). In addition, compound **7**, the dehydrated dimer of **4**, was also obtained and unambiguously determined by X-ray single crystal diffraction. Compounds **4** and **7** had the almost identical NMR spectral data and similar specific rotation value (–2.06° for **4**, about half of –6.65° for **7**) indicating the same stereochemistry.

Compound **5** was proposed the molecular formula of $C_{10}H_{14}O_4$ based on positive HRESIMS, 14 mass units ($CH_2$) higher than swerimilegenin D (**4**). Its $^1$H and $^{13}$C NMR spectroscopic data were similar to those of **4** except for an additional methoxy group ($\delta_H$ 3.48, $\delta_C$ 55.7). With the aid of HMBC analysis, the methoxy group was deduced at C-3 by the correlation of OMe with C-3. Similar to swerimilegenin D, no correlation between H-3 (or OMe) and H-8 (or H-10) was detected in the ROESY experiment due to the *W* configuration. The stereocenters of C-3 and C-8 in compound **5** were deduced to be the same as those of **4** by comparing their coupling constant in $^1$H NMR spectrum and $[\alpha]_D$ values.

Compound **6** was designated the molecular formula of $C_{10}H_{12}O_5$ by positive HRESIMS ($[M + Na]^+$, as detected at *m/z* 235.0586, calcd. for 235.0582). A methoxy group was speculated by the ion peak at *m/z* 181 ($M^+$ – OMe) in the EIMS spectrum. Its $^1$H and $^{13}$C NMR data were similar to those of **5**, except for the additional carbonyl group (196.6, s) instead of methylene [$\delta_C$ 36.8 (t, C-9)] in **5**. The C(9)=O was evidenced based on the HMBC correlations of H-10/C-9, H-8/C-9 and C-5. Similar to compounds **4** and **5**, neither H-8/H-3 nor Me-10/H-3 was detected in its ROESY spectrum due to the stable *W* configuration. The stereochemistry of compound **6** was proposed to be the same as compounds **4** and **5** by the coupling constant ($J_{H-10/H-8}$ = 6.5 Hz) and optical

**Table 2. $^{1}$H and $^{13}$C NMR data for compounds 6–9 ($\delta$ in ppm, $J$ in Hz)**

| NO. | 6[a] | | 7[b] | | 8[c] | | 9[a] | |
|---|---|---|---|---|---|---|---|---|
| | $\delta_H$ | $\delta_C$ | $\delta_H$ | $\delta_C$ | $\delta_H$ | $\delta_C$ | $\delta_H$ | $\delta_C$ |
| 1 | – | – | – | – | 5.90, d, 5.0 | 88.0, d | 5.42, s | 94.7, d |
| 3 | 5.46, d, 1.2 | 93.0, d | 6.27, s | 88.2, d | 4.50, d, 16.0<br>4.23, d, 16.4 | 56.5, t | 4.41, m | 56.7, t |
| 4 | – | 135.8, s | – | 123.3, s | – | 131.3, s | – | 130.8, s |
| 5 | – | 139.9, s | – | 154.8, s | – | 142.8, s | – | 142.4, s |
| 6 | 2.66, m | 19.6, t | 2.30, ddd, 17.5, 9.6, 6.5<br>2.06, dt, 17.5, 4.9 | 28.6, t | 2.63, m<br>2.55, m | 22.4, t | 2.56, m | 22.4, t |
| 7 | 4.48, m | 66.4, t | 4.23, m | 65.7, t | 4.39, m | 65.7, t | 4.41, m | 65.6, t |
| 8 | 4.64, q, 6.5 | 69.6, d | 4.71, m | 62.5, d | 6.12, q, 7.3 | 130.7, d | 6.09, q, 7.1 | 130.7, d |
| 9 | – | 196.6, s | 2.00, dd, 18.8, 10.8<br>1.88, dd, 18.8, 3.8 | 36.6, t | – | 120.4, s | – | 120.7, s |
| 10 | 1.45, d, 6.5 | 15.6, q | 1.36, d, 6.3 | 20.8, q | 1.89, d, 7.3 | 13.8, q | 1.89, d, 7.1 | 13.8, q |
| 11 | – | 162.4, s | – | 162.8, s | – | 164.2, s | – | 163.8, s |
| OMe/OH | 3.59, s | 57.0, q | – | – | 5.49, d, 5.0 | – | 3.51, s | 55.1, q |

[a]Measured in $CDCl_3$; [b]Measured in $C_5D_5N$; [c]Measured in acetone-$d_6$

rotation value ($[\alpha]_D^{24}$ = – 4.0).

Compound **7** was determined with the molecular formula of $C_{18}H_{22}O_7$ from positive HRESIMS (detected at *m/z* 373.1258). Only nine carbons and 11 protons were displayed in the $^{13}$C and $^{1}$H NMR spectra, indicating a symmetrical skeleton. Its $^{1}$H and $^{13}$C NMR spectral data were almost identical with those of **4**, indicating the similar structures. The above analysis together with its molecular formula of $C_{18}H_{22}O_7$ (18 mass units less than two molecules of **4**) suggested that compound **7** should be the dehydrated dimer of **4**, which was finally confirmed by extensive 2D NMR experiment and X-ray single crystal diffraction (Figure 3).

Compound **8** possessed the molecular formula of $C_{10}H_{12}O_4$ in accordance with the ion peaks of $M^+$ at *m/z* 196 in EIMS and $[M + Na]^+$ at *m/z* 219.0641 in HRESIMS. The presence of OH and C=O groups was evidenced by the absorption bands at 3362 and 1699 $cm^{-1}$ in IR spectrum. An obvious *α,β*-unsaturated *δ*-lactone fragment was revealed from its $^{1}$H and $^{13}$C NMR spectroscopic data (Table 2). In addition, one $CH_3CH$=C moiety was proposed to be linked with C-5 and C-1 from the HMBC correlations of H-8 with C-5, H-6 with C-9 and H-1 with C-5 and C-8. The HMBC correlations of H-3 with C-4, C-5 and C-1 revealed the linkage of C(4)-C(3)-O-C(1). The *Z*-form of the double bond between C-8 and C-9 was determined by the correlation of H-8 with H-6 in the ROESY spectrum.

Compound **9** had the molecular formula of $C_{11}H_{14}O_4$ as a result of a positive HRESIMS experiment, with an additional $CH_2$ moiety compared to compound **8**. Its IR, UV and NMR spectroscopic data were similar to **8**, except for an additional methoxy group. In HMBC spectrum, the correlations of OC<u>H</u>$_3$/C-1 and H-1/O<u>C</u>H$_3$ indicated that the methoxy group was located at C-1 position. Finally, its structure was determined by an X-ray single crystal diffraction experiment.

Compound **10** was proposed with a molecular formula of $C_{10}H_{14}O_4$ by HREIMS at *m/z* 198.0897. The IR spectrum displayed the absorption bands at 3472, 1679, 1595 and 1404 $cm^{-1}$ denoting the presence of OH, C=O and C=C groups. Its NMR spectroscopic data was close to that of dihydroepinaucledal (**14**), indicating a similar structure. From its $^{1}$H-$^{1}$H COSY and HMBC experiments, compound **10** was established the same planar structure as **14**. Therefore, their $^{1}$H and $^{13}$C NMR spectral differences (Table 3) might be ascribed to the stereochemical variation. In the ROESY spectrum, the correlations of H-5 with H-9 and H-10, and H-1 with H-8 suggested the same orientation of H-5 with H-9 and H-10, which display an obvious difference from the ROESY experiment of **14**, in which the correlations of H-5/H-1 and H-10, and H-9/H-8 were observed (see Electronic Supplementary Material).

Compound **11** possessed the molecular formula of $C_{10}H_{14}O_4$ identical with swerimilegenin J from HRESIMS. The same planar structures of **11** with **10** and **14** were constructed based on extensive $^{1}$H-$^{1}$H COSY and HMBC analysis. In the ROESY spectrum, the correlations of H-5 with H-1 and H-8, and H-9

**Figure 3.** X-ray crystal structures of compounds **7** and **9**

**Table 3.** $^1H$ **and** $^{13}C$ **NMR data for compounds 10, 11 and dihydroepinaucledal (14) (δ in ppm, J in Hz)**

| NO. | 10[a] $\delta_H$ | 10[a] $\delta_C$ | 11[a] $\delta_H$ | 11[a] $\delta_C$ | 14[a] $\delta_H$ | 14[a] $\delta_C$ |
|---|---|---|---|---|---|---|
| 1 | 3.74, dd, 10.4, 5.8<br>3.48, dd, 10.4, 8.6 | 60.1, t | 3.90, d, 2.7 | 59.2, t | 3.76, dd, 10.8, 5.1<br>3.45, dd, 10.8, 8.7 | 60.9, t |
| 3 | 7.69, d, 2.2 | 155.3, d | 7.71, s | 156.5, d | 7.61, d, 1.8 | 155.6, d |
| 4 | – | 101.6, s | – | 104.6, s | – | 102.4, s |
| 5 | 2.88, dddd, 11.5, 5.8, 5.8, 2.2 | 28.2, d | 2.74, ddd, 12.4, 12.4, 1.9 | 31.3, d | 2.31, dddd, 11.6, 11.6, 4.0, 1.8 | 28.4, d |
| 6 | 1.86, m | 25.4, t | 2.15, m<br>1.52, m | 27.6, t | 2.02, m<br>1.55, m | 26.9, t |
| 7 | 4.49, ddd, 11.3, 4.1, 2.4<br>4.30, ddd, 11.3, 11.3, 3.9 | 68.5, t | 4.47, ddd, 11.3, 4.3, 1.9<br>4.30, ddd, 11.3, 11.3, 2.4 | 68.3, t | 4.40, ddd, 11.5, 4.4, 1.8<br>4.23, ddd, 11.5, 11.5, 2.5 | 68.0, t |
| 8 | 4.76, qd, 6.5, 1.2 | 73.0, d | 4.18, dq, 10.4, 6.3 | 75.2, d | 4.61, qd, 6.8, 4.6 | 73.5, d |
| 9 | 1.95, dddd, 8.6, 5.8, 5.8, 1.2 | 39.8, d | 1.23, dddd, 12.4, 10.4, 2.7, 2.7 | 44.2, d | 1.77, dddd, 11.6, 8.7, 5.1, 4.6 | 41.7, d |
| 10 | 1.35, d, 6.5 | 19.3, q | 1.44, d, 6.3 | 18.8, q | 1.16, d, 6.8 | 14.1, q |
| 11 | – | 166.2, s | – | 166.5, s | – | 166.4, s |

[a]Measured in $CDCl_3$

with H-10 manifested the α-orientation of H-5, H-8 and hydroxymethyl (C-1), and the β-orientation of H-9 and Me-10.

The known dihydroepinaucledal (**14**) was determined by comparing its NMR data with that of a previous report[20] and further confirmed by a 2D NMR experiment (see Electronic Supplementary Material). The other known compounds were determined as erythrocentaurin (**12**),[21] gentiolactone (**13**),[22] gentiogenal (**15**),[23] secostrychnosin (**16**)[24] and angelone (**17**)[5] by comparing their spectroscopic data to existing report data.

In order to evaluate their anti-HBV properties, namely inhibiting the secretions of hepatitis B surface antigen (HBsAg) and hepatitis B e antigen (HBeAg), as well as HBV DNA replication, the isolates were assayed on HepG 2.2.15 cell line *in vitro* (Table 4). Most of the tested compounds exhibited low activity and cytotoxicity at the highest tested concentration, except for erythrocentaurin and gentiogenal. Erythrocentaurin could inhibit the HBsAg secretion and HBV DNA replication with the $IC_{50}$ values of 1.39 ± 0.54 and 0.96 ± 0.45 mM, respectively. Gentiogenal showed activity inhibiting the secretion of HBsAg and HBeAg with the $IC_{50}$ values of 3.92 ± 0.82 and 2.99 ± 0.72 mM.

Previously, most of the secoiridoids isolated from *Swertia* plants were secoiridoid glycosides and always contained a $C_{10}$ aglycone part. Interestingly, this investigation resulted in eleven new secoiridoid aglycones including unusual $C_9$-skeleton (**1**–**6**), bis-$C_9$-skeleton (**7**) and $C_{10}$-skeleton (**8**–**11**) from the low-polarity part of *S. mileensis*. Structurally, compound **1** belonged to 10-nor-secoiridoid, and compounds **2**–**6** were attributed to 1-nor-secoiridoids, which further enriched the skeleton types of secoiridoids. It is concluded that diverse secoiridoid aglycones are contained in *S. mileensis*, although these compounds may be not corresponding to the anti-HBV activity *in vitro*.

## Experimental Section

**General Experimental Procedures.** Melting points were measured on a SGWX-4B instrument (Shanghai Jingke, Shanghai, China). 1D and 2D NMR spectra were recorded on Bruker AV-400 NMR, DRX-500 or AVANCE III-600 spectrometers (Bruker, Bremerhaven, Germany) corrected by deuterated solvent signals. MS data was collected on LCMS-IT-TOF (Shimadzu, Kyoto, Japan) or VG Auto Spec-3000 (VG, Manchester, UK) spectrometers. IR (KBr) spectra was recorded on a Bruker Tensor 27 FT-IR (Bruker Optics GmbH, Ettlingen, Germany). UV data was collected on a Shimadzu UV-2401A spectrophotometer (Shimadzu, Kyoto, Japan). Optical rotations were collected on a Jasco model 1020 polarimeter (Horiba, Tokyo, Japan). Sephadex LH-20 (20–150 μm) was purchased from Pharmacia Fine Chemicals Co. Ltd. (Pharmacia, Uppsala, Sweden). Silica gel (200–300 mesh) for column chromatography was obtained from Qingdao Makall Chemical Company (Makall, Qingdao, China). CHP20P MCI gel (Mitsubishi Chemical Corporation, Tokyo, Japan) and Merck Lichrosorb RP-18 (Merck KGaA, Darmstadt, Germany) were applied for medium pressure liquid chromatographic (MPLC) preparation, which was performed on a Dr-Flash-S MPLC system (Lisui, Suzhou, China). Agilent Eclipse XDB-C18 column (Agilent Technologies, Santa Clara, USA) was used for semi-preparation, which was conducted on a Waters 600-2695 HPLC instrument (Waters, Milford, MA, USA), equipped with a photodiode array detector (Waters 2996).

**Plant Material.** The whole plants of *Swertia mileensis* T. N. Ho et W. L. Shi were collected in Mile County, Yunnan Province, China, in November, 2008, and were identified by Prof. Li-Gong Lei. A voucher specimen (No. 2008-11-01) was deposited in the Laboratory of Antivirus and Natural Medicine Chemistry, Kunming Institute of Botany, CAS.

**Extraction and Isolation.** The air-dried and powered whole plant (5.0 kg) of *S. mileensis* was extracted with ethanol and condensed to provide a residue (1.3 kg). The residue was partitioned between water and petroleum ether (PE, 1 L × 2), ethyl acetate (EtOAc, 1 L × 3) and *n*-butanol (1 L × 3) successively. The EtOAc part (170 g) was chromatographed on silica gel column (2 kg, 110 mm id × 500 mm) eluted with $CHCl_3$-MeOH to provide fractions A−J. Fraction A (29 g) was separated by silica gel column chromatography (CC, 300 g, 50 mm id × 240 mm) with an eluent of $CHCl_3$-$Me_2CO$ system (0:100 → 50:50, *v/v*) to yield Frs. $A_1$–$A_5$.[11] Frs. $A_1$ and $A_2$ were combined based on TLC detection which displayed many identical spots both under UV detector and treated with $H_2SO_4$ regent, and subjected to silica gel CC (200 g, 25 mm id × 600 mm) eluted with $CHCl_3$/MeOH gradient (100:0, 95:5, 90:10 and 80:20, with 2 L, respectively), to obtain three fractions $A_{1A}$–$A_{1C}$. Fraction $A_{1B}$ (5 g) was separated by CHP20P MCI gel column chromatography (100.0 g, 25 mm id × 300 mm) on a MPLC system eluted with MeOH/$H_2O$ (from 20:80 to 80:20, 10 L) to yield seven sub-fractions (Frs. $A_{1B-1}$ to $A_{1B-7}$). Fr. $A_{1B-1}$ (300 mg) was purified with a silica gel column (50 g, 25 mm id × 150 mm) eluting with PE/acetone (from 80:20 to

**Table 4. Anti-HBV activities of the isolates from *S. mileensis* (mM)**

| Compounds | MTT | HBsAg | | HBeAg | | HBV DNA | |
|---|---|---|---|---|---|---|---|
| | $CC_{50}$ | $IC_{50}$ | SI[a] | $IC_{50}$ | SI | $IC_{50}$ | SI |
| **1** | > 1.55 | > 1.55 | –[b] | > 1.55 | – | > 0.39 | – |
| **2** | > 1.62 | > 1.62 | – | > 1.62 | – | > 0.40 | – |
| **3** | > 3.86 | > 3.86 | – | > 3.86 | – | > 0.98 | – |
| **4** | > 2.67 | > 2.67 | – | > 2.67 | – | > 0.67 | – |
| **5** | > 2.83 | > 2.83 | – | > 2.83 | – | > 0.71 | – |
| **6** | > 6.52 | > 6.52 | – | > 6.52 | – | > 1.63 | – |
| **7** | > 1.40 | > 1.40 | – | > 1.40 | – | > 0.35 | – |
| **8** | > 2.70 | > 2.70 | – | > 2.70 | – | > 0.68 | – |
| **9** | > 0.64 | > 0.64 | – | > 0.64 | – | > 0.16 | – |
| **10** | > 2.47 | > 2.47 | – | > 2.47 | – | > 0.62 | – |
| **11** | > 2.07 | > 2.07 | – | > 2.07 | – | > 0.52 | – |
| **12** | 1.97 ± 0.38[d] | 1.39 ± 0.54 | 1.4 | 2.84 ± 0.79 | <1 | 0.96 ± 0.45 | 2.0 |
| **13** | > 0.69 | > 0.69 | – | > 0.69 | – | > 0.17 | – |
| **14** | > 0.55 | > 0.55 | – | > 0.55 | – | > 0.14 | – |
| **15** | 3.97 ± 0.78 | 3.92 ± 0.82 | 1.0 | 2.99 ± 0.72 | 1.3 | > 1.24 | – |
| **16** | > 1.13 | > 1.13 | – | > 1.13 | – | > 0.28 | – |
| **17** | > 0.44 | > 0.44 | – | > 0.44 | – | > 0.11 | – |
| **tenofovir**[c] | > 1865.36 | > 1865.36 | – | > 1865.36 | | 3.12 ± 0.78 | > 597.9 |

[a]SI (selective index: $CC_{50}/IC_{50}$); [b]$IC_{50}$ and $CC_{50}$ values were not reached at the (highest) tested concentrations.
[c]Tenofovir was used as the positive control; [d]Results were expressed as the mean (mM) ± standard deviations (SD) from three independent experiments.

50:50, 800 mL) to afford compounds **3** (6 mg), **6** (8 mg), **12** (150 mg) and **15** (9 mg). Compounds **4** (5.0 mg), **5** (5.0 mg), **7** (6.0 mg), **8** (9.0 mg) and **9** (6.0 mg) were isolated from Fr. $A_{1B\text{-}2}$ (100 mg) by Sephadex LH-20 (50.0 g, 14 mm id × 1450 mm, MeOH, 300 mL) and repeated silica gel CC (20 g, 14 mm id × 250 mm, PE/acetone = 80:20, 500 mL). Fr. $A_{1B\text{-}3}$ (400 mg) was separated by RP-18 column (50 g, 25 mm id × 130 mm) eluted with MeOH/$H_2O$ (40:60, 1000 mL) on MPLC system and further purified by repeated silica gel CC to give compounds **1** (3 mg), **2** (3 mg) and **13** (6 mg). Compounds **17** (7 mg) and **16** (3 mg) were obtained from Fr. $A_{1B\text{-}4}$ (60 mg) by repeated silica gel CC (20 g, 14 mm id × 250 mm) eluted with PE/acetone (70:30, 300 mL) and $CHCl_3$/acetone (85:15, 300 mL) systems. Fr. $A_{1B\text{-}5}$ (50 mg) was loaded on a HPLC apparatus using an Eclipse XDB-C18 column (94 mm id × 250 mm, 5 µm) and eluted with MeOH-$H_2O$ (25:75, flow rate = 3 mL/min) providing compounds **10** (4 mg), **11** (6 mg) and **14** (8 mg). The purity of all the isolates was proposed to be higher than 95% based on TLC (only one spot under UV radiation and iodine atmosphere) and NMR (smooth baseline without impurity peaks) methods.

**Swerimilegenin A (1):** white amorphous powder; $[\alpha]_D^{18}$ – 12.4 (*c* 0.06, MeOH); UV (MeOH) $\lambda_{max}$ (log *ε*) 222 (3.54) nm; IR (KBr) $\nu_{max}$ 3418, 2926, 1701, 1631, 1413, 1385, 1048 $cm^{-1}$; for $^1$H and $^{13}$C NMR data, see Table 1; HRESIMS *m/z* 183.0686 $[M – H]^-$ (calcd. for $C_9H_{11}O_4$, 183.0663).

**Swerimilegenin B (2):** white amorphous powder; UV (MeOH) $\lambda_{max}$ (log *ε*) 223 (3.82) nm; IR (KBr) $\nu_{max}$ 3430, 2963, 1711, 1469, 1404, 1314, 1170, 1142, 1074, 1008 $cm^{-1}$; $^1$H and $^{13}$C NMR data, see Table 1; HREIMS *m/z* 170.0944 $M^+$ (calcd. for $C_9H_{14}O_3$, 170.0943).

**Swerimilegenin C (3):** white amorphous powder; UV (MeOH) $\lambda_{max}$ (log *ε*) 229 (3.83) nm; IR (KBr) $\nu_{max}$ 2939, 1720, 1403, 1309, 1173, 1130, 1072, 1130, 795 $cm^{-1}$; $^1$H and $^{13}$C NMR data, see Table 1; ESIMS *m/z* 191 $[M + Na]^+$; HRESIMS *m/z* 169.0866 $[M + H]^+$ (calcd. for $C_9H_{13}O_3$, 169.0864).

**Swerimilegenin D (4):** white amorphous powder; $[\alpha]_D^{25}$ – 2.0 (*c* 0.2, MeOH); UV (MeOH) $\lambda_{max}$ (log *ε*) 209 (3.89) nm; IR (KBr) $\nu_{max}$ 3385, 2976, 1686, 1405, 1279, 1169, 1086, 1056, 1023, 986 $cm^{-1}$; $^1$H and $^{13}$C NMR data, see Table 1; EIMS *m/z* 184 $M^+$ (17), 183 (100), 167 (53), 140 (76), 125 (55), 94 (50); HRESIMS *m/z* 207.0624 $[M + Na]^+$ (calcd. for $C_9H_{12}O_4Na$, 207.0633).

**Swerimilegenin E (5):** white amorphous powder; $[\alpha]_D^{22}$ – 6.1 (*c* 0.2, MeOH); UV (MeOH) $\lambda_{max}$ (log *ε*) 207 (3.87) nm; IR (KBr) $\nu_{max}$ 2908, 1712, 1410, 1279, 1150, 983, 966 $cm^{-1}$; $^1$H and $^{13}$C NMR data, see Table 1; HRESIMS *m/z* 221.0763 $[M + Na]^+$ (calcd. for $C_{10}H_{14}O_4Na$, 221.0784).

**Swerimilegenin F (6):** colorless gum; $[\alpha]_D^{24}$ – 4.0 (*c* 0.2, MeOH); UV (MeOH) $\lambda_{max}$ (log *ε*) 227 (3.87) nm; IR (KBr) $\nu_{max}$ 2937, 1727, 1700, 1415, 1214, 1083, 1060, 975, 851 $cm^{-1}$; $^1$H and $^{13}$C NMR data, see Table 2; EIMS *m/z* 181 (35), 168 (100), 151 (25), 140 (20), 110 (17), 82 (55); ESIMS *m/z* 235 $[M + Na]^+$; HRESIMS *m/z* 235.0586 $[M + Na]^+$ (calcd. for $C_{10}H_{12}O_5Na$, 235.0582).

**Swerimilegenin G (7):** colorless cubic crystals; mp 228–230 °C; $[\alpha]_D^{26}$ – 6.6 (*c* 0.3, MeOH/$CHCl_3$); UV (MeOH) $\lambda_{max}$ (log *ε*) 210 (4.22) nm; IR (KBr) $\nu_{max}$ 2976, 2936, 2908, 1721, 1707, 1473, 1414, 1323, 1281, 1156, 1060, 962, 809, 661, 561 $cm^{-1}$; $^1$H and $^{13}$C NMR data, see Table 2; ESIMS *m/z* 373 $[M + Na]^+$; HRESIMS *m/z* 373.1258 $[M + Na]^+$ (calcd. for $C_{18}H_{22}O_7Na$, 373.1263).

**Swerimilegenin H (8):** colorless gum; $[\alpha]_D^{25}$ – 3.9 (*c* 0.4, MeOH); UV (MeOH) $\lambda_{max}$ (log *ε*) 273 (3.78), 237 (3.82) nm; IR (KBr) $\nu_{max}$ 3362, 2954, 1699, 1637, 1419, 1286, 1064, 1030, 912, 773, 747 $cm^{-1}$; $^1$H and $^{13}$C NMR data, see Table 2; EIMS *m/z* 196 $M^+$ (10), 178 (90), 167 (20), 149 (24), 121 (30), 105

(26), 91 (100), 79 (25), 77 (45); ESIMS *m/z* 219 $[M + Na]^+$; HRESIMS *m/z* 219.0641 $[M + Na]^+$ (calcd. for $C_{10}H_{12}O_4Na$, 219.0633).

**Swerimilegenin I (9):** colorless needles; mp 144–145 °C; $[\alpha]_D^{16}$ – 2.4 (*c* 0.1, MeOH); UV (MeOH) $\lambda_{max}$ (log $\varepsilon$) 273 (4.21), 212 (3.66), 191 (3.90) nm; IR (KBr) $\nu_{max}$ 2915, 1700, 1637, 1426, 1304, 1246, 1160, 1124, 1054, 1041 $cm^{-1}$; $^1H$ and $^{13}C$ NMR data, see Table 2; ESIMS *m/z* 233 $[M + Na]^+$; HRESIMS *m/z* 211.0964 $[M + H]^+$ (calcd. for $C_{11}H_{15}O_4$, 211.0970).

**Swerimilegenin J (10):** colorless gum; $[\alpha]_D^{13}$ – 90.6 (*c* 0.1, MeOH); UV (MeOH) $\lambda_{max}$ (log $\varepsilon$) 250 (3.98) nm; IR (KBr) $\nu_{max}$ 3472, 2893, 1679, 1595, 1404, 1272, 1209, 1100, 1041, 962, 760 $cm^{-1}$; $^1H$ and $^{13}C$ NMR data, see Table 3; EIMS *m/z* 199 $[M + H]^+$ (40), 198 $M^+$ (50), 168 (70), 127 (95), 123 (45), 97 (50), 57 (100); HREIMS *m/z* 198.0897 $M^+$ (calcd. for $C_{10}H_{14}O_4$, 198.0892).

**Swerimilegenin K (11):** colorless gum; $[\alpha]_D^{19}$ + 2.2 (*c* 0.2, MeOH); UV (MeOH) $\lambda_{max}$ (log $\varepsilon$) 249 (3.60) nm; IR (KBr) $\nu_{max}$ 3431, 2921, 1695, 1614, 1409, 1277, 1110, 1053 $cm^{-1}$; $^1H$ and $^{13}C$ NMR data, see Table 3; HRESIMS *m/z* 233.0567 $[M + Cl]^-$ (calcd. for $C_{10}H_{14}O_4Cl$, 233.0586).

**Crystallographic Data of Compounds 7 and 9:** Swerimilegenin G (**7**): $C_{18}H_{22}O_7$, MW = 350.14; monoclinic, space group $p2_1$; $a$ = 9.5350(17) Å, $b$ = 8.4098(15) Å, $c$ = 21.881(4) Å, $\beta$ = 101.266(2), $V$ = 1720.8(5) Å$^3$, $Z$ = 7, $d$ = 1.315 g/cm$^3$, crystal dimensions 0.28 × 0.19 × 0.08 mm$^3$ were used for measurement on a Rigaku D/MAX-3B X-ray diffractometer with a graphite monochromater, Mo Kα radiation. The total number of reflections measured was 10830, of which 4045, were observed, $I > 2\sigma(I)$. Final indices: $R_1$= 0.0523, w$R_2$ = 0.1341.

Swerimilegenin I (**9**): $C_{11}H_{14}O_4$, MW = 210.22; monoclinic, space group $p2_1$; $a$ = 4.287(2) Å, $b$ = 9.320(5) Å, $c$ = 27.001(14) Å, $\beta$ = 100.268(9), $V$ = 1061.6(9) Å$^3$, $Z$ = 2, $d$ = 1.315 g/cm$^3$, crystal dimensions 0.20 × 0.13 × 0.06 mm$^3$ were used for measurement on a Rigaku D/MAX-3B X-ray diffractometer with a graphite monochromater, Mo Kα radiation. The total number of reflections measured was 5809, of which 1253, were observed, $I > 2\sigma(I)$. Final indices: $R_1$= 0.1011, w$R_2$ = 0.4433.

**Anti-HBV Assay.** The anti-HBV procedure was performed in accordance with our previous report.[15] Tenofovir (Lot No. 200904009, purity > 97.6%) was used as the positive control, which was purchased from Jiangxi Chenyang Pharmaceutical Co. Ltd..

## Electronic Supplementary Material

Supplementary material is available in the online version of this article at http://dx.doi.org/10.1007/s13659-013-0059-y and is accessible for authorized users.

## Acknowledgments

This work was funded by the National Natural Science Foundation of China and Yunnan Province (U0832603), the National Science Foundation of China for Distinguished Young Scholars (81025023), the West Light Foundation of the Chinese Academy of Sciences, the International Foundation for Science (No. F/5202-1), and the Youth Innovation Promotion Association, CAS.

**Open Access** This article is distributed under the terms of the Creative Commons Attribution License which permits any use, distribution, and reproduction in any medium, provided the original author(s) and source are credited.

## Electronic supplementary material

Supplementary material, approximately 21.5 MB.

Supplementary material, approximately 16.4 MB.

Supplementary material, approximately 29.8 MB.
