# Supplementary material for: Minor secoiridoid aglycones from the low-polarity part of the traditional Chinese herb: Swertia mileensis
Source: Nat Prod Bioprospect. 2013 Oct 16;3(5):243–9. doi: 10.1007/s13659-013-0059-y (PMC4131618; doi:10.1007/s13659-013-0059-y)

Electronic Supplementary Material

Minor secoiridoid aglycones from the low-polarity part of the traditional Chinese herb: *Swertia mileensis*

Chang-An GENG, Xue-Mei ZHANG, Yun-Bao MA, Xiao-Yan HUANG, and Ji-Jun CHEN*

State Key Laboratory of Phytochemistry and Plant Resources in West China, Kunming Institute of Botany, Chinese Academy of Sciences, Kunming 650201, China

Received 20 July 2013; Accepted 8 September 2013

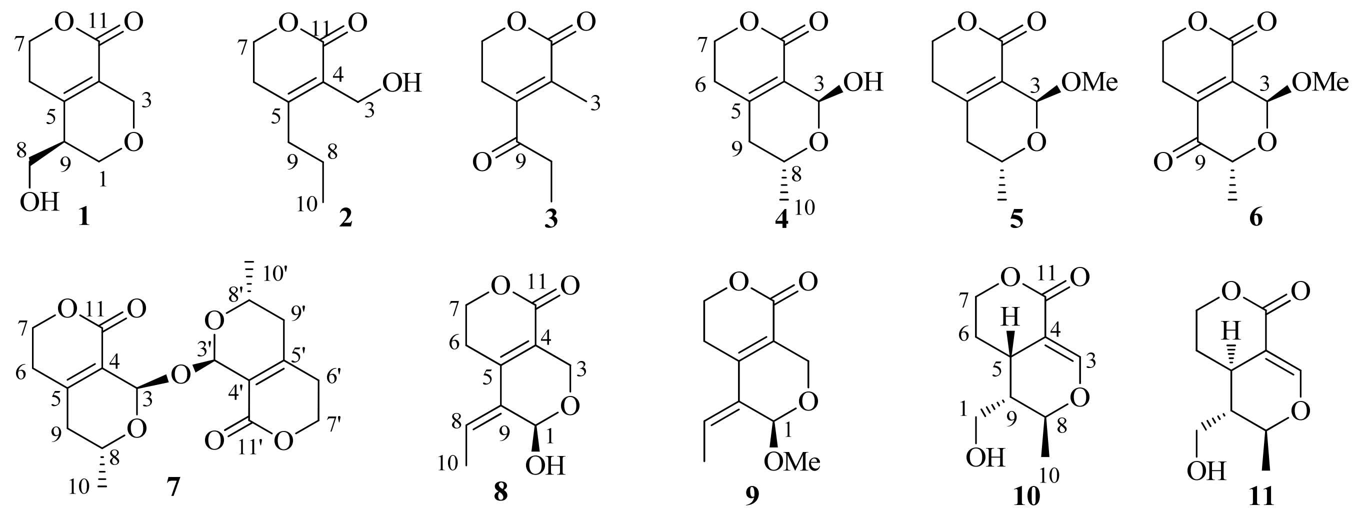

Structures of compounds **1**–**11**

*To whom correspondence should be addressed. E-mail: chenjj@mail.kib.ac.cn

The ^{1}H NMR spectrum of compound **1** in $CDCl_3$

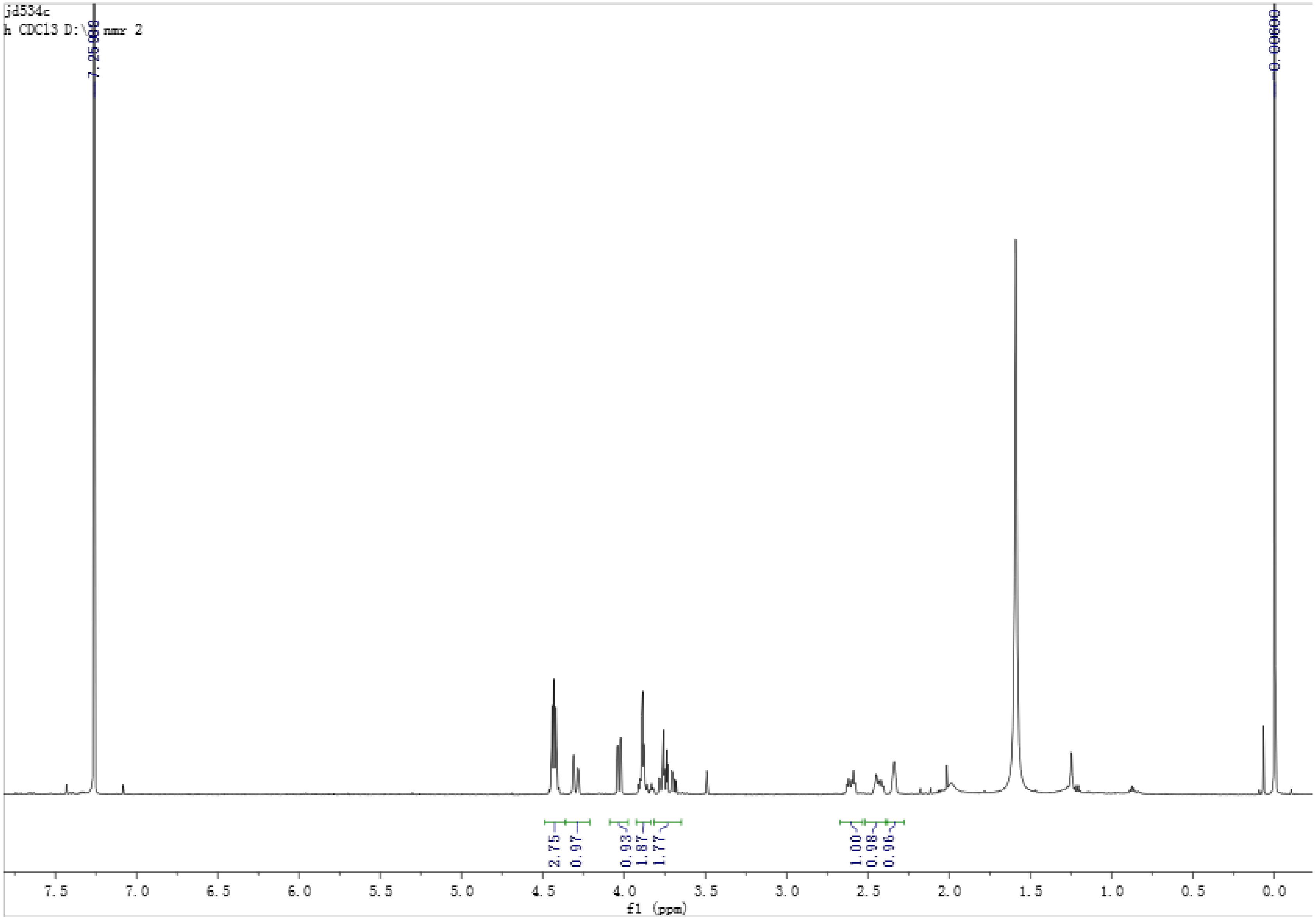

The ^{13}C NMR (DEPT) spectrum of compound **1** in $CDCl_3$

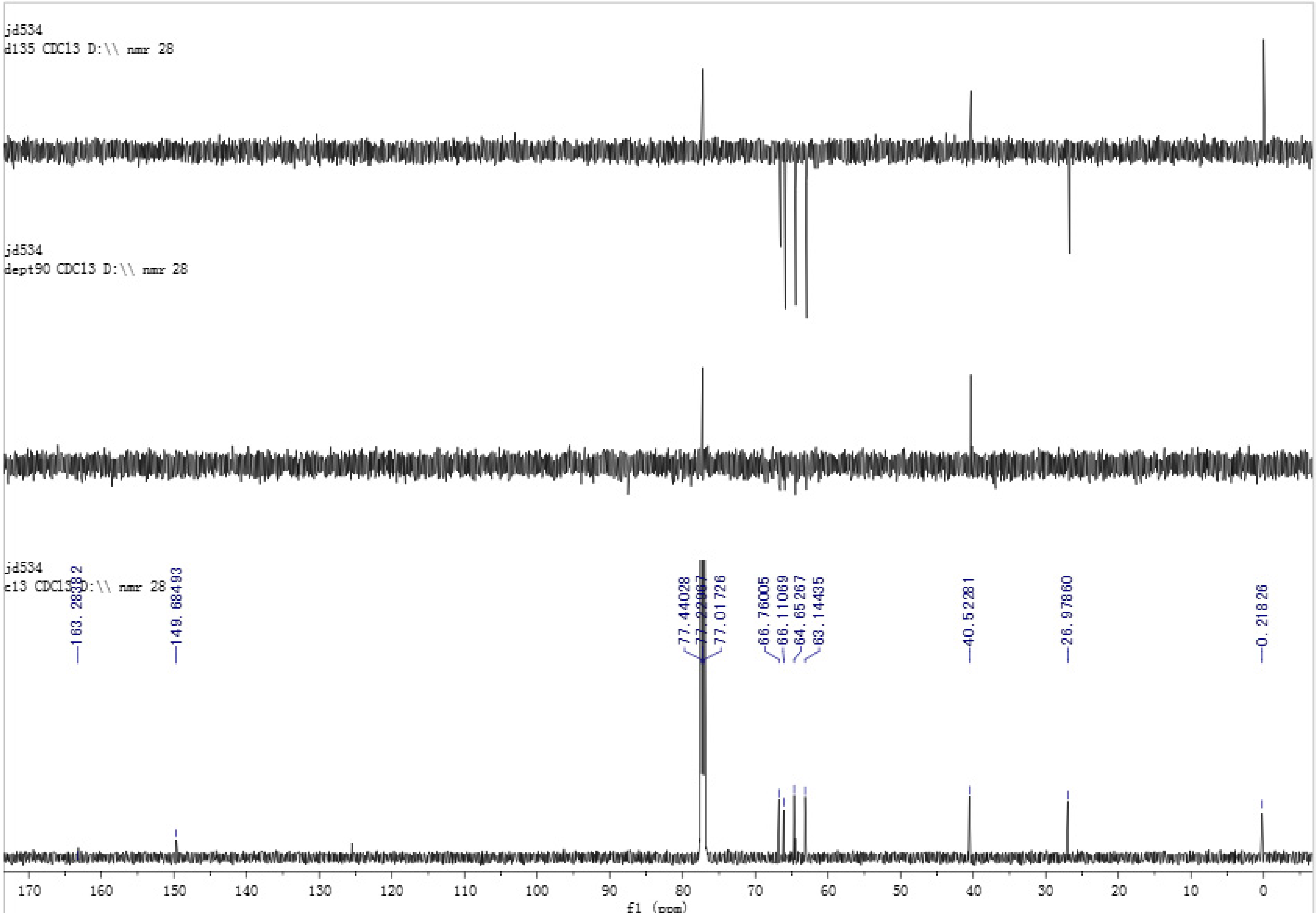

The HSQC spectrum of compound **1** in $CDCl_3$

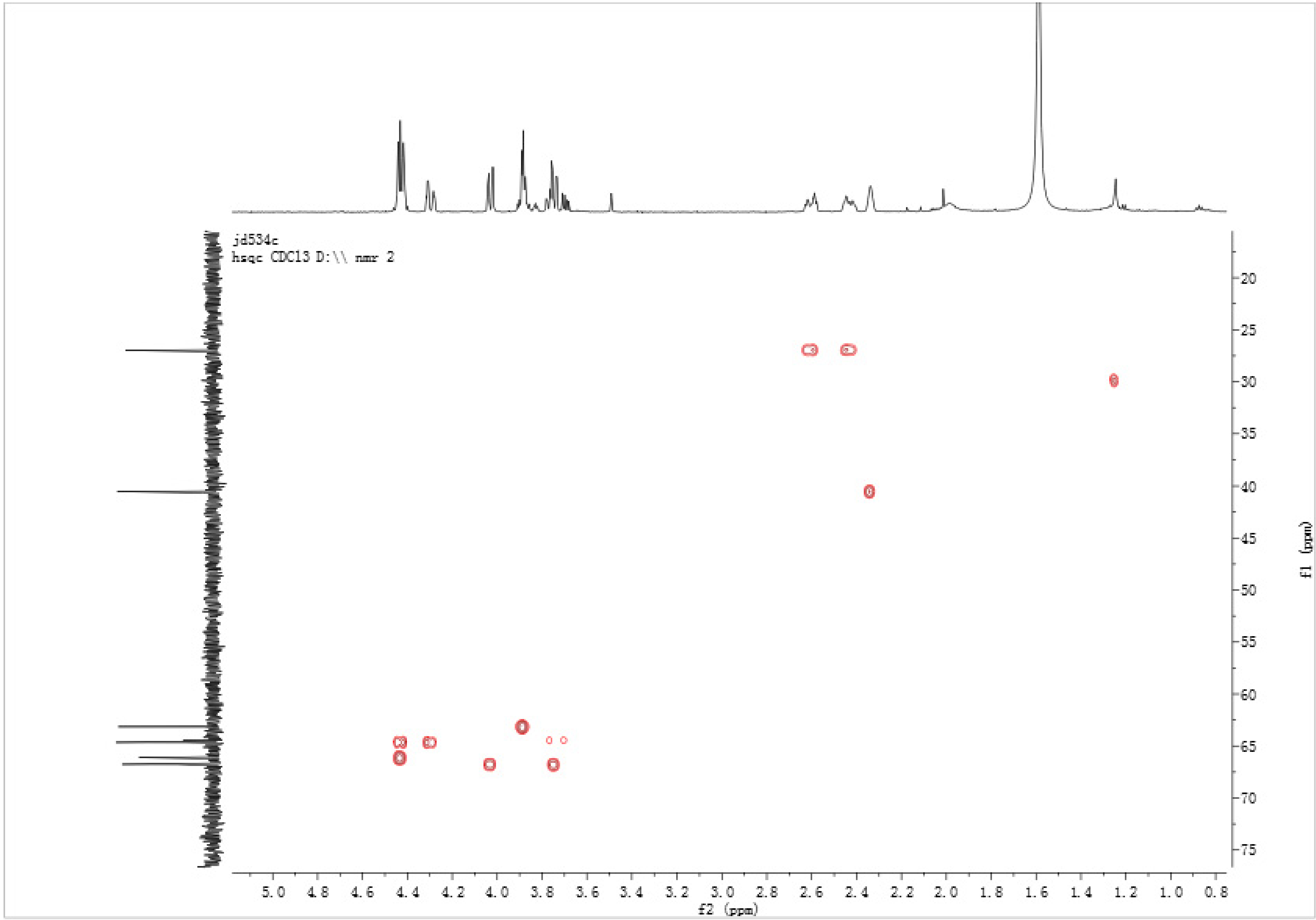

The HMBC spectrum of compound **1** in $CDCl_3$

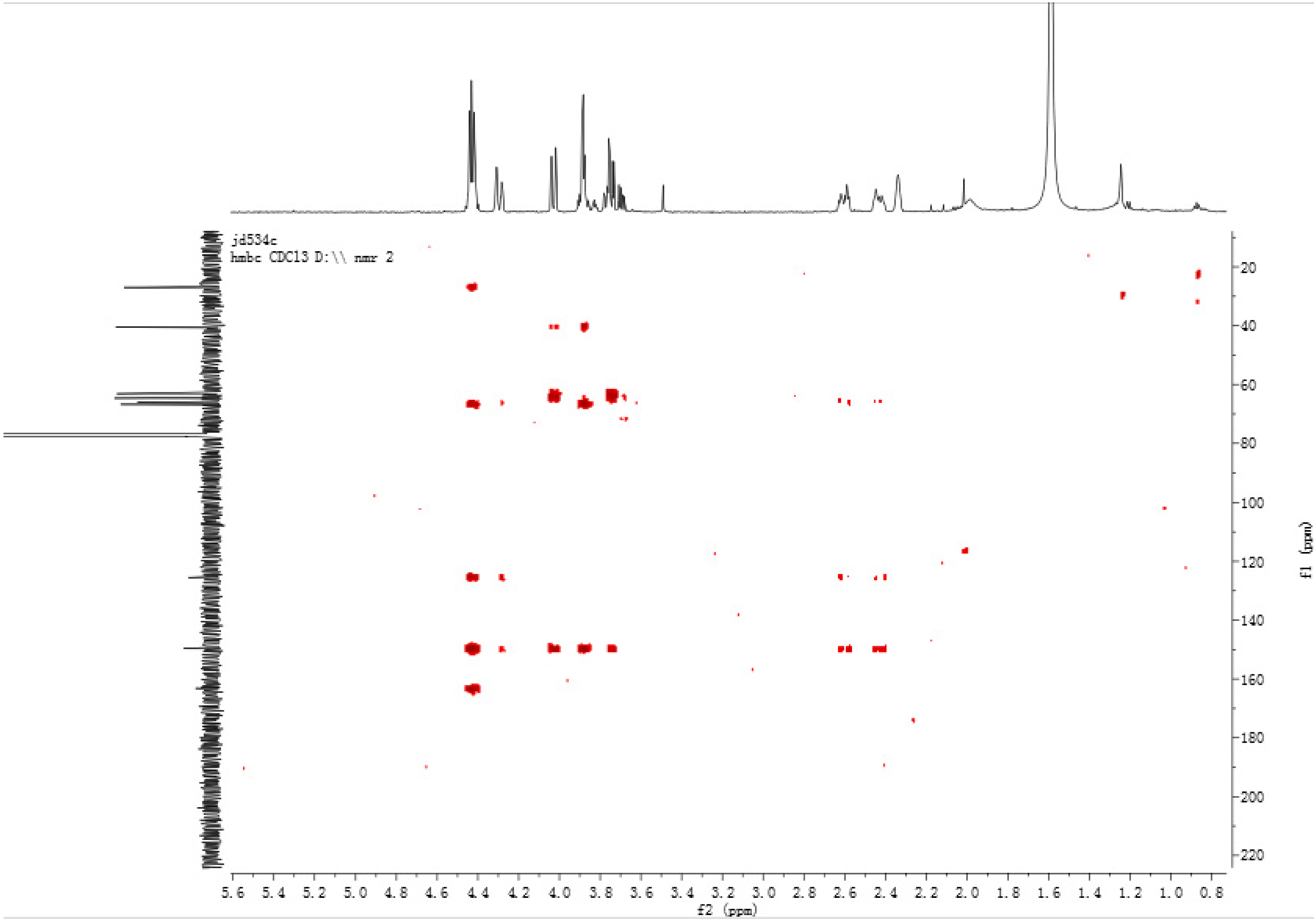

The ^{1}H ^{1}H COSY spectrum of compound **1** in $CDCl_3$

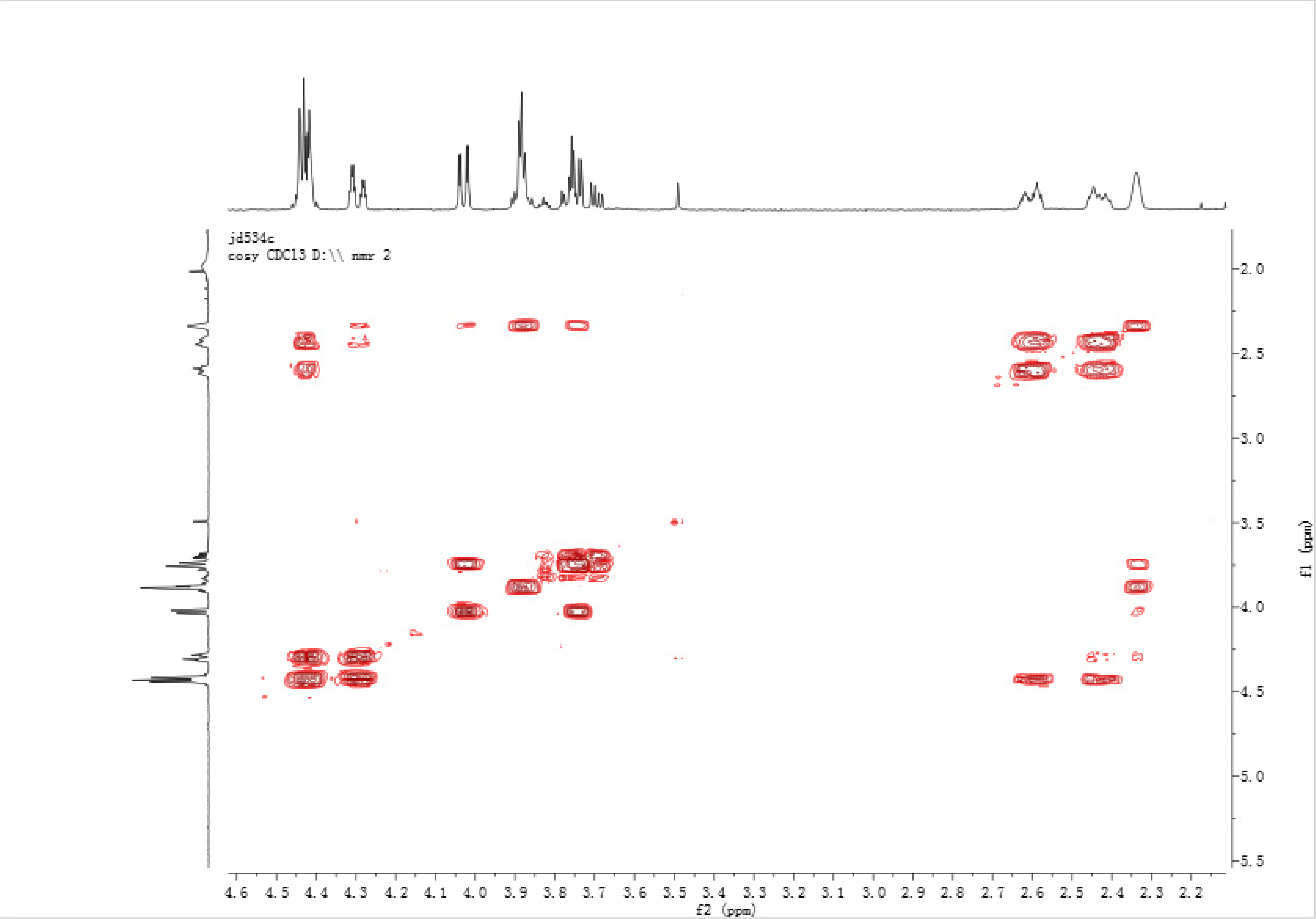

The ROESY spectrum of compound **1** in $CDCl_3$

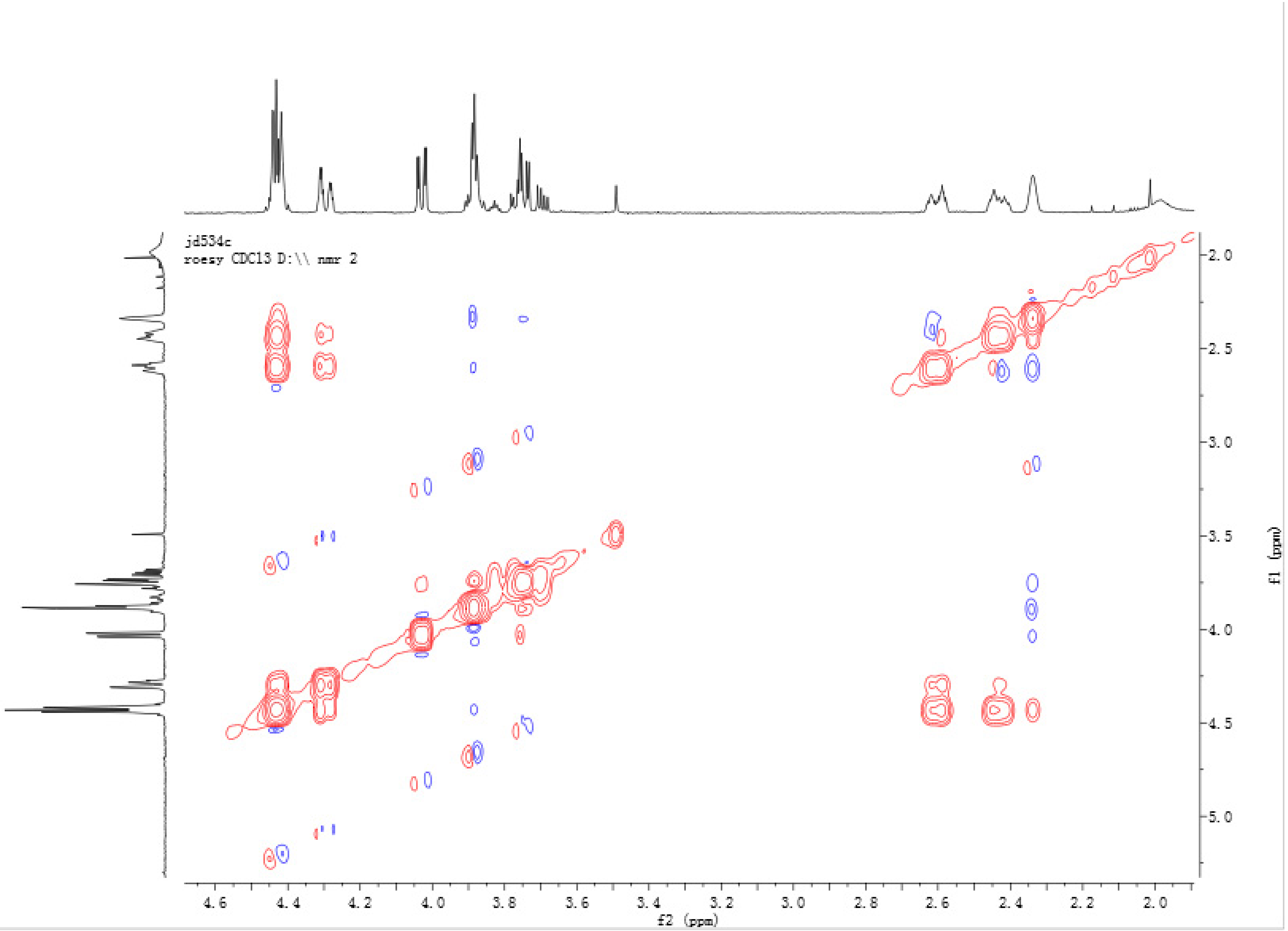

The HRESIMS spectrum of compound **1**

Formula Predictor Report - JD534-3.lcd

Page 1 of 1

Data File: D:\Gavin\Data\JD534-3.lcd

Elmt	Val.	Min	Max	Elmt	Val.	Min	Max	Elmt	Val.	Min	Max	Use Adduct
H	1	0	100	O	2	0	40	S	2	0	0	H
C	4	0	100	Na	1	0	0	Cl	1	0	0	Na
N	3	0	0	P	3	0	0	Br	1	0	0	

Error Margin (ppm): 20
HC Ratio: unlimited
Max Isotopes: all
MSn Iso RI (%): 75.00

DBE Range: 0.0 - 30.0
Apply N Rule: yes
Isotope RI (%): 1.00
MSn Logic Mode: AND

Electron Ions: both
Use MSn Info: no
Isotope Res: 10000
Max Results: 500

Event#: 1 MS(C+) Ret. Time : 1.053 -> 1.107 - 1.133 -> 1.169 Scan# : 159 -> 167 - 171 -> 177

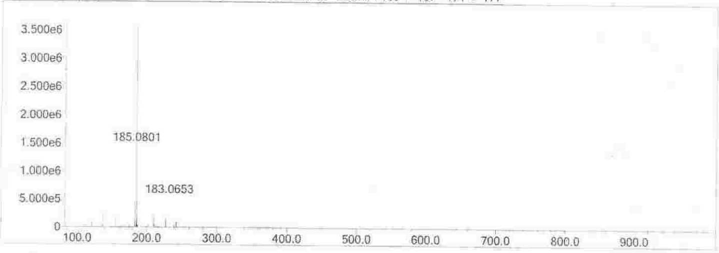

Measured region for 185.0801 m/z

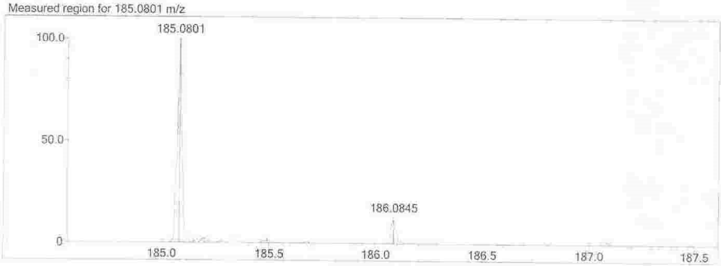

C9 H12 O4 [M+H]+ : Predicted region for 185.0808 m/z

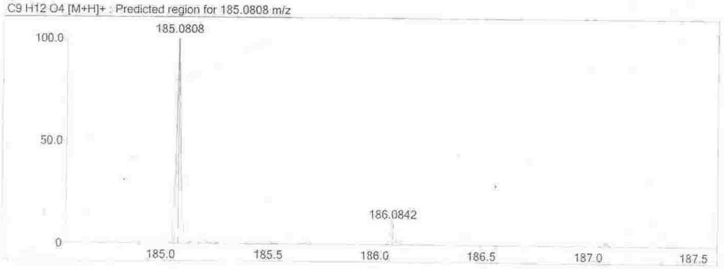

Rank	Score	Ion	Formula (M)	Pred. m/z	Meas. m/z	Df. (mDa)	Df. (ppm)	Iso	DBE
1	70.97	[M+H]+	C9 H12 O4	185.0808	185.0801	-0.7	-3.78	76.27	4.0

Formula Predictor Report - JD534-3.lcd

Page 1 of 1

Data File: D:\Gavin\Data\JD534-3.lcd

Elmt	Val.	Min	Max	Elmt	Val.	Min	Max	Elmt	Val.	Min	Max	Use Adduct
H	1	0	100	O	2	0	40	S	2	0	0	H
C	4	0	100	Na	1	0	0	Cl	1	0	0	Cl
N	3	0	0	P	3	0	0	Br	1	0	0	

Error Margin (ppm): 20
HC Ratio: unlimited
Max Isotopes: all
MSn Iso RI (%): 75.00

DBE Range: 0.0 - 30.0
Apply N Rule: yes
Isotope RI (%): 1.00
MSn Logic Mode: AND

Electron Ions: both
Use MSn Info: no
Isotope Res: 10000
Max Results: 500

Event#: 2 MS(C-) Ret. Time : 1.053 -> 1.107 - 1.133 -> 1.169 Scan# : 160 -> 168 - 172 -> 178

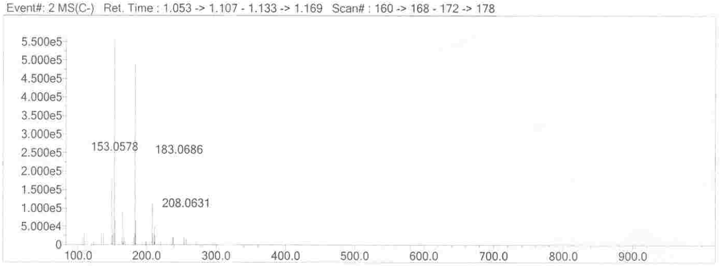

Measured region for 183.0686 m/z

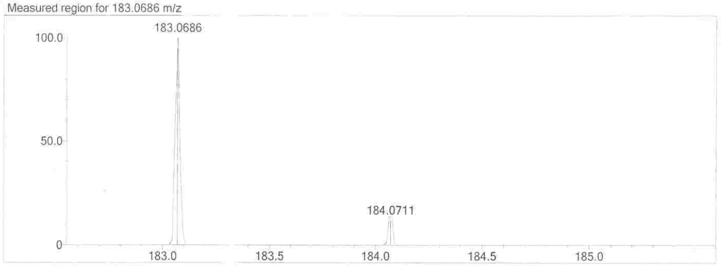

C9 H12 O4 [M-H]- : Predicted region for 183.0663 m/z

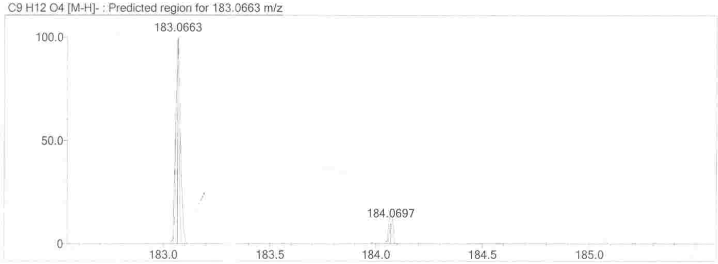

Rank	Score	Ion	Formula (M)	Pred. m/z	Meas. m/z	Df. (mDa)	Df. (ppm)	Iso	DBE
1	14.66	[M-H]-	C9 H12 O4	183.0663	183.0686	2.3	12.56	44.19	4.0

The IR spectrum of compound **1**

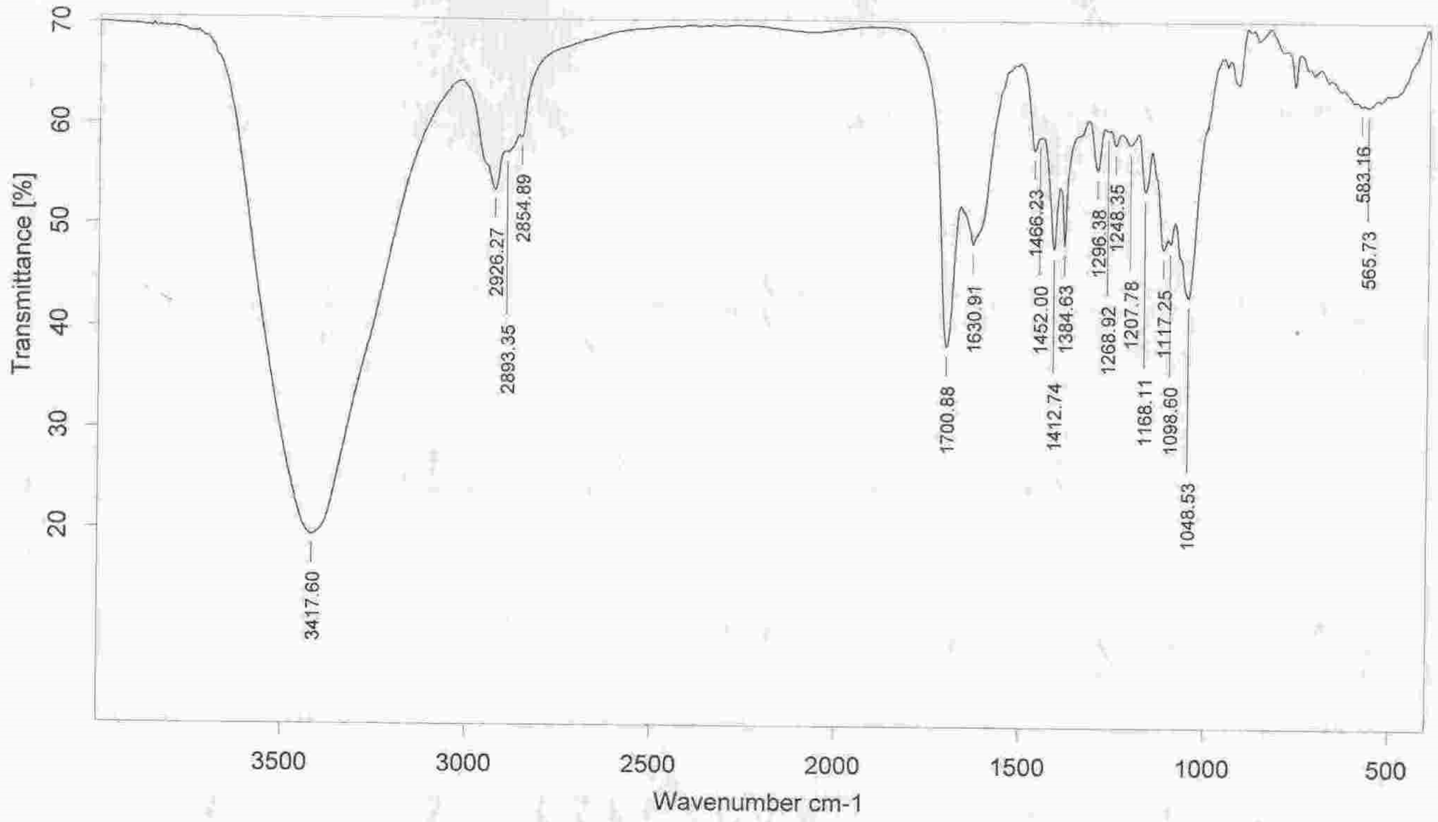

Sample : JD534C	Frequency Range : 399.246 - 3996.32	Measured on : 14/03/2012	
Technique : KBr压片	Resolution : 4	Instrument : Tensor27	Sample Scans : 16
Customer : 120314IR1	Zerofilling : 2	Acquisition : Double Sided,For	

The UV spectrum of compound **1**

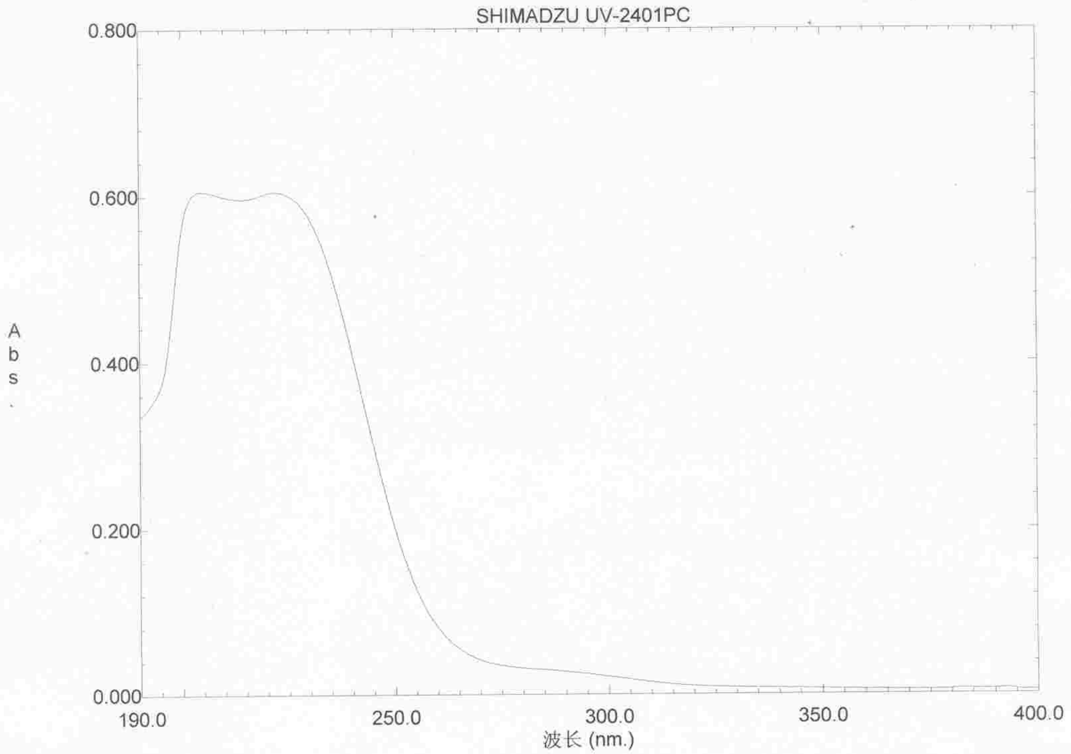

文件名: JD534C

JD534C

样品浓度：0.0320毫克/毫升
溶剂：甲醇

创建于: 15:25 12-03-12
数据: 原始

测量模式: Abs.
扫描速度: 中速
狭缝: 5.0
采样间隔: 0.2

否.	波长 (nm.)	Abs.
1	392.80	0.0063
2	222.00	0.6049
3	204.80	0.6056

The $[\alpha]_D$ spectrum of compound **1**

Optical rotation measurement

Model : P-1020 (A060460638)

No.	Sample	Mode	Data	Monitor<br>Blank	Temp.<br>Cell<br>Temp Point	Date<br>Comment<br>Sample Name	Light<br>Filter<br>Operator	Cycle Time<br>Integ Time
No.1	3 (1/3)	Sp.Rot	-11.8750	-0.0038<br>0.0000	18.5<br>50.00<br>Cell	Mon Mar 12 15:11:38 2012<br>0.00064g/mlMeOH<br>JD534C	Na<br>589nm	2 sec<br>10 sec
No.2	3 (2/3)	Sp.Rot	-12.8130	-0.0041<br>0.0000	18.5<br>50.00<br>Cell	Mon Mar 12 15:11:51 2012<br>0.00064g/mlMeOH<br>JD534C	Na<br>589nm	2 sec<br>10 sec
No.3	3 (3/3)	Sp.Rot	-12.5000	-0.0040<br>0.0000	18.5<br>50.00<br>Cell	Mon Mar 12 15:12:05 2012<br>0.00064g/mlMeOH<br>JD534C	Na<br>589nm	2 sec<br>10 sec

-12.3958°

The ^{1}H NMR spectrum of compound **2** in $CDCl_3$

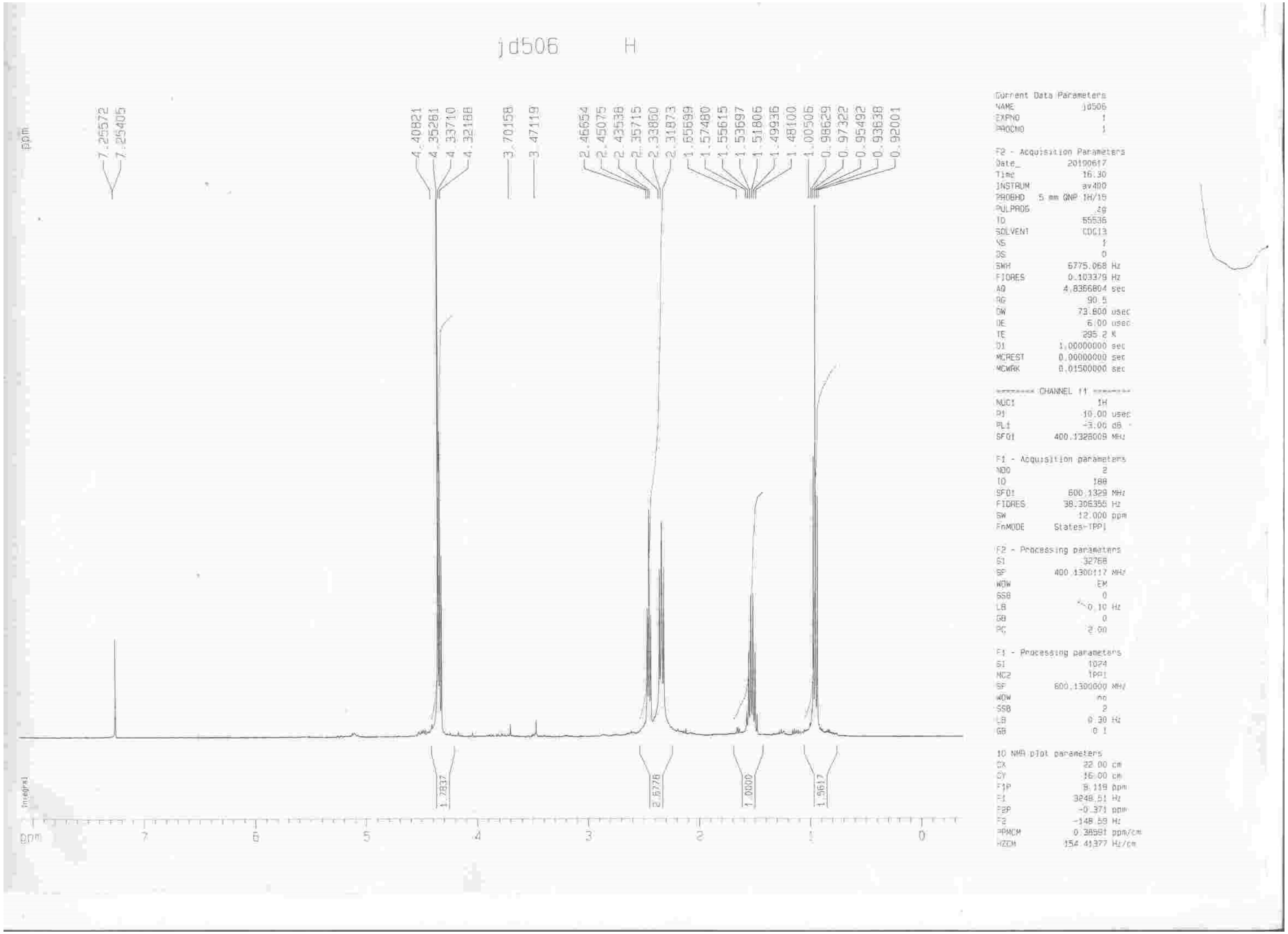

The ^{13}C NMR (DEPT) spectrum of compound **2** in $CDCl_3$

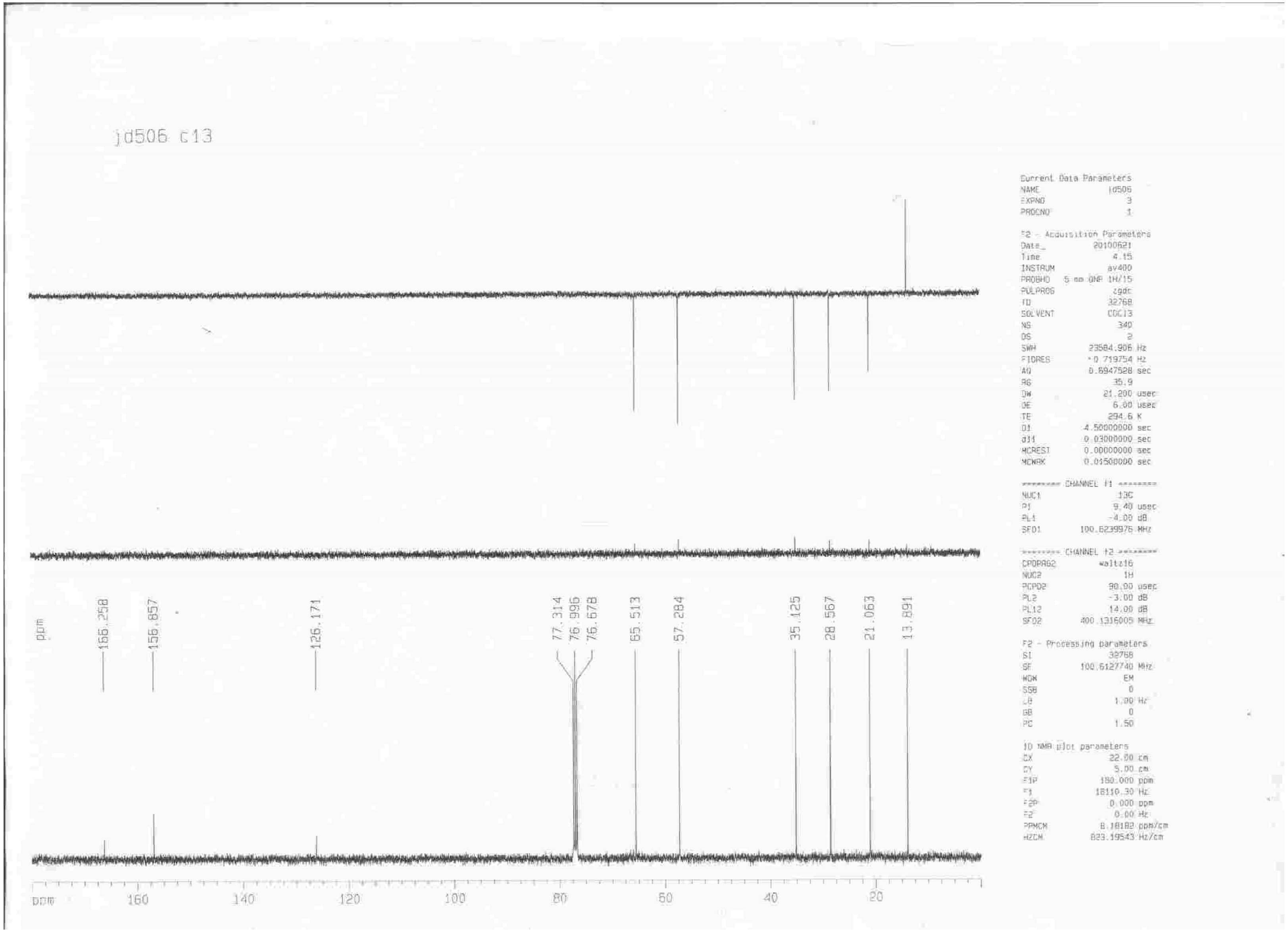

The HSQC spectrum of compound **2** in $CDCl_3$

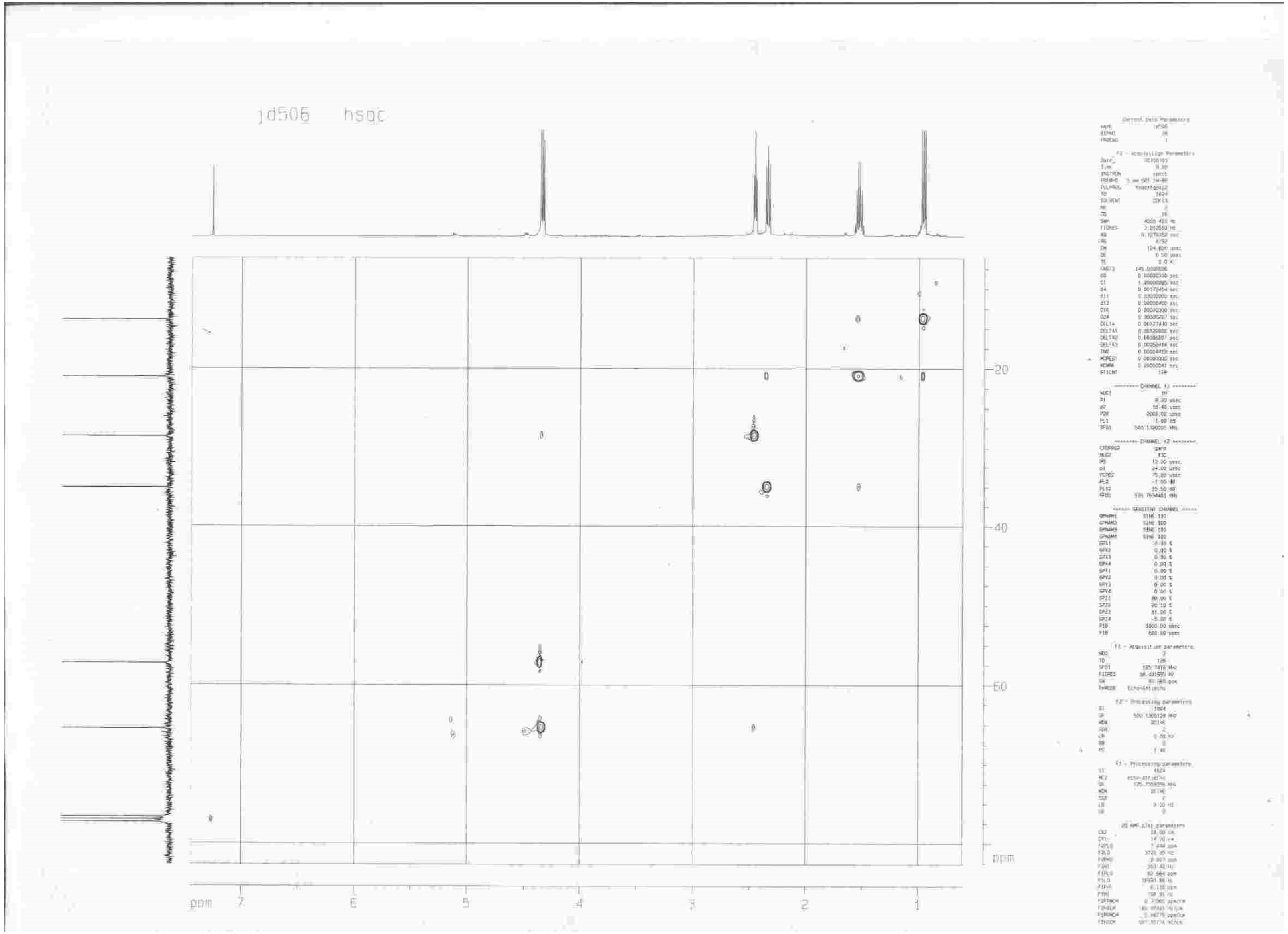

The HMBC spectrum of compound **2** in $CDCl_3$

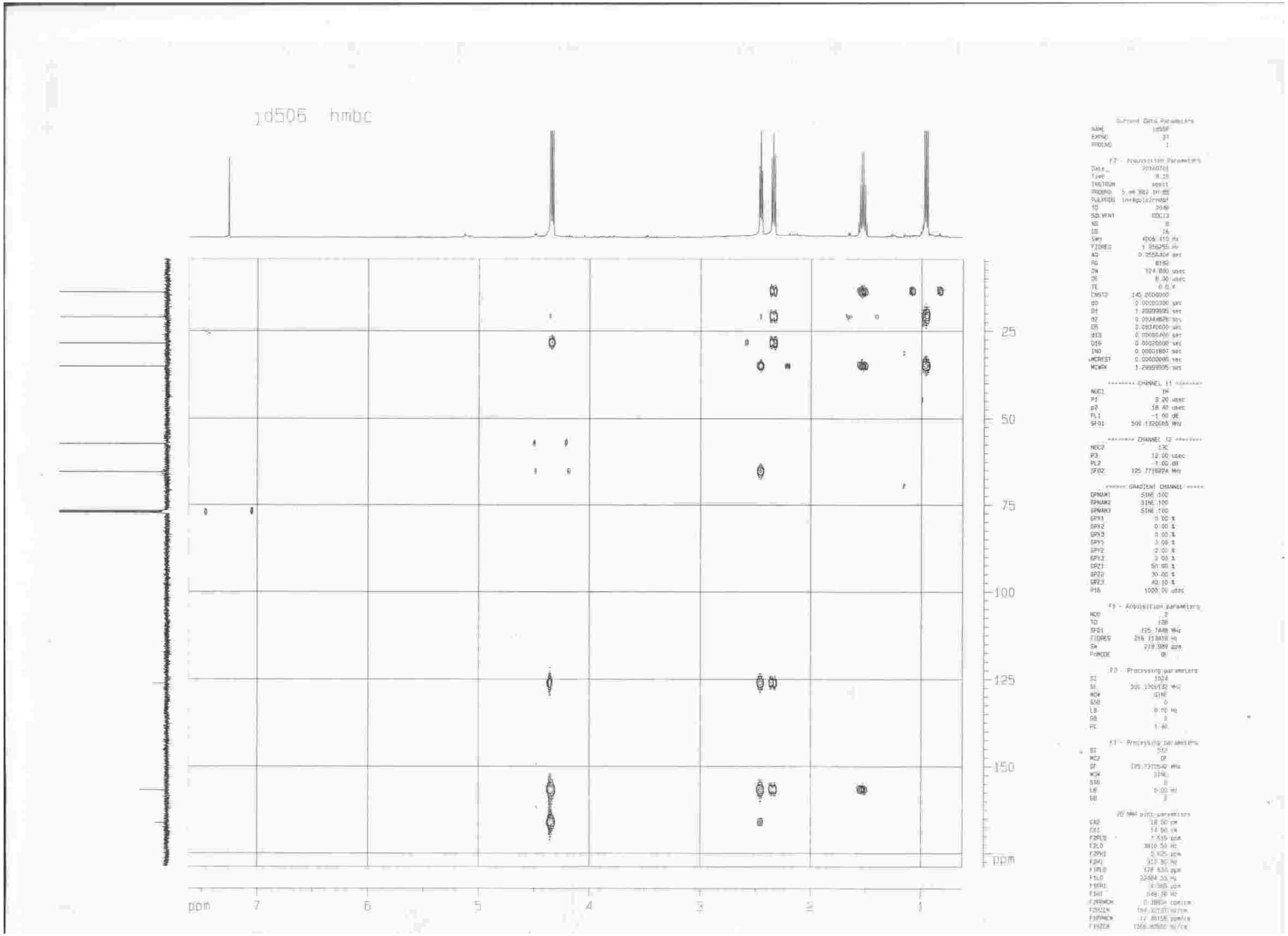

The ^1H ^1H COSY spectrum of compound **2** in $CDCl_3$

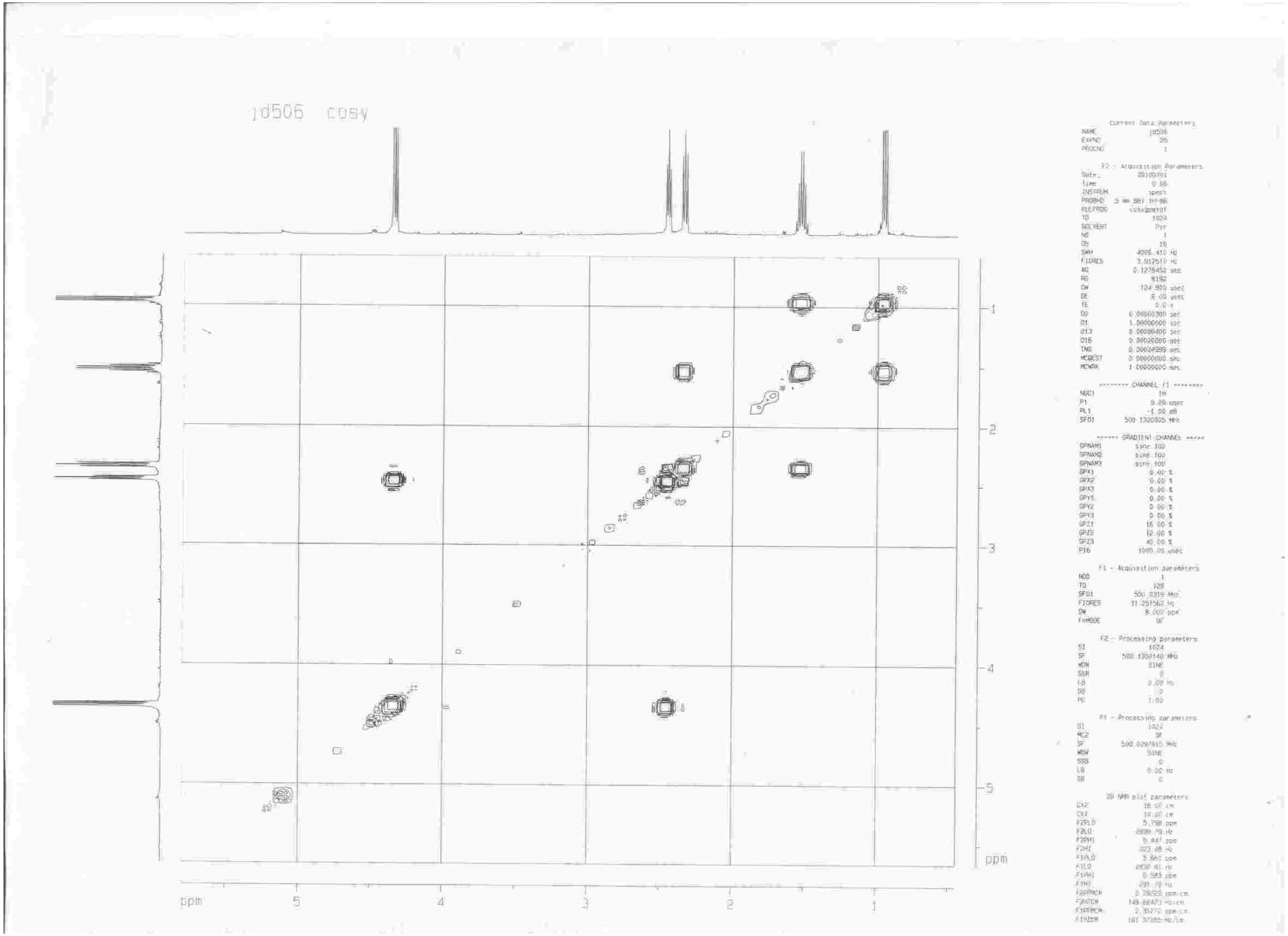

The HREIMS spectrum of compound **2**

Elemental Composition Report

Page 1

Single Mass Analysis (displaying only valid results)
Tolerance = 10.0 PPM / DBE: min = 0.5, max = 40.0
Selected filters: None

Monoisotopic Mass, Odd and Even Electron Ions
9 formula(e) evaluated with 1 results within limits (up to 51 closest results for each mass)
Elements Used:
C: 0-200 H: 0-400 O: 0-3

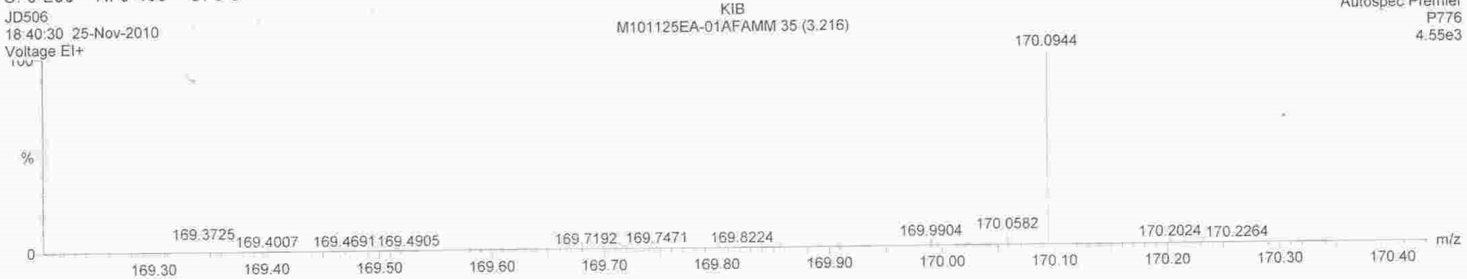

Minimum:				0.5		
Maximum:		100.0	10.0	40.0		
Mass	Calc. Mass	mDa	PPM	DBE	i-FIT	Formula
170.0944	170.0943	0.1	0.6	3.0	5548244.5	C9 H14 O3

The IR spectrum of compound **2**

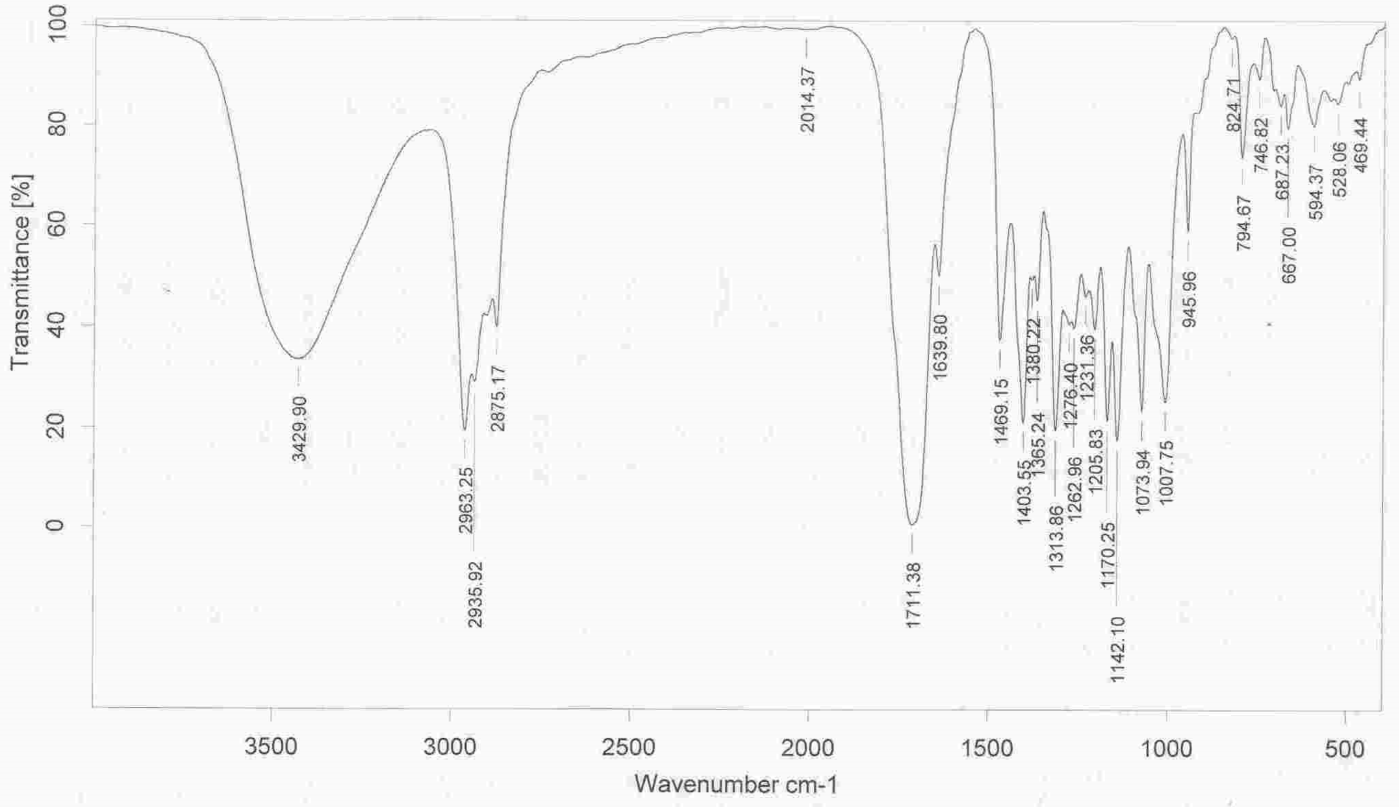

Sample : JD506	Frequency Range : 399.271 - 3996.57	Measured on : 07/03/2011	
Technique : KBr压片	Resolution : 4	Instrument : Tensor27	Sample Scans : 16
Customer : 110307IR	Zerofilling : 2	Acquisition : Double Sided,For	

The UV spectrum of compound **2**

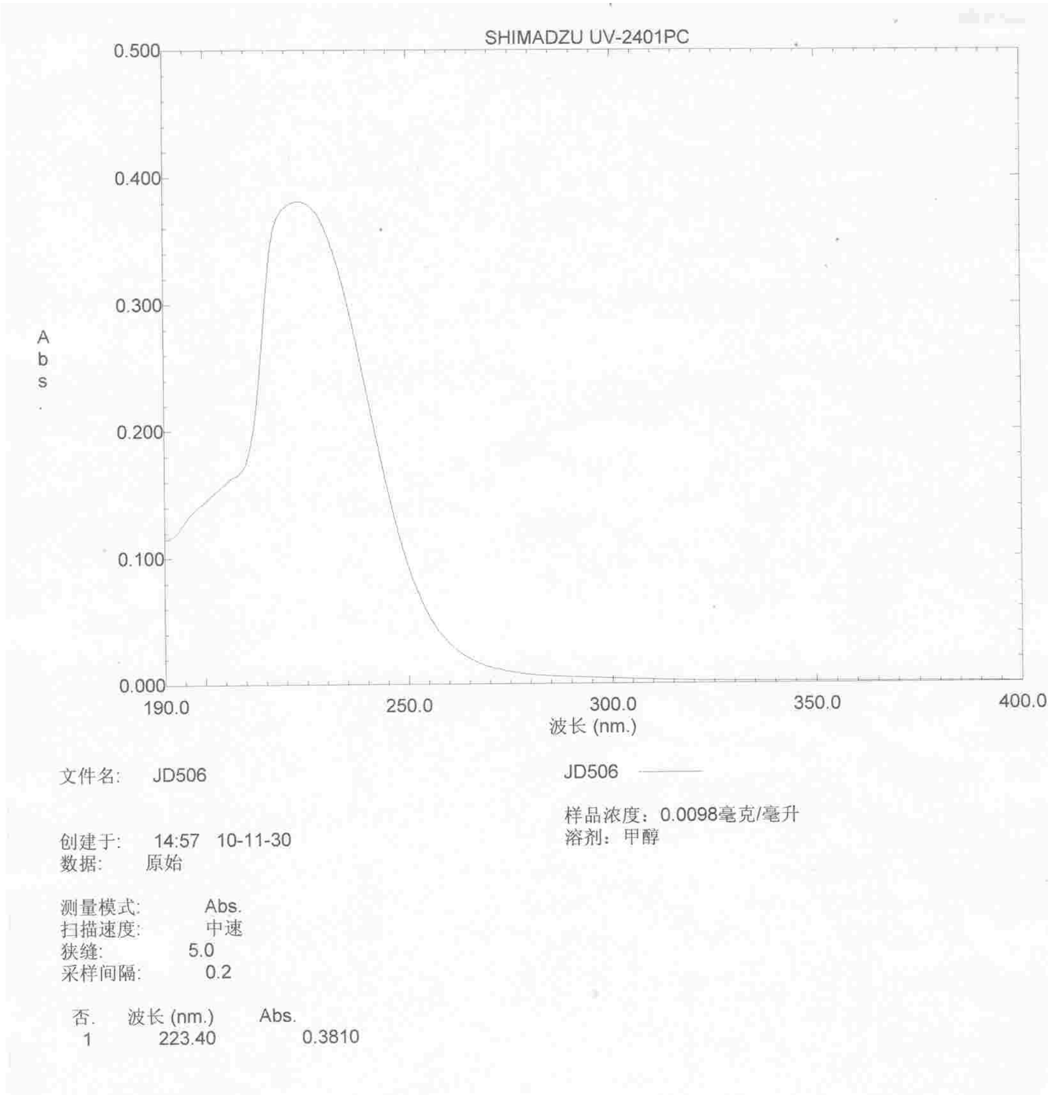

The ^{1}H NMR spectrum of compound **3** in $CDCl_3$

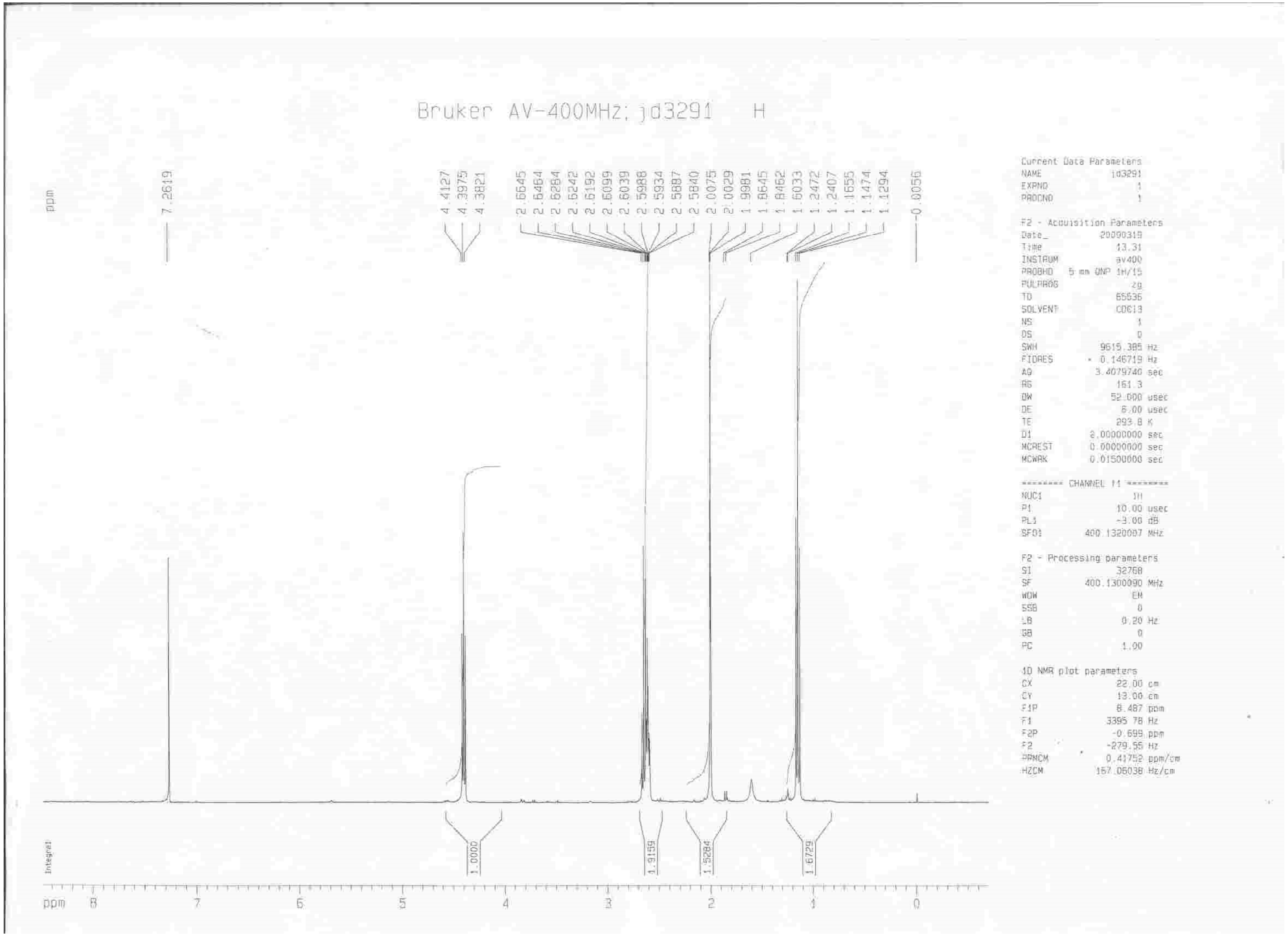

The ^{13}C NMR spectrum of compound **3** in $CDCl_3$

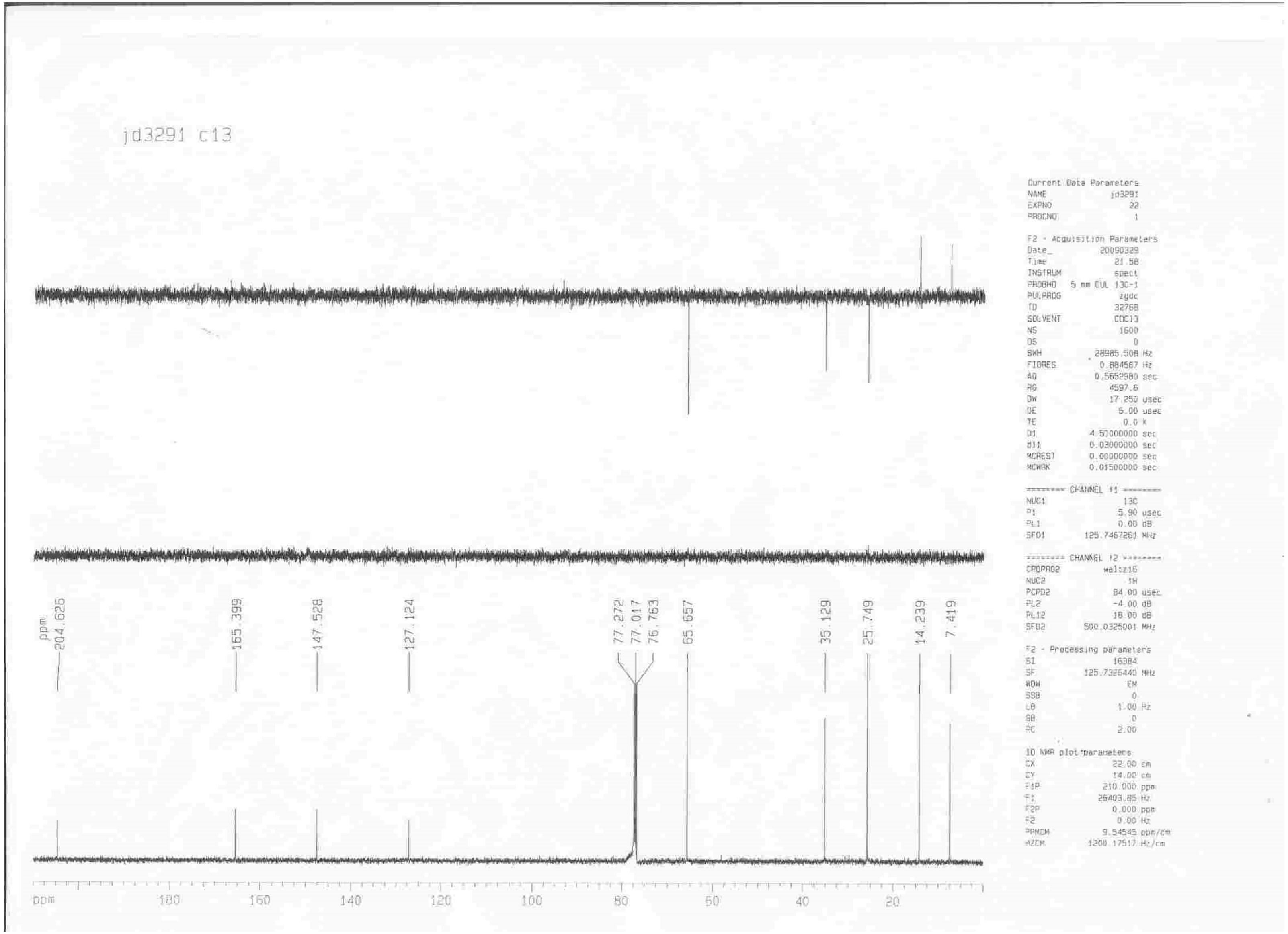

The HSQC spectrum of compound **3** in $CDCl_3$

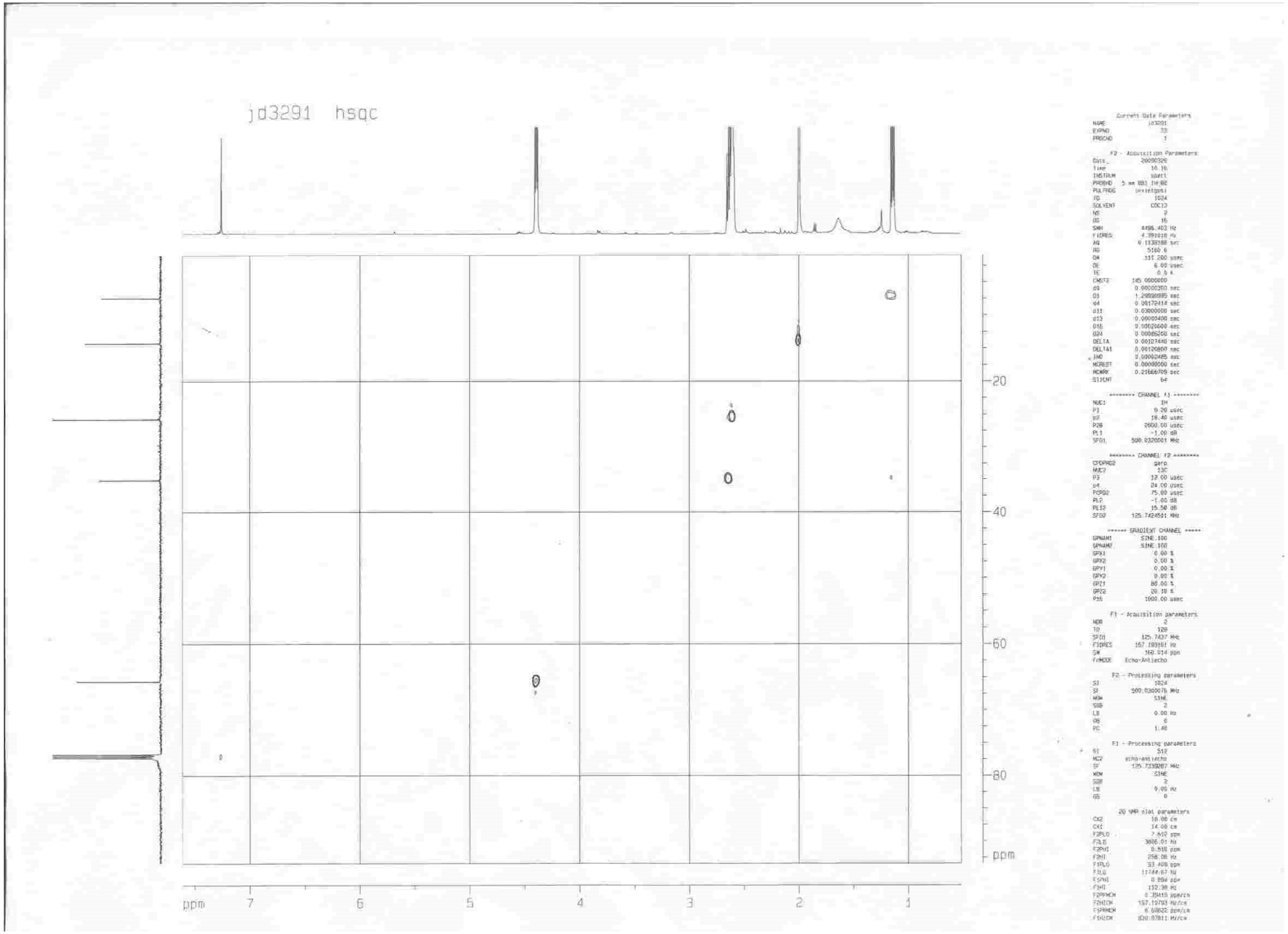

The HMBC spectrum of compound **3** in $CDCl_3$

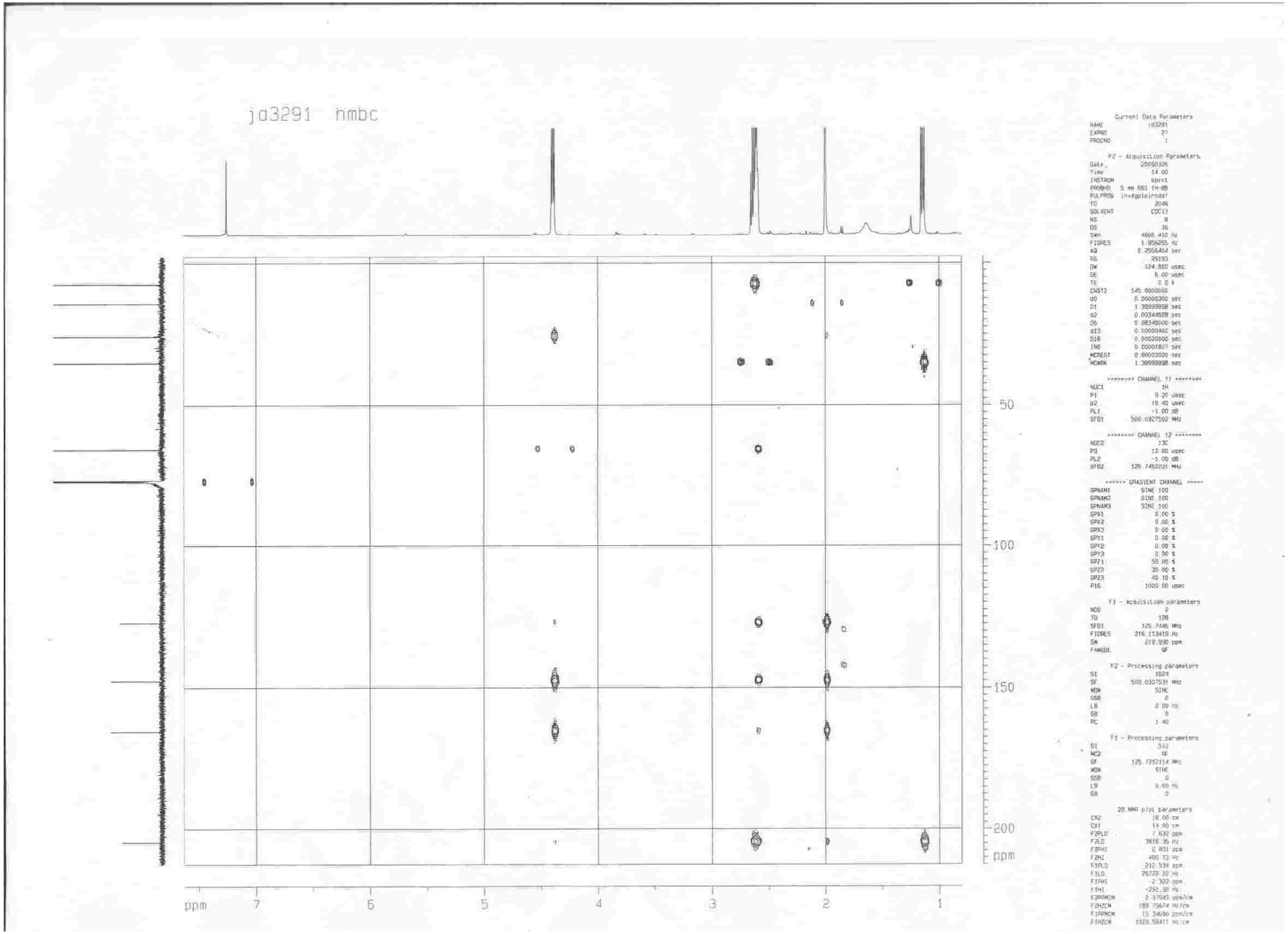

The EIMS spectrum of compound **3**

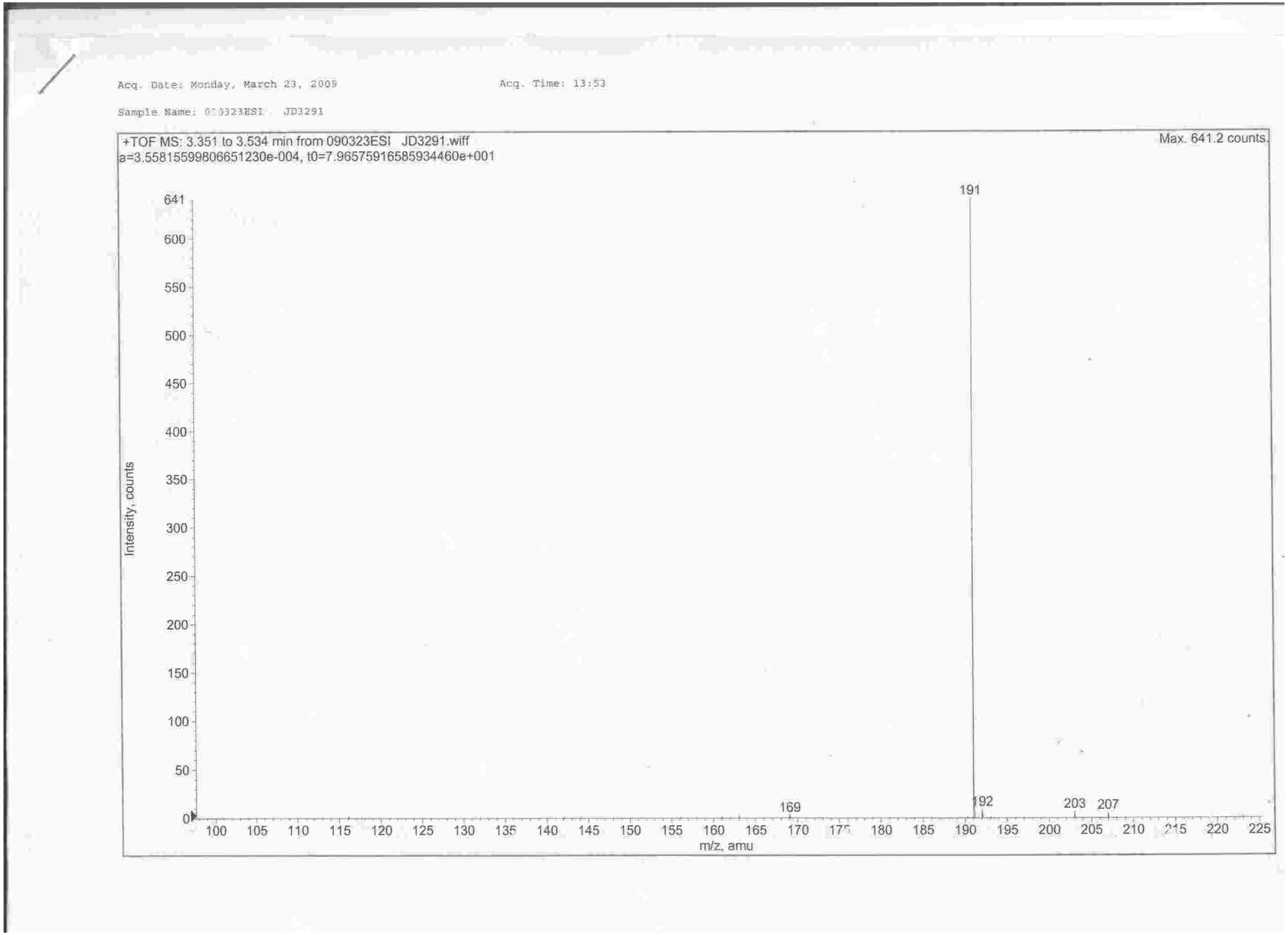

The HRESIMS spectrum of compound **3**

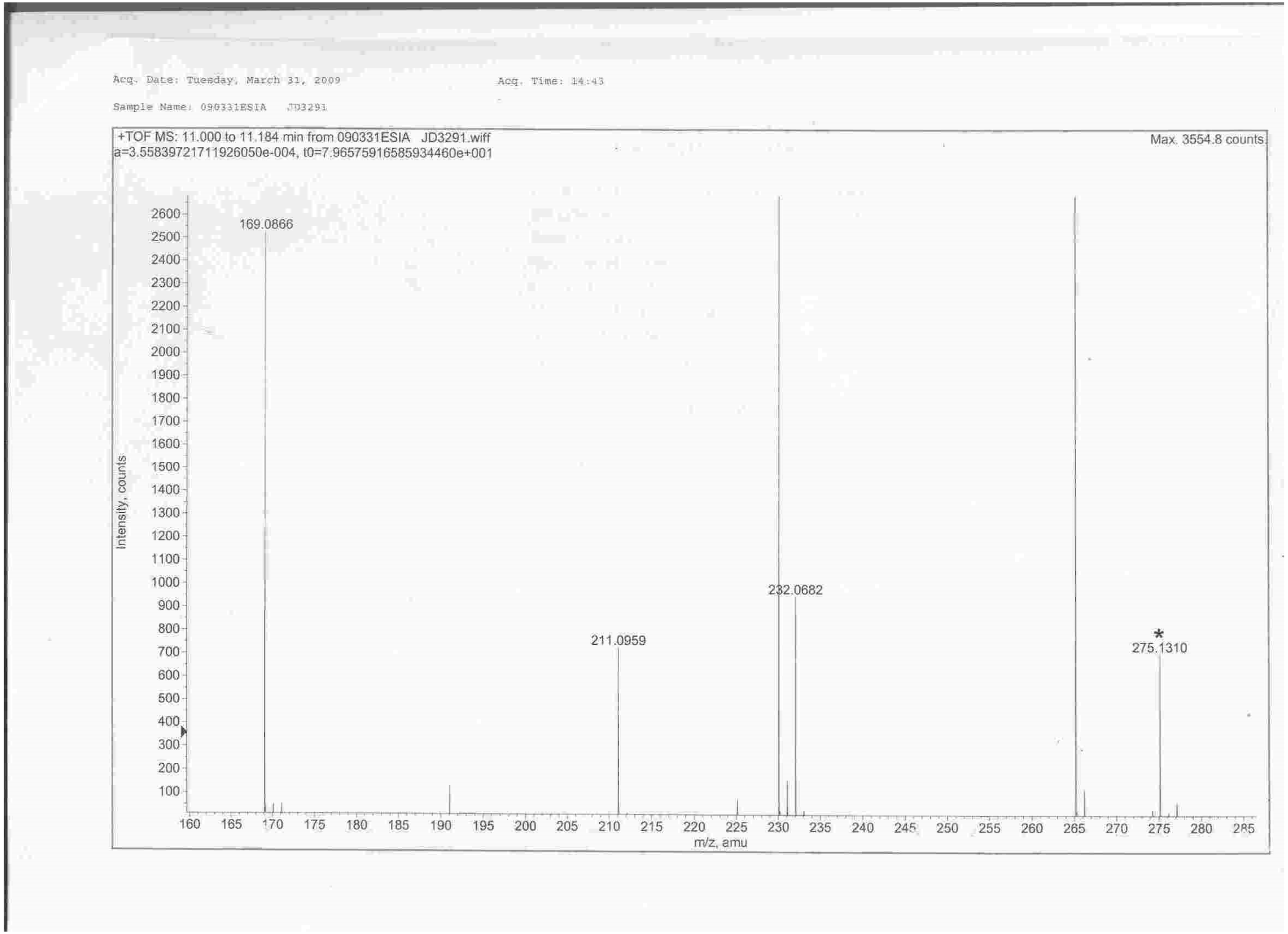

The IR spectrum of compound **3**

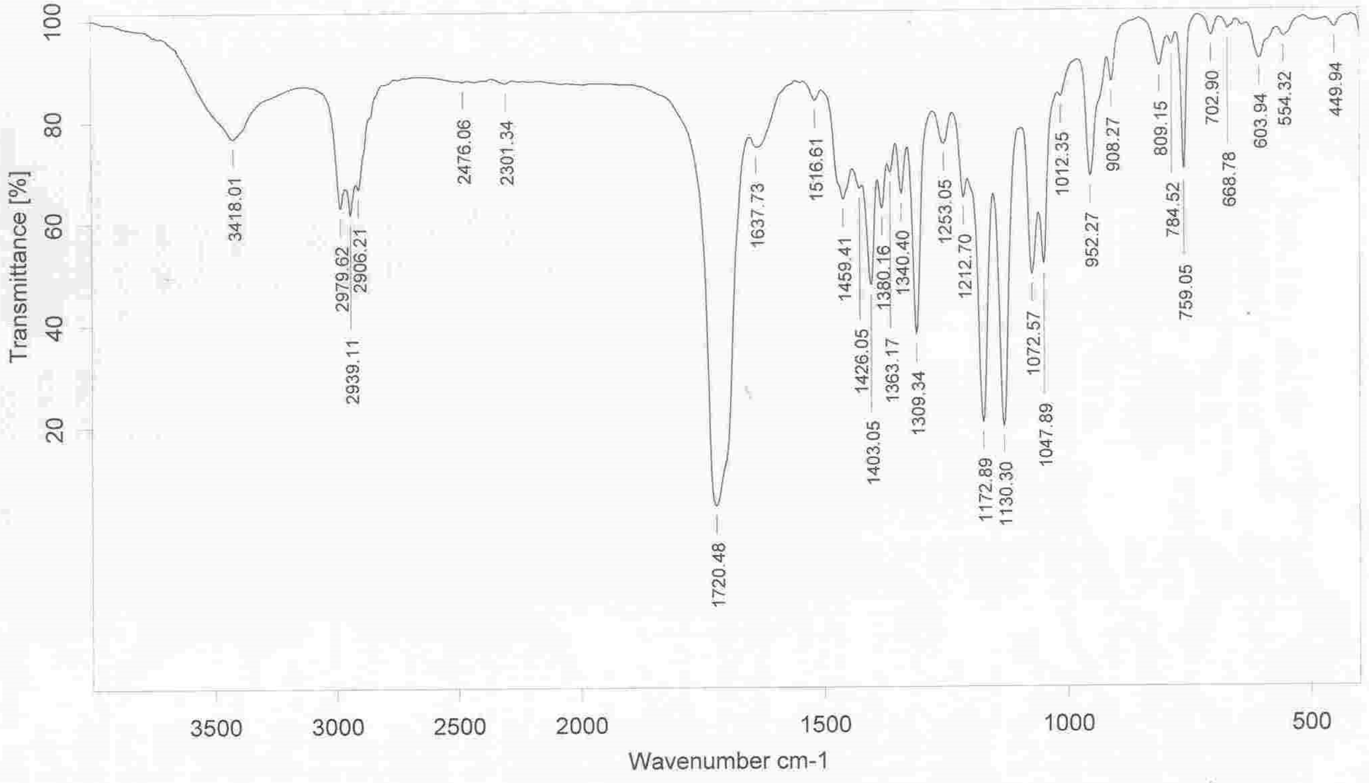

Sample : JD3291	Frequency Range : 399.271 - 3996.57		Measured on : 14/04/2009
Technique : KBr压片	Resolution : 4	Instrument : Tensor27	Sample Scans : 16
Customer : 090414IR4	Zerofilling : 2	Acquisition : Double Sided,For	

The UV spectrum of compound **3**

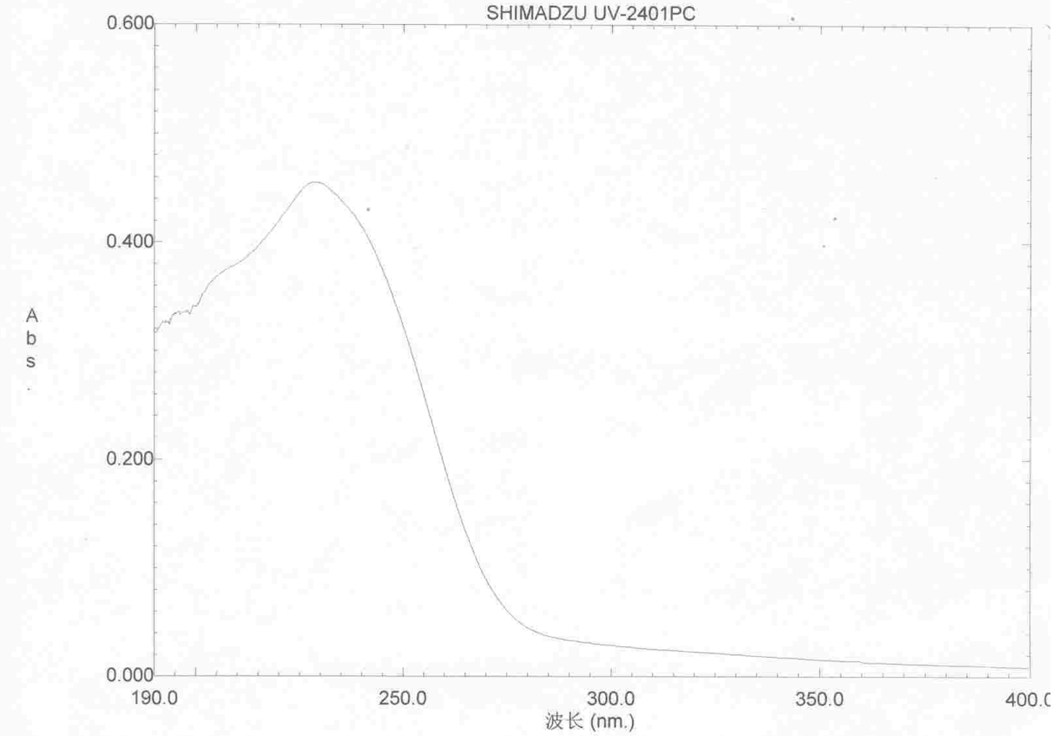

文件名:　JD3291

JD3291 ———

样品浓度：0.0113毫克/毫升
溶剂：甲醇

创建于:　11:50　09-04-07
数据:　原始

测量模式:　Abs.
扫描速度:　中速
狭缝:　2.0
采样间隔:　0.2

否.	波长 (nm.)	Abs.
1	229.20	0.4551
2	197.80	0.3369

The ^{1}H NMR spectrum of compound **4** in acetone-d_6

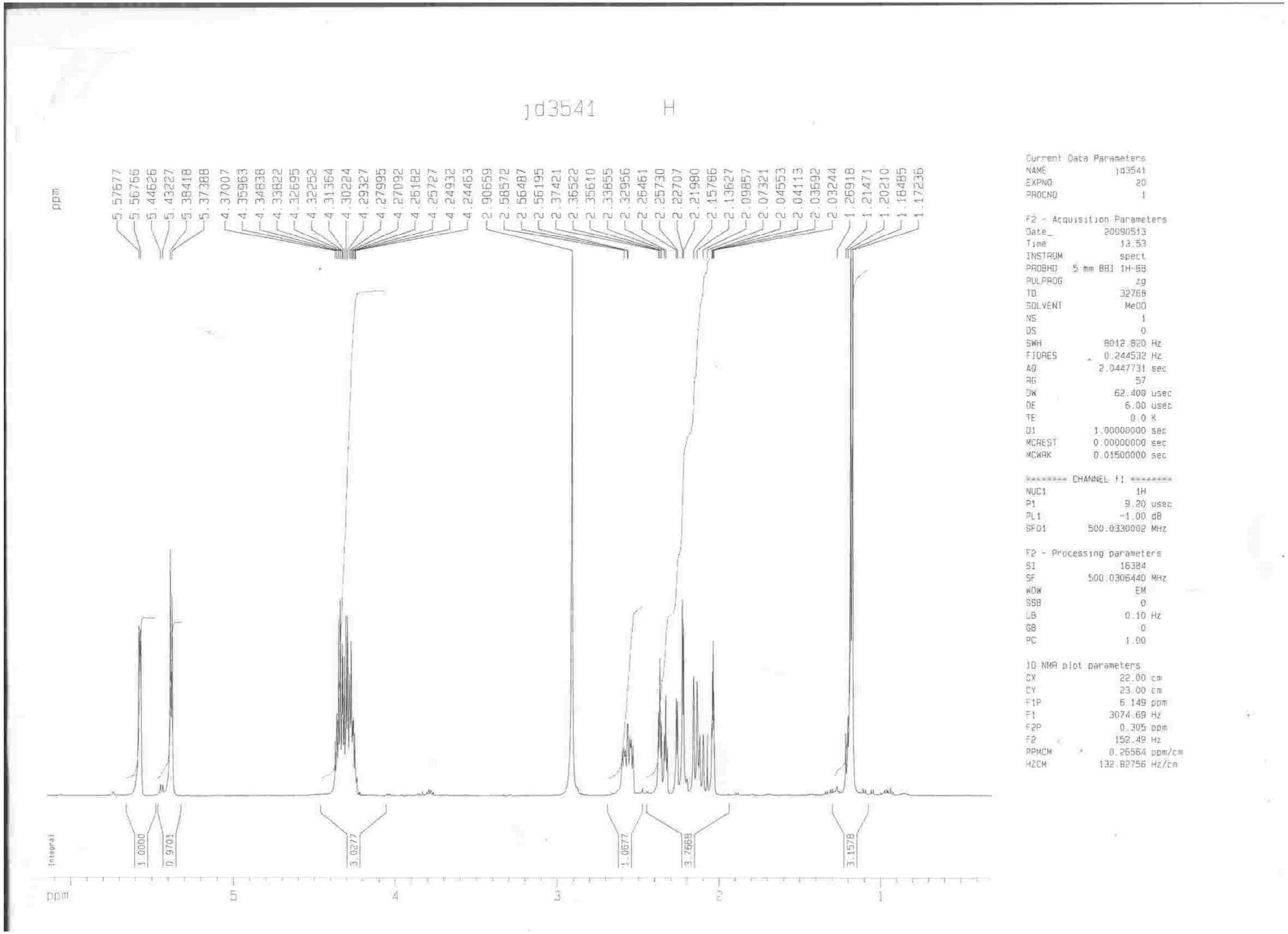

The ^{13}C NMR spectrum of compound **4** in acetone-d_6

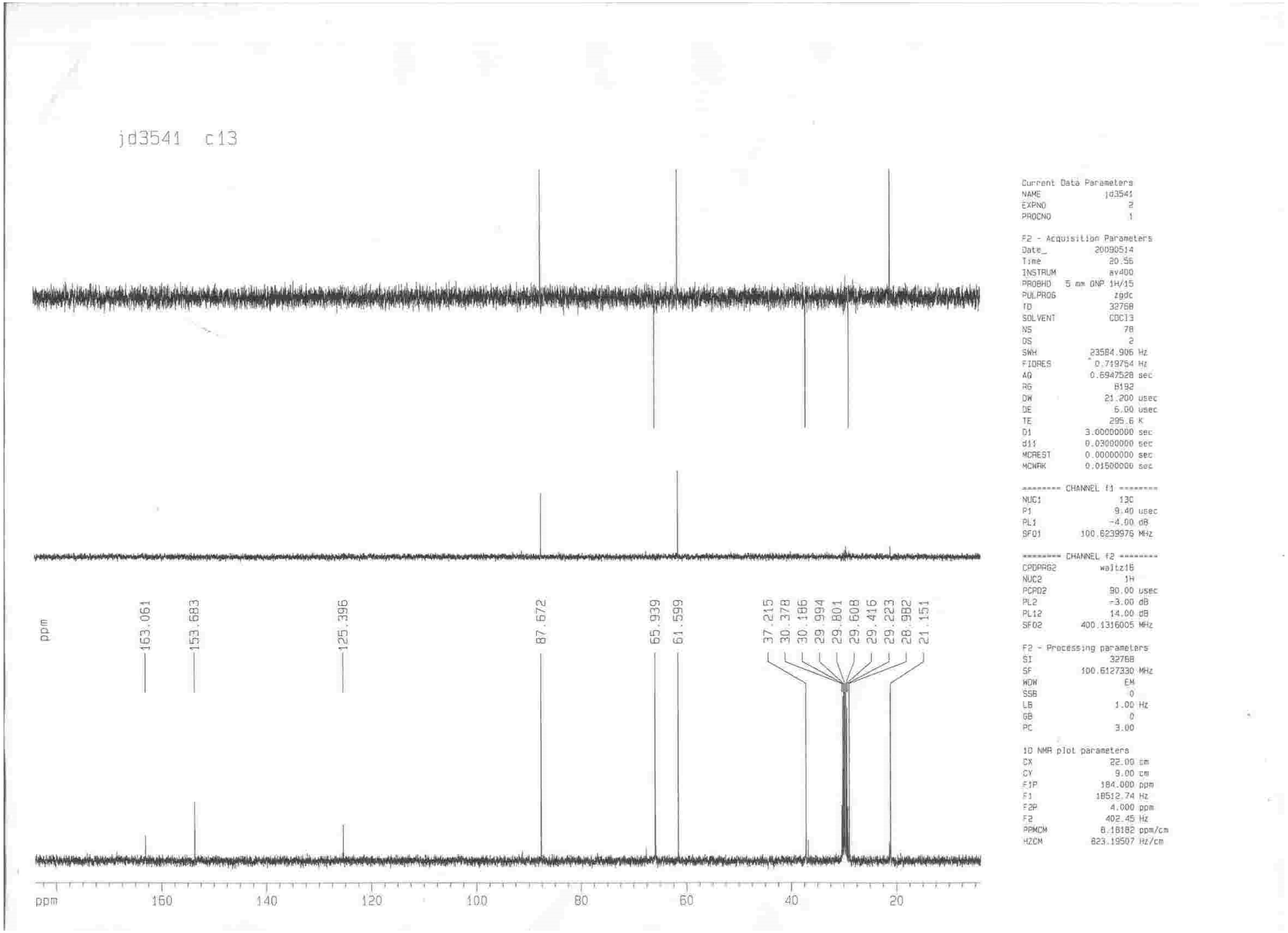

The HSQC spectrum of compound **4** in acetone-d_6

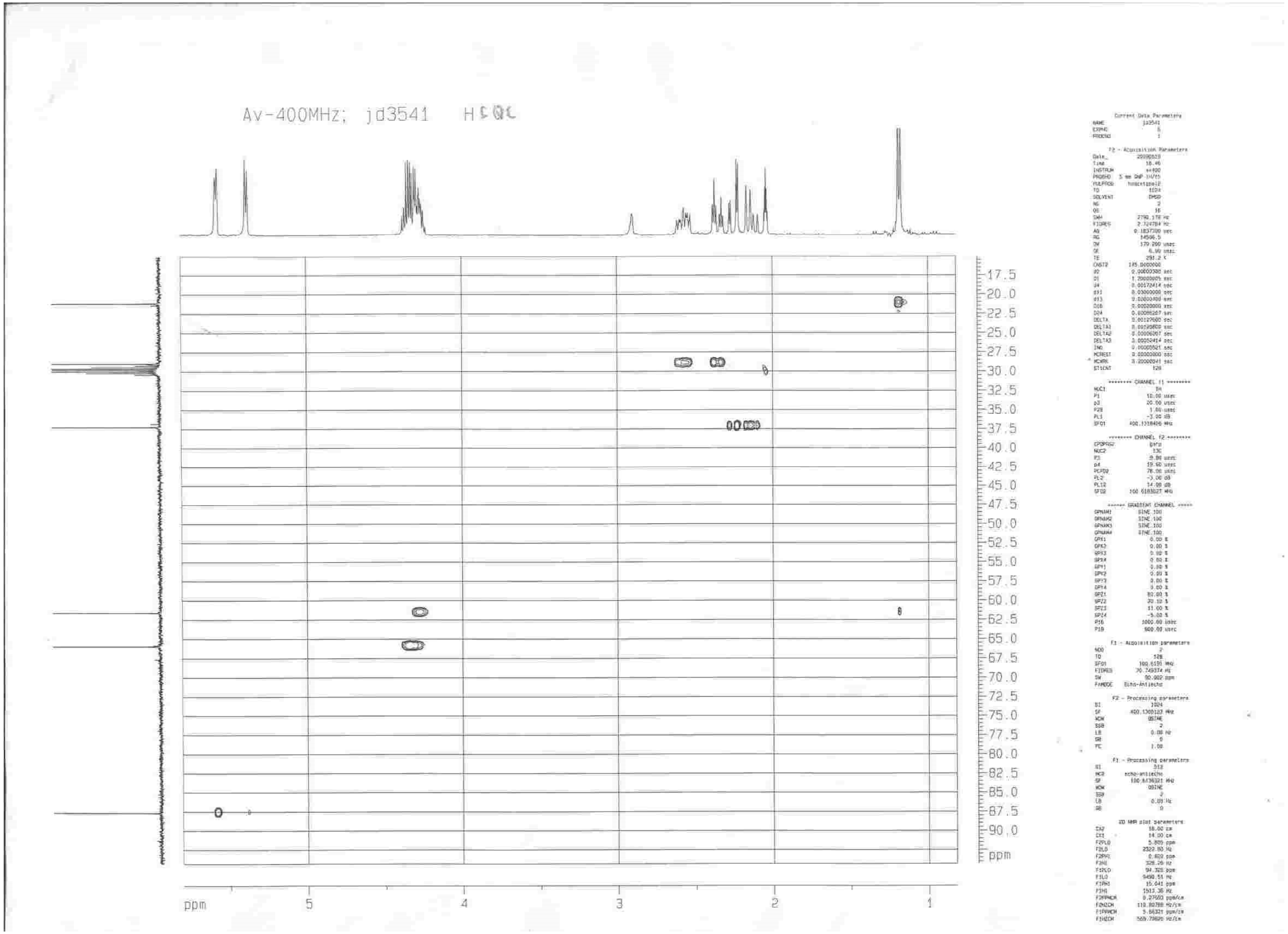

The HMBC spectrum of compound **4** in acetone-d_6

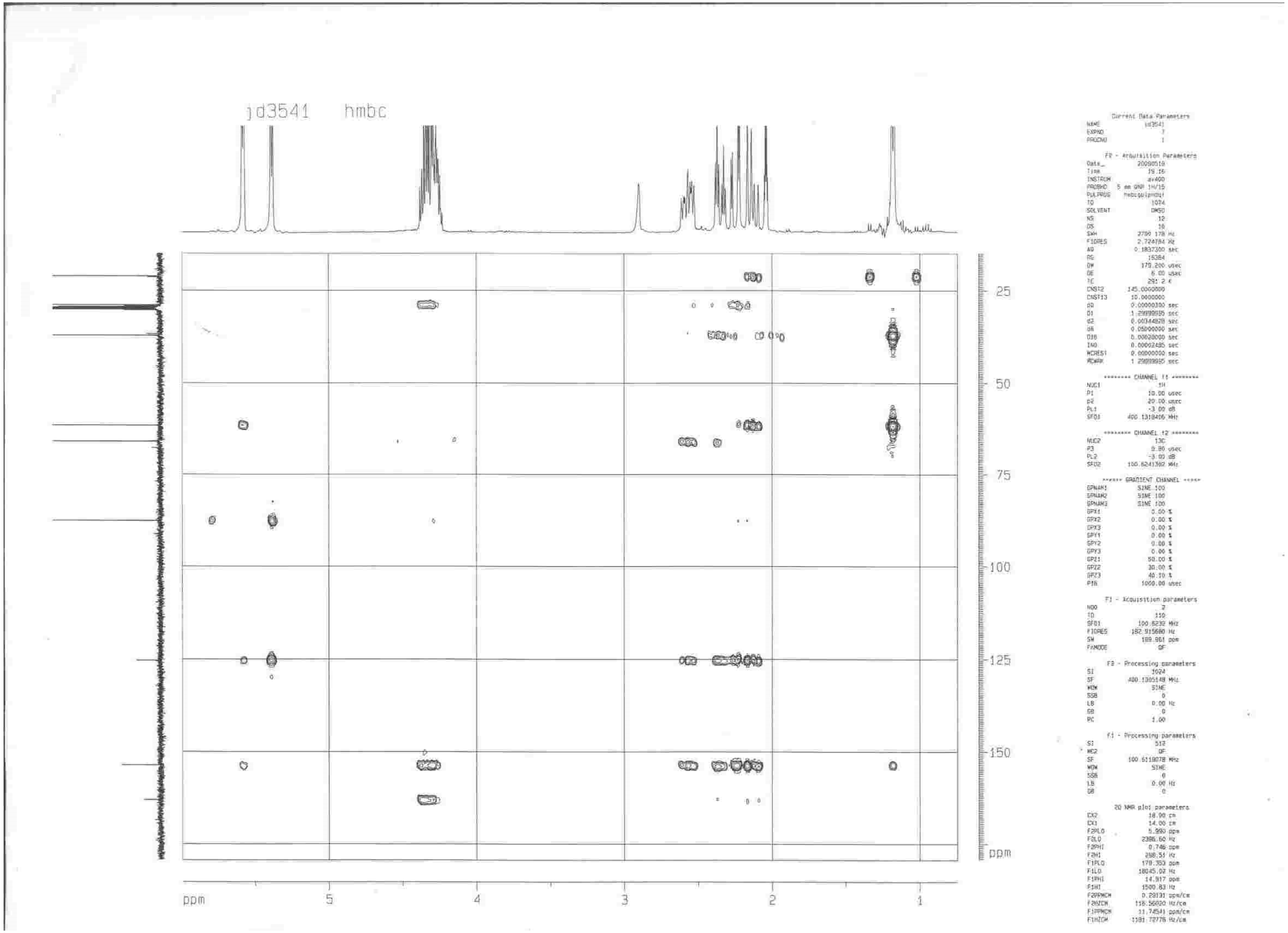

The ROESY spectrum of compound **4** in acetone-d_6

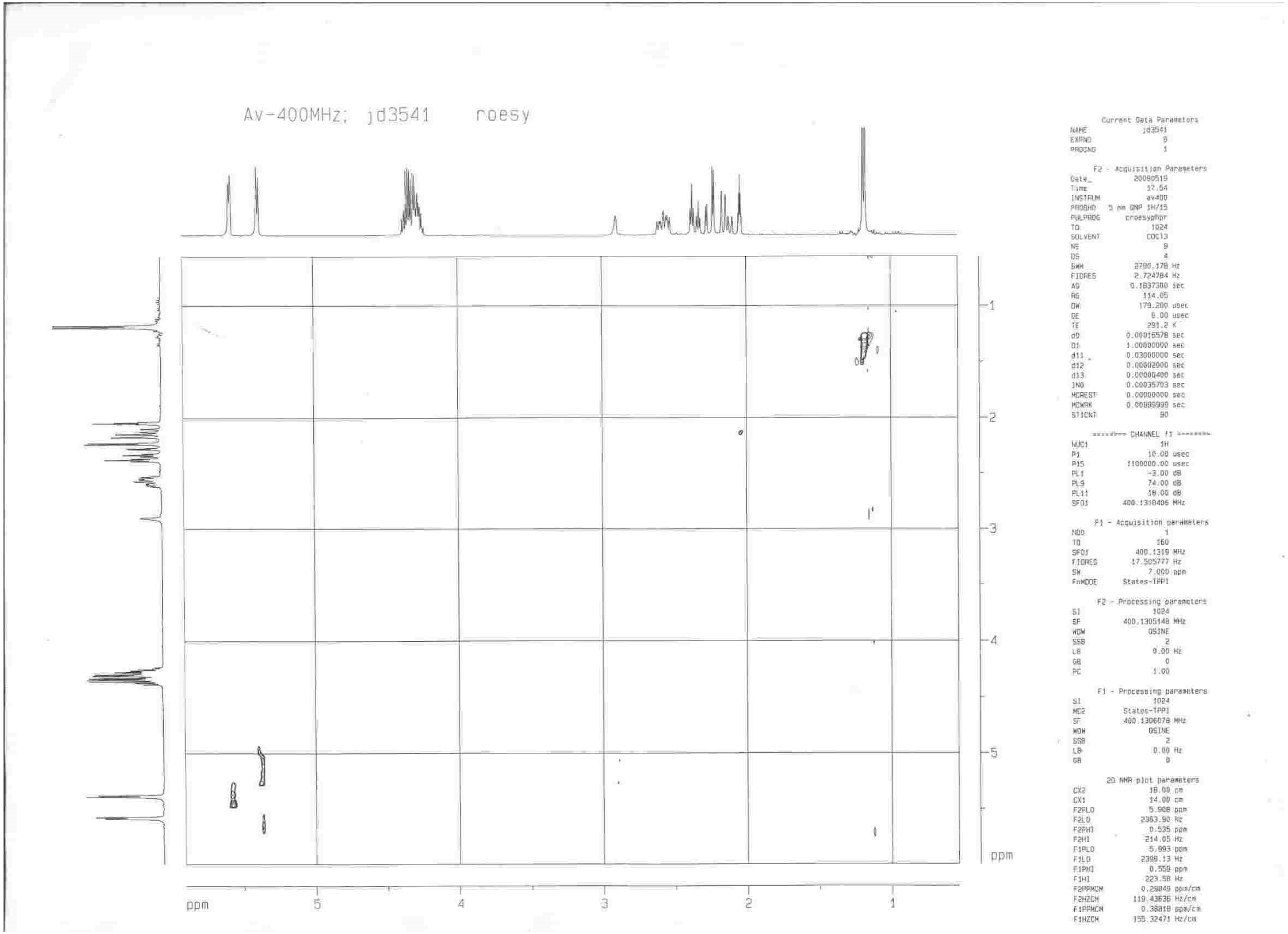

The EIMS spectrum of compound **4**

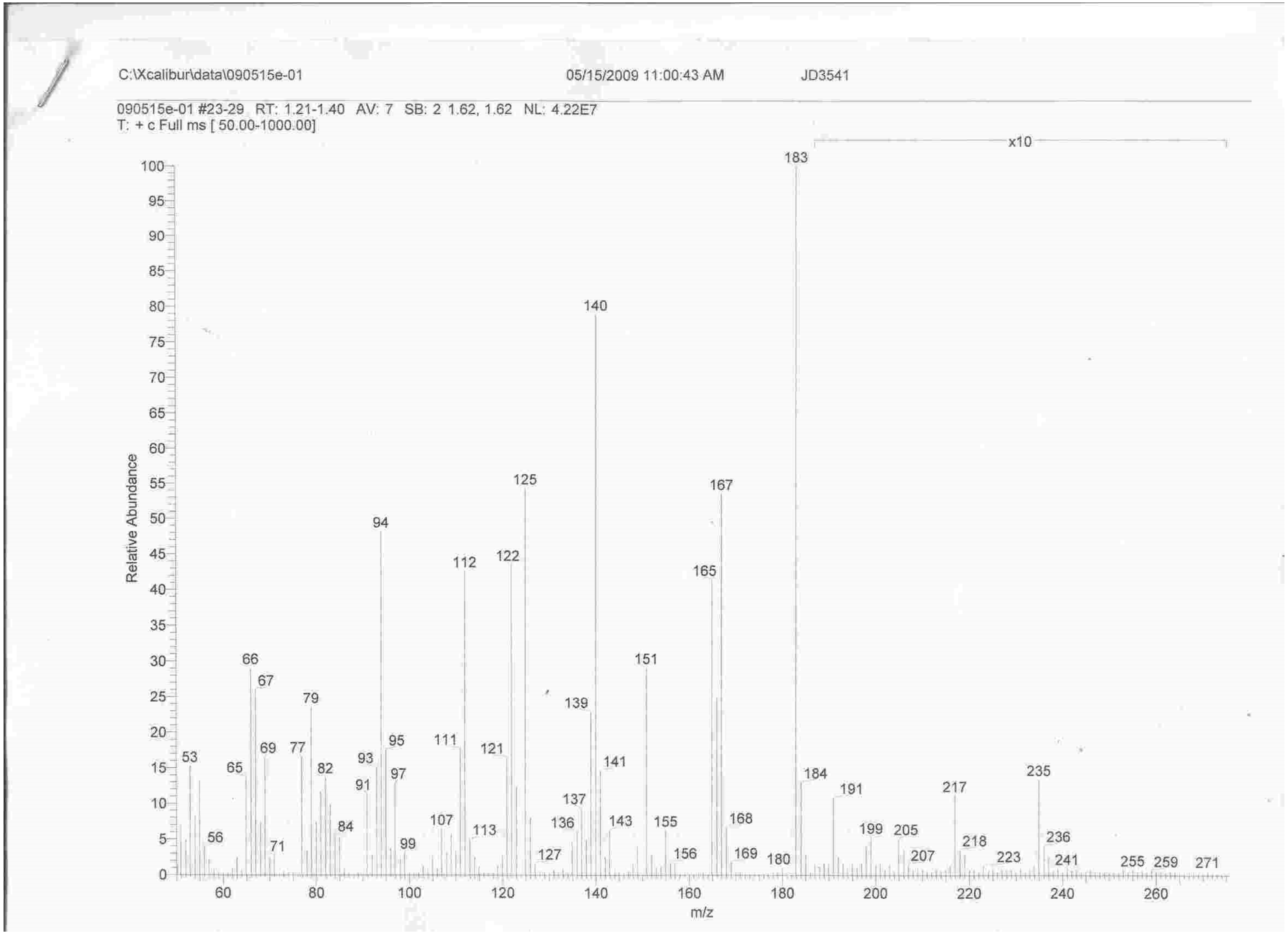

The HRESIMS spectrum of compound **4**

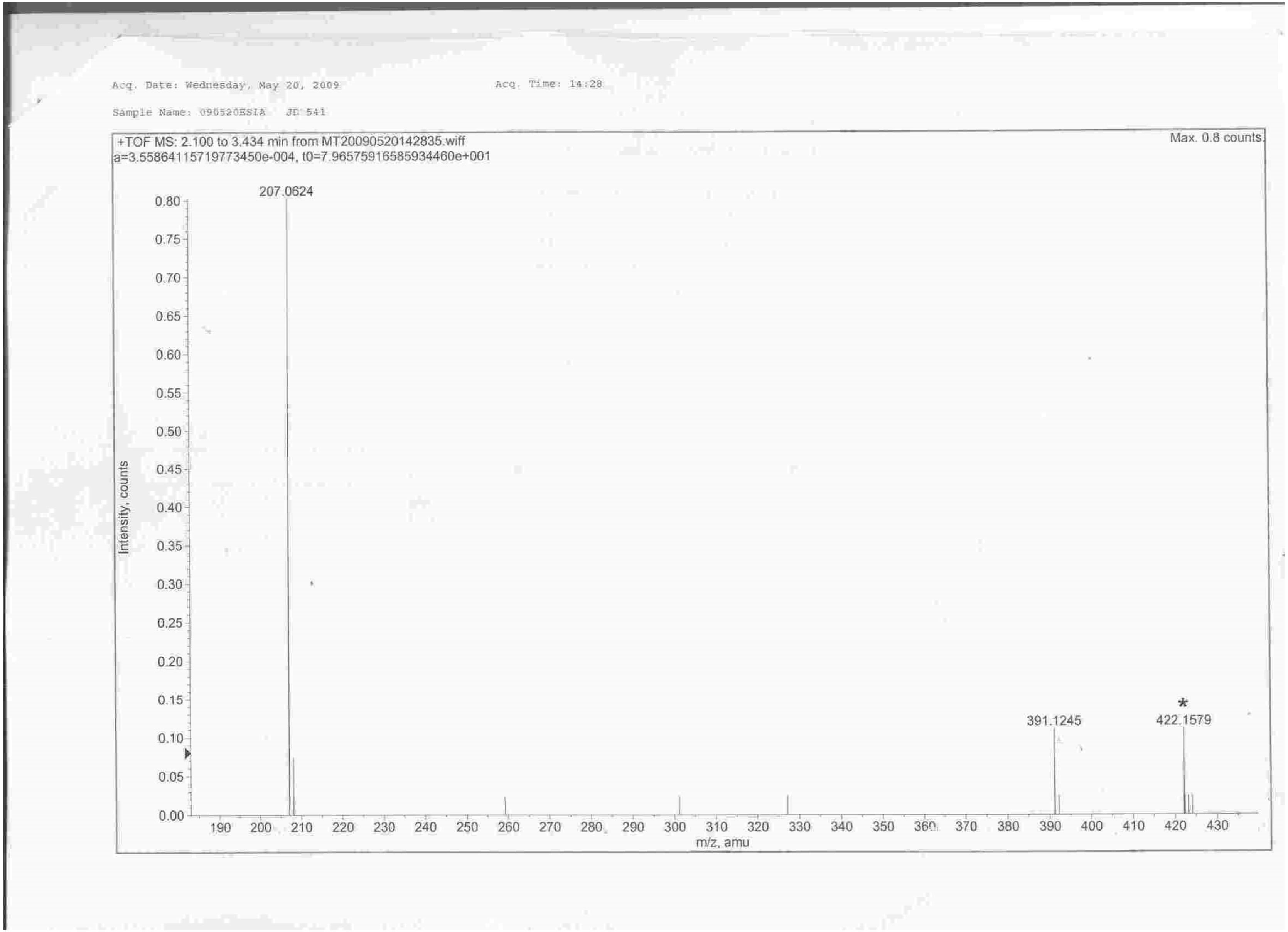

The IR spectrum of compound **4**

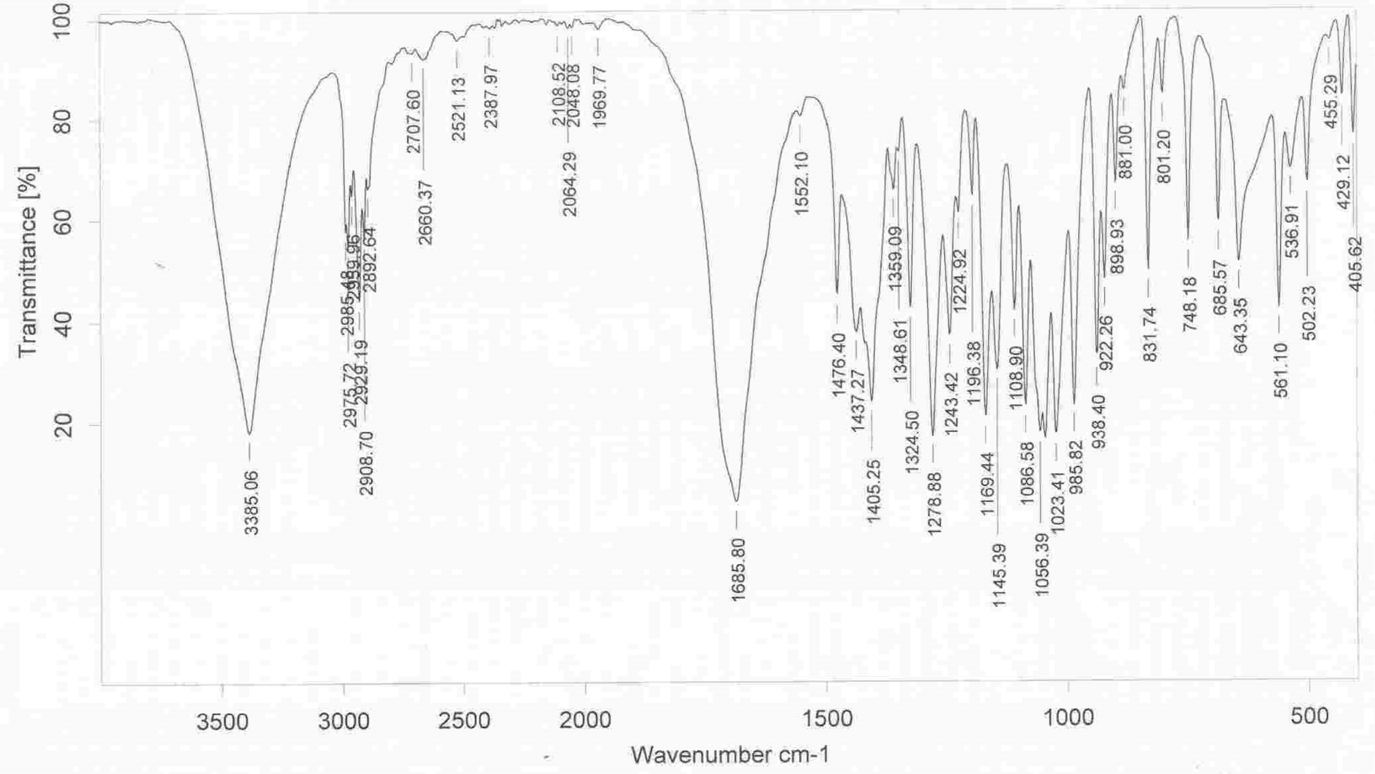

Sample : JD3541	Frequency Range : 399.271 - 3996.57		Measured on : 03/06/2009
Technique : KBr压片	Resolution : 4	Instrument : Tensor27	Sample Scans : 16
Customer : 090603IR3	Zerofilling : 2	Acquisition : Double Sided,For	

The UV spectrum of compound **4**

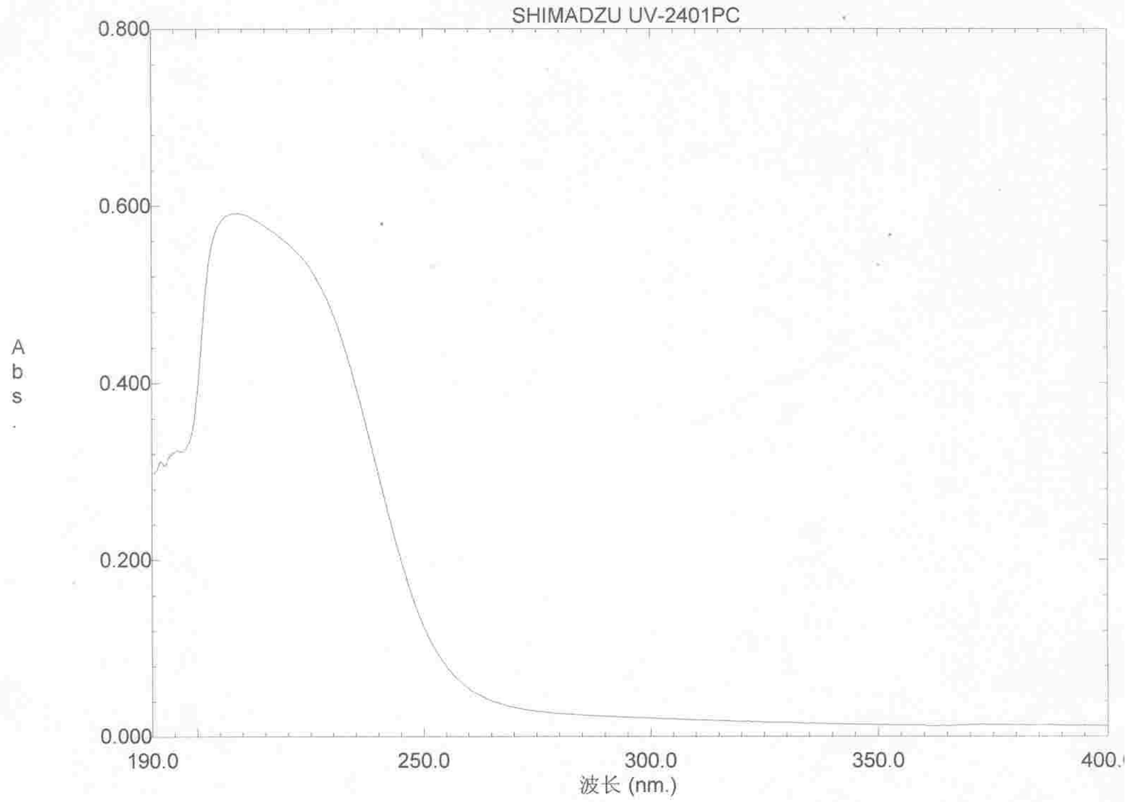

文件名: JD3541

JD3541 ———

样品浓度：0.0140毫克/毫升
溶剂：甲醇

创建于: 16:05 09-06-02
数据: 原始

测量模式: Abs.
扫描速度: 中速
狭缝: 2.0
采样间隔: 0.2

否.	波长 (nm.)	Abs.
1	209.20	0.5915
2	191.80	0.3117

The $[\alpha]_D$ spectrum of compound **4**

Optical rotation measurement

Model : P-1020 (A060460638)

No.	Sample	Mode	Data	Monitor<br>Blank	Temp.<br>Cell<br>Temp Point	Date<br>Comment<br>Sample Name	Light<br>Filter<br>Operator	Cycle Time<br>Integ Time
No.1	1 (1/3)	Sp.Rot	-3.5390	-0.0043<br>0.0000	24.9<br>50.00<br>Cell	Wed Jun 03 13:35:08 2009<br>0.00243g/mlMeOH<br>JD3541	Na<br>589nm	2 sec<br>10 sec
No.2	1 (2/3)	Sp.Rot	-2.1400	-0.0026<br>0.0000	24.9<br>50.00<br>Cell	Wed Jun 03 13:35:22 2009<br>0.00243g/mlMeOH<br>JD3541	Na<br>589nm	2 sec<br>10 sec
No.3	1 (3/3)	Sp.Rot	-0.4940	-0.0006<br>0.0000	24.9<br>50.00<br>Cell	Wed Jun 03 13:35:35 2009<br>0.00243g/mlMeOH<br>JD3541	Na<br>589nm	2 sec<br>10 sec

−2.0576°

The ^{1}H NMR spectrum of compound **5** in $CDCl_3$

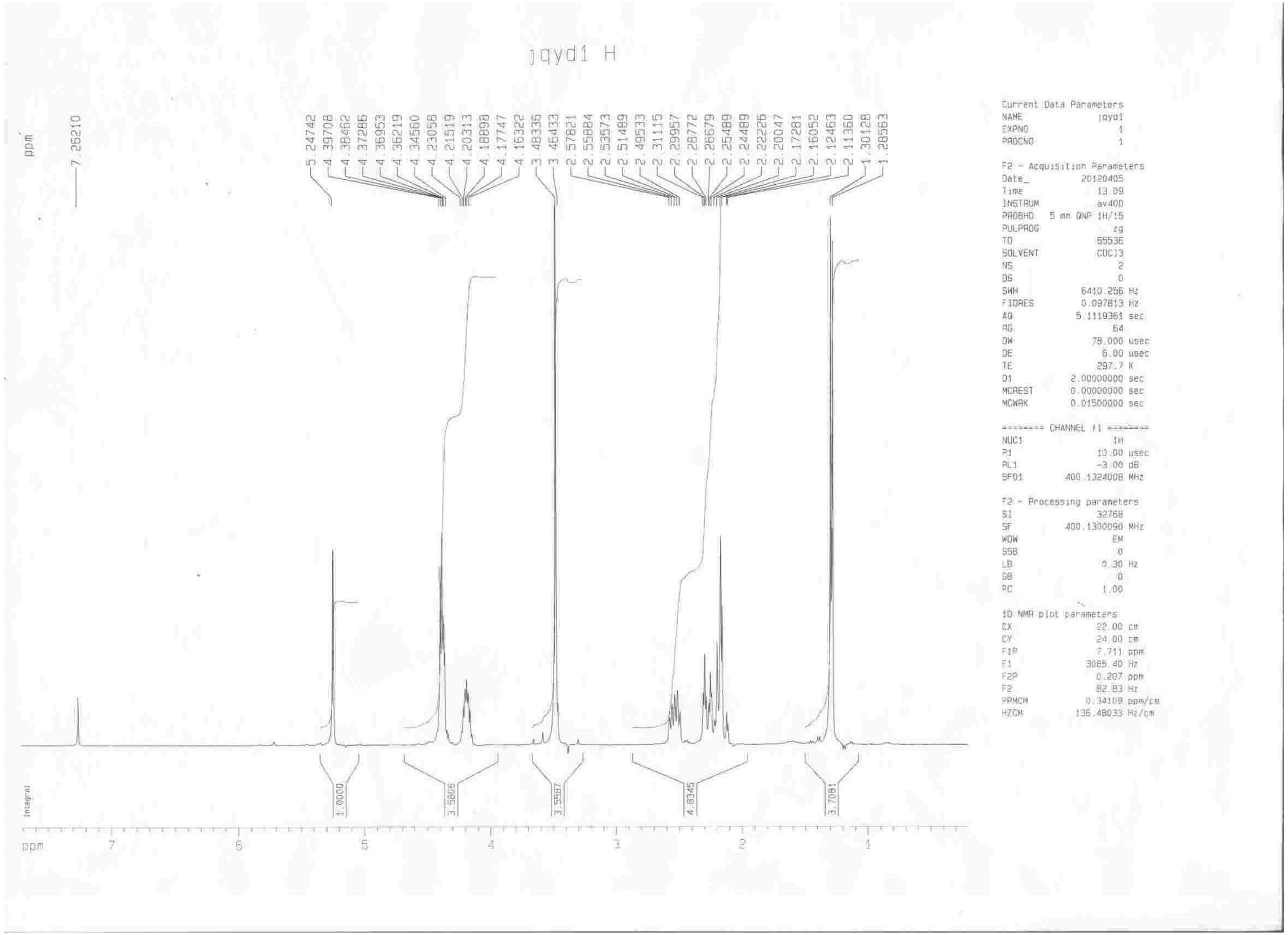

The ^{13}C NMR spectrum of compound **5** in $CDCl_3$

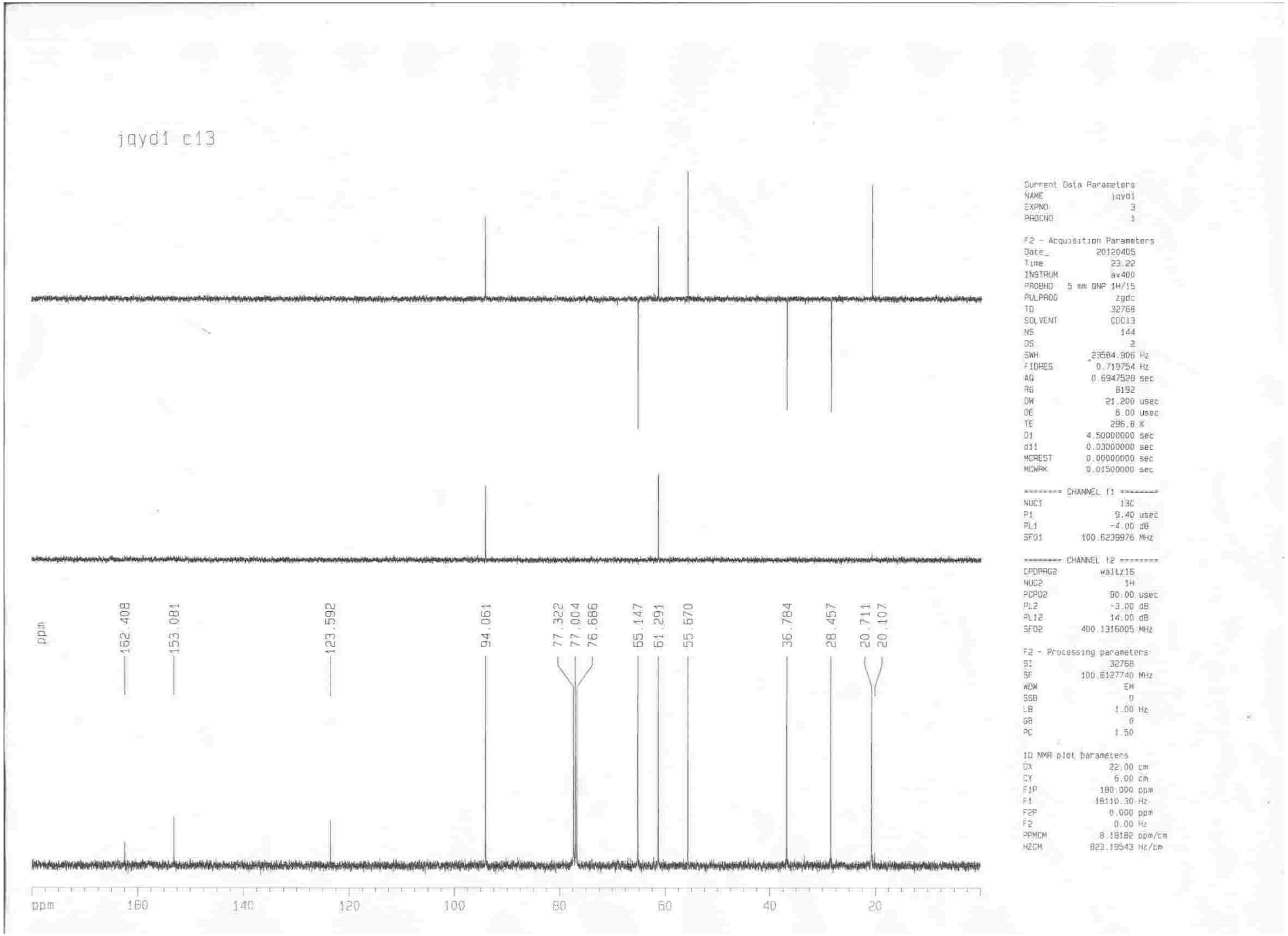

The HSQC spectrum of compound **5** in $CDCl_3$

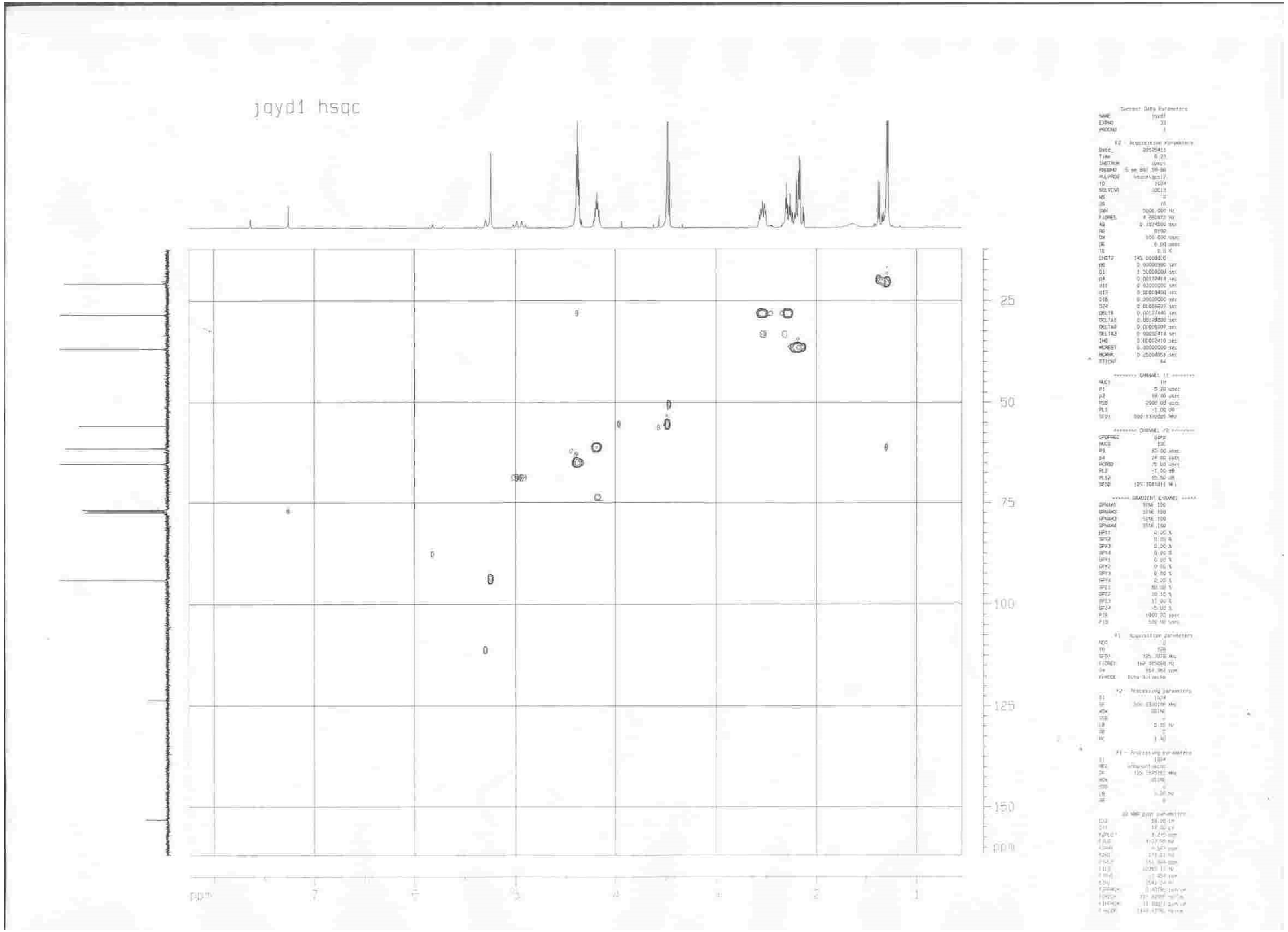

The HMBC spectrum of compound **5** in $CDCl_3$

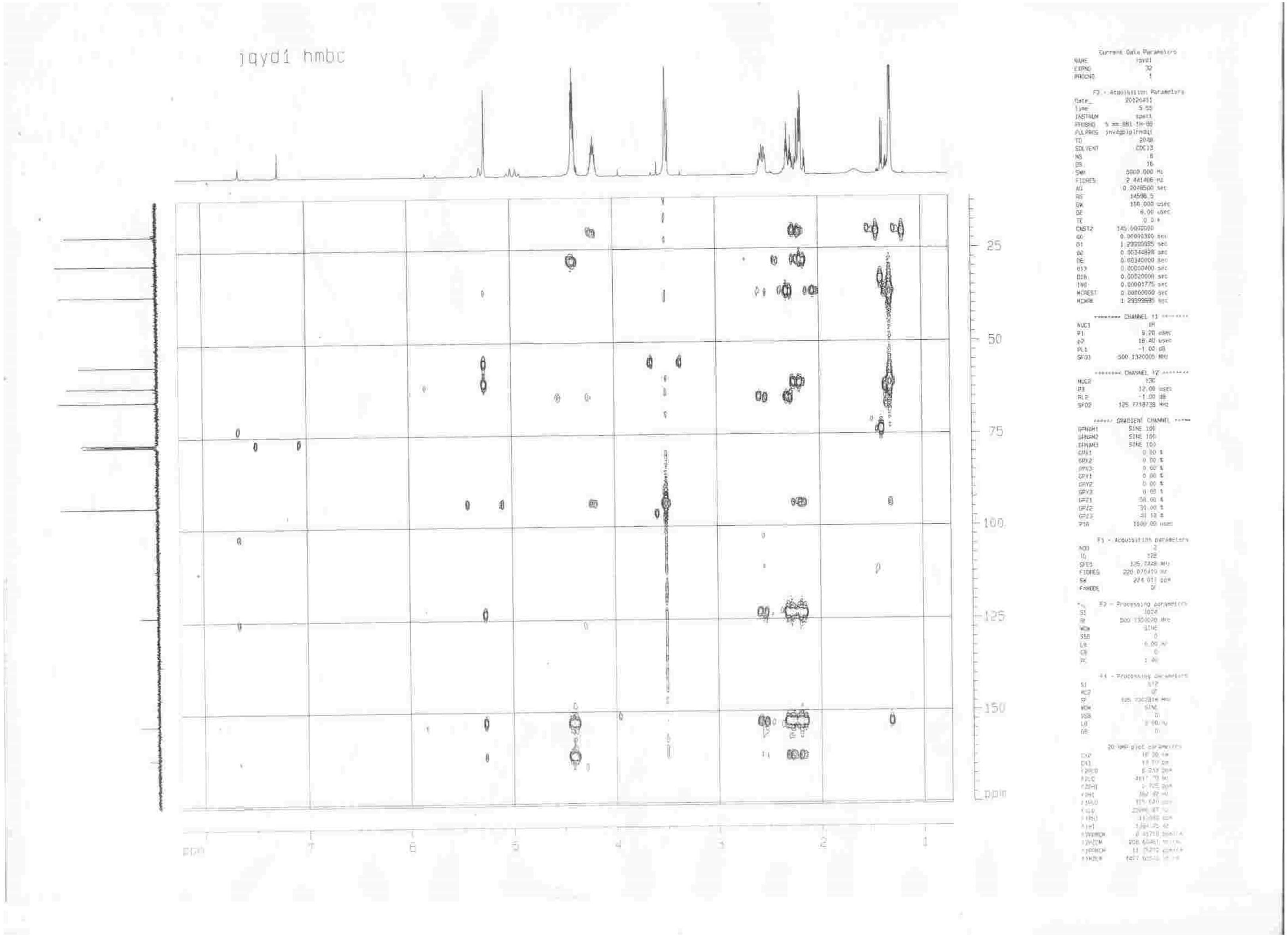

The ^{1}H ^{1}H COSY spectrum of compound **5** in $CDCl_3$

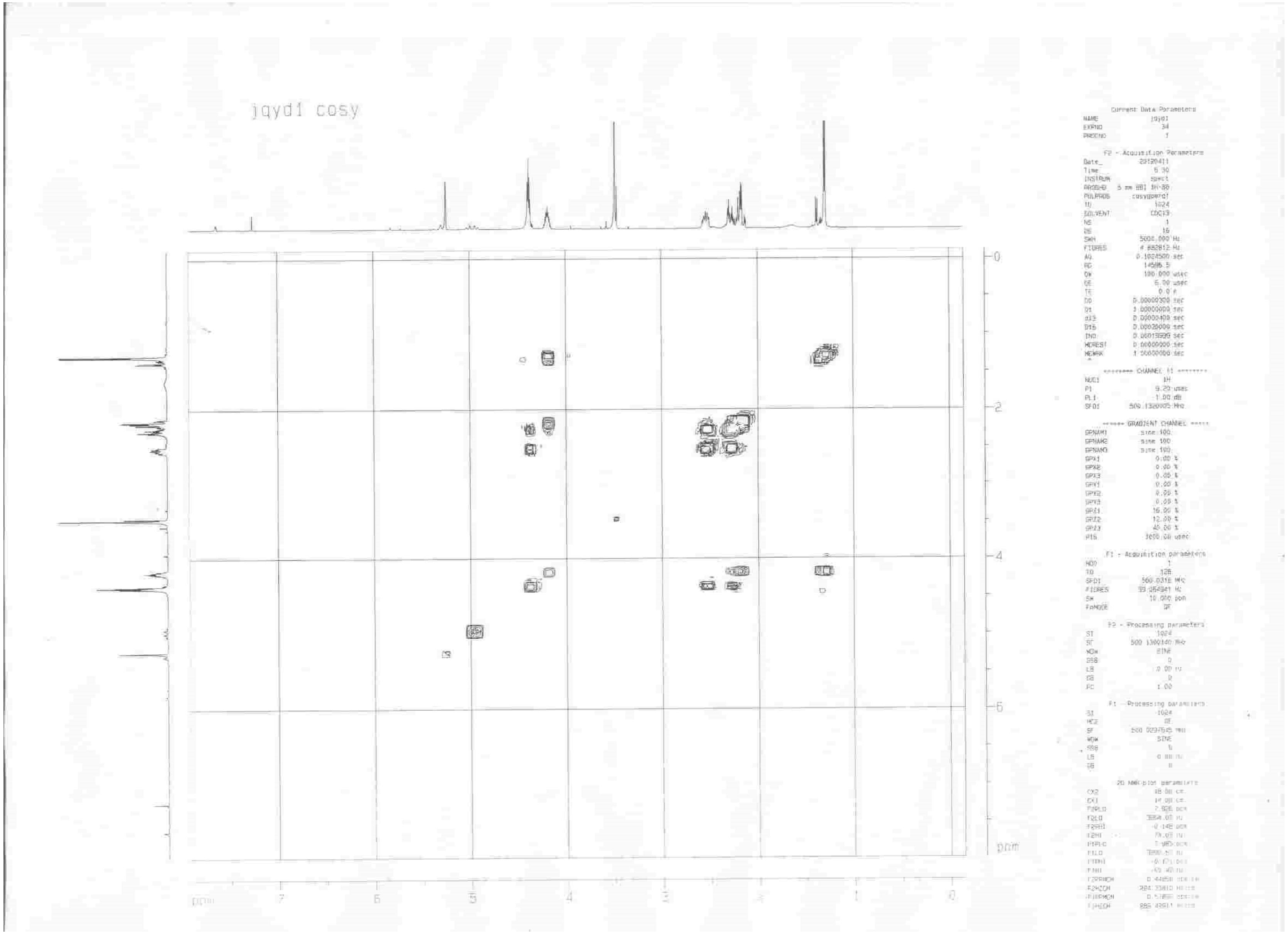

The ROESY spectrum of compound **5** in $CDCl_3$

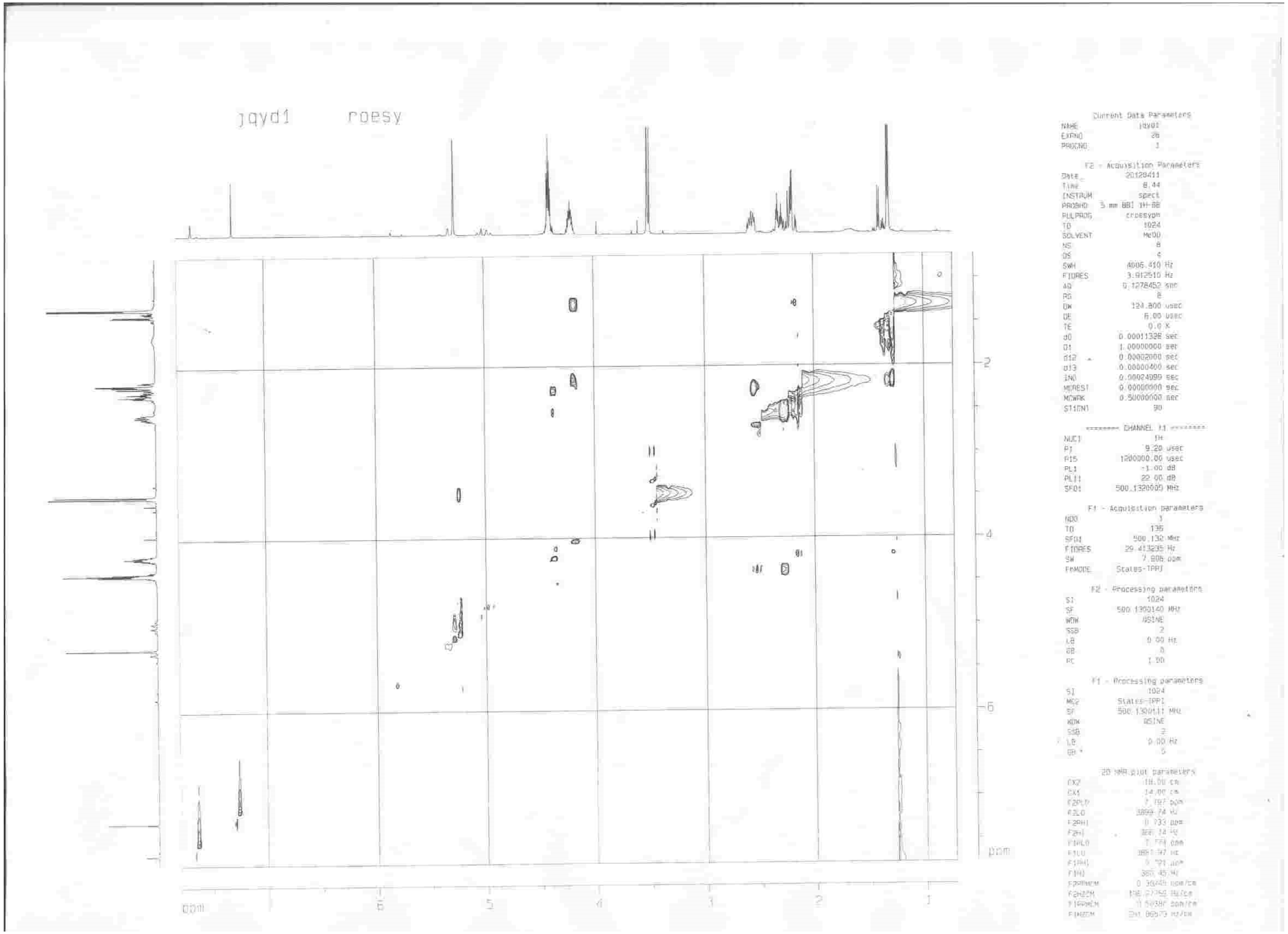

The HRESIMS spectrum of compound **5**

Formula Predictor Report - gca11_QYD-1_2.lcd

Page 1 of 1

Data File: D:\Gavin\Data\gca11_QYD-1_2.lcd

Elmt	Val.	Min	Max	Elmt	Val.	Min	Max	Use Adduct
H	1	0	50	O	2	0	11	H
C	4	0	34					Na
N	3	0	1					

Error Margin (mDa): 10.0
HC Ratio: unlimited
Max Isotopes: all
MSn Iso RI (%): 75.00

DBE Range: 0.0 - 30.0
Apply N Rule: yes
Isotope RI (%): 1.00
MSn Logic Mode: OR

Electron Ions: both
Use MSn Info: yes
Isotope Res: 10000
Max Results: 500

Event#: 1 MS(E+) Ret. Time : 0.147 -> 0.320 - 1.720 -> 2.533 Scan# : 23 -> 49 - 259 -> 381

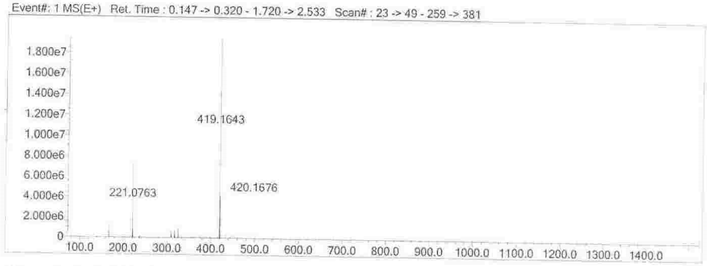

Measured region for 221.0763 m/z

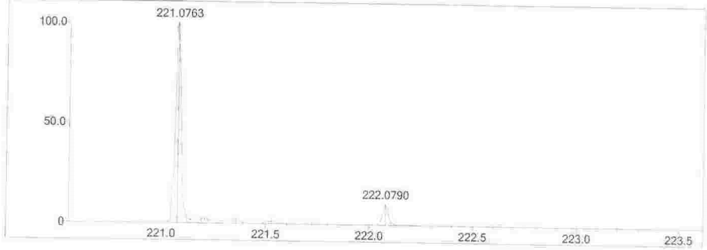

C10 H14 O4 [M+Na]+ : Predicted region for 221.0784 m/z

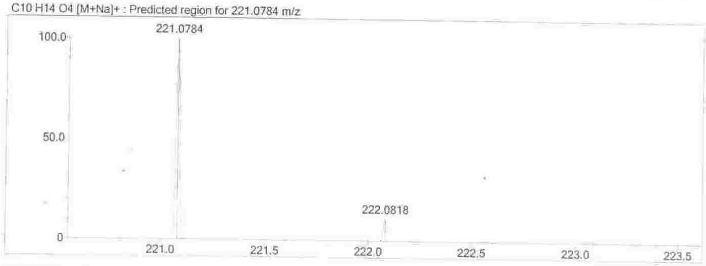

Rank	Score	Ion	Formula (M)	Pred. m/z	Meas. m/z	Df. (mDa)	Df. (ppm)	Iso	DBE
1	21.79	[M+Na]+	C10 H14 O4	221.0784	221.0763	-2.1	-9.50	48.41	4.0

The IR spectrum of compound **5**

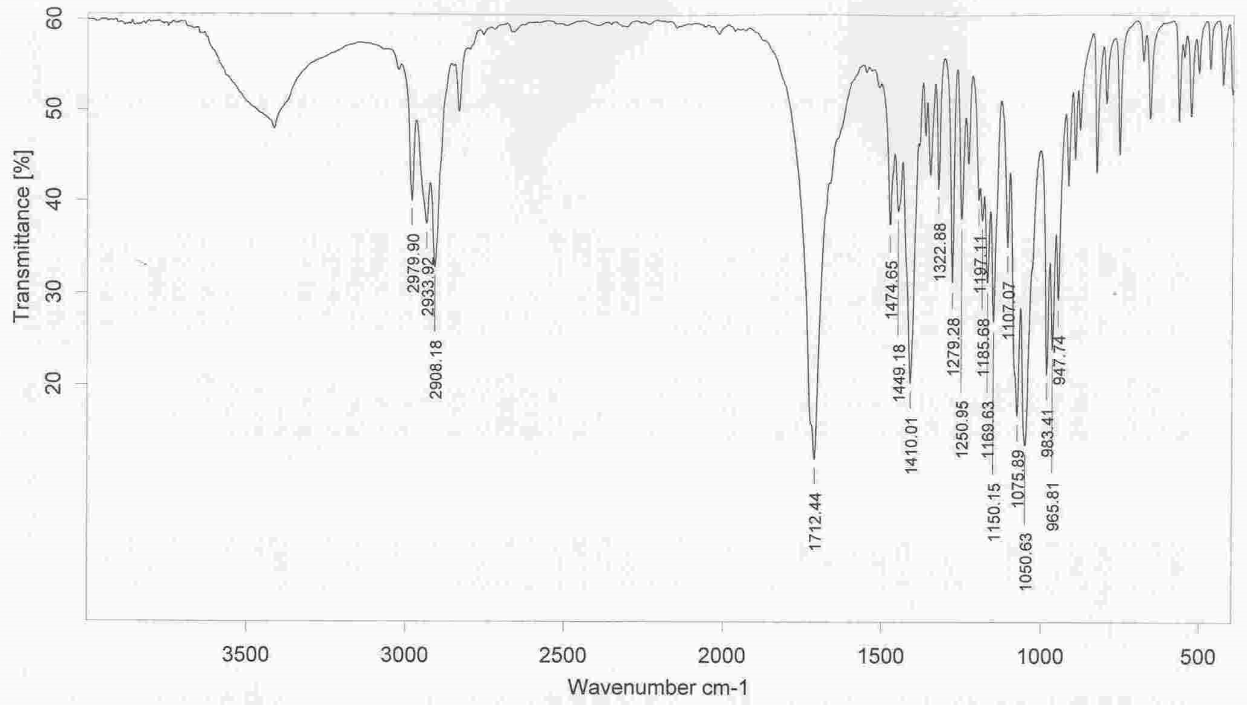

Sample : JQYD-1	Frequency Range : 399.246 - 3996.32	Measured on : 17/04/2012	
Technique : KBr压片	Resolution : 4	Instrument : Tensor27	Sample Scans : 16
Customer : 120417IR2	Zerofilling : 2	Acquisition : Double Sided,For	

The UV spectrum of compound **5**

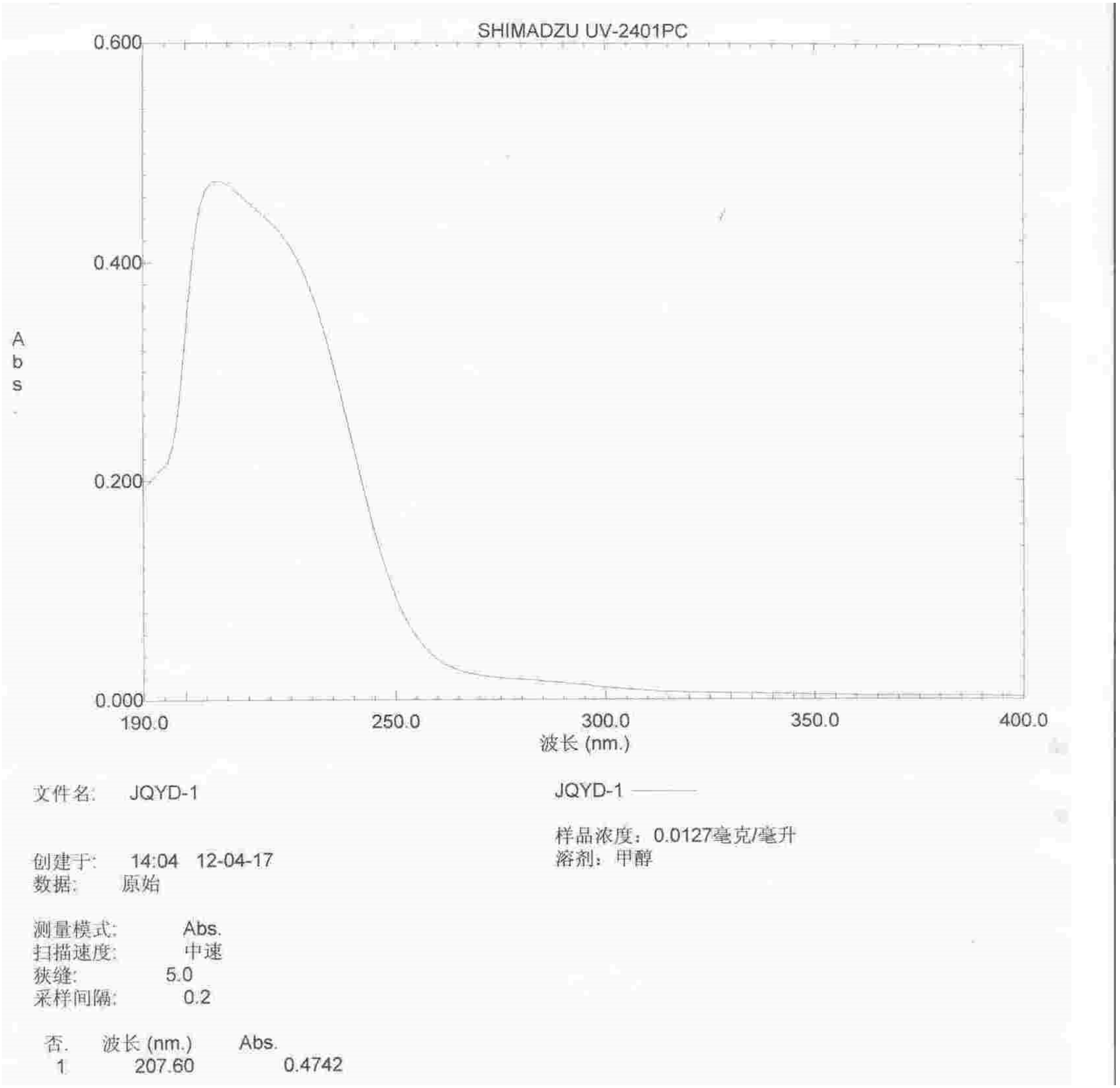

The [α]$_D$ spectrum of compound **5**

Optical rotation measurement

Model : P-1020 (A060460638)

No.	Sample	Mode	Data	Monitor<br>Blank	Temp.<br>Cell<br>Temp Point	Date<br>Comment<br>Sample Name	Light<br>Filter<br>Operator	Cycle Time<br>Integ Time
No.1	2 (1/3)	Sp.Rot	-5.9730	-0.0066<br>0.0000	22.1<br>50.00<br>Cell	Tue Apr 17 10:58:04 2012<br>0.00221g/mlMeOH<br>JQYD-1	Na<br>589nm	2 sec<br>10 sec
No.2	2 (2/3)	Sp.Rot	-6.1540	-0.0068<br>0.0000	22.1<br>50.00<br>Cell	Tue Apr 17 10:58:18 2012<br>0.00221g/mlMeOH<br>JQYD-1	Na<br>589nm	2 sec<br>10 sec
No.3	2 (3/3)	Sp.Rot	-6.0630	-0.0067<br>0.0000	22.1<br>50.00<br>Cell	Tue Apr 17 10:58:31 2012<br>0.00221g/mlMeOH<br>JQYD-1	Na<br>589nm	2 sec<br>10 sec

-6.0633 °

The ^{1}H NMR spectrum of compound **6** in $CDCl_3$

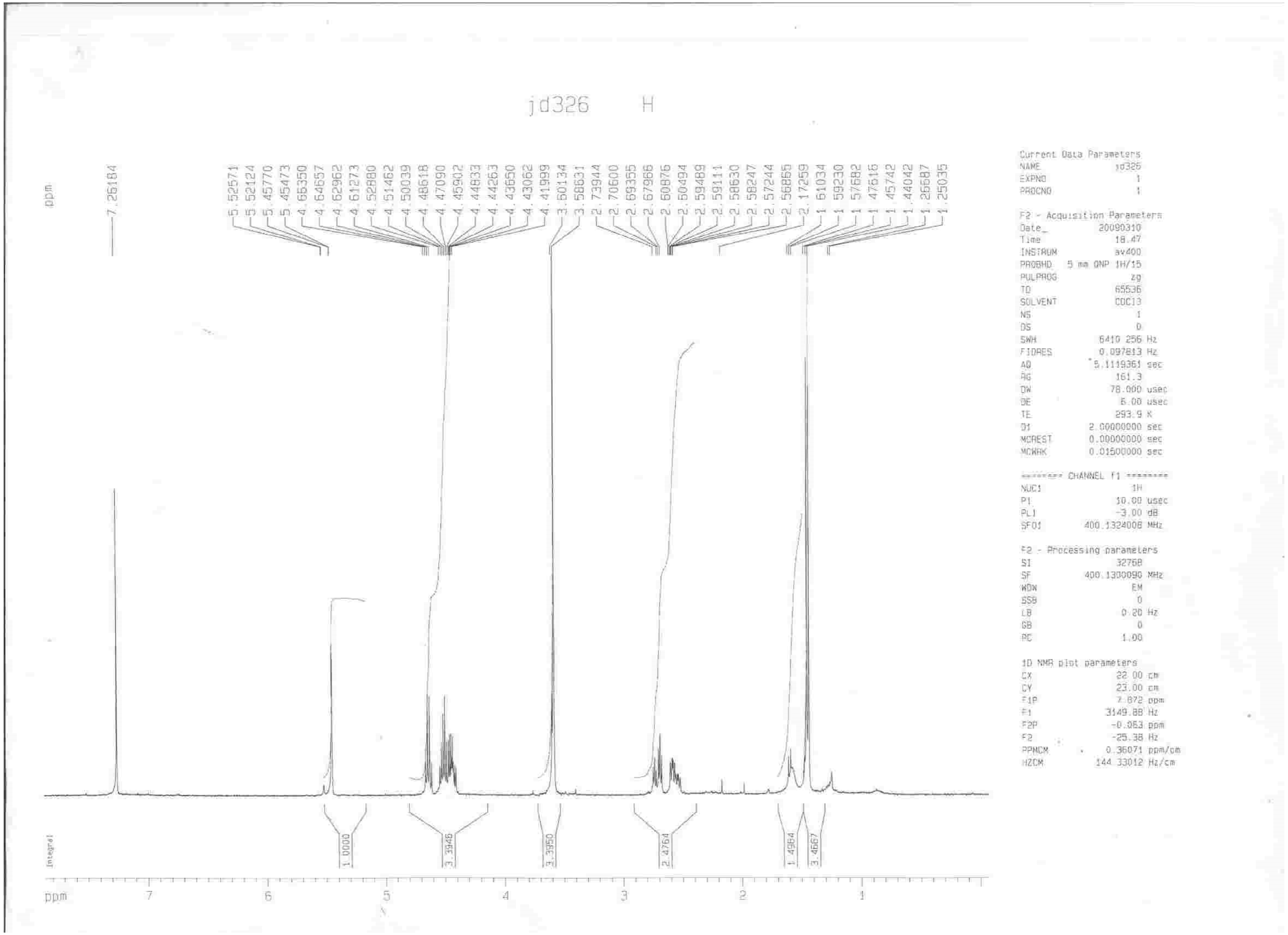

The ^{13}C NMR spectrum of compound **6** in $CDCl_3$

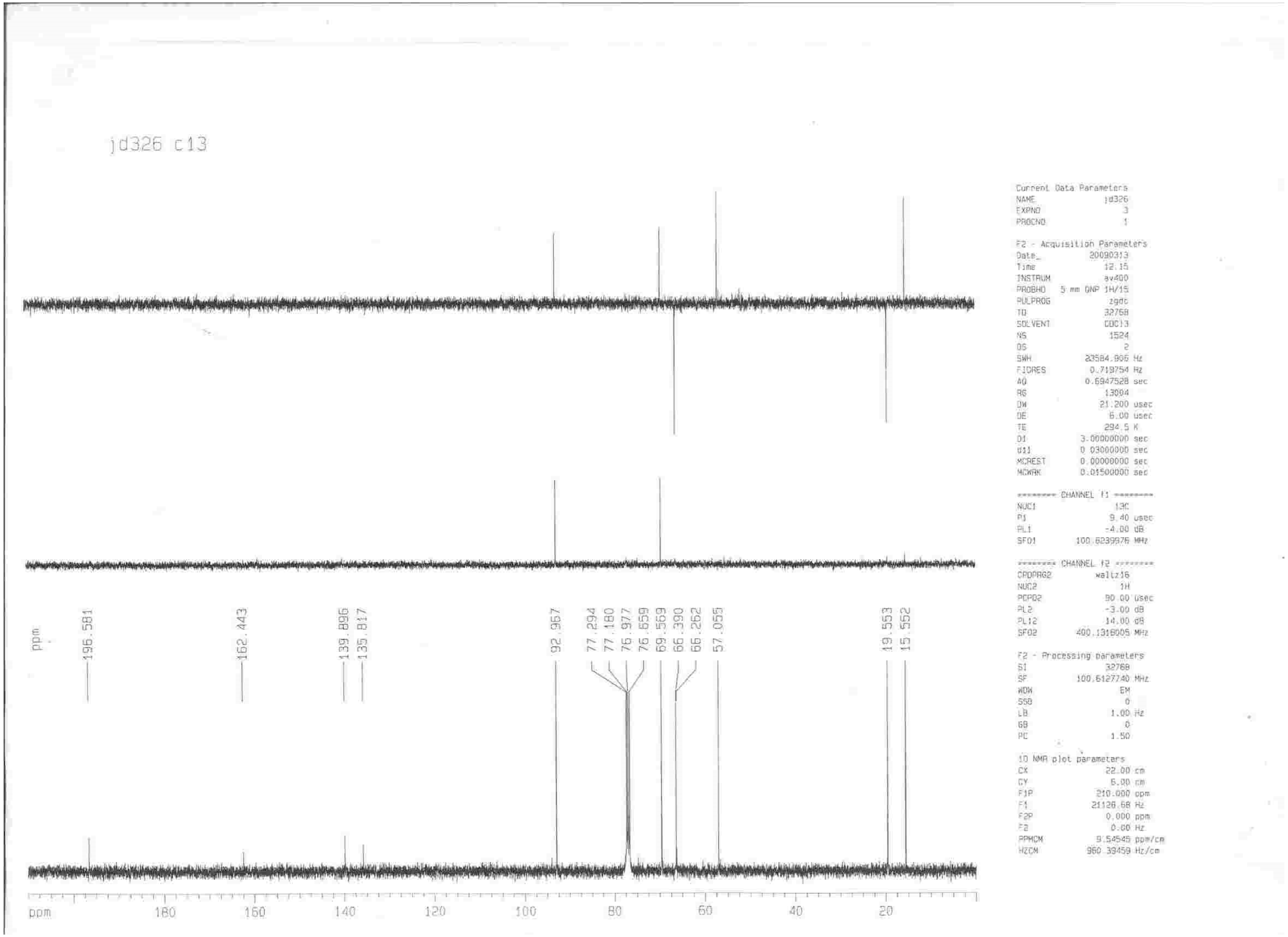

The HSQC spectrum of compound **6** in $CDCl_3$

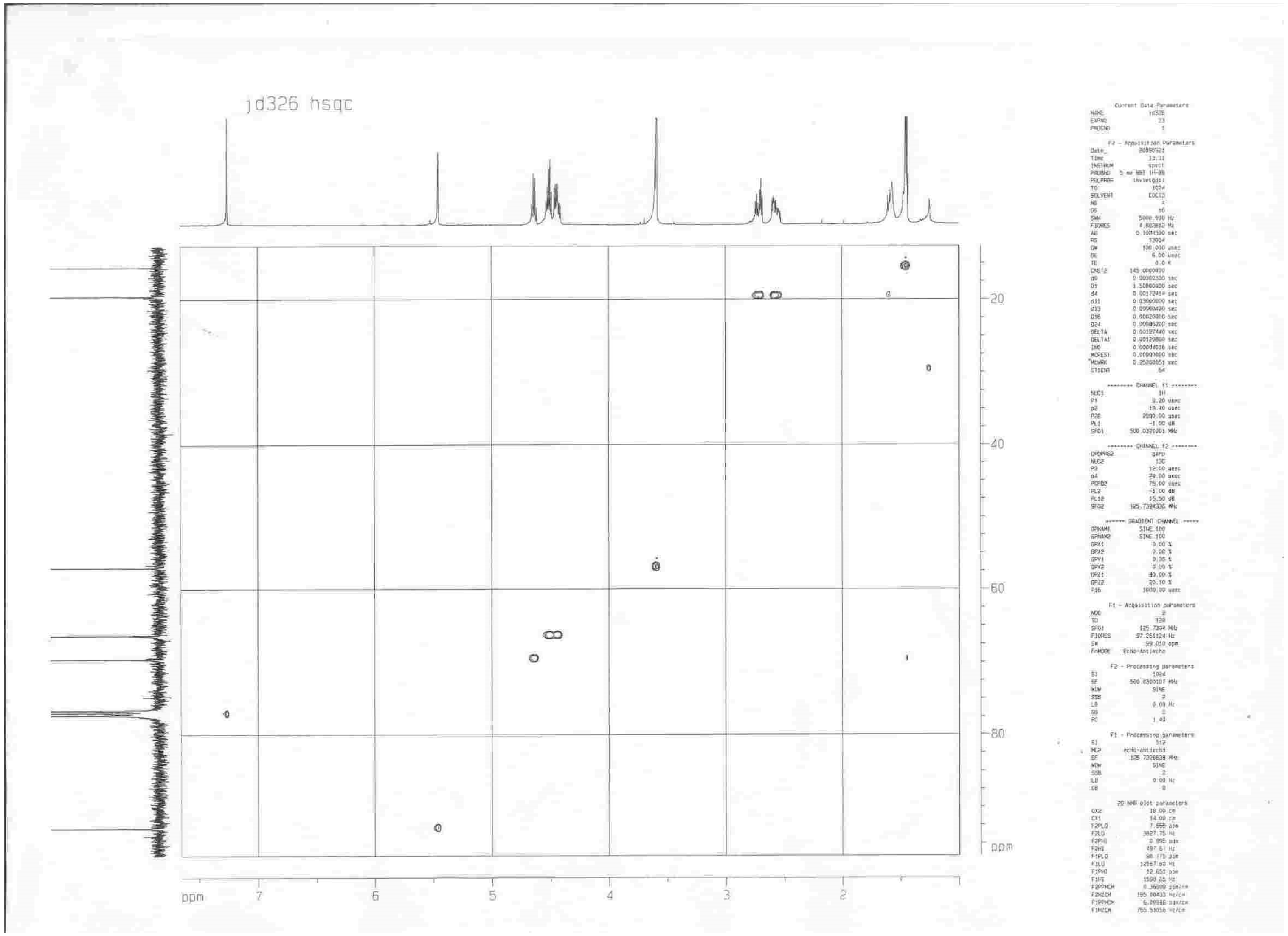

The HMBC spectrum of compound **6** in $CDCl_3$

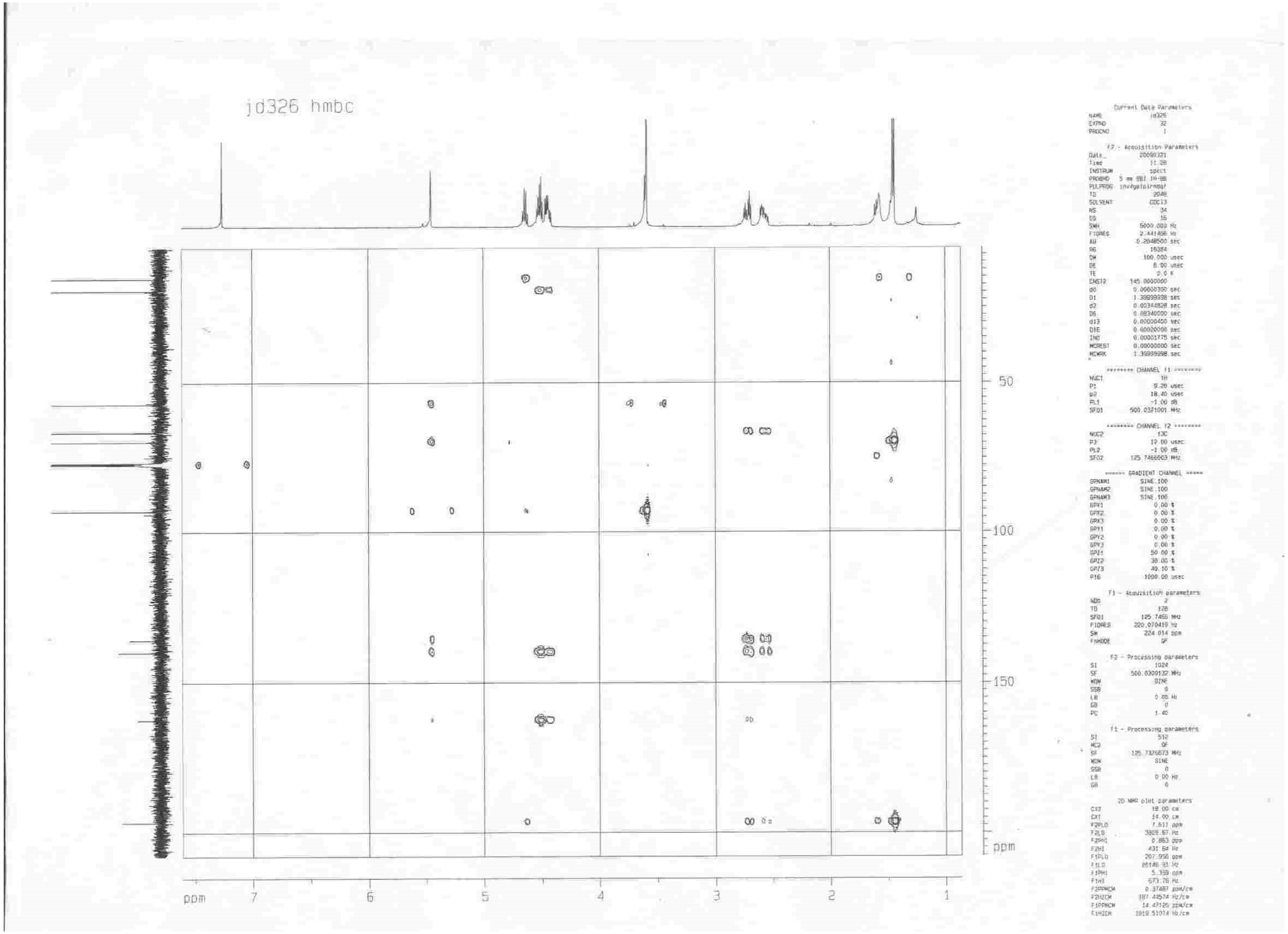

The ROESY spectrum of compound **6** in $CDCl_3$

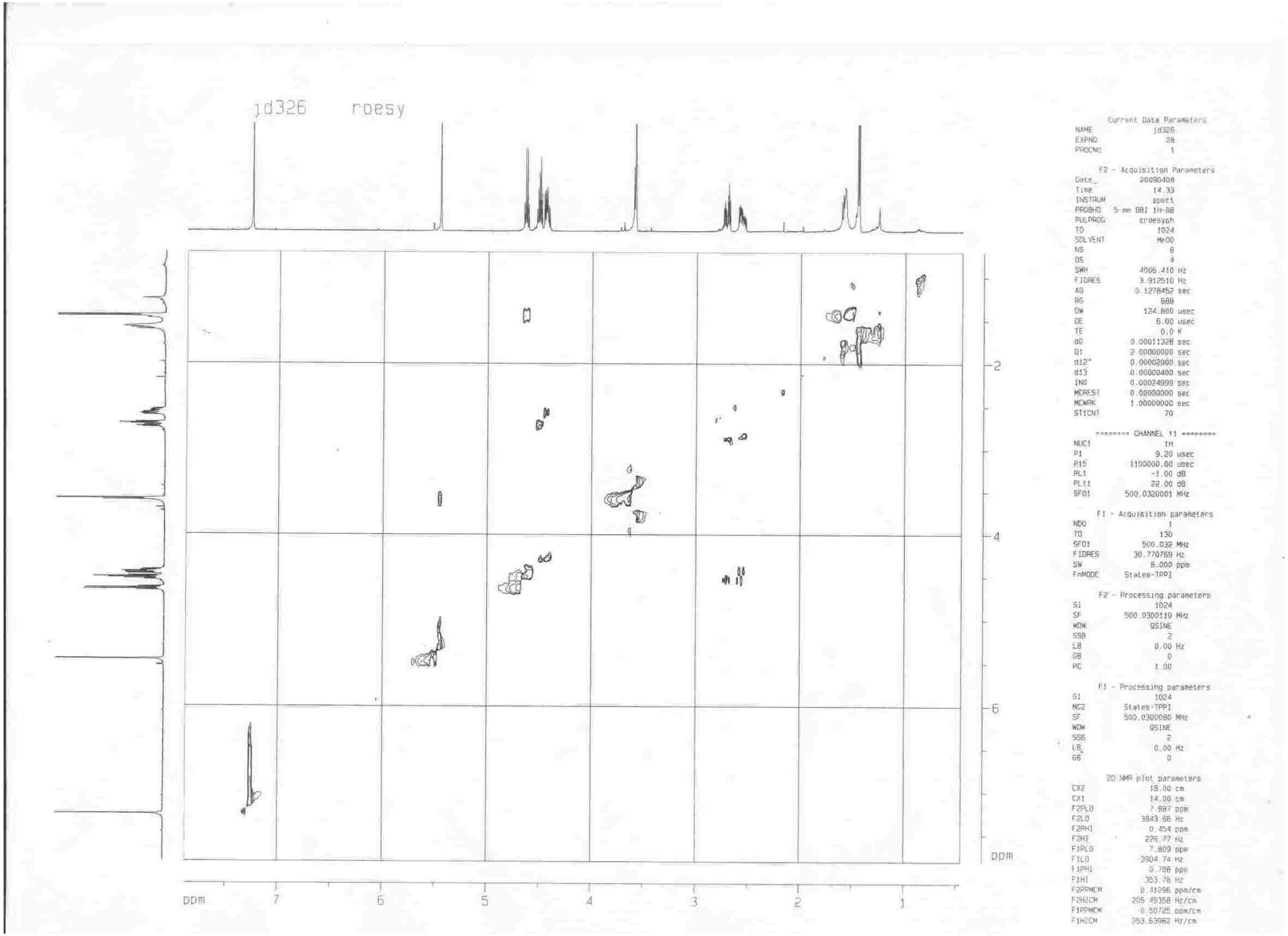

The ESIMS spectrum of compound **6**

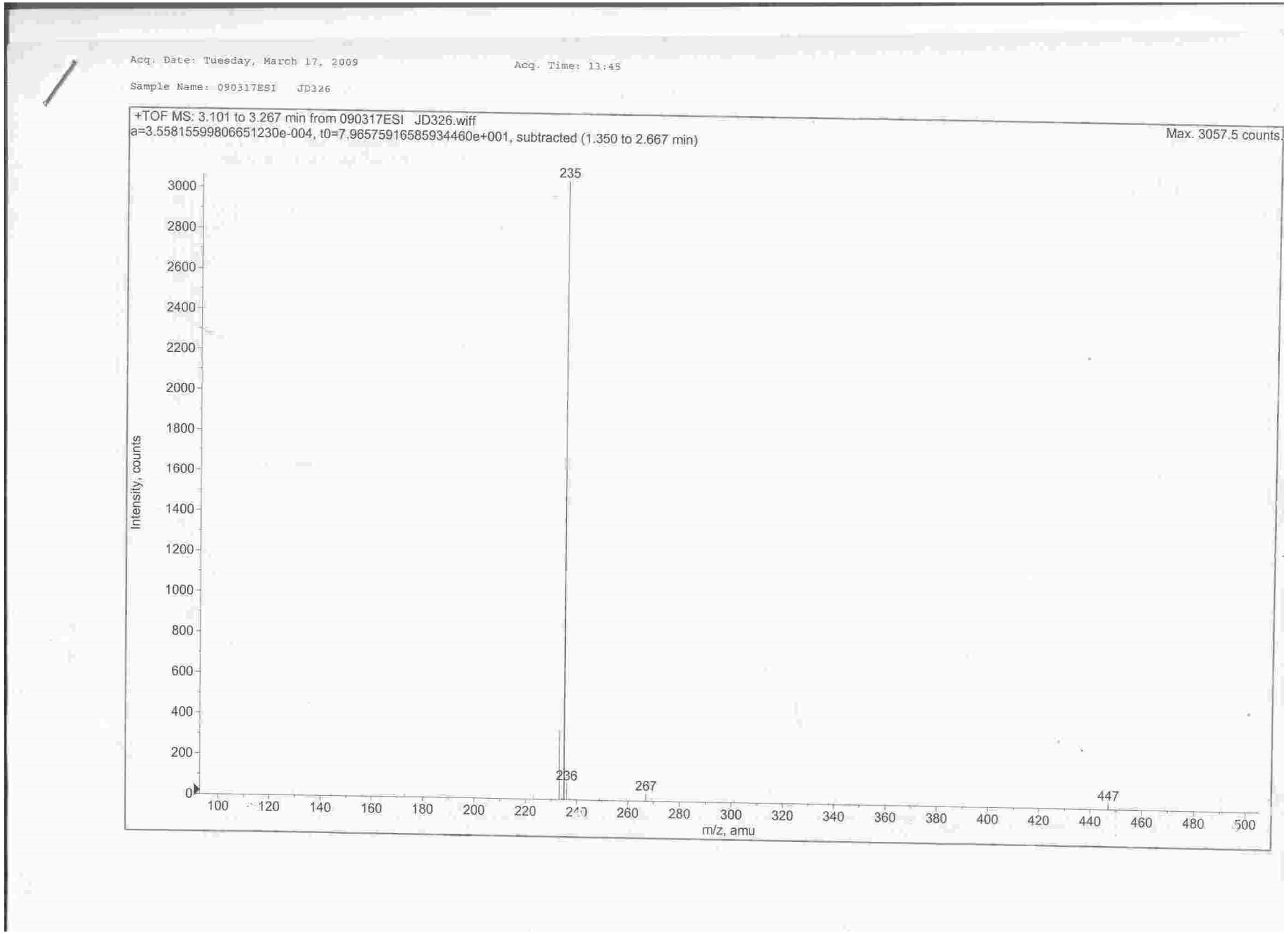

The HRESIMS spectrum of compound **6**

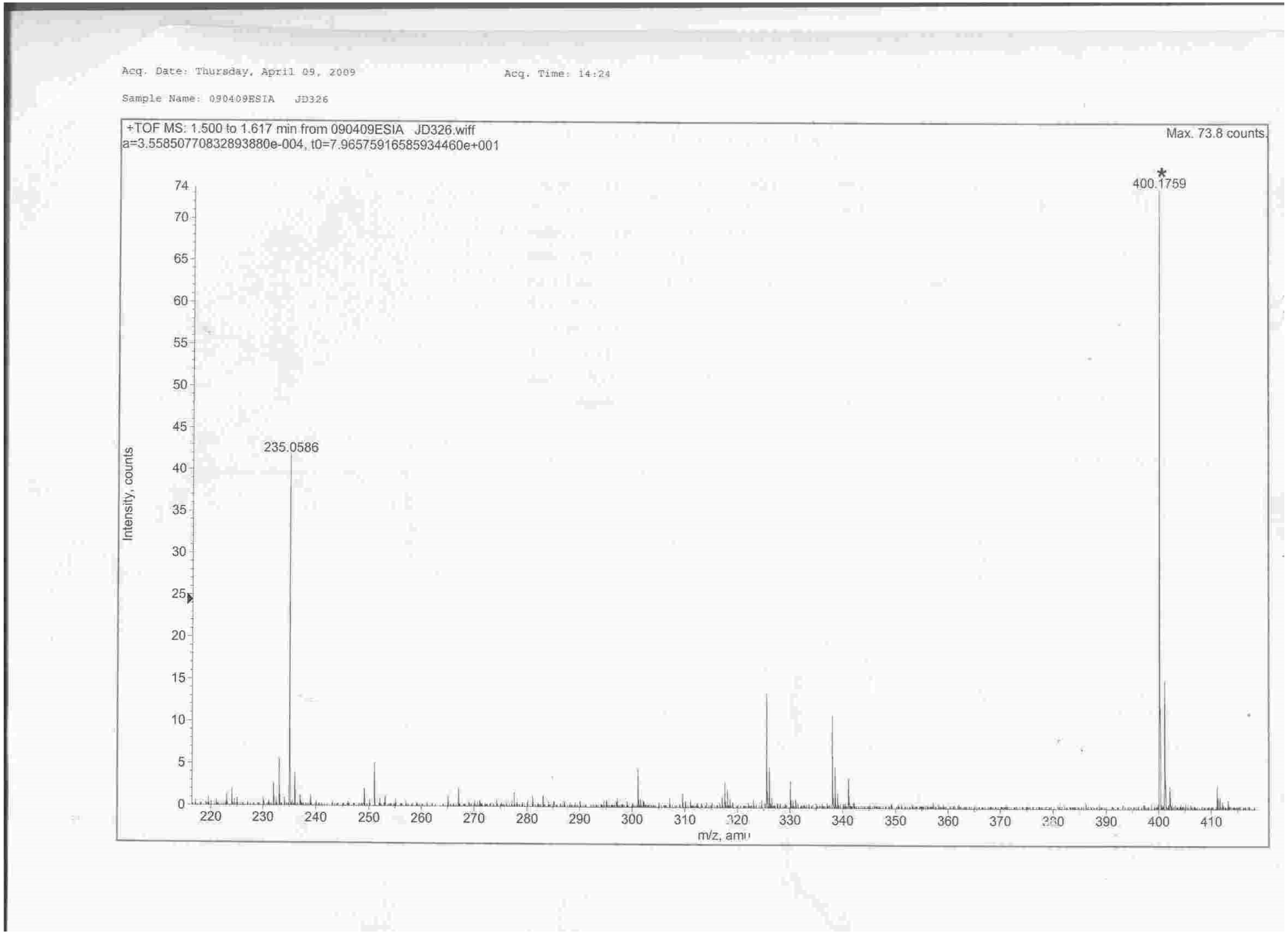

The IR spectrum of compound **6**

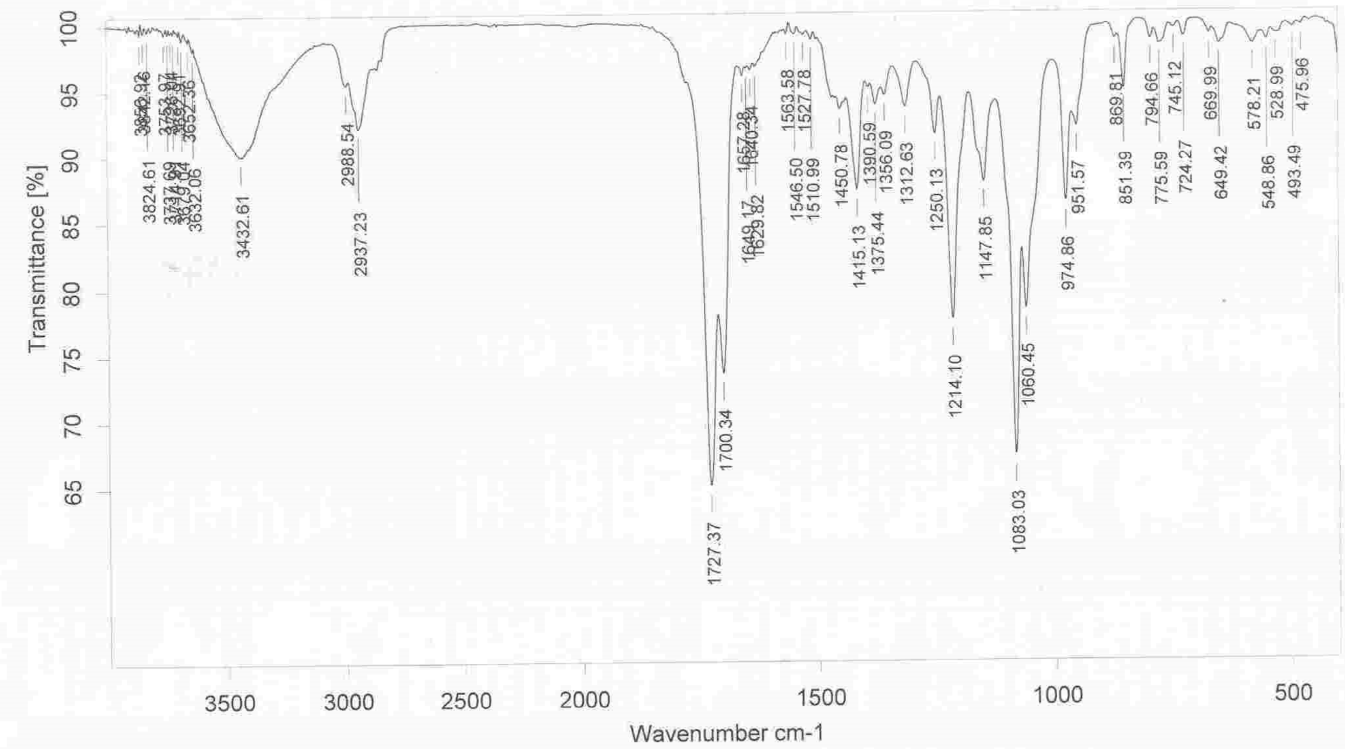

Sample : JD326	Frequency Range : 399.271 - 3996.57		Measured on : 16/04/2009
Technique : KBr压片	Resolution : 4	Instrument : Tensor27	Sample Scans : 16
Customer : 090416IR1	Zerofilling : 2	Acquisition : Double Sided,For	

The UV spectrum of compound **6**

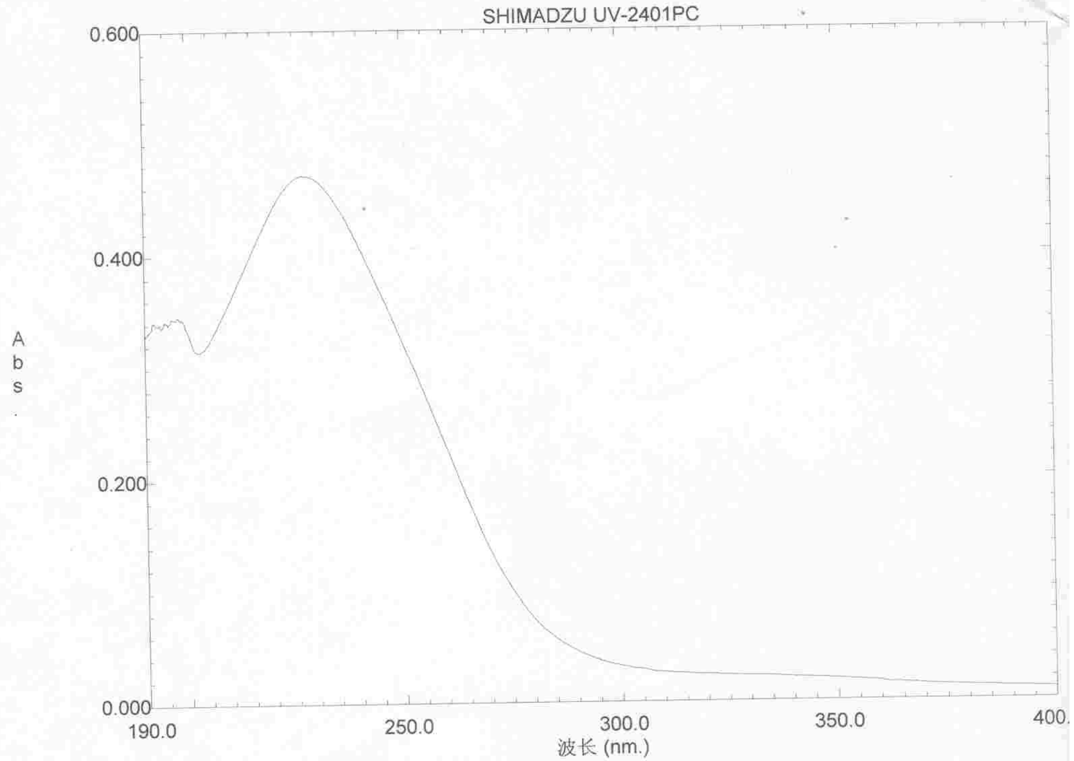

文件名: JD326

JD326

样品浓度：0.0135毫克/毫升
溶剂：甲醇

创建于: 10:23 09-04-13
数据: 原始

测量模式: Abs.
扫描速度: 中速
狭缝: 2.0
采样间隔: 0.2

否.	波长 (nm.)	Abs.
1	227.00	0.4711
2	197.60	0.3464

The $[\alpha]_D$ spectrum of compound **6**

Optical rotation measurement

Model : P-1020 (A060460638)

No.	Sample	Mode	Data	Monitor<br>Blank	Temp.<br>Cell<br>Temp Point	Date<br>Comment<br>Sample Name	Light<br>Filter<br>Operator	Cycle Time<br>Integ Time
No.1	4 (1/3)	Sp.Rot	-3.7230	-0.0035<br>0.0000	24.0<br>50.00<br>Cell	Mon Apr 13 13:19:55 2009<br>0.00188g/mlMeOH<br>JD326	Na<br>589nm	2 sec<br>10 sec
No.2	4 (2/3)	Sp.Rot	-3.5110	-0.0033<br>0.0000	24.0<br>50.00<br>Cell	Mon Apr 13 13:20:08 2009<br>0.00188g/mlMeOH<br>JD326	Na<br>589nm	2 sec<br>10 sec
No.3	4 (3/3)	Sp.Rot	-4.6810	-0.0044<br>0.0000	24.0<br>50.00<br>Cell	Mon Apr 13 13:20:21 2009<br>0.00188g/mlMeOH<br>JD326	Na<br>589nm	2 sec<br>10 sec

-3.9716°

The ^{1}H NMR spectrum of compound **7** in pyridine-d_5

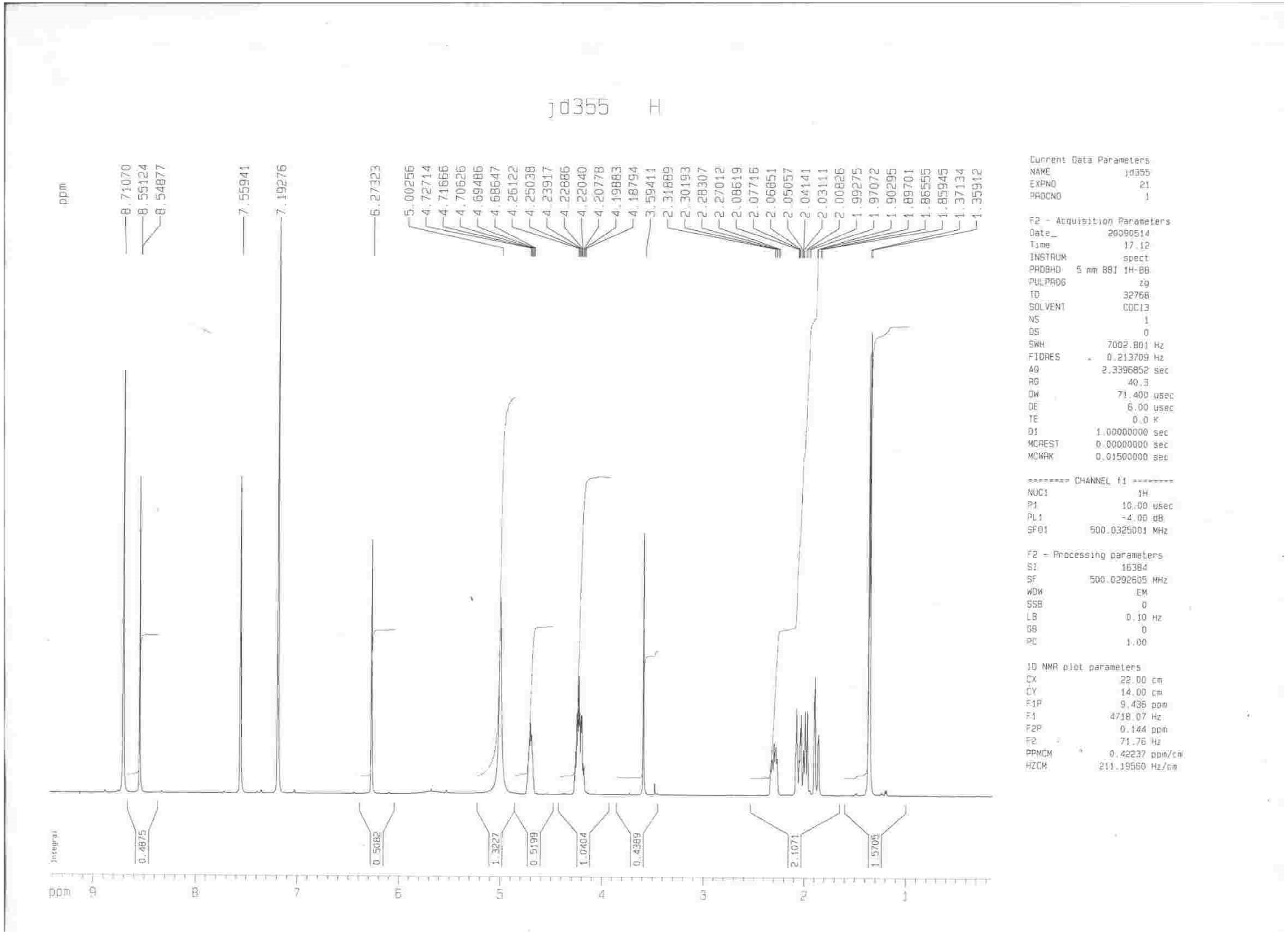

The ^{13}C NMR spectrum of compound **7** in pyridine-d_5

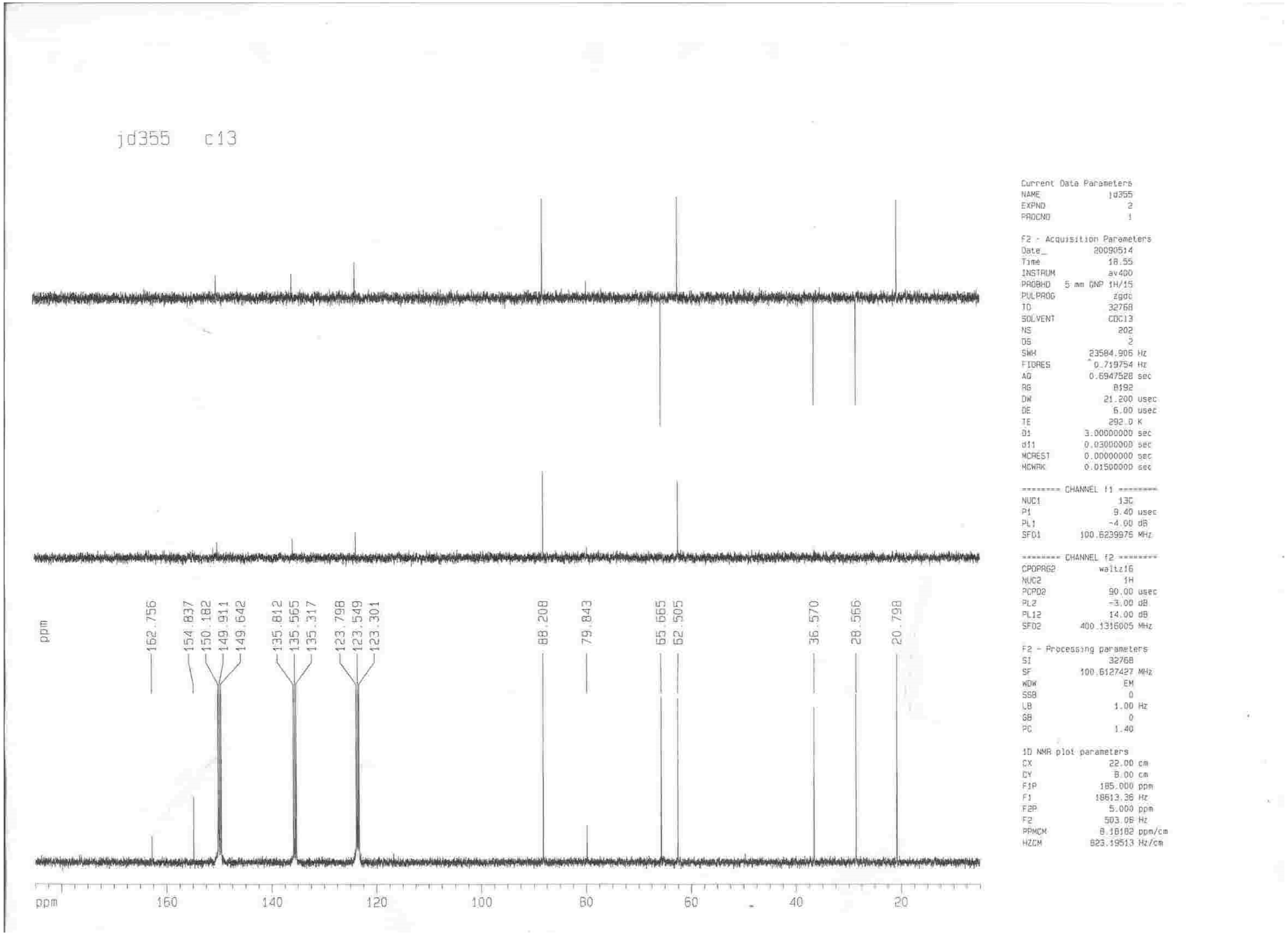

The HSQC spectrum of compound **7** in pyridine-d_5

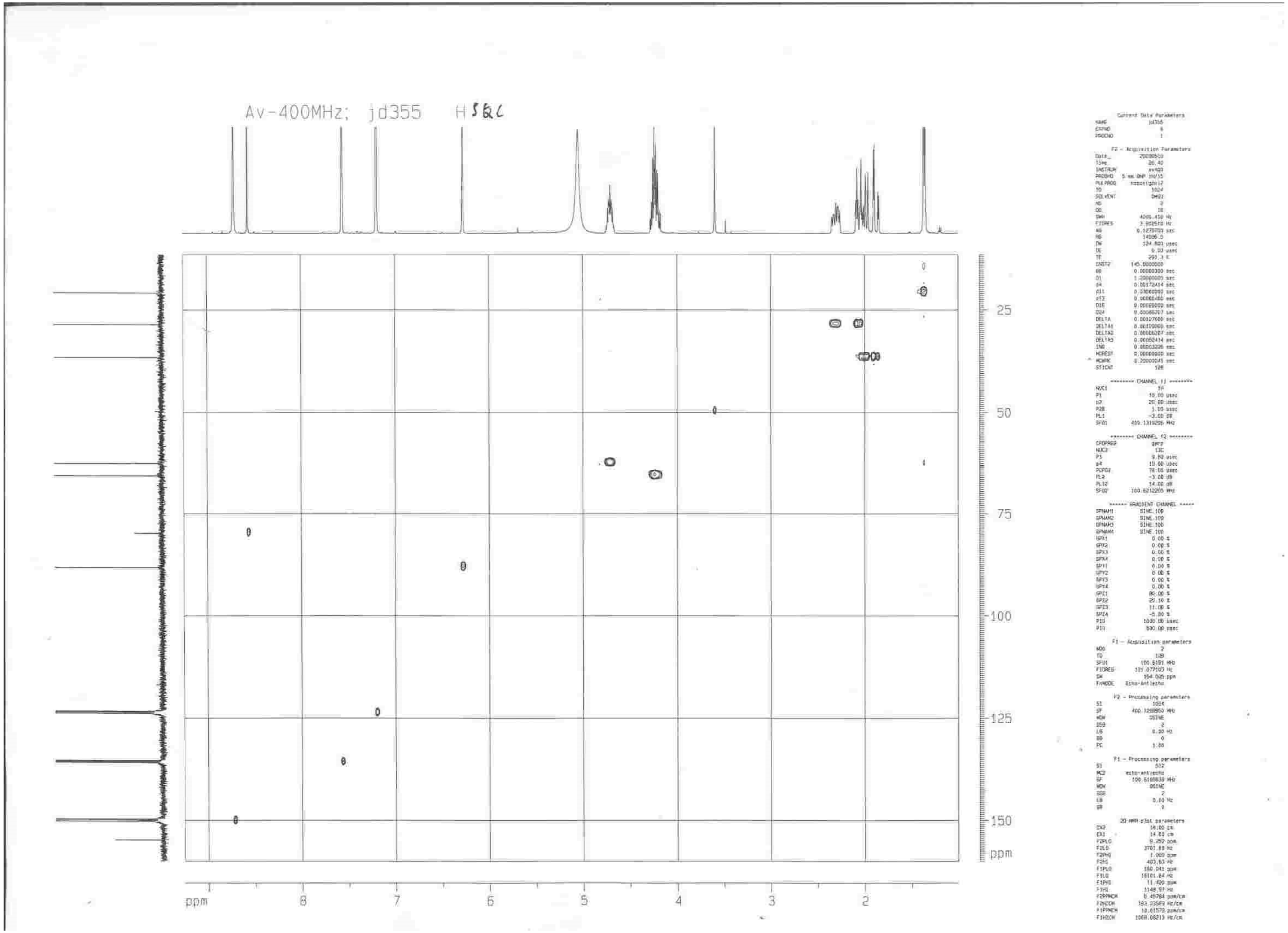

The HMBC spectrum of compound **7** in pyridine-d_5

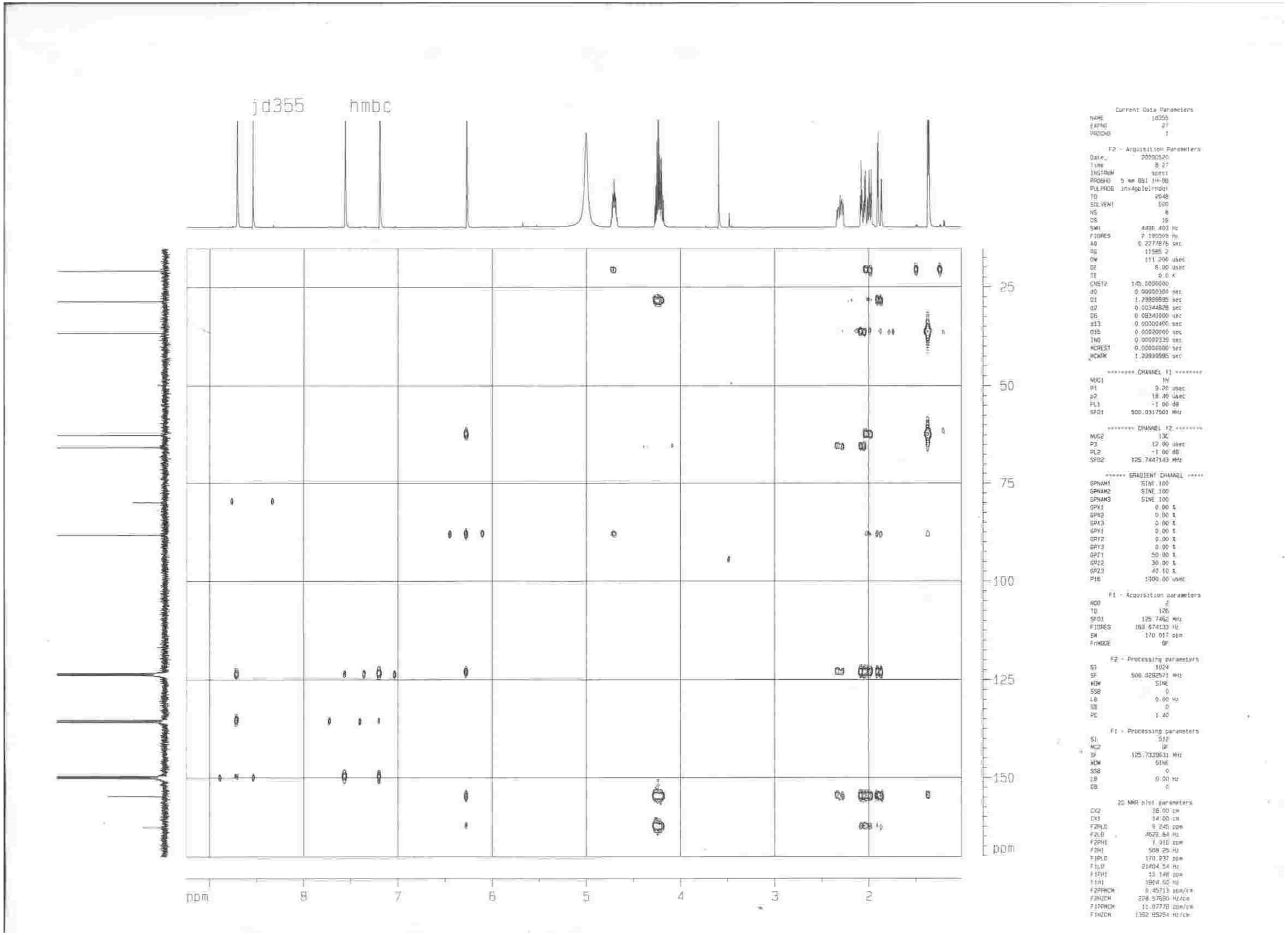

The ^{1}H ^{1}H COSY spectrum of compound **7** in pyridine-d_5

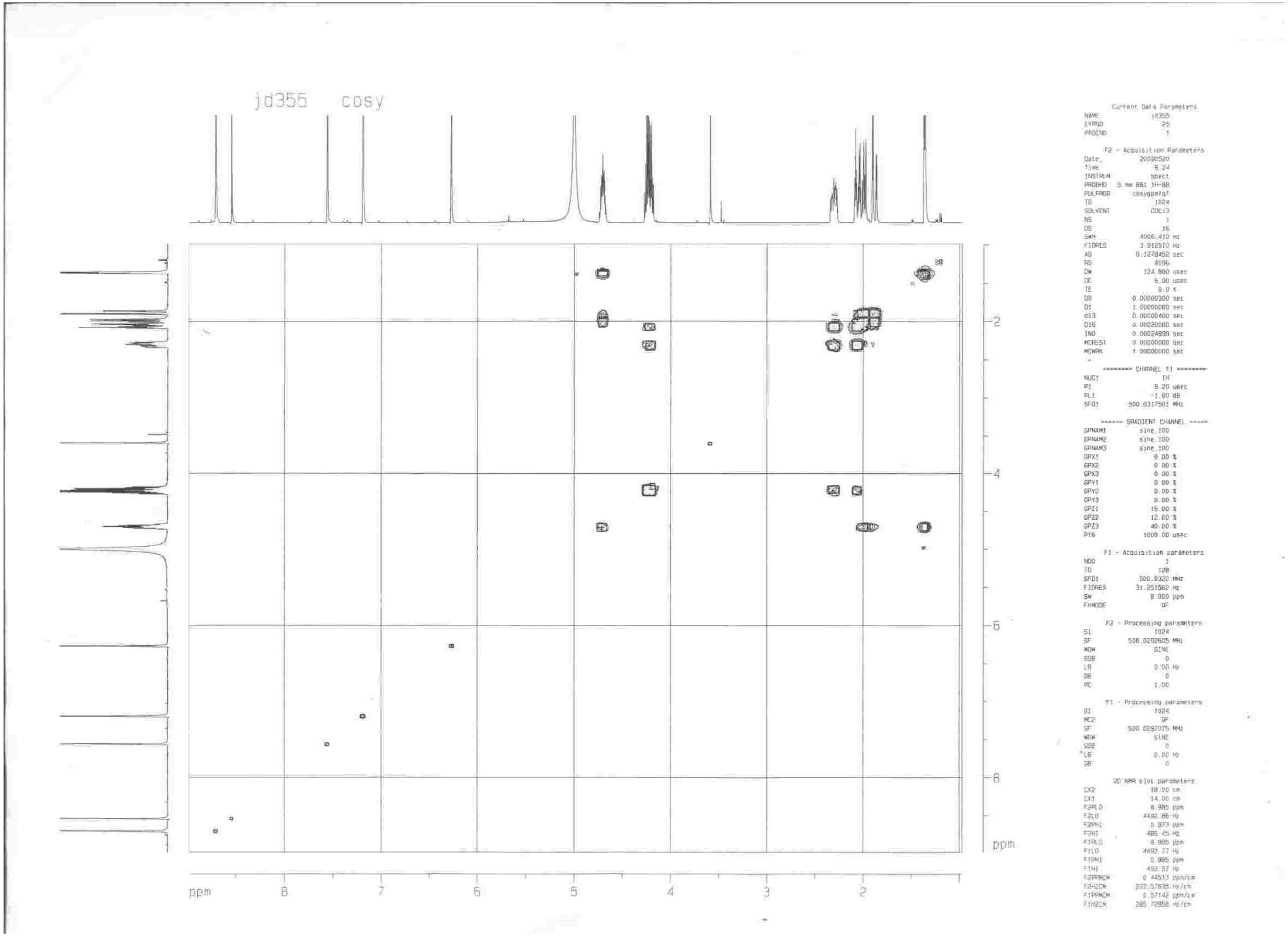

The ROESY spectrum of compound **7** in pyridine-d_5

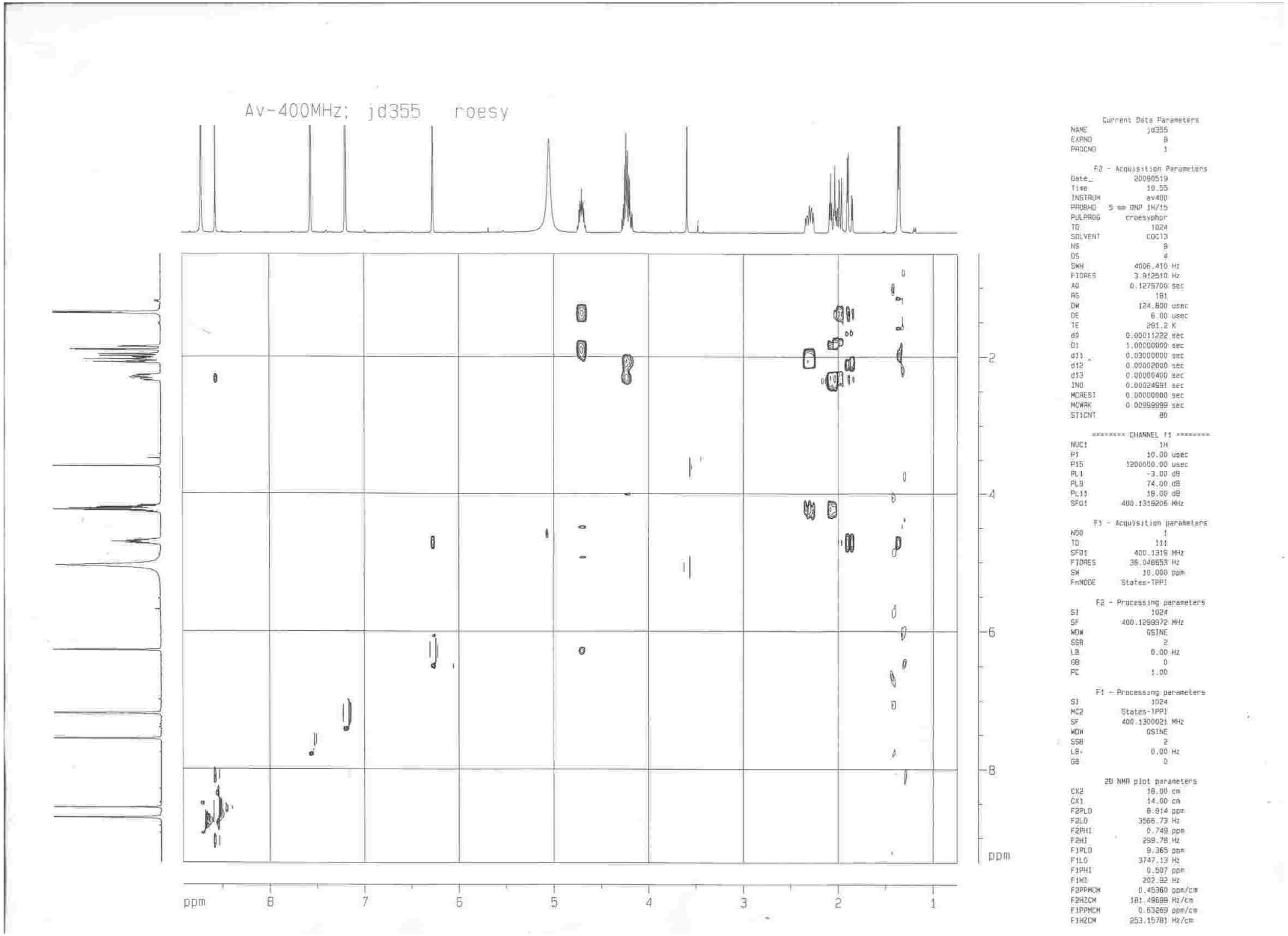

The ESIMS spectrum of compound **7**

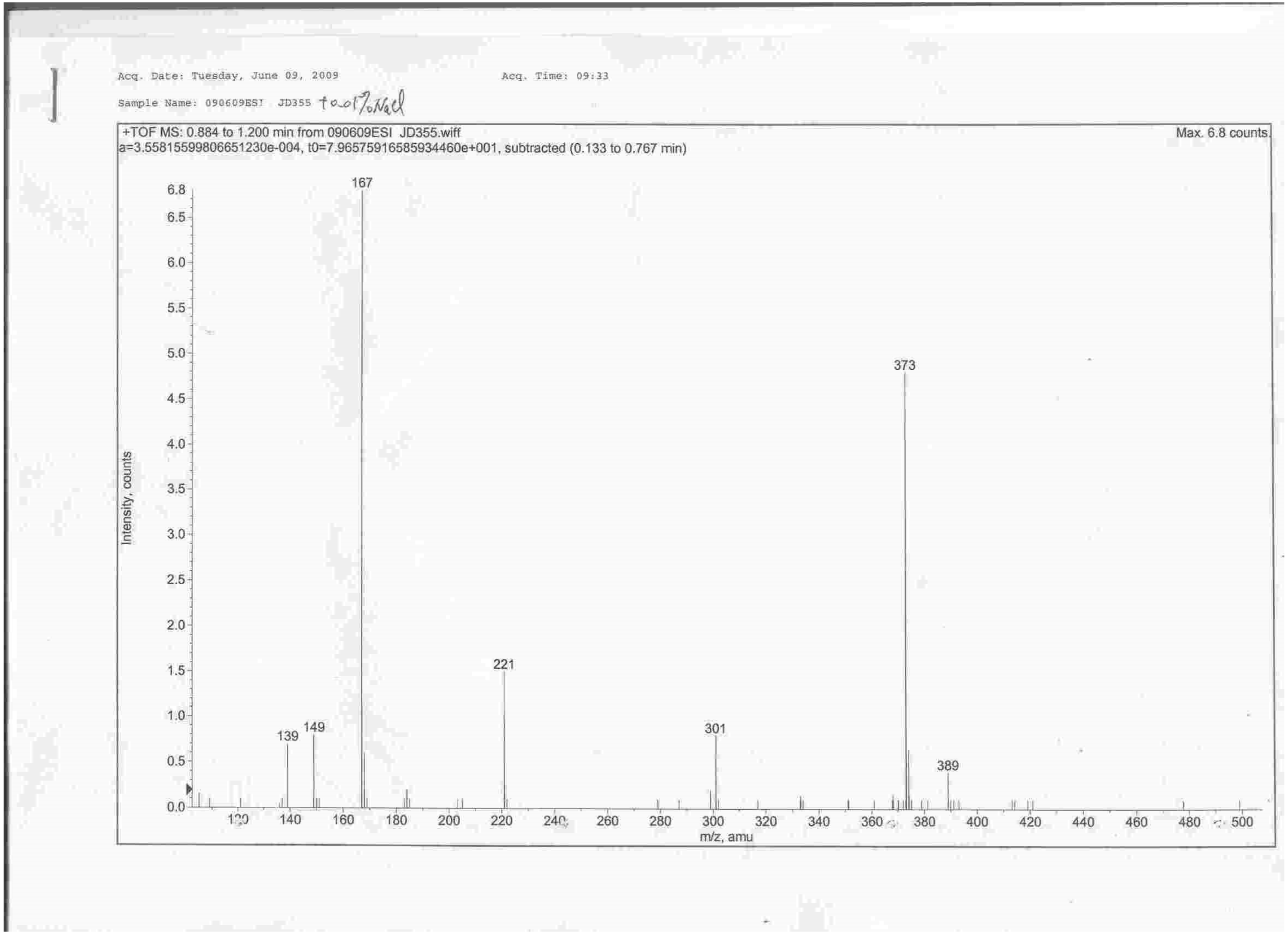

The HRESIMS spectrum of compound **7**

Acq. Date: Tuesday, June 09, 2009 Acq. Time: 16:59

Sample Name: 090609ESIA JD355

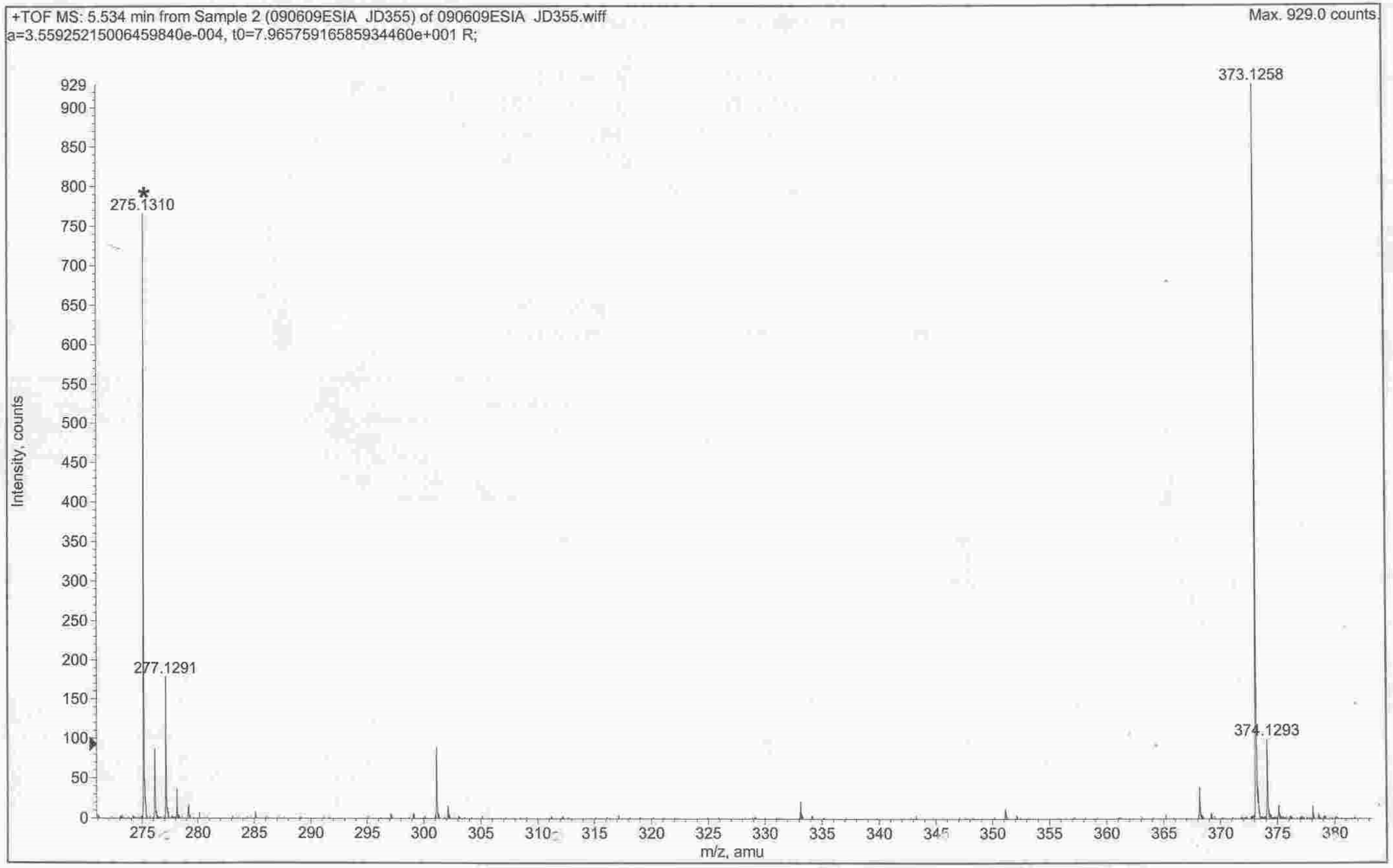

The IR spectrum of compound **7**

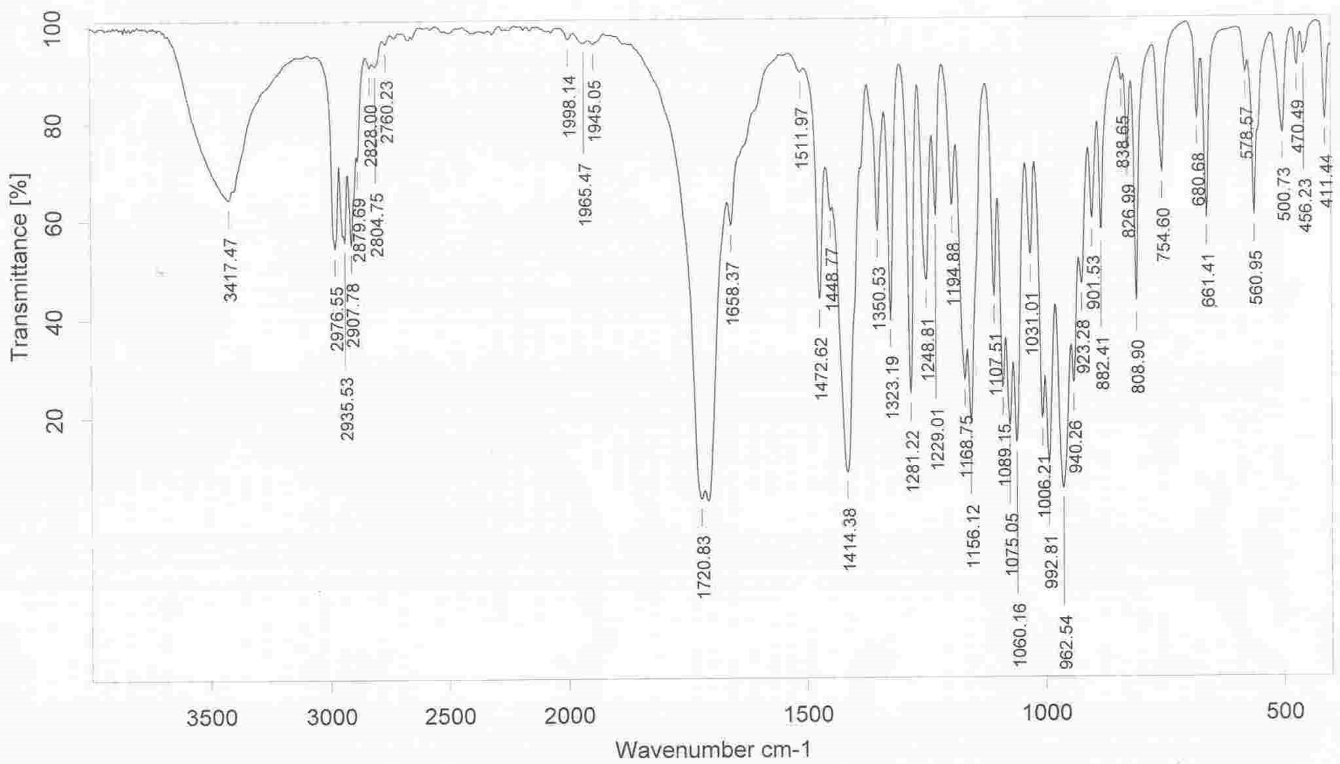

Sample : JD355	Frequency Range : 399.271 - 3996.57	Measured on : 10/06/2009	
Technique : KBr压片	Resolution : 4	Instrument : Tensor27	Sample Scans : 16
Customer : 090610IR1	Zerofilling : 2	Acquisition : Double Sided,For	

The UV spectrum of compound **7**

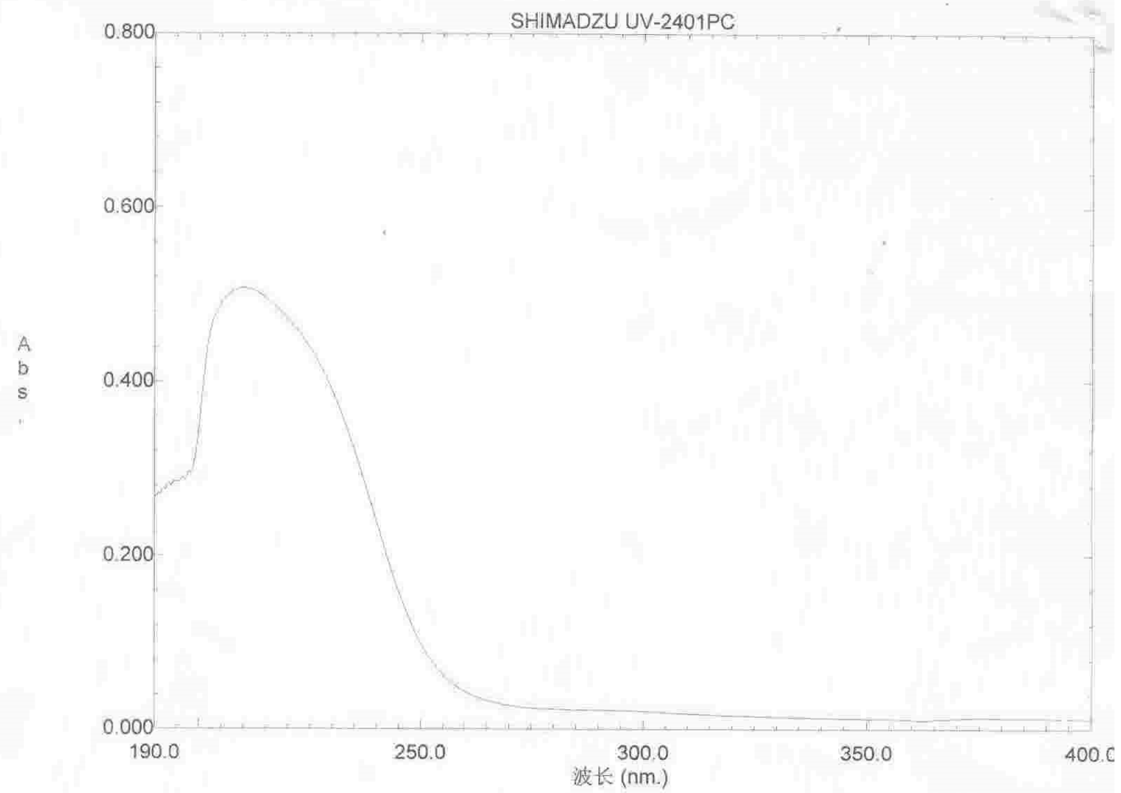

文件名: JD355

JD355 ———

样品浓度：0.0107毫克/毫升
溶剂：甲醇

创建于: 15:06 09-06-08
数据: 原始

测量模式: Abs.
扫描速度: 中速
狭缝: 2.0
采样间隔: 0.2

否.	波长 (nm.)	Abs.
1	210.20	0.5078

The $[\alpha]_D$ spectrum of compound **7**

Optical rotation measurement

Model : P-1020 (A060460638)

No.	Sample	Mode	Data	Monitor<br>Blank	Temp.<br>Cell<br>Temp Point	Date<br>Comment<br>Sample Name	Light<br>Filter<br>Operator	Cycle Time<br>Integ Time
No.1	3 (1/3)	Sp.Rot	-6.7540	-0.0090<br>0.0000	25.6<br>50.00<br>Cell	Tue Jun 09 15:41:38 2009<br>0.00267g/mlMeOH+CHCl3<br>JD355	Na<br>589nm	2 sec<br>10 sec
No.2	3 (2/3)	Sp.Rot	-6.7540	-0.0090<br>0.0000	25.7<br>50.00<br>Cell	Tue Jun 09 15:41:51 2009<br>0.00267g/mlMeOH+CHCl3<br>JD355	Na<br>589nm	2 sec<br>10 sec
No.3	3 (3/3)	Sp.Rot	-6.4540	-0.0086<br>0.0000	25.7<br>50.00<br>Cell	Tue Jun 09 15:42:04 2009<br>0.00267g/mlMeOH+CHCl3<br>JD355	Na<br>589nm	2 sec<br>10 sec

−6.6542°

The ^{1}H NMR spectrum of compound **8** in $CDCl_3$

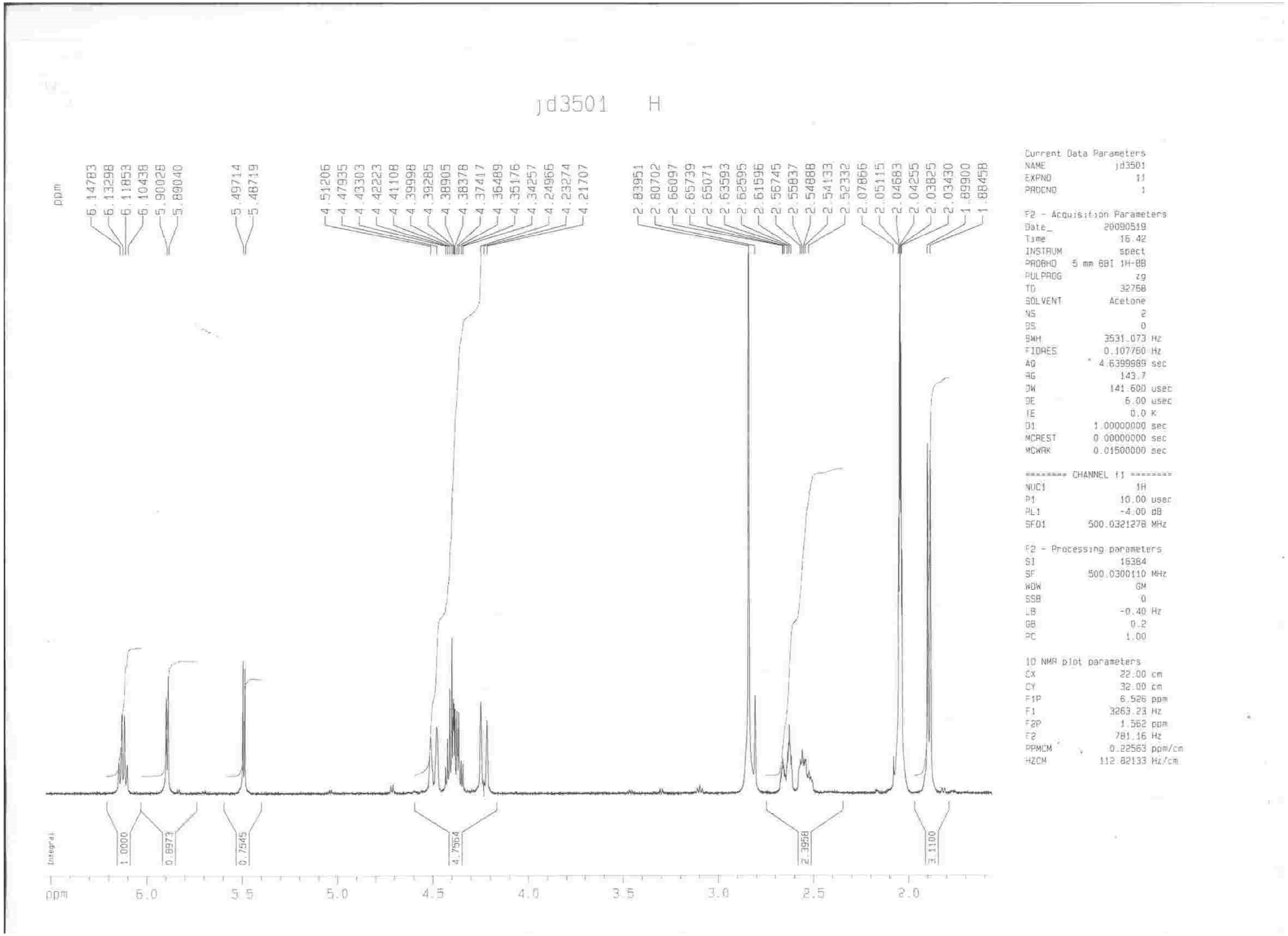

The ^{13}C NMR spectrum of compound **8** in $CDCl_3$

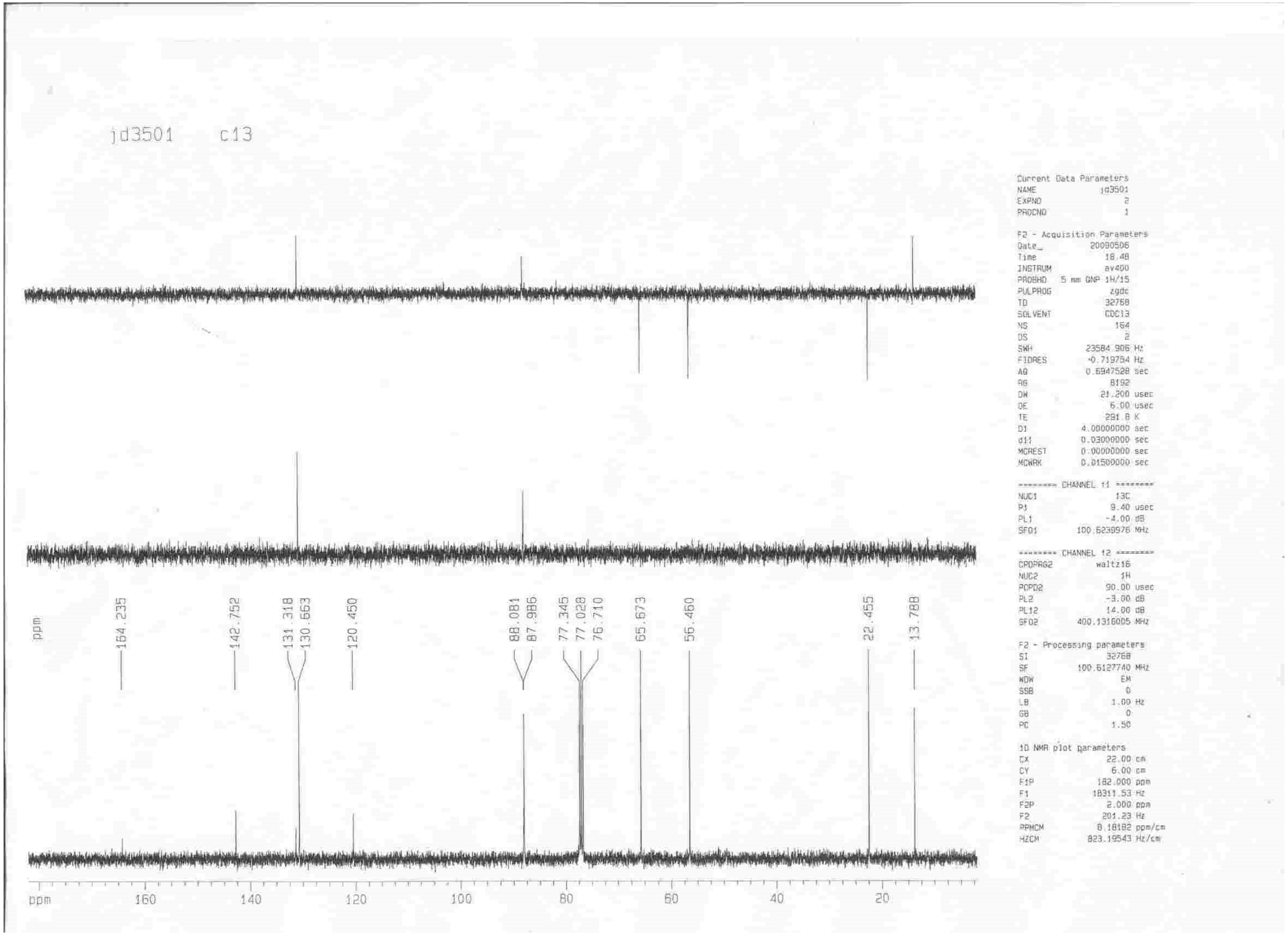

The HSQC spectrum of compound **8** in $CDCl_3$

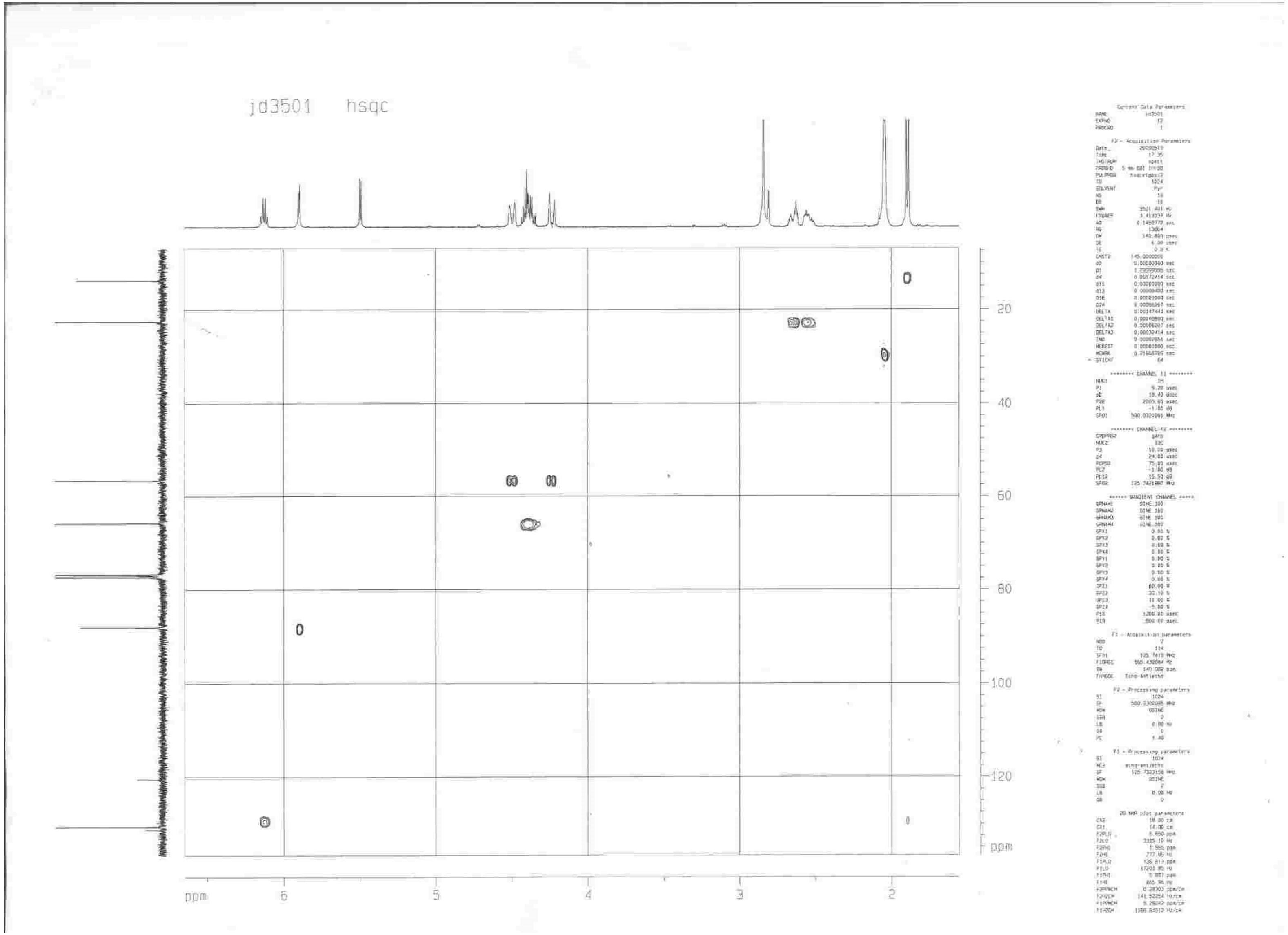

The HMBC spectrum of compound **8** in $CDCl_3$

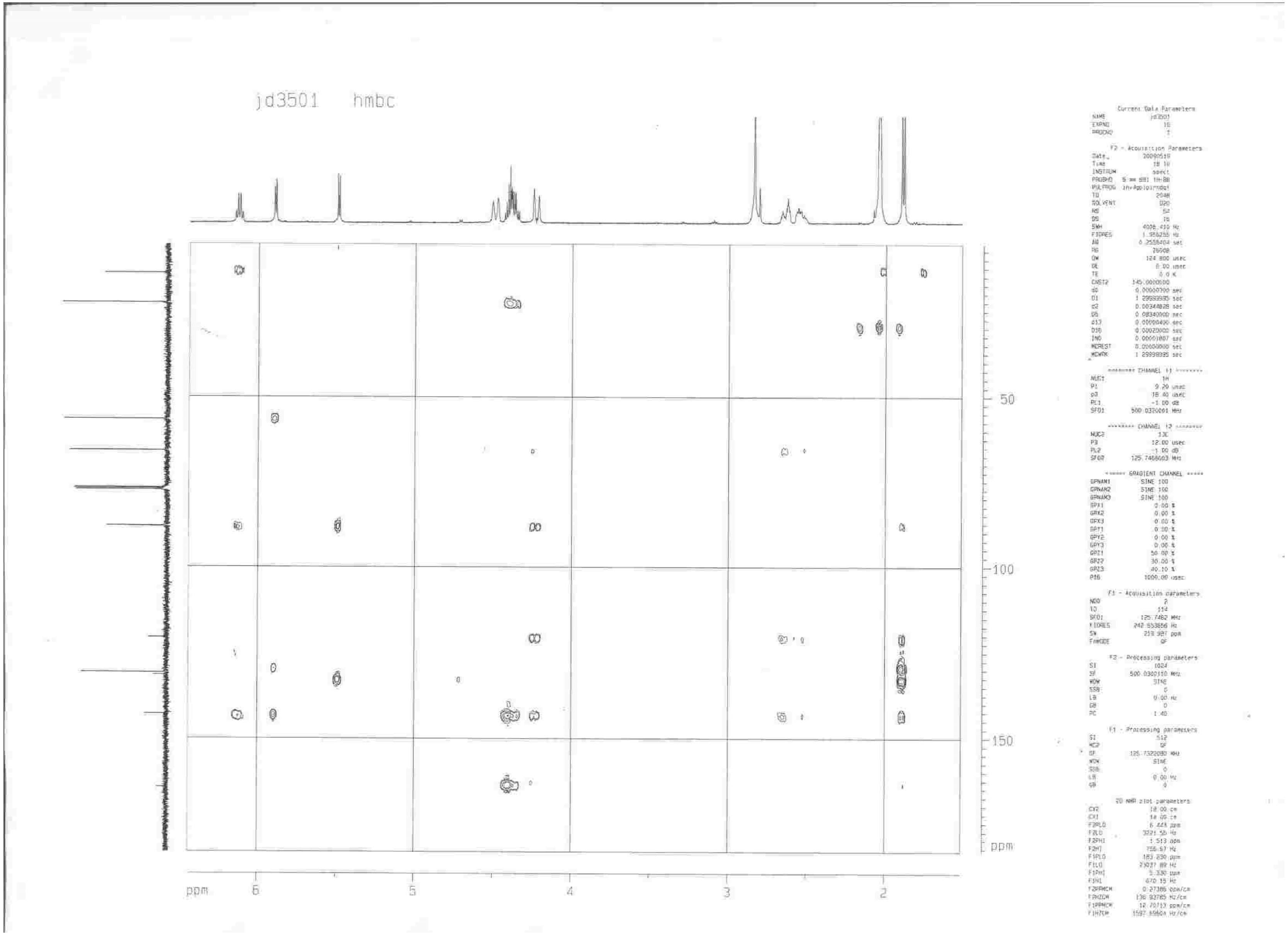

The ^{1}H ^{1}H COSY spectrum of compound **8** in $CDCl_3$

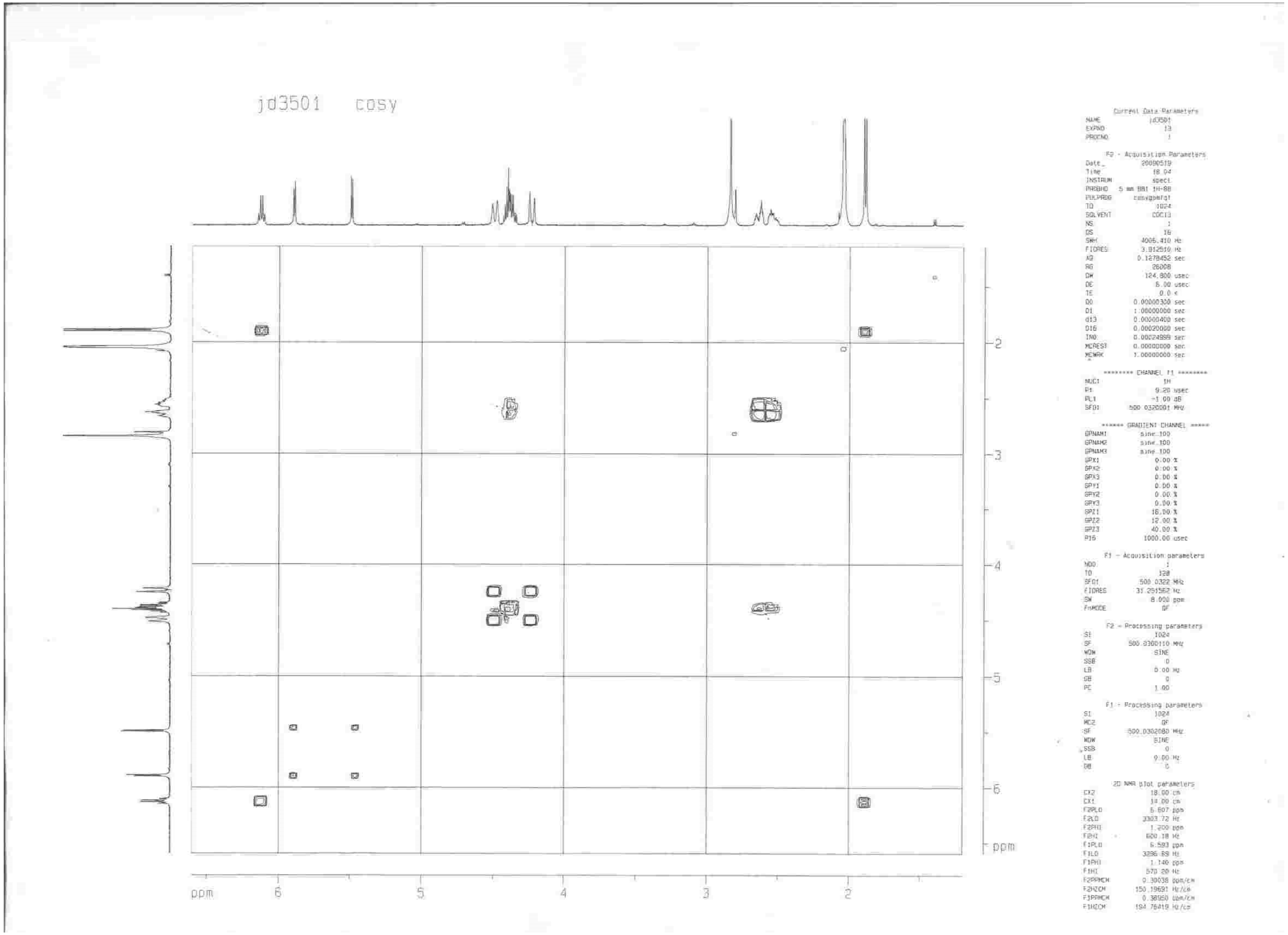

The ROESY spectrum of compound **8** in $CDCl_3$

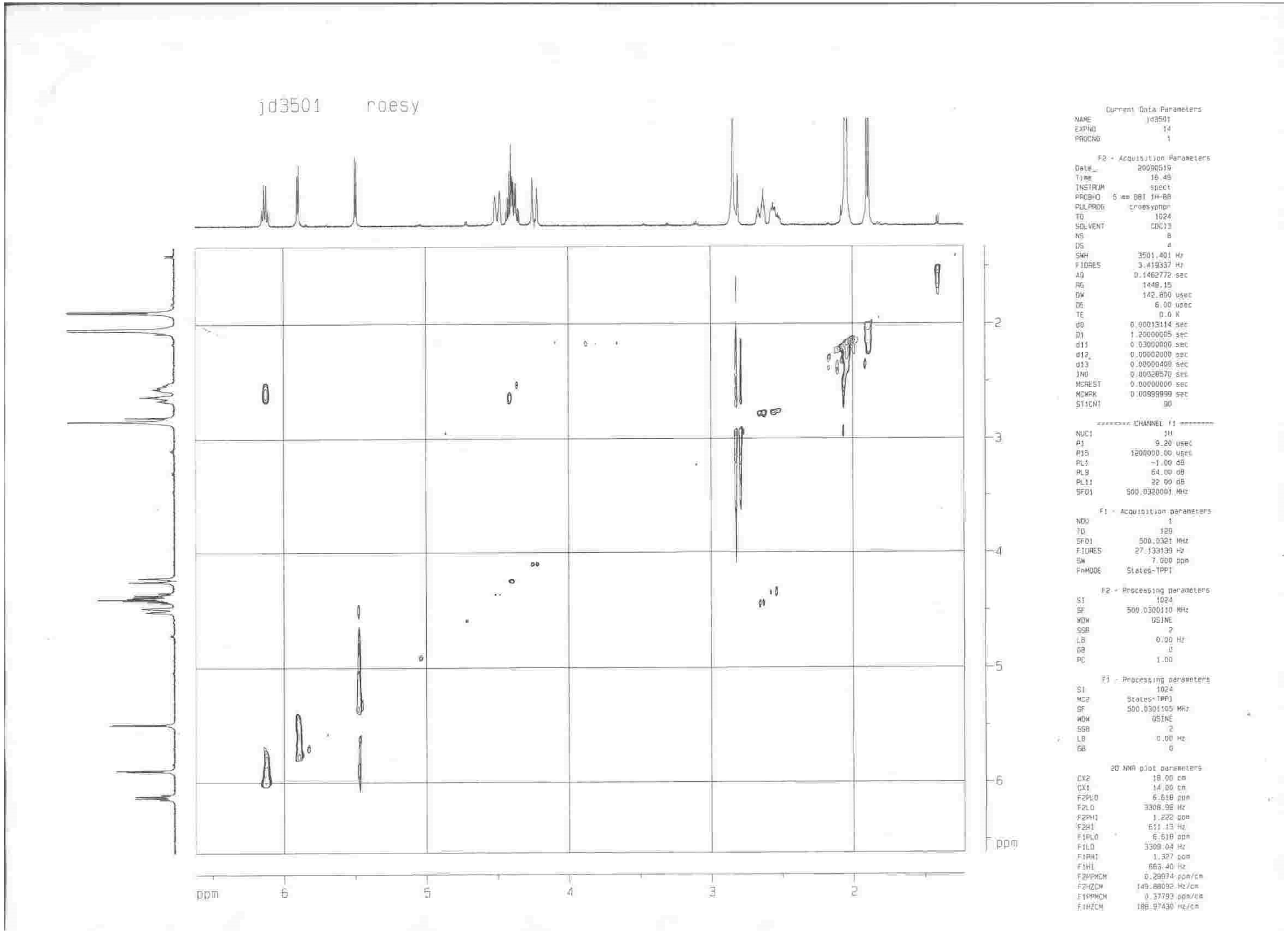

The EIMS spectrum of compound **8**

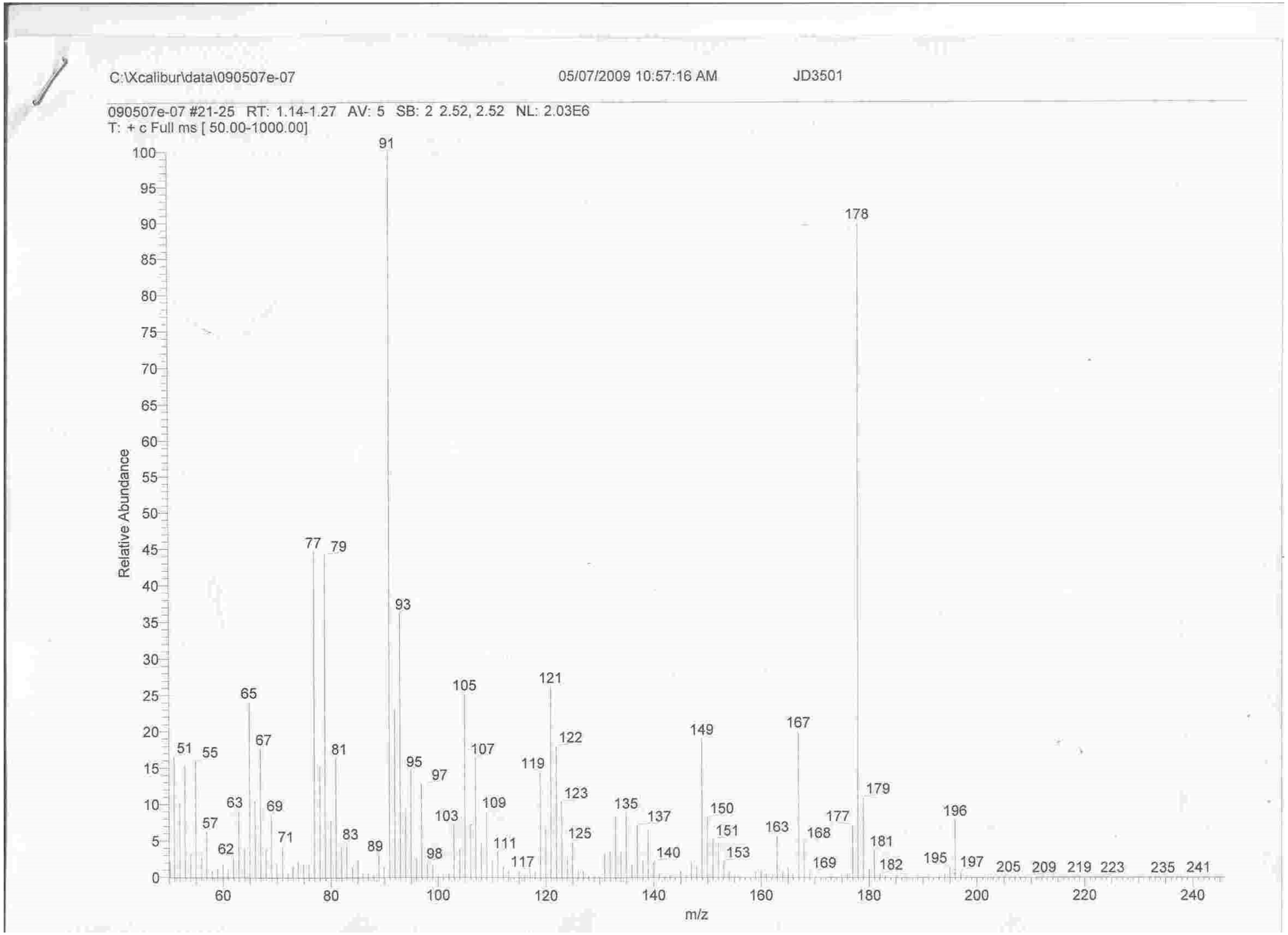

The HRESIMS spectrum of compound **8**

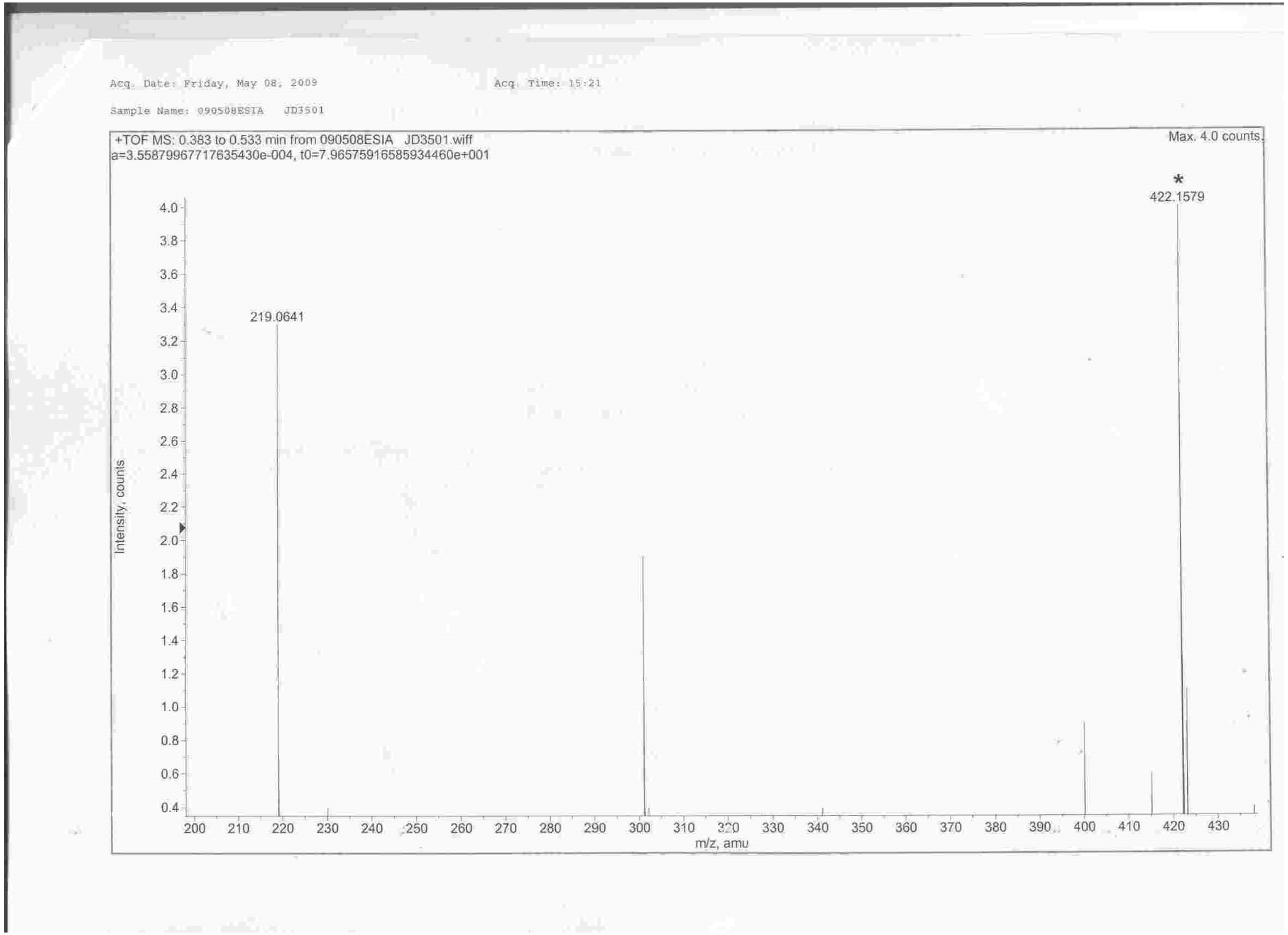

The IR spectrum of compound **8**

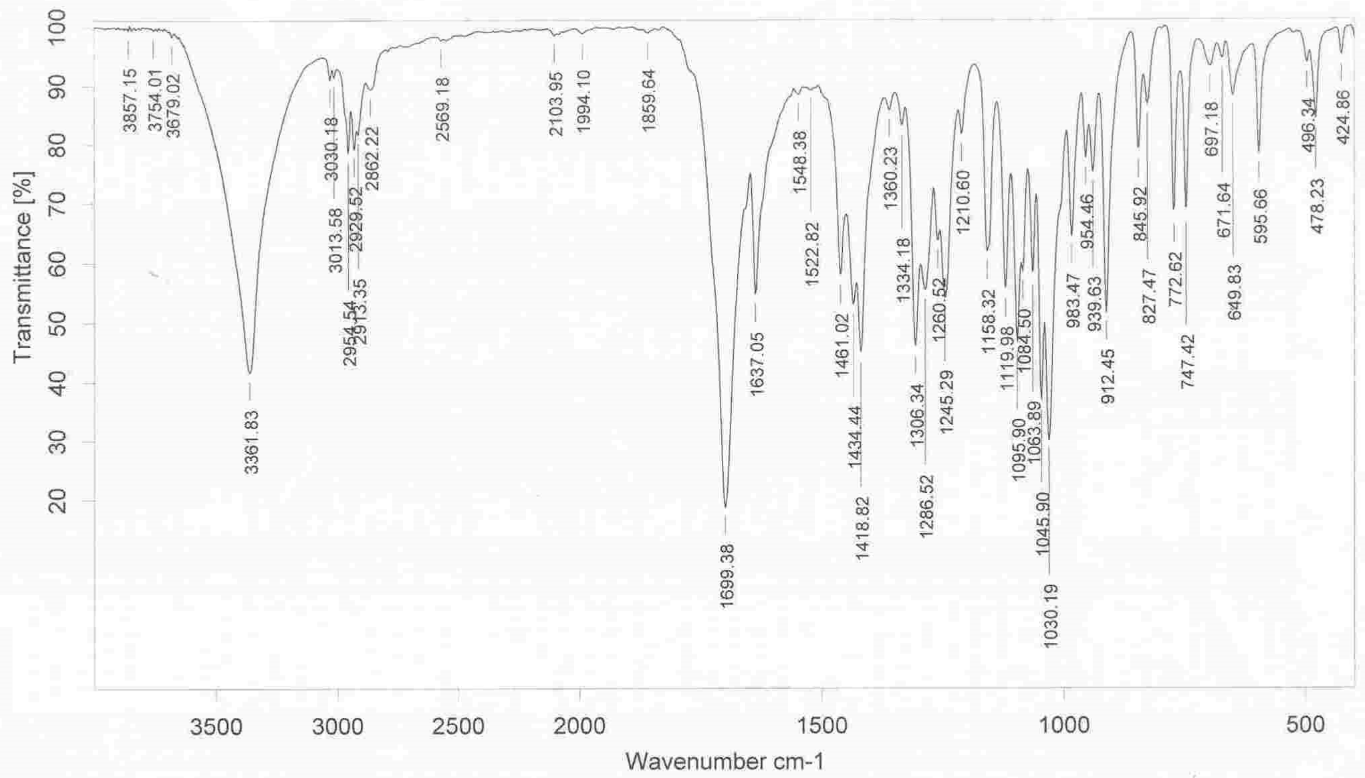

Sample : JD3501	Frequency Range : 399.271 - 3996.57		Measured on : 03/06/2009
Technique : KBr压片	Resolution : 4	Instrument : Tensor27	Sample Scans : 16
Customer : 090603IR2	Zerofilling : 2	Acquisition : Double Sided,For	

The UV spectrum of compound **8**

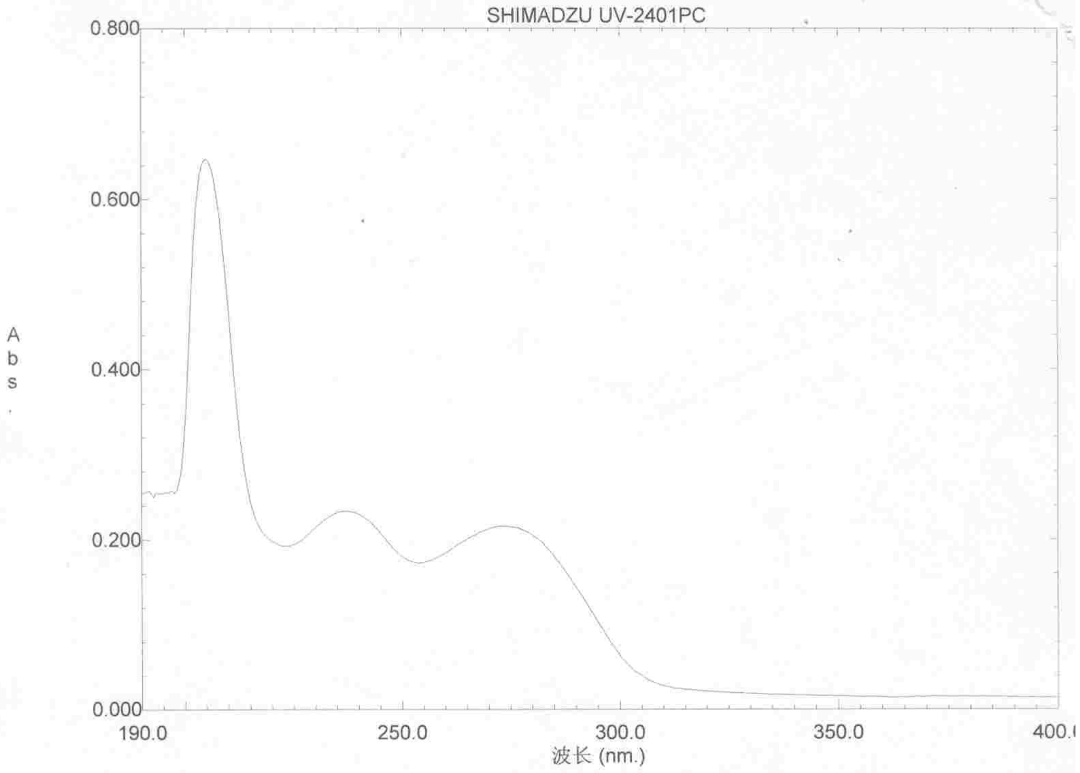

文件名: JD3501

JD3501 ———

样品浓度：0.0070毫克/毫升
溶剂：甲醇

创建于: 15:39 09-06-02
数据: 原始

测量模式: Abs.
扫描速度: 中速
狭缝: 2.0
采样间隔: 0.2

否.	波长 (nm.)	Abs.
1	273.20	0.2156
2	237.00	0.2334
3	205.00	0.6470

The $[\alpha]_D$ spectrum of compound **8**

Optical rotation measurement

Model : P-1020 (A060460638)

No.	Sample	Mode	Data	Monitor<br>Blank	Temp.<br>Cell<br>Temp Point	Date<br>Comment<br>Sample Name	Light<br>Filter<br>Operator	Cycle Time<br>Integ Time	
No.1	3 (1/3)	Sp.Rot	-2.7320	-0.0050<br>0.0000	25.2<br>50.00<br>Cell	Wed Jun 03 13:47:29 2009<br>0.00366g/mlMeOH<br>JD3501	Na<br>589nm	2 sec<br>10 sec	
No.2	3 (2/3)	Sp.Rot	-6.7210	-0.0123<br>0.0000	25.2<br>50.00<br>Cell	Wed Jun 03 13:47:42 2009<br>0.00366g/mlMeOH<br>JD3501	Na<br>589nm	2 sec<br>10 sec	-3.8977
No.3	3 (3/3)	Sp.Rot	-2.2400	-0.0041<br>0.0000	25.3<br>50.00<br>Cell	Wed Jun 03 13:47:56 2009<br>0.00366g/mlMeOH<br>JD3501	Na<br>589nm	2 sec<br>10 sec	

The ^{1}H NMR spectrum of compound **9** in $CDCl_3$

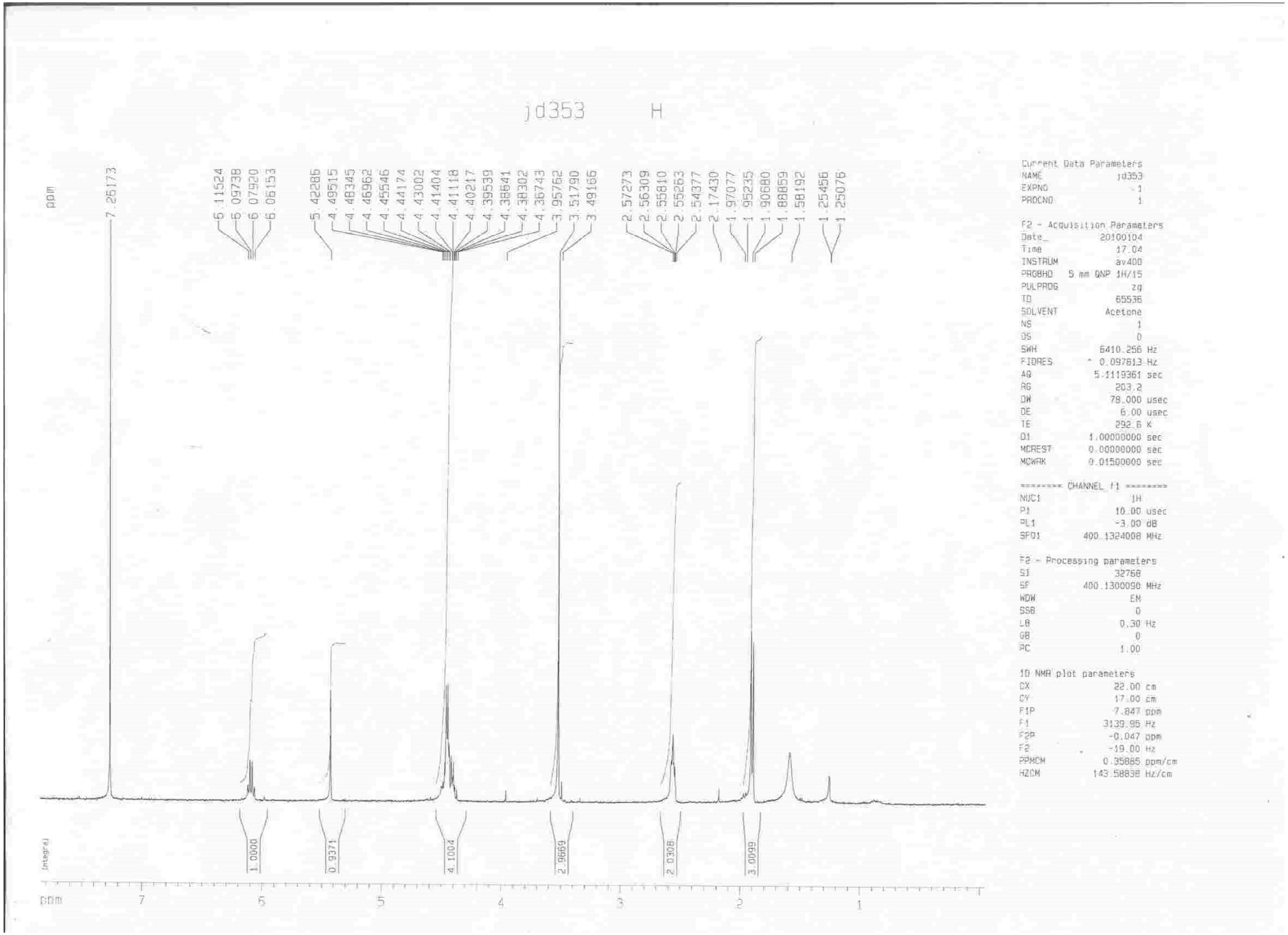

The ^{13}C NMR spectrum of compound **9** in $CDCl_3$

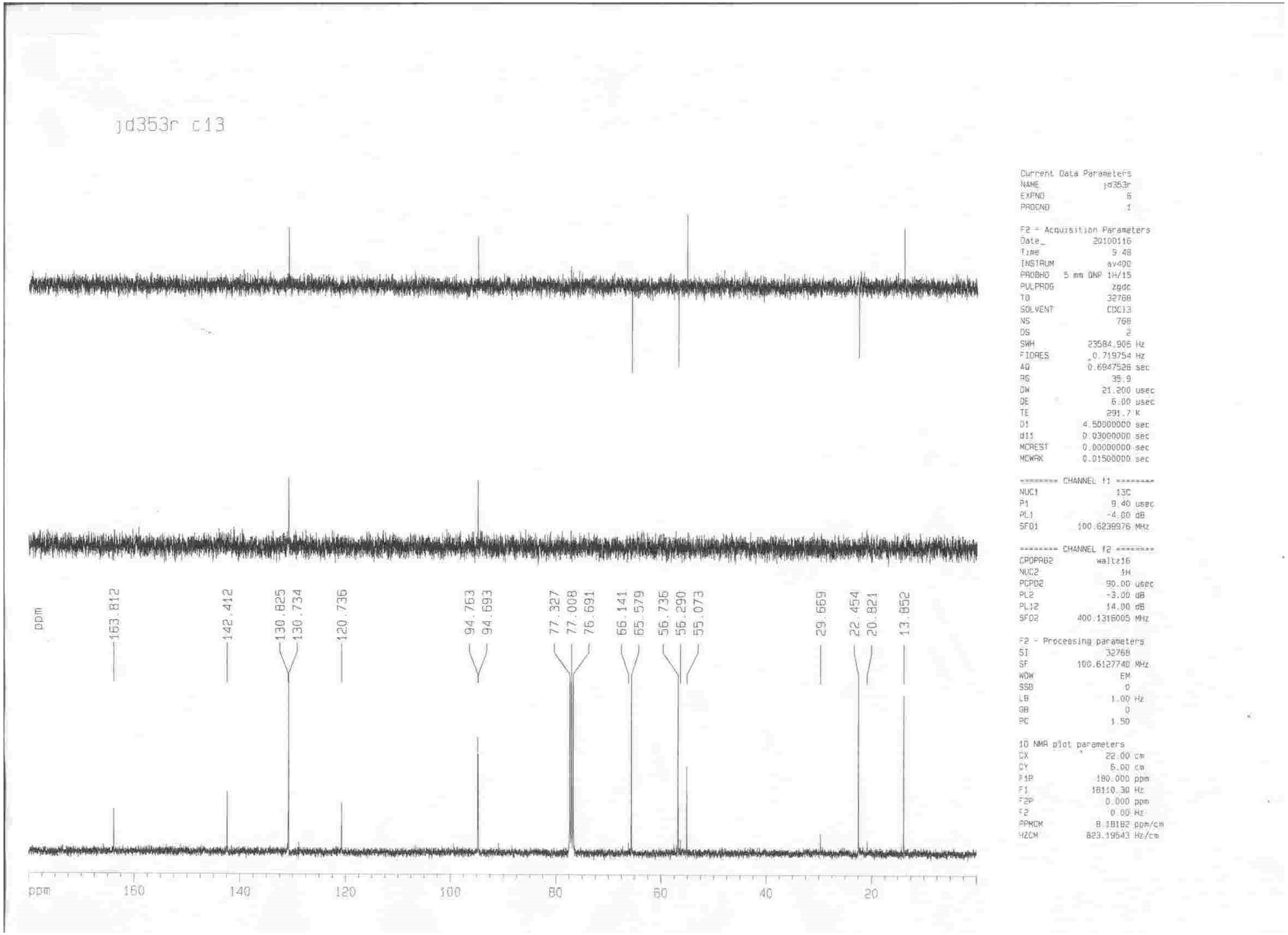

The HSQC spectrum of compound **9** in $CDCl_3$

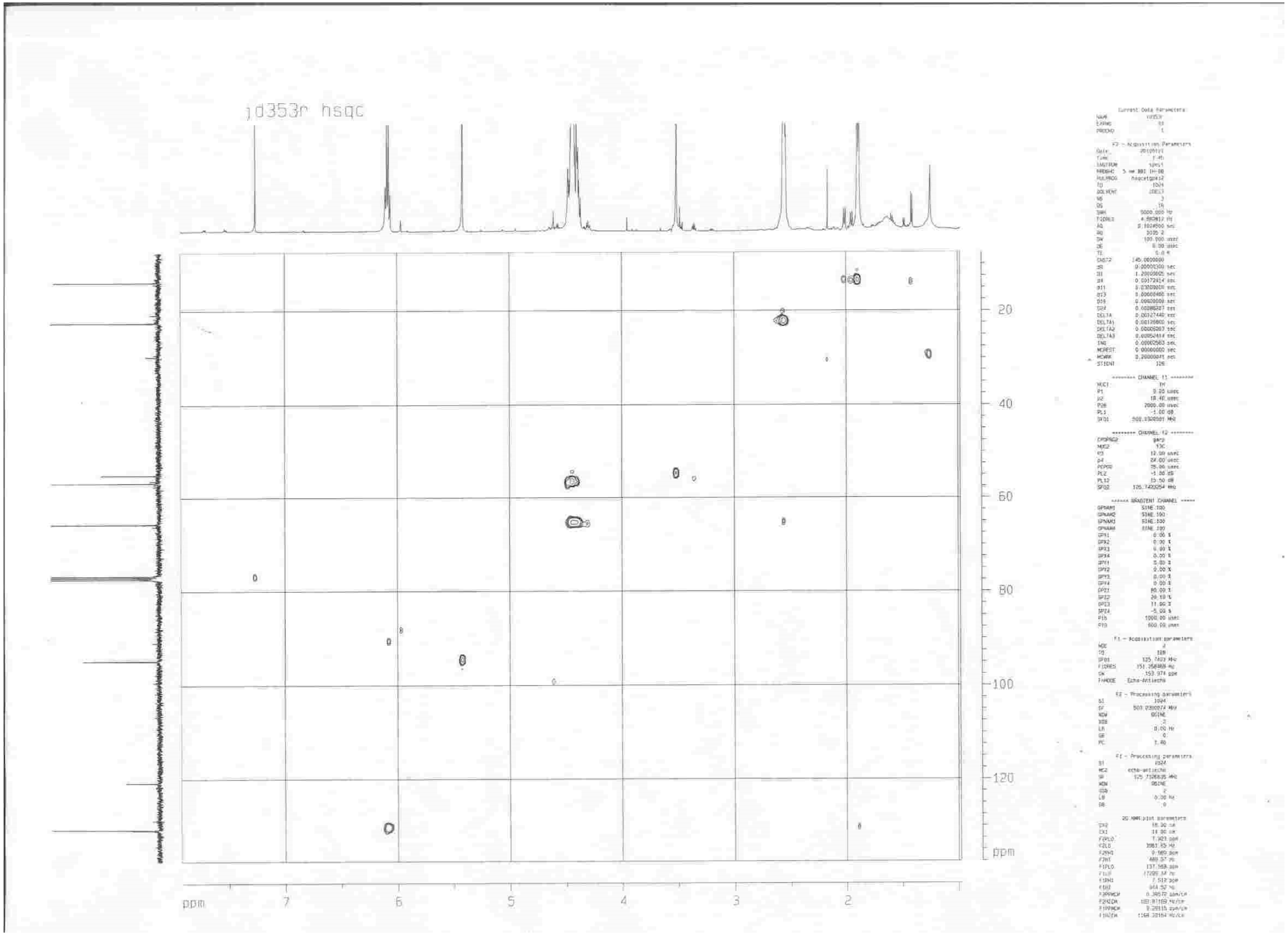

The HMBC spectrum of compound **9** in $CDCl_3$

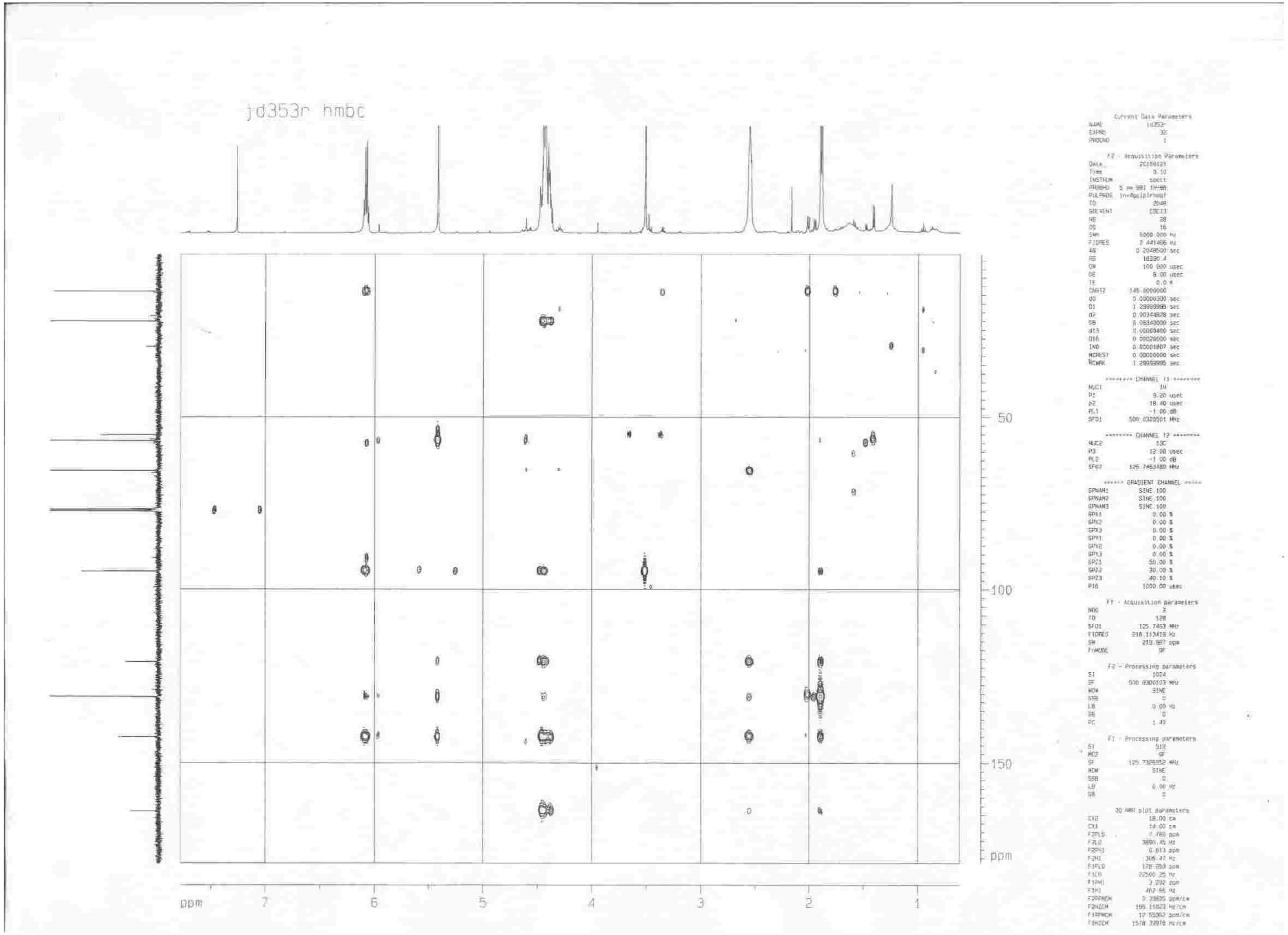

The ^{1}H ^{1}H COSY spectrum of compound **9** in $CDCl_3$

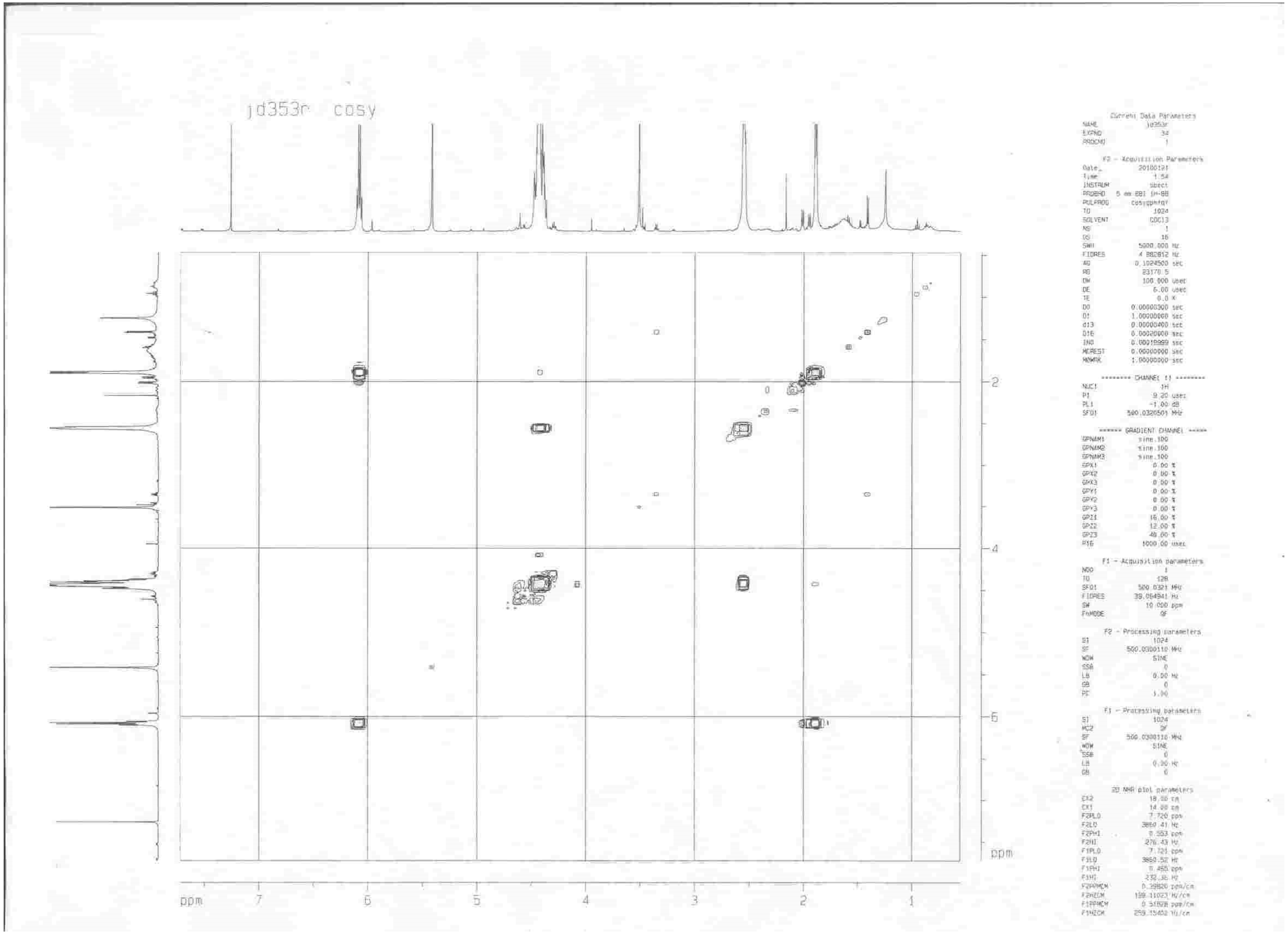

The ROESY spectrum of compound **9** in $CDCl_3$

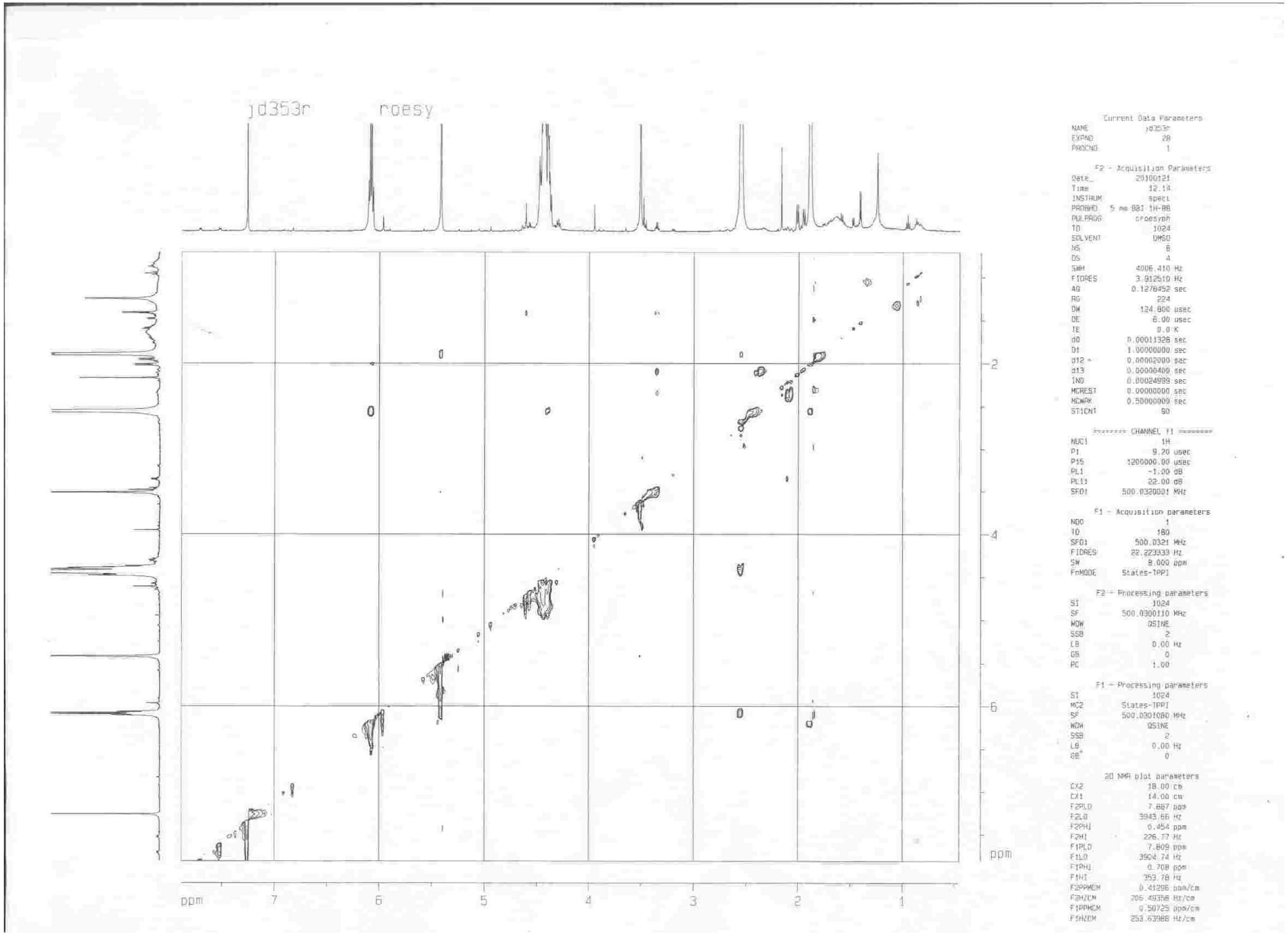

The ESIMS spectrum of compound **9**

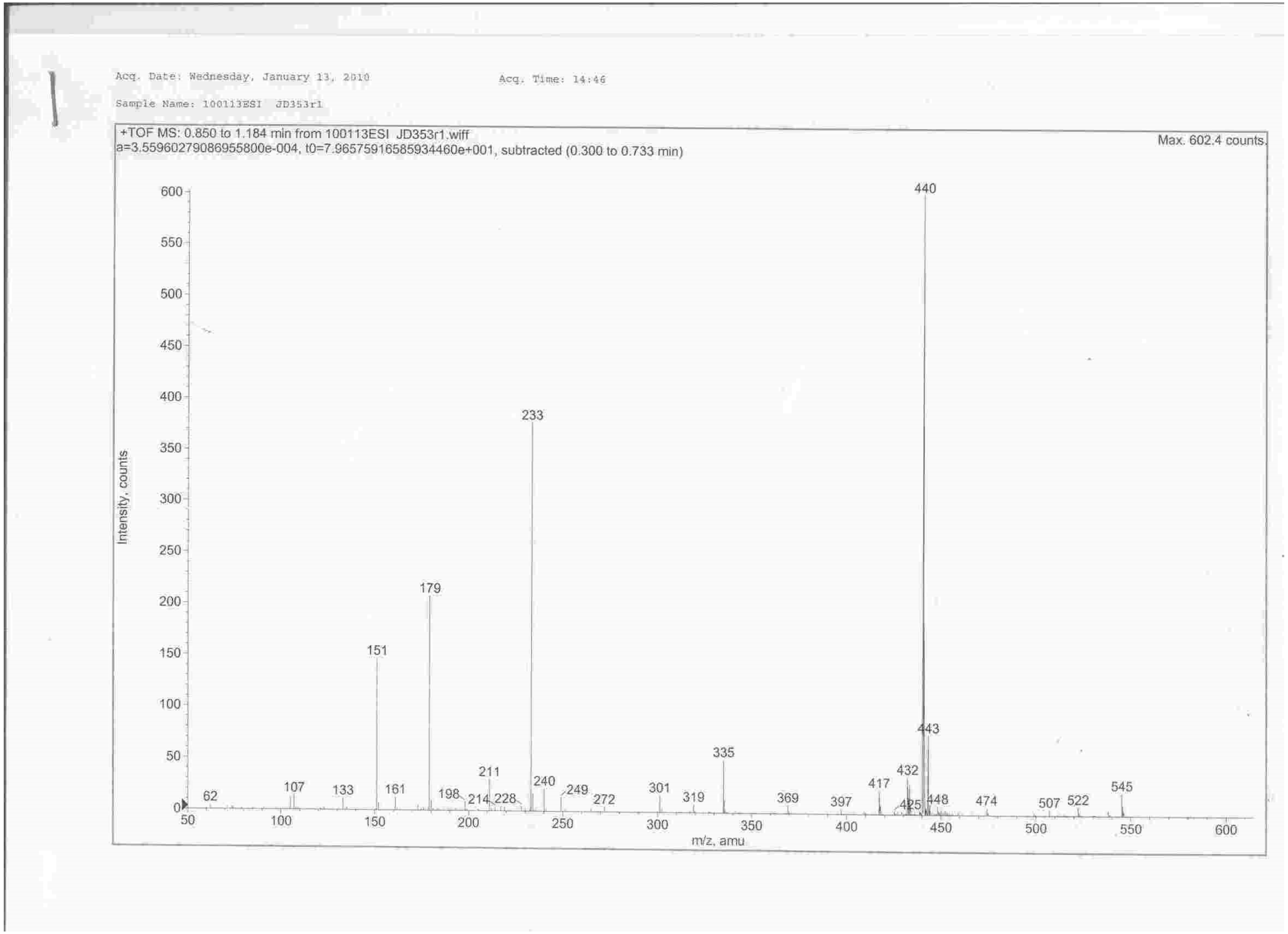

The HRESIMS spectrum of compound **9**

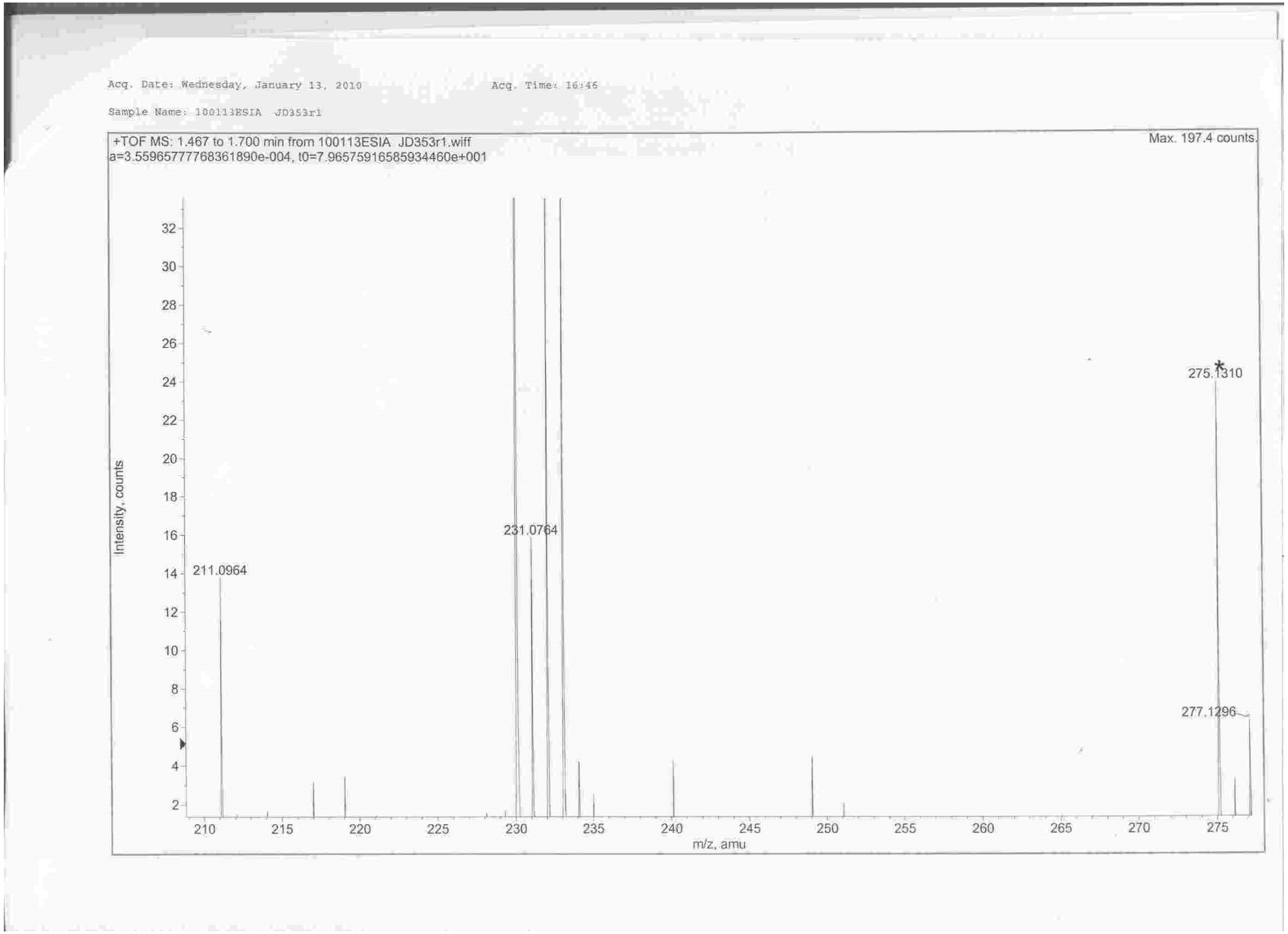

The IR spectrum of compound **9**

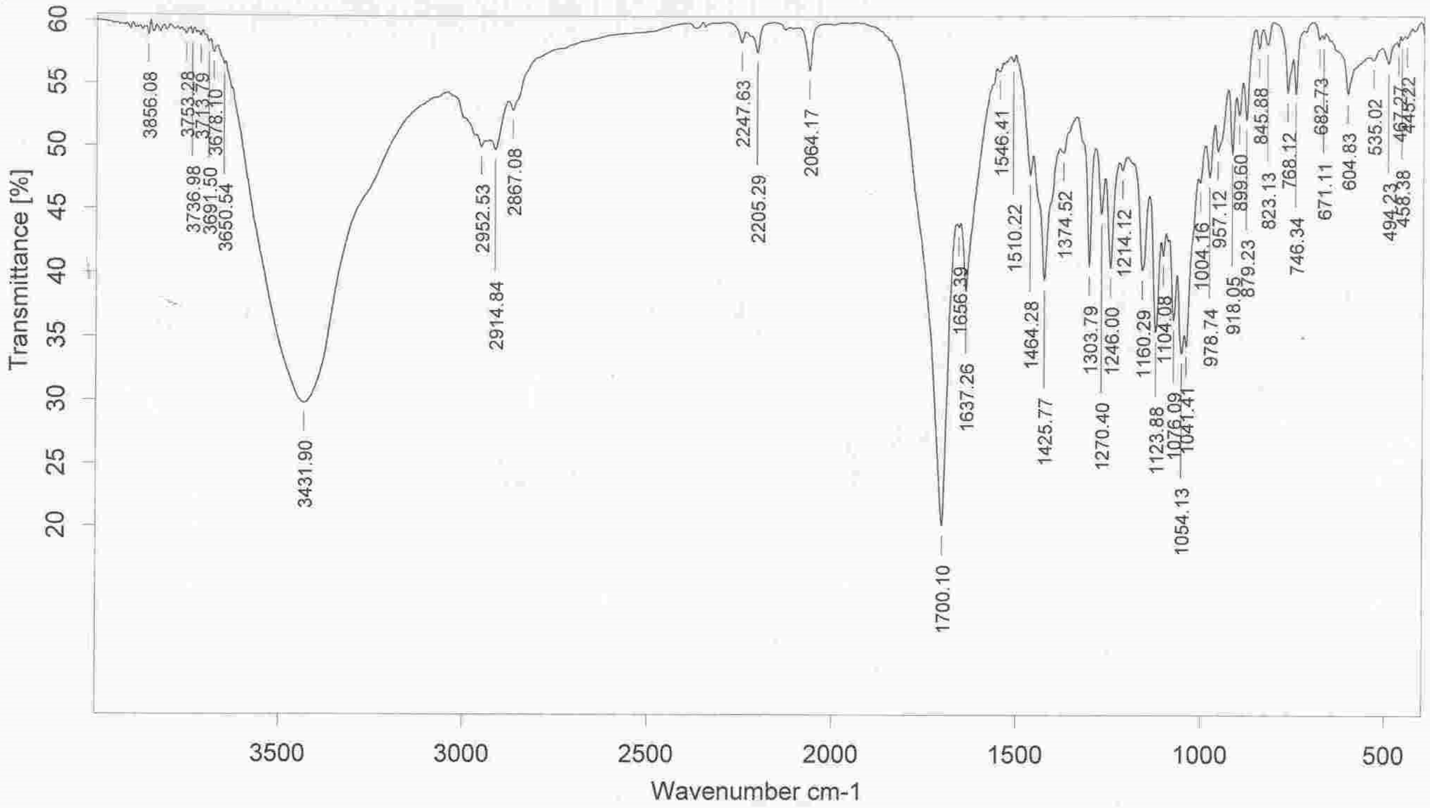

Sample : JD3531	Frequency Range : 399.271 - 3996.57		Measured on : 27/01/2010
Technique : KBr压片	Resolution : 4	Instrument : Tensor27	Sample Scans : 16
Customer : 100127IR1	Zerofilling : 2	Acquisition : Double Sided,For	

The UV spectrum of compound **9**

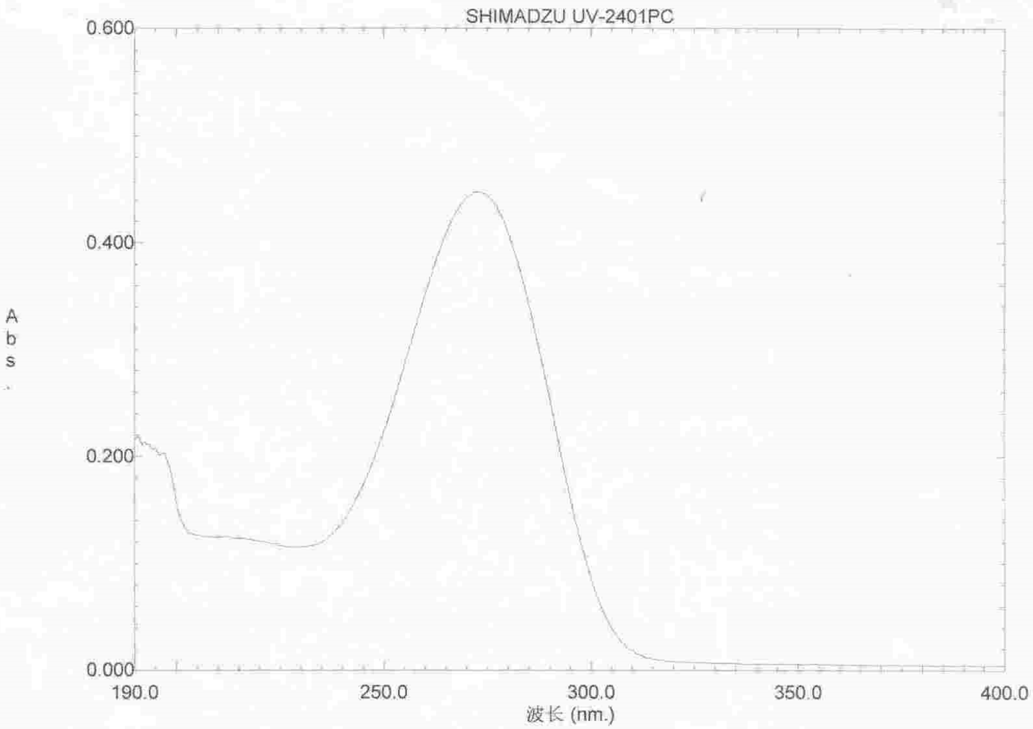

文件名: JD3531

JD3531 ———

样品浓度：0.0058毫克/毫升
溶剂：甲醇

创建于: 09:08 10-01-26
数据: 原始

测量模式: Abs.
扫描速度: 中速
狭缝: 2.0
采样间隔: 0.2

否.	波长 (nm.)	Abs.
1	272.60	0.4472
2	212.20	0.1249
3	191.00	0.2188

The $[\alpha]_D$ spectrum of compound **9**

Optical rotation measurement

Model : P-1020 (A060460638)

No	Sample	Mode	Data	Monitor<br>Blank	Temp.<br>Cell<br>Temp Point	Date<br>Comment<br>Sample Name	Light<br>Filter<br>Operator	Cycle Time<br>Integ Time
No.1	4 (1/3)	Sp.Rot	-0.4080	-0.0003<br>0.0000	15.8<br>50.00<br>Cell	Tue Jan 26 14:58:23 2010<br>0.00147g/mlMeOH<br>JD3531	Na<br>589nm	2 sec<br>10 sec
No.2	4 (2/3)	Sp.Rot	-0.4080	-0.0003<br>0.0000	15.9<br>50.00<br>Cell	Tue Jan 26 14:58:36 2010<br>0.00147g/mlMeOH<br>JD3531	Na<br>589nm	2 sec<br>10 sec
No.3	4 (3/3)	Sp.Rot	-6.3950	-0.0047<br>0.0000	15.9<br>50.00<br>Cell	Tue Jan 26 14:58:50 2010<br>0.00147g/mlMeOH<br>JD3531	Na<br>589nm	2 sec<br>10 sec

-2.4036°

The ^{1}H NMR spectrum of compound **10** in $CDCl_3$

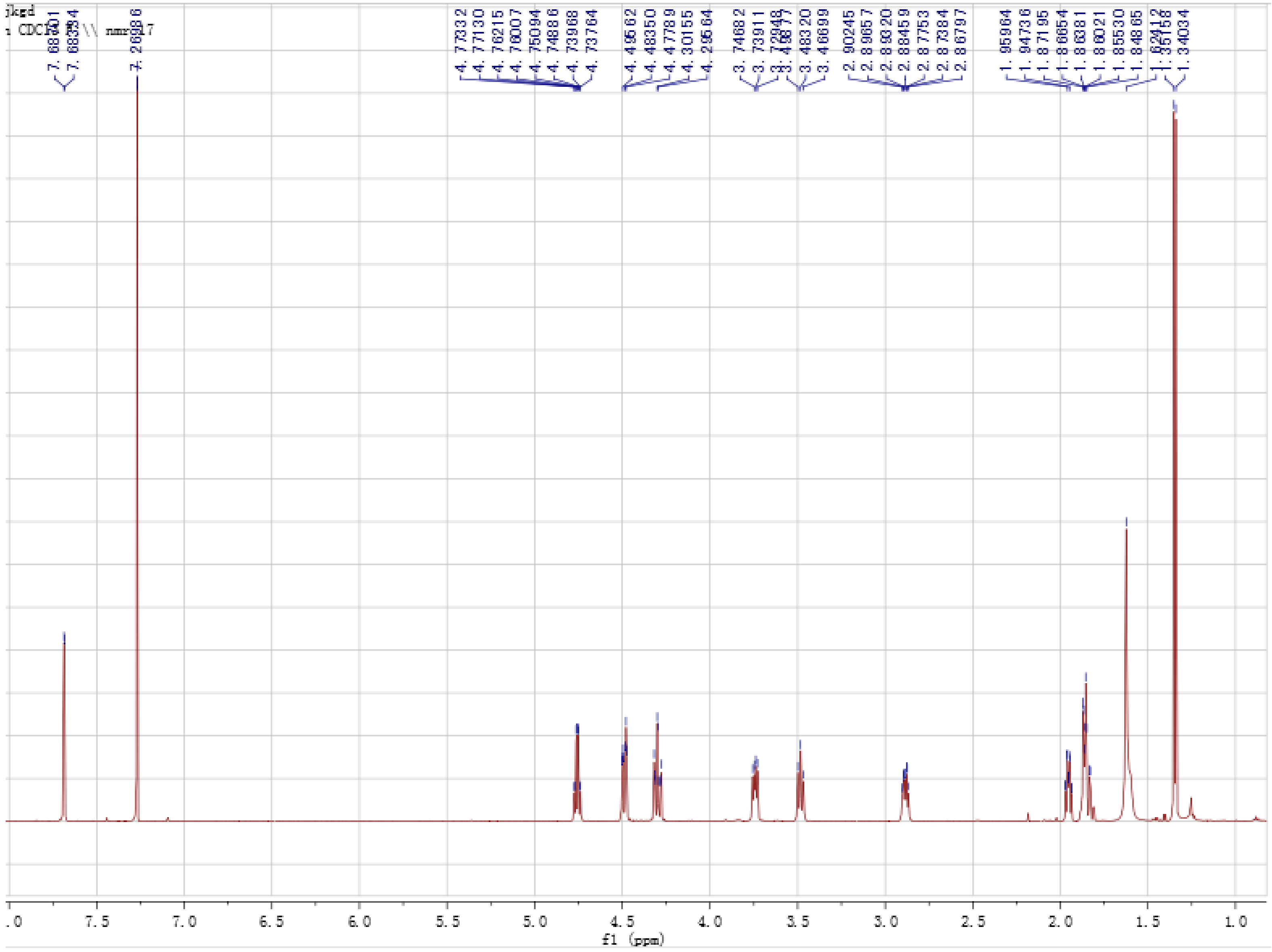

The ^{13}C NMR spectrum of compound **10** in $CDCl_3$

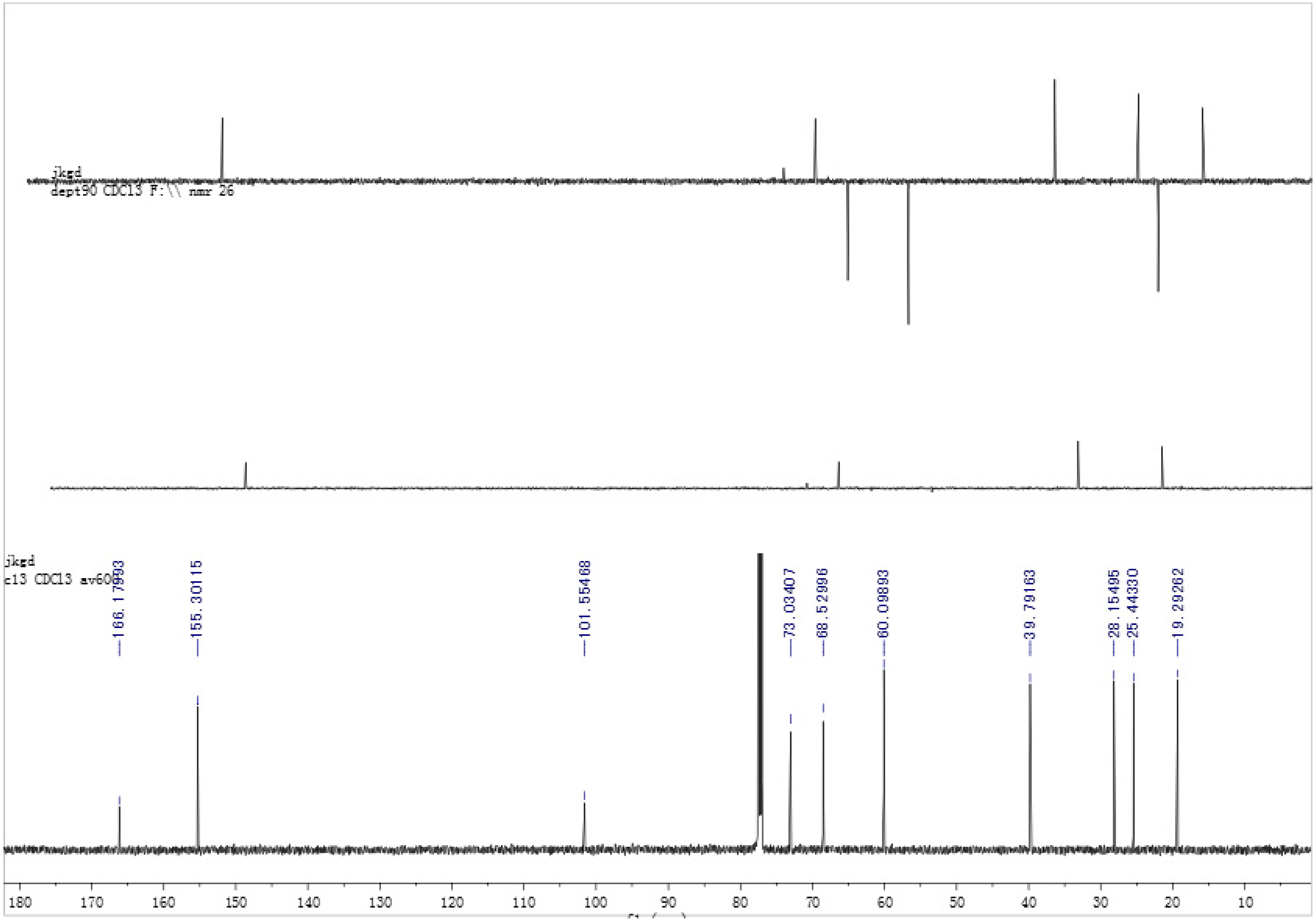

The HSQC spectrum of compound **10** in $CDCl_3$

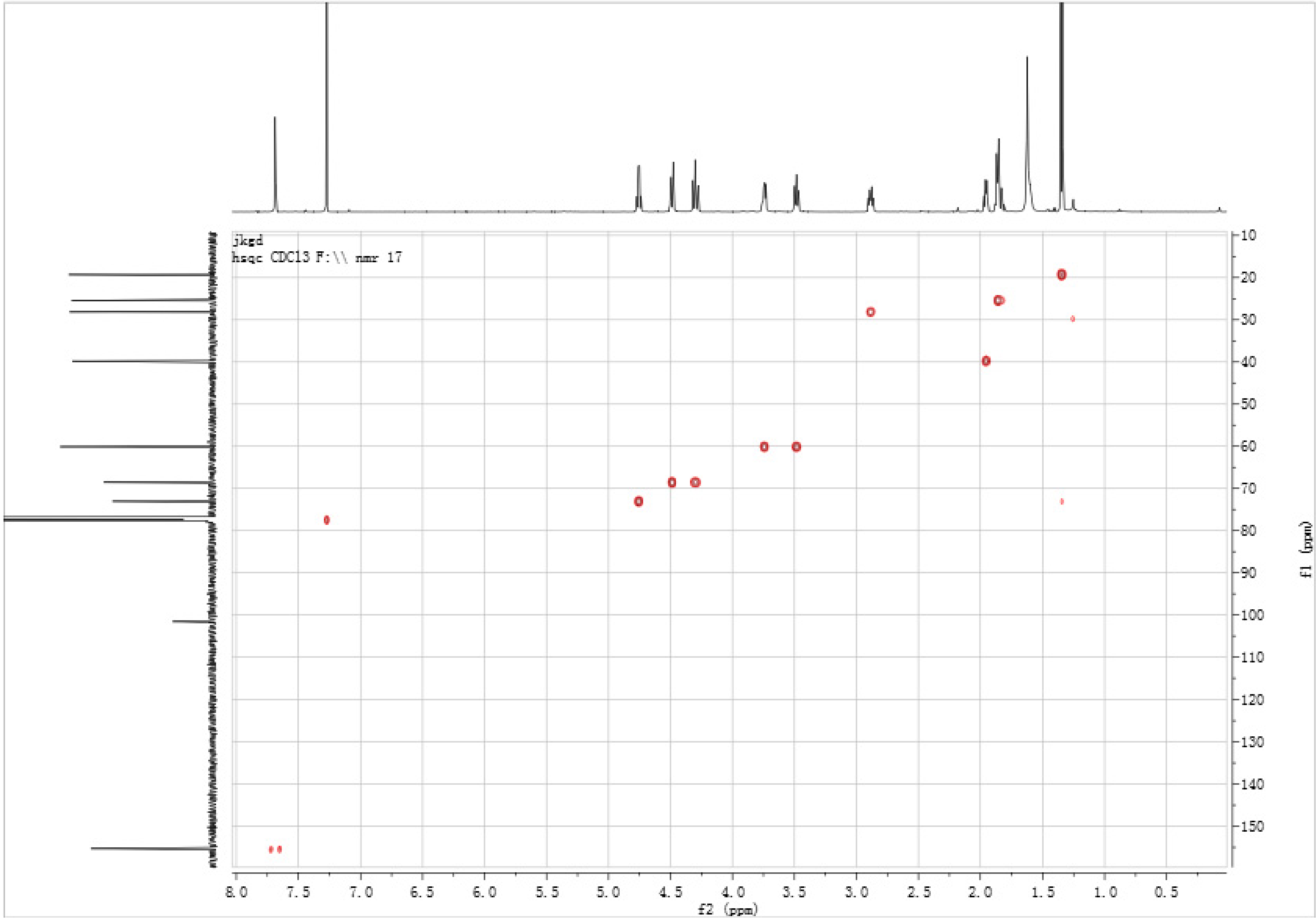

The HMBC spectrum of compound **10** in $CDCl_3$

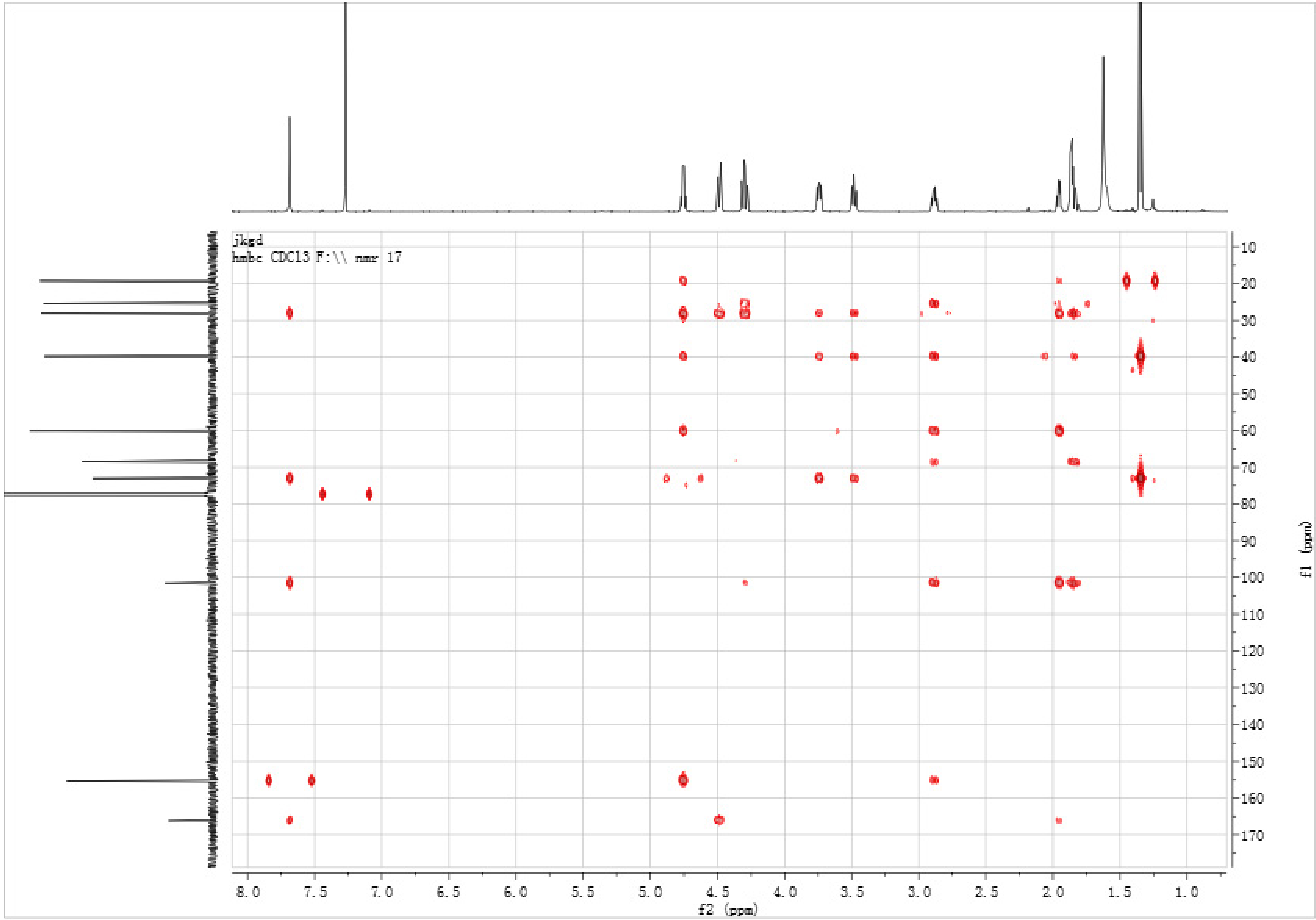

The ^{1}H ^{1}H COSY spectrum of compound **10** in $CDCl_3$

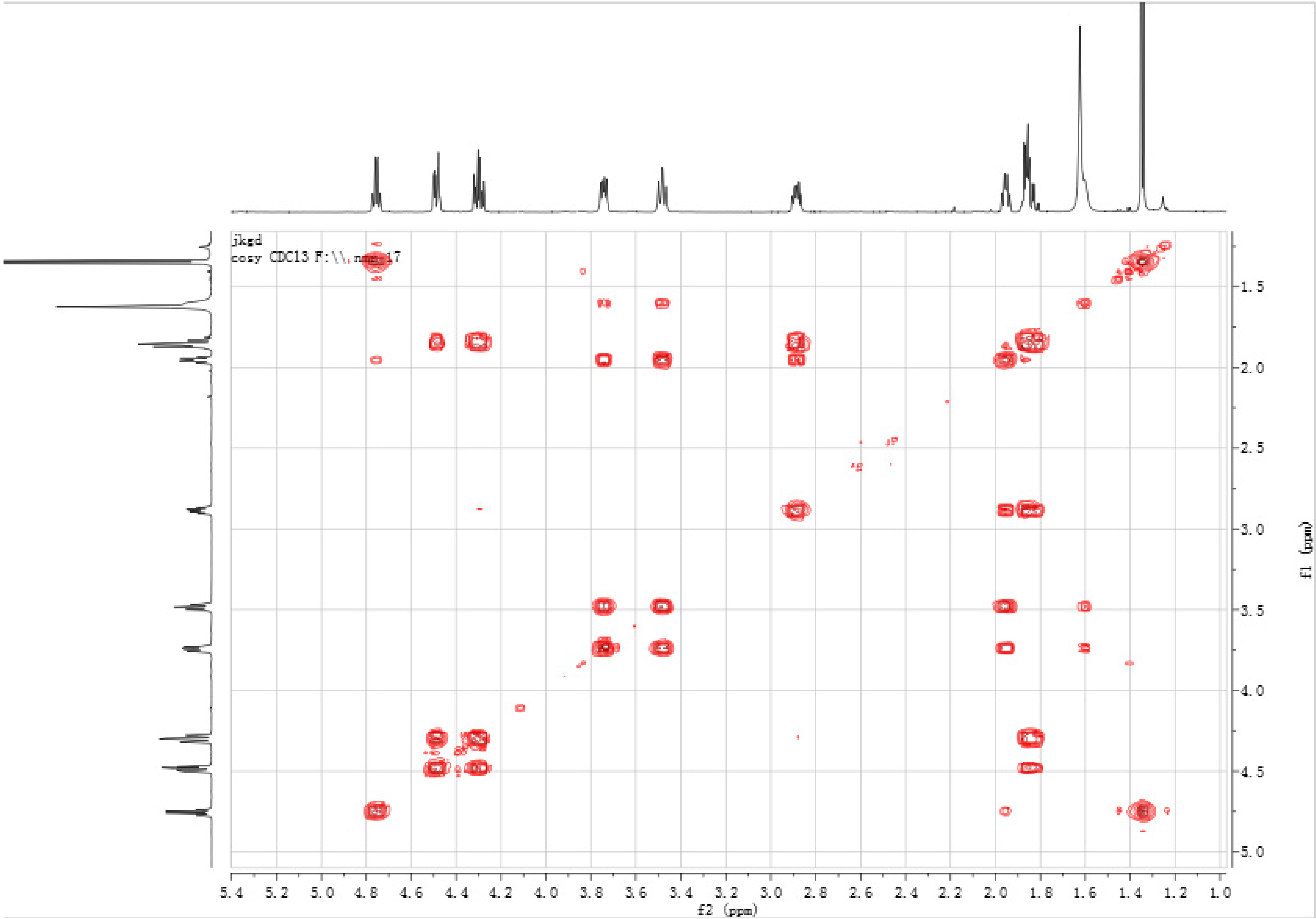

The ROESY spectrum of compound **10** in $CDCl_3$

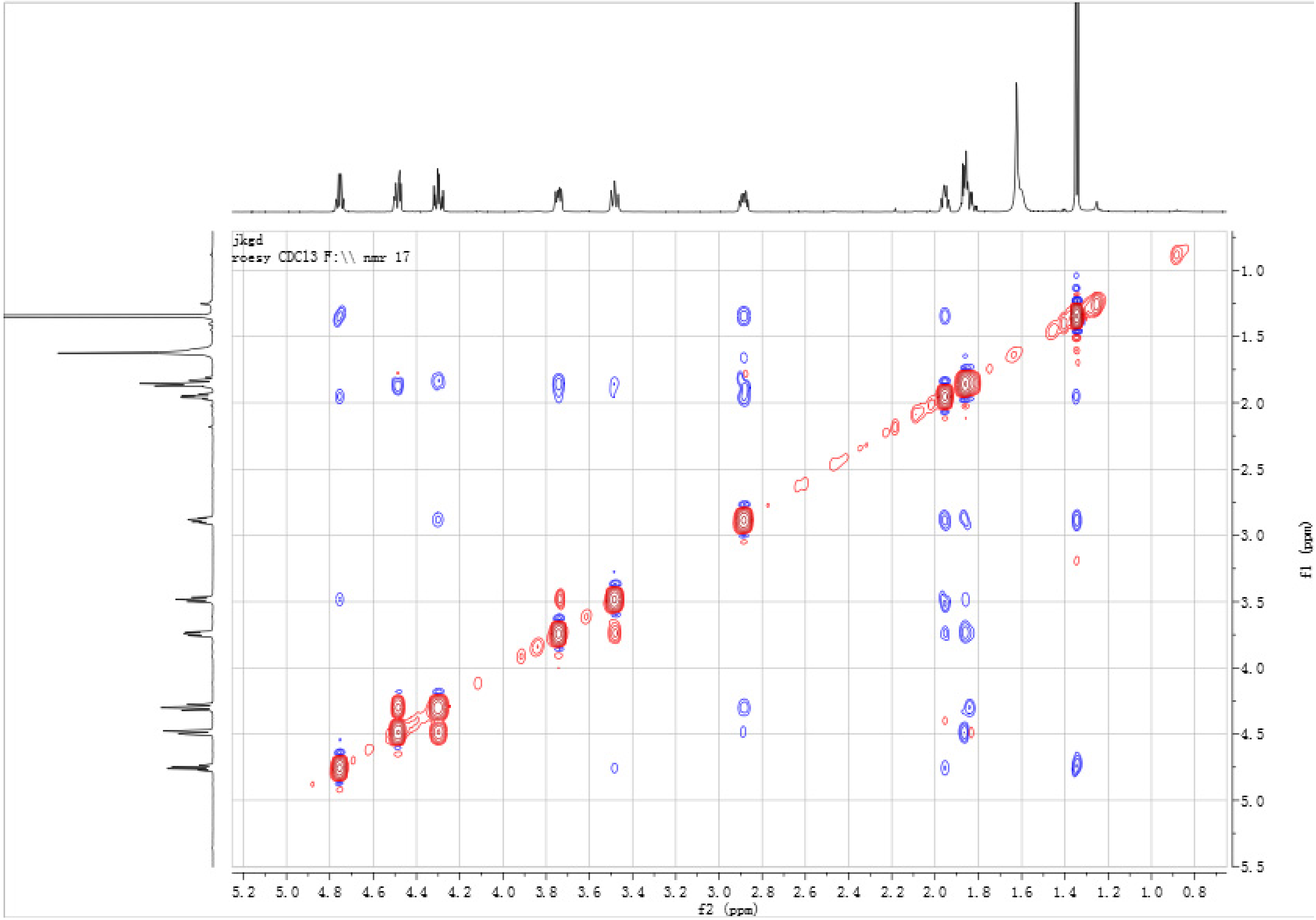

The EIMS spectrum of compound **10**

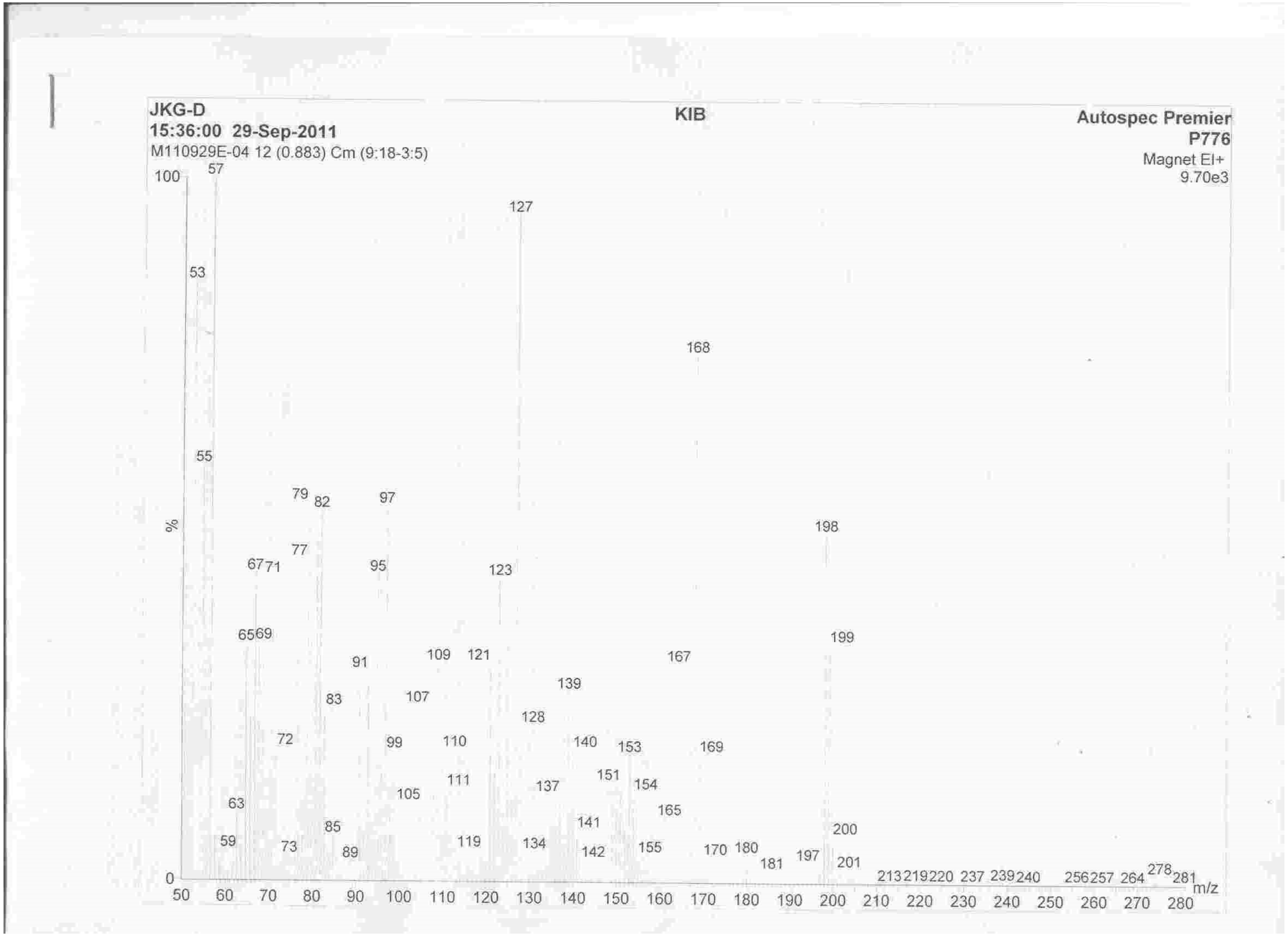

The HREIMS spectrum of compound **10**

Elemental Composition Report

Single Mass Analysis (displaying only valid results)
Tolerance = 10.0 mDa / DBE: min = 0.5, max = 40.0
Selected filters: None

Monoisotopic Mass, Odd and Even Electron Ions
13 formula(e) evaluated with 1 results within limits (up to 51 closest results for each mass)
Elements Used:
C: 0-200 H: 0-400 O: 0-4

JKG-D KIB Autospec Premier
15:24:04 29-Sep-2011 M110929EA-02AFAMM 65 (5.973) P776
Voltage EI+ 517

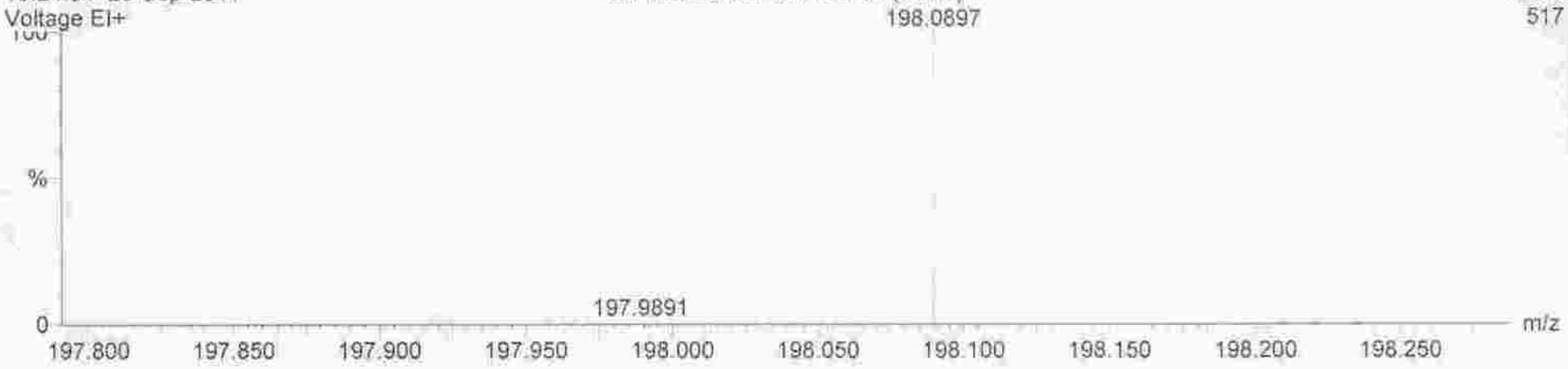

Minimum:				0.5		
Maximum:		10.0	10.0	40.0		
Mass	Calc. Mass	mDa	PPM	DBE	i-FIT	Formula
198.0897	198.0892	0.5	2.5	4.0	5546243.0	C10 H14 O4

The IR spectrum of compound **10**

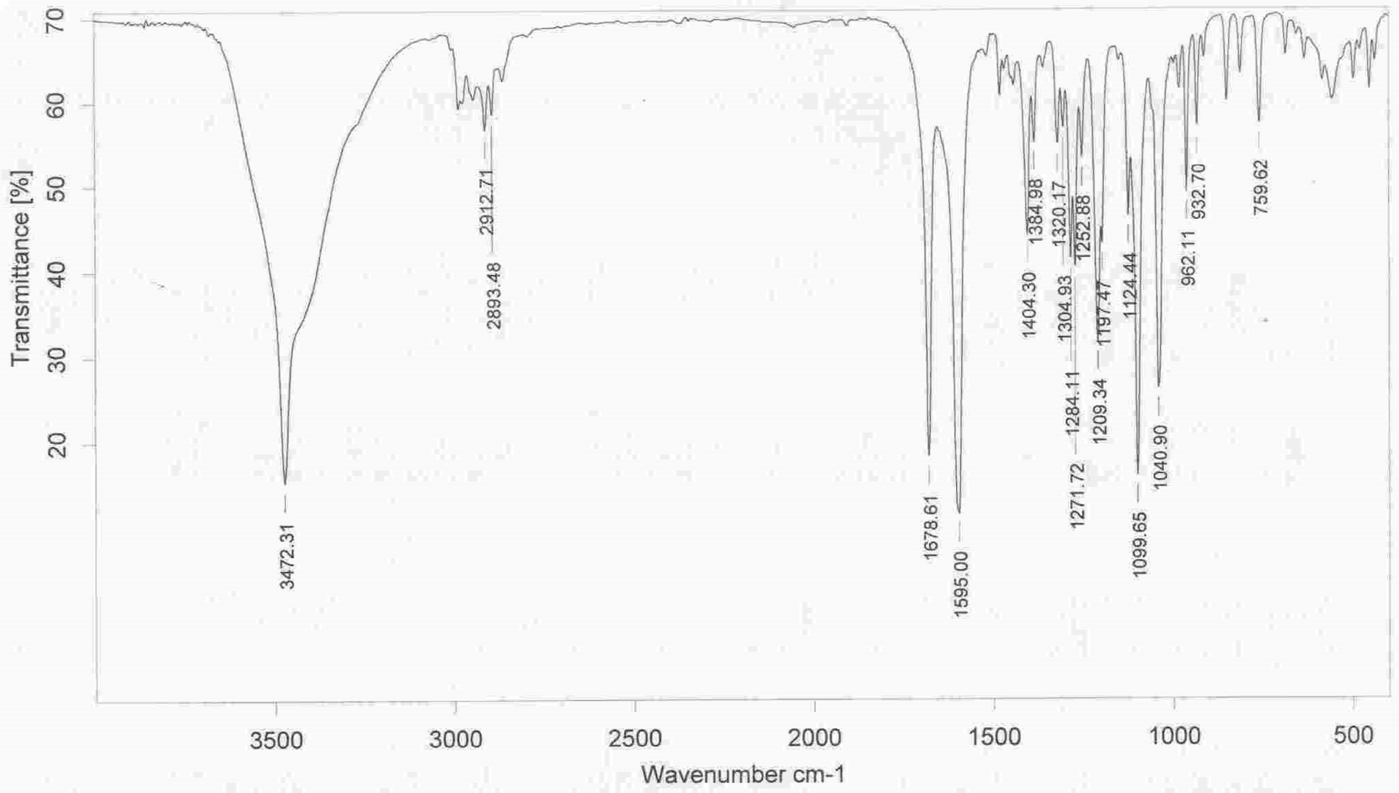

Sample : JKGD	Frequency Range : 399.246 - 3996.32		Measured on : 12/03/2012
Technique : KBr压片	Resolution : 4	Instrument : Tensor27	Sample Scans : 16
Customer : 120312IR14	Zerofilling : 2	Acquisition : Double Sided,For	

The UV spectrum of compound **10**

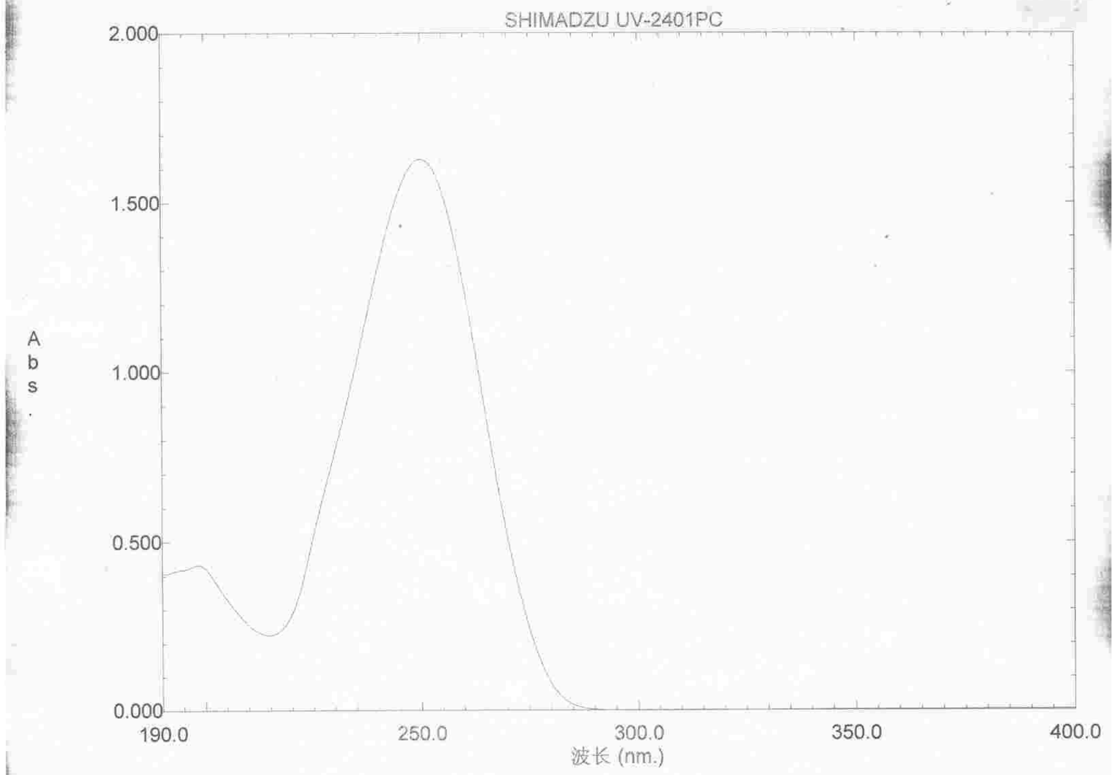

文件名: JKG-D

JKG-D ———

样品浓度：0.0336毫克/毫升
溶剂：甲醇

创建于: 20:26 11-07-25
数据: 原始

测量模式: Abs.
扫描速度: 中速
狭缝: 5.0
采样间隔: 0.2

否.	波长 (nm.)	Abs.
1	250.20	1.6277
2	198.20	0.4312

The $[\alpha]_D$ spectrum of compound **10**

Optical rotation measurement

Model : P-1020 (A060460638)

No.	Sample	Mode	Data	Monitor<br>Blank	Temp.<br>Cell<br>Temp Point	Date<br>Comment<br>Sample Name	Light<br>Filter<br>Operator	Cycle Time<br>Integ Time
No.1	3 (1/3)	Sp.Rot	-89.8570	-0.0629<br>0.0000	13.2<br>50.00<br>Cell	Wed Nov 09 10:22:19 2011<br>0.00140g/mlMeOH<br>JKG-D	Na<br>589nm	2 sec<br>10 sec
No.2	3 (2/3)	Sp.Rot	-90.8570	-0.0636<br>0.0000	13.2<br>50.00<br>Cell	Wed Nov 09 10:22:32 2011<br>0.00140g/mlMeOH<br>JKG-D	Na<br>589nm	2 sec<br>10 sec
No.3	3 (3/3)	Sp.Rot	-91.0000	-0.0637<br>0.0000	13.2<br>50.00<br>Cell	Wed Nov 09 10:22:46 2011<br>0.00140g/mlMeOH<br>JKG-D	Na<br>589nm	2 sec<br>10 sec

-90.5714

The ^{1}H NMR spectrum of compound **11** in $CDCl_3$

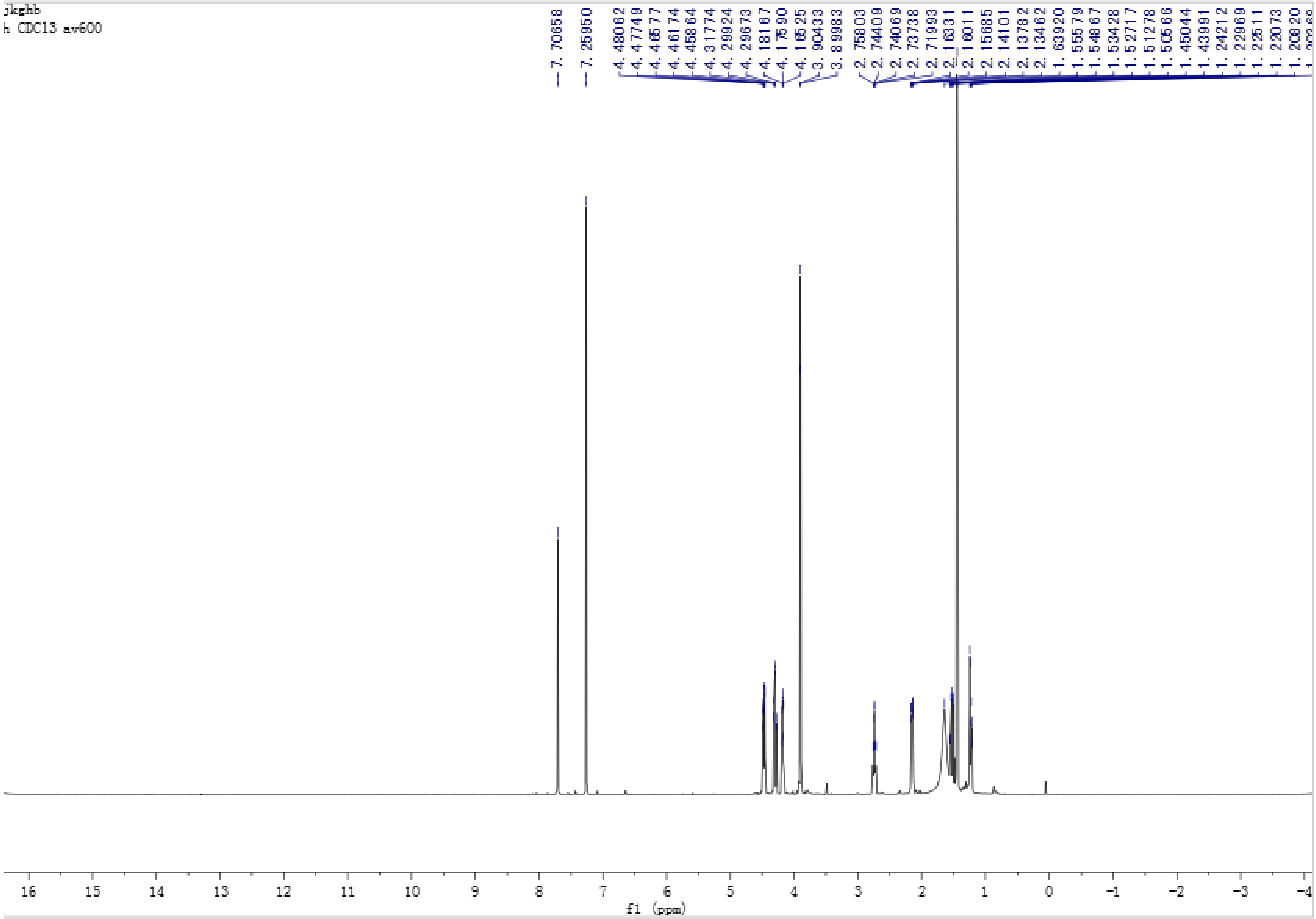

The ^{13}C NMR spectrum of compound **11** in $CDCl_3$

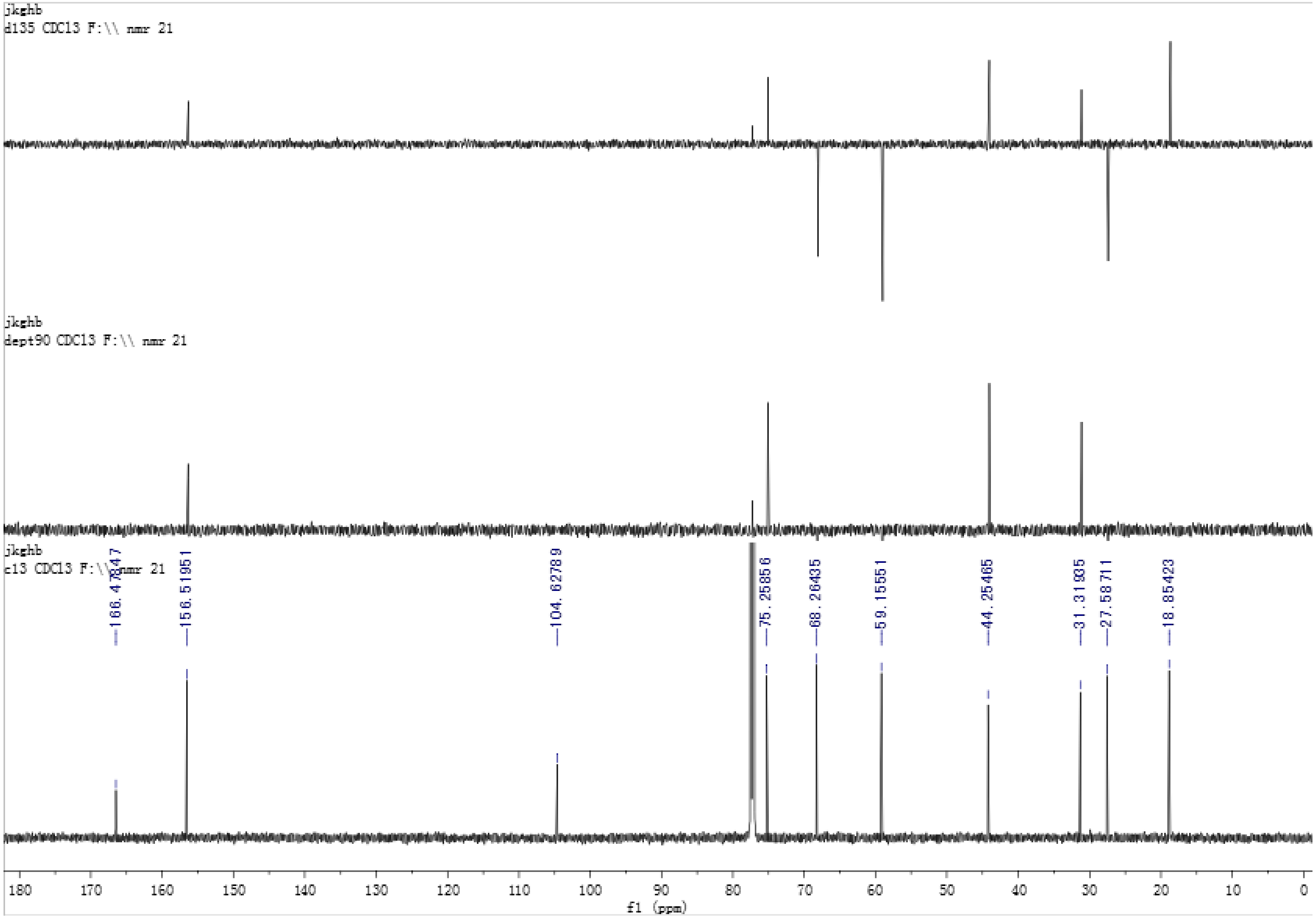

The HSQC spectrum of compound **11** in $CDCl_3$

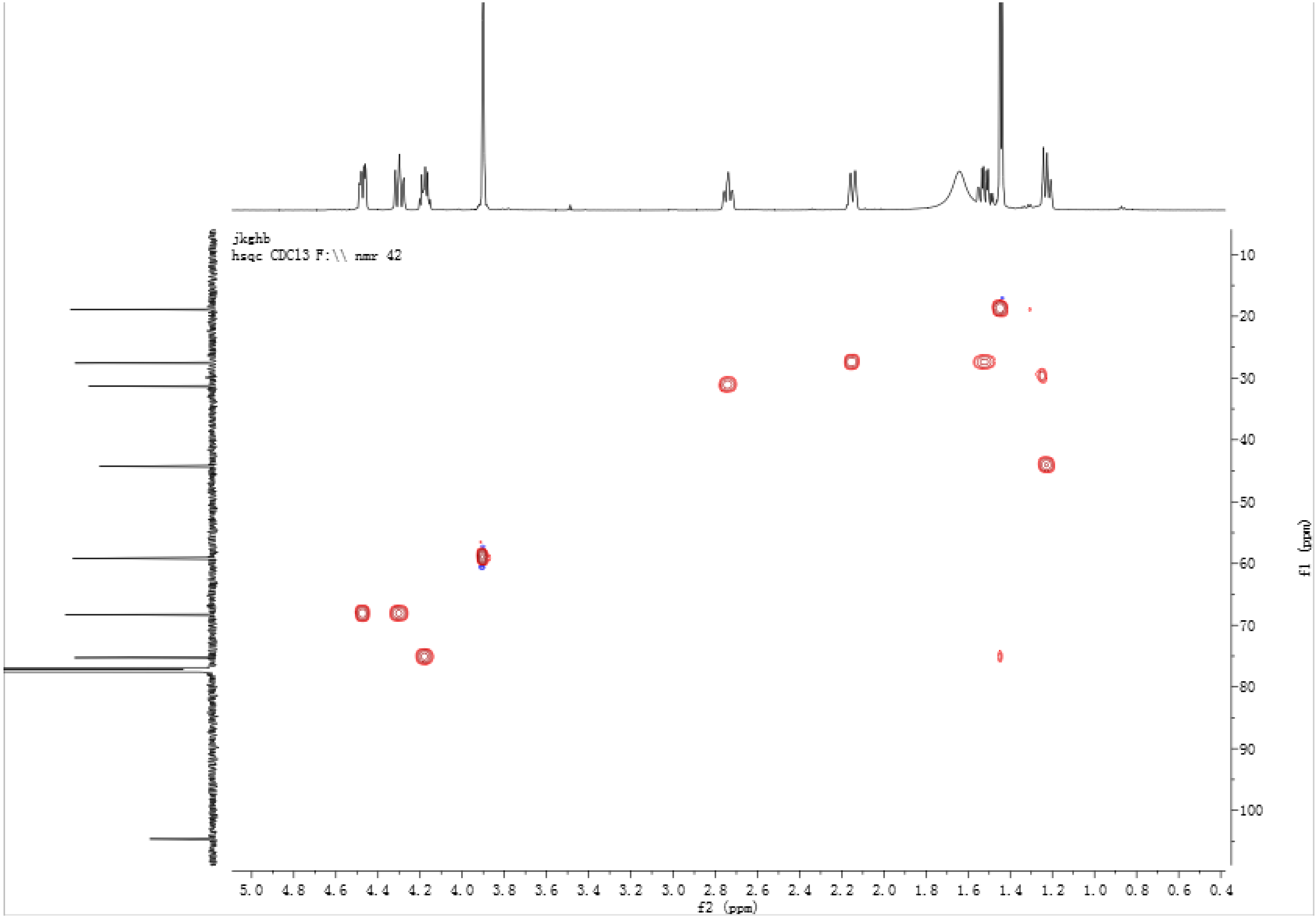

The HMBC spectrum of compound **11** in $CDCl_3$

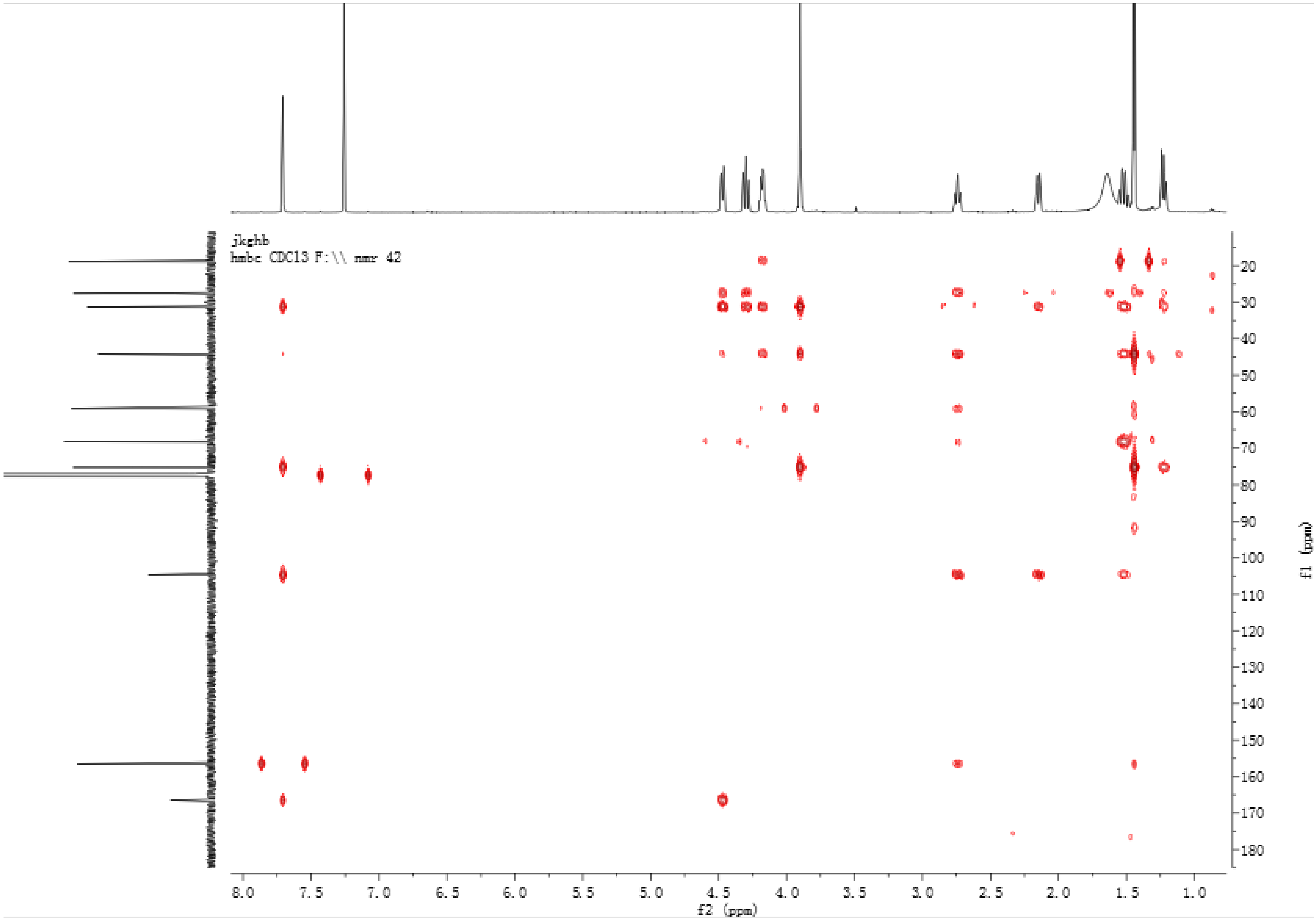

The ^1H ^1H COSY spectrum of compound **11** in $CDCl_3$

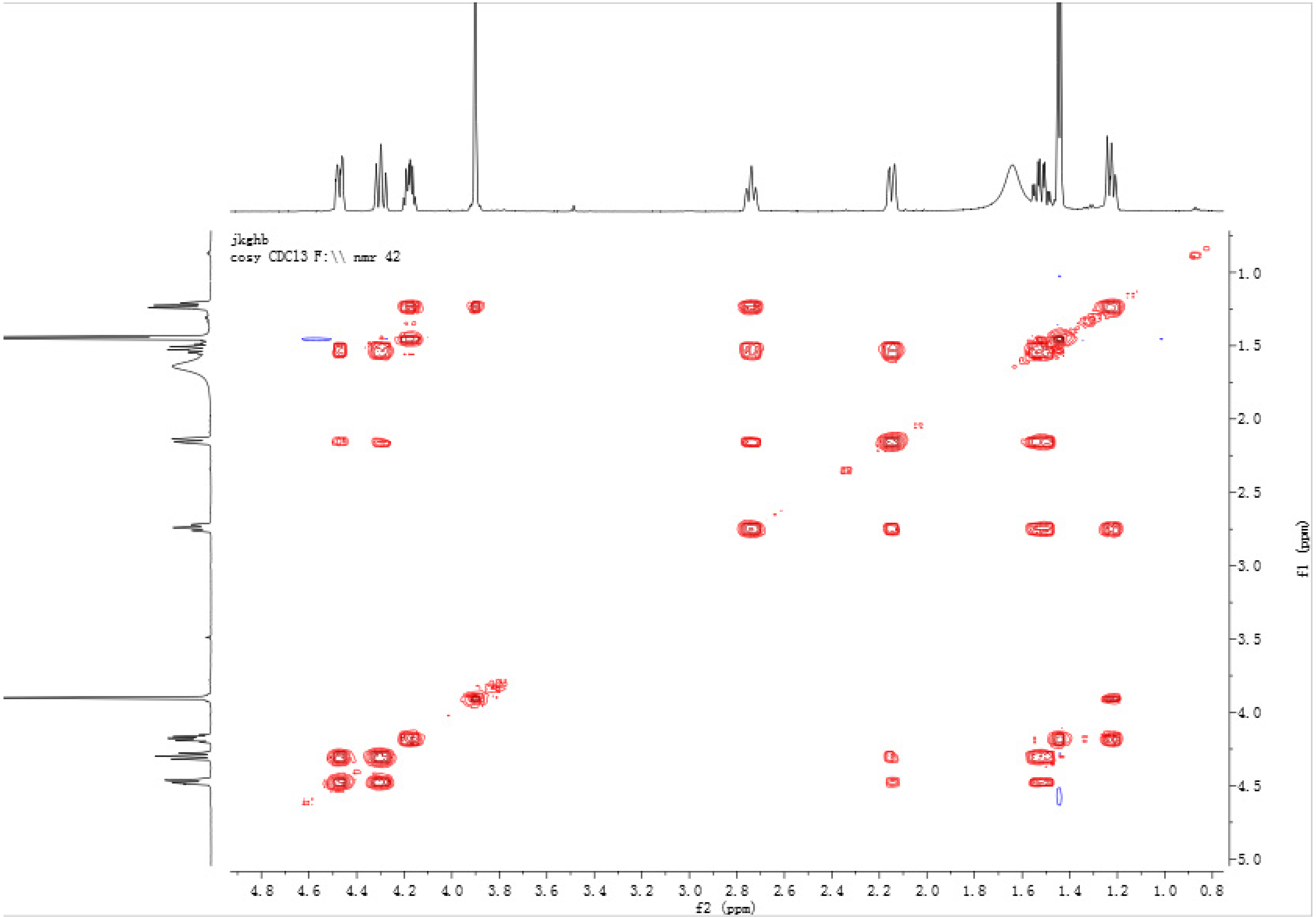

The ROESY spectrum of compound **11** in $CDCl_3$

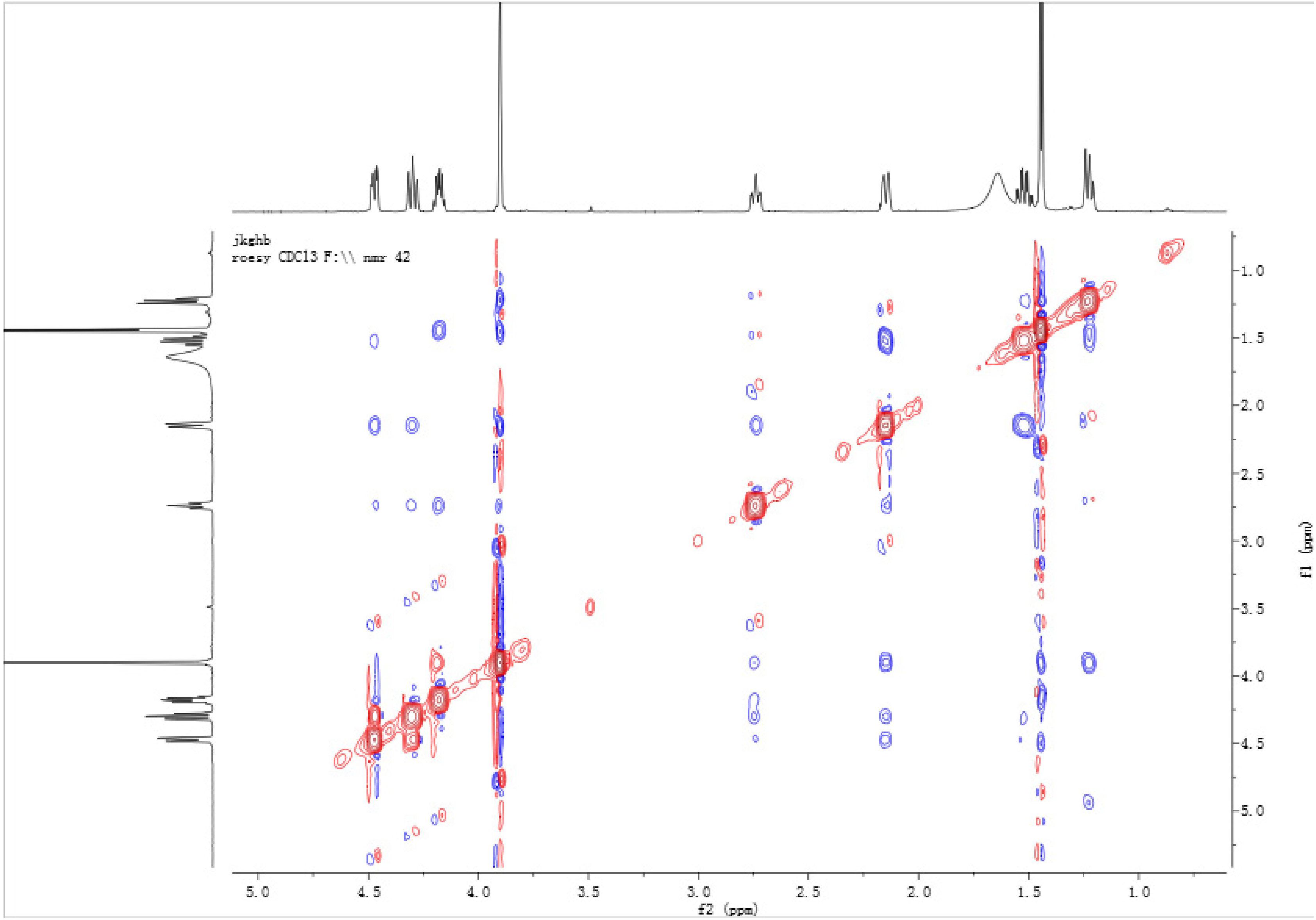

The EIMS spectrum of compound **11**

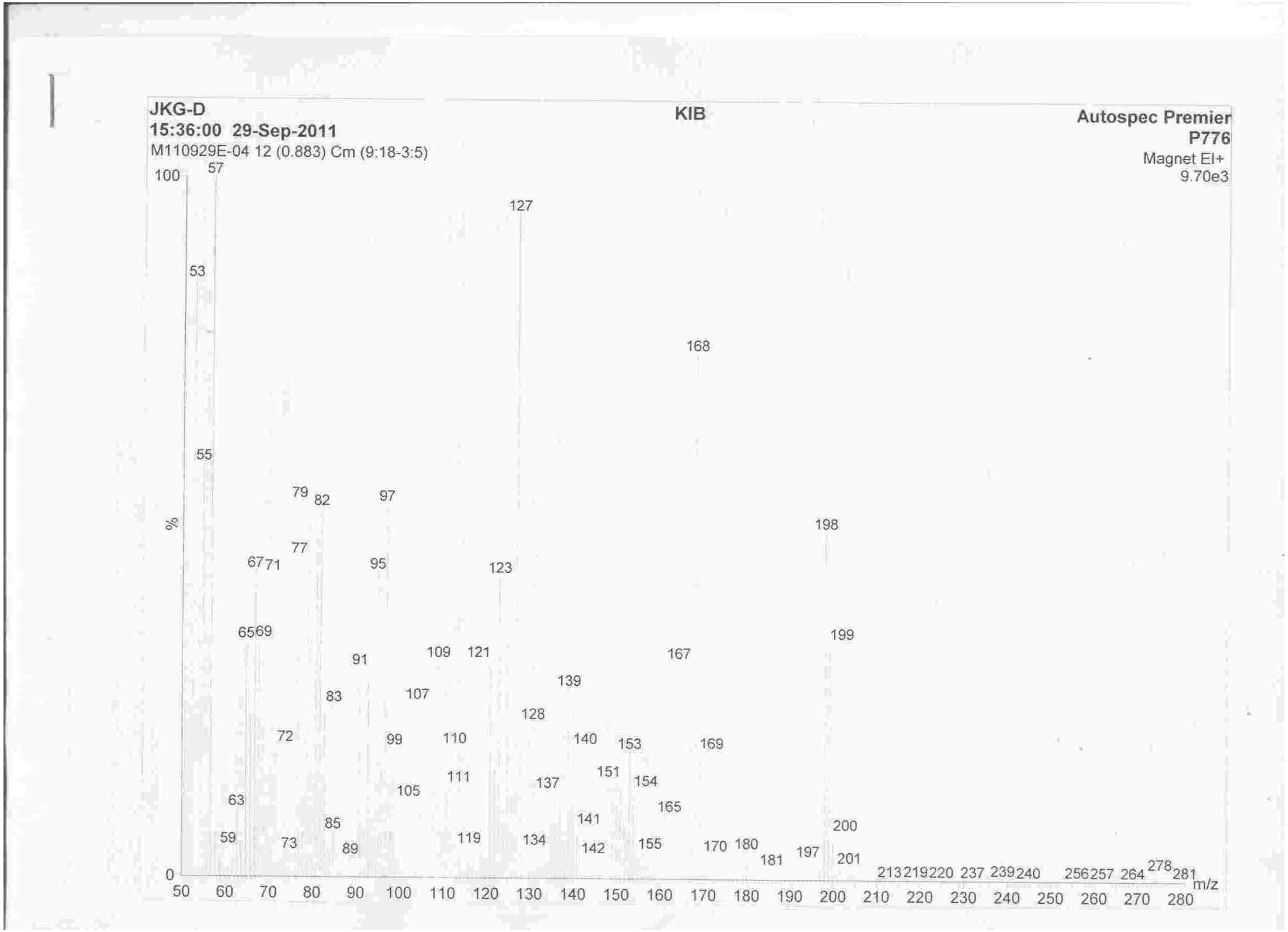

The HREIMS spectrum of compound **11**

Elemental Composition Report

Single Mass Analysis (displaying only valid results)
Tolerance = 10.0 mDa / DBE: min = 0.5, max = 40.0
Selected filters: None

Monoisotopic Mass, Odd and Even Electron Ions
13 formula(e) evaluated with 1 results within limits (up to 51 closest results for each mass)
Elements Used:
C: 0-200 H: 0-400 O: 0-4

JKG-D KIB Autospec Premier
15:24:04 29-Sep-2011 M110929EA-02AFAMM 65 (5.973) P776
Voltage EI+ 517

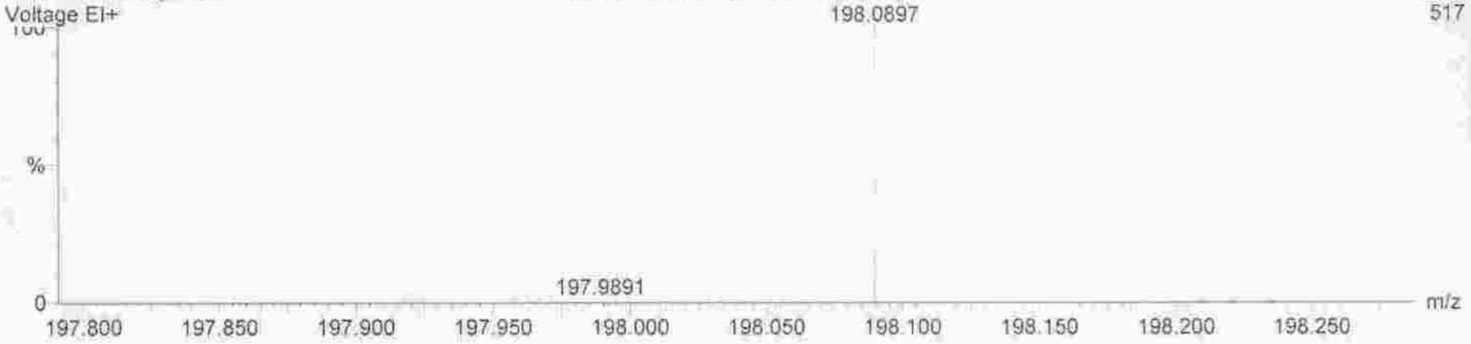

Minimum:				0.5		
Maximum:		10.0	10.0	40.0		
Mass	Calc. Mass	mDa	PPM	DBE	i-FIT	Formula
198.0897	198.0892	0.5	2.5	4.0	5546243.0	C10 H14 O4

The IR spectrum of compound **11**

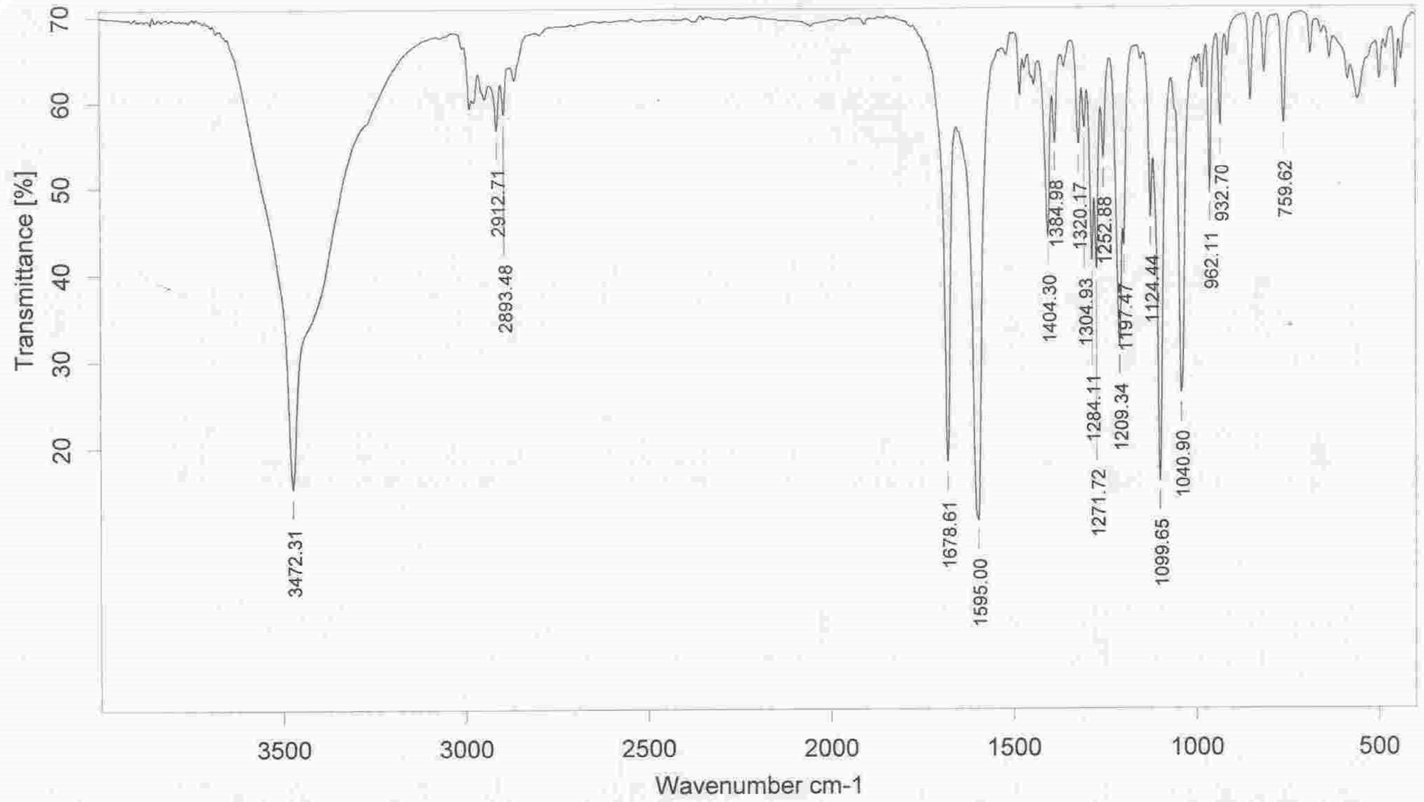

Sample : JKGD	Frequency Range : 399.246 - 3996.32		Measured on : 12/03/2012
Technique : KBr压片	Resolution : 4	Instrument : Tensor27	Sample Scans : 16
Customer : 120312IR14	Zerofilling : 2	Acquisition : Double Sided,For	

The UV spectrum of compound **11**

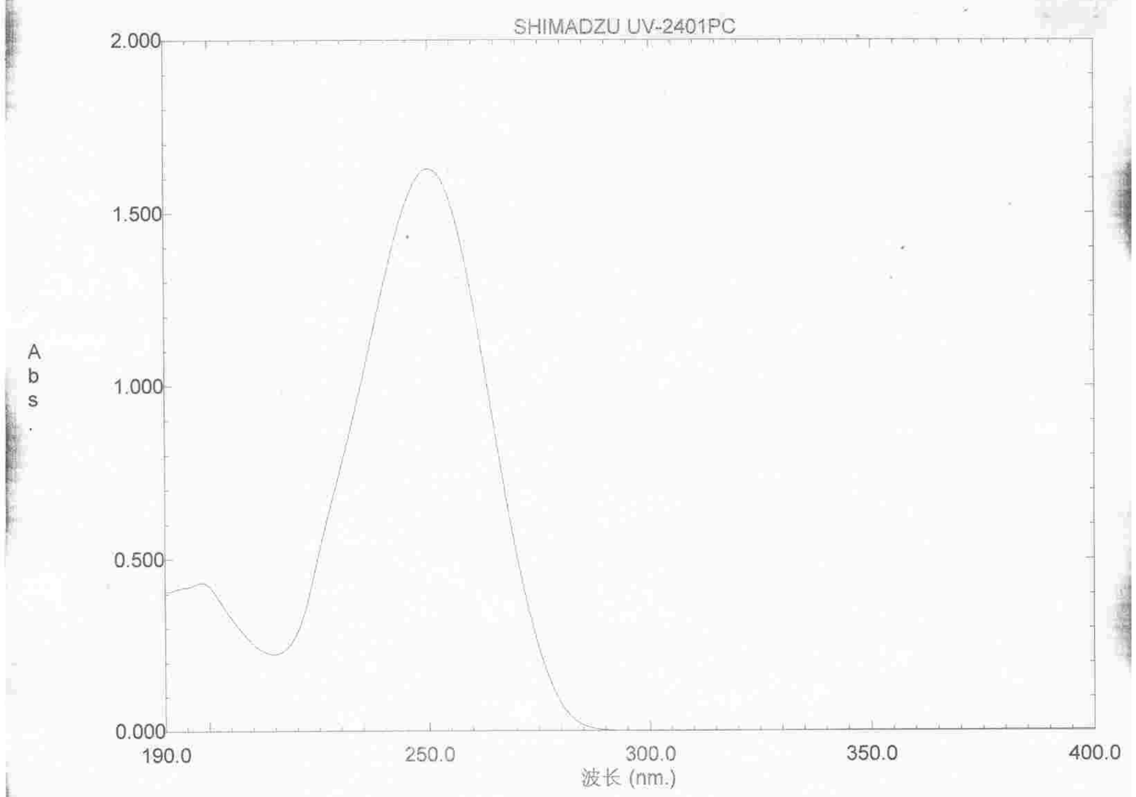

文件名: JKG-D

JKG-D ———

样品浓度：0.0336毫克/毫升
溶剂：甲醇

创建于: 20:26 11-07-25
数据: 原始

测量模式: Abs.
扫描速度: 中速
狭缝: 5.0
采样间隔: 0.2

否.	波长 (nm.)	Abs.
1	250.20	1.6277
2	198.20	0.4312

The $[\alpha]_D$ spectrum of compound **11**

Optical rotation measurement

Model : P-1020 (A060460638)

No.	Sample	Mode	Data	Monitor<br>Blank	Temp.<br>Cell<br>Temp Point	Date<br>Comment<br>Sample Name	Light<br>Filter<br>Operator	Cycle Time<br>Integ Time
No.1	3 (1/3)	Sp.Rot	-89.8570	-0.0629<br>0.0000	13.2<br>50.00<br>Cell	Wed Nov 09 10:22:19 2011<br>0.00140g/mlMeOH<br>JKG-D	Na<br>589nm	2 sec<br>10 sec
No.2	3 (2/3)	Sp.Rot	-90.8570	-0.0636<br>0.0000	13.2<br>50.00<br>Cell	Wed Nov 09 10:22:32 2011<br>0.00140g/mlMeOH<br>JKG-D	Na<br>589nm	2 sec<br>10 sec
No.3	3 (3/3)	Sp.Rot	-91.0000	-0.0637<br>0.0000	13.2<br>50.00<br>Cell	Wed Nov 09 10:22:46 2011<br>0.00140g/mlMeOH<br>JKG-D	Na<br>589nm	2 sec<br>10 sec

-90.[illegible]

The ^{1}H NMR spectrum of dihydroepinaucledal (**14**) in $CDCl_3$

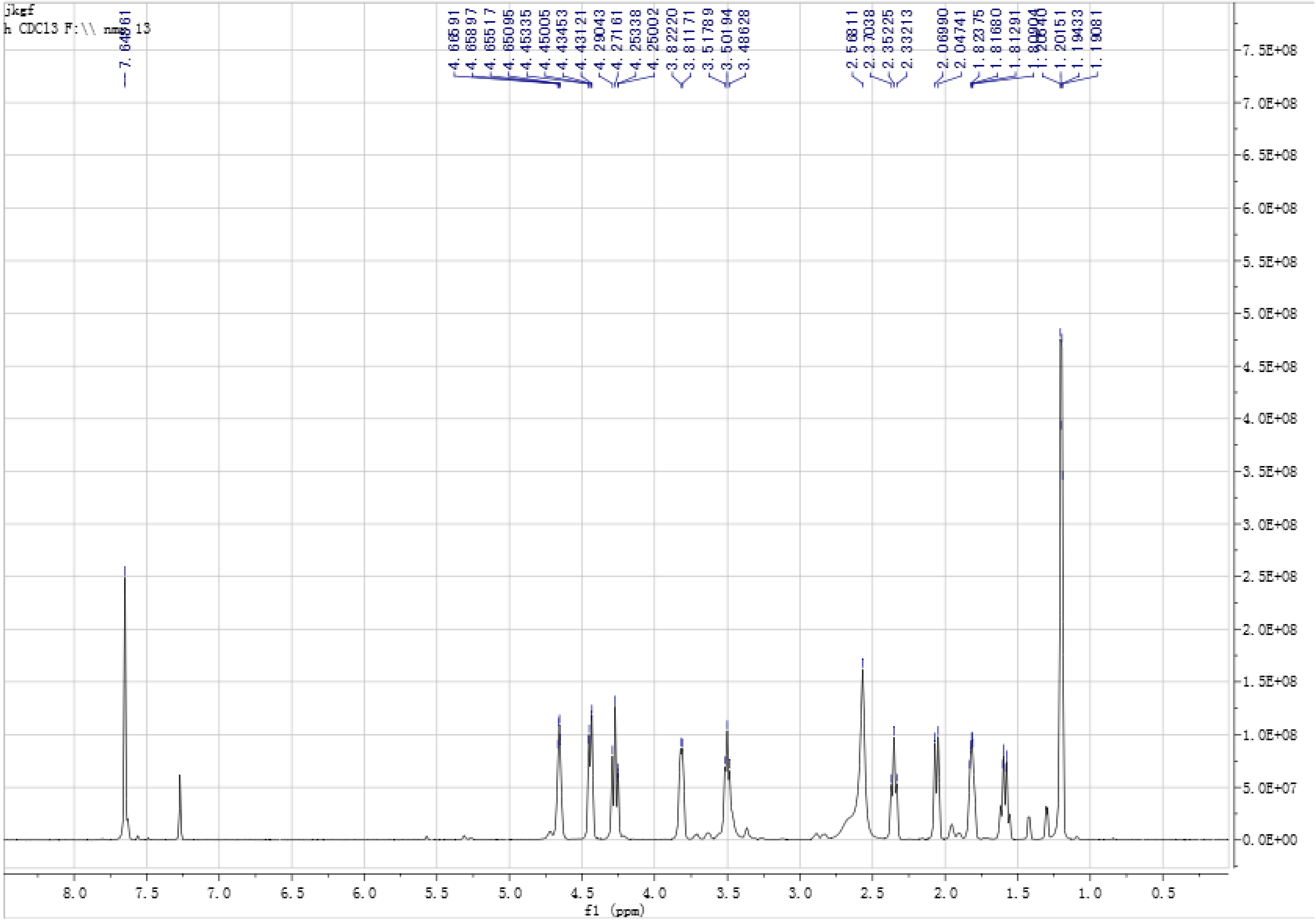

The ^{13}C NMR spectrum of dihydroepinaucledal (**14**) in $CDCl_3$

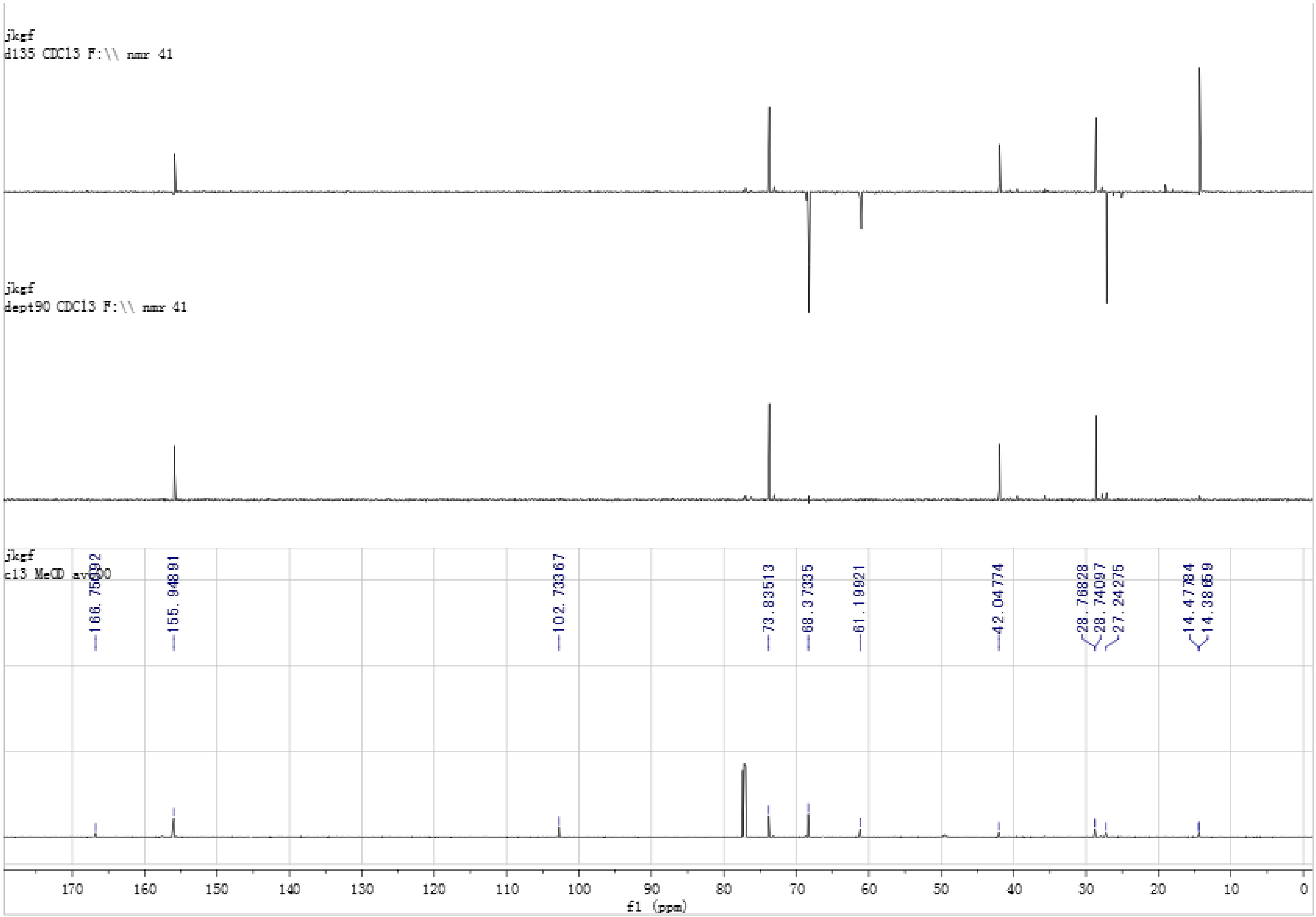

The HSQC spectrum of dihydroepinaucledal (**14**) in $CDCl_3$

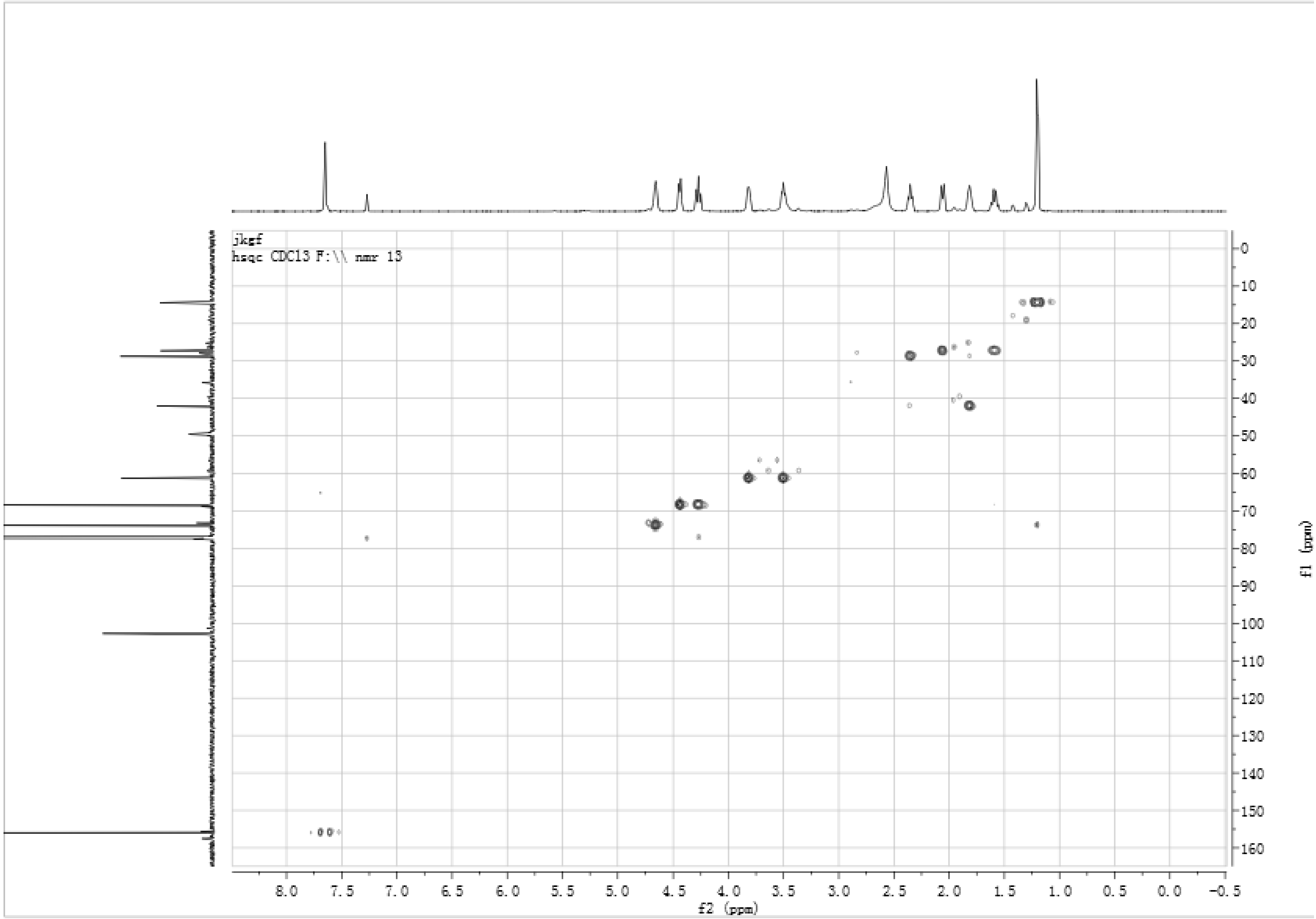

The HBMC spectrum of dihydroepinaucledal (**14**) in $CDCl_3$

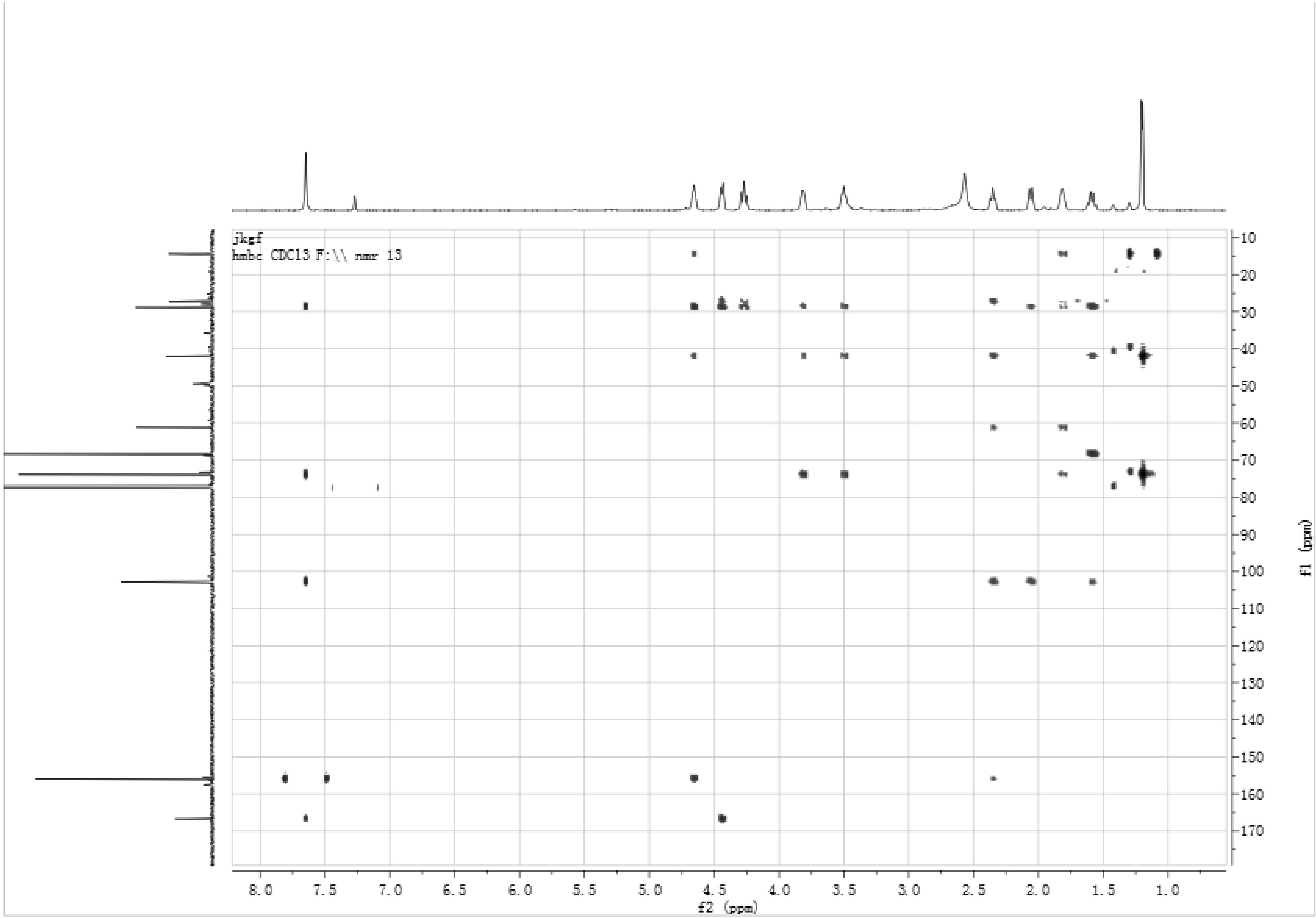

The ^{1}H ^{1}H COSY spectrum of dihydroepinaucledal (**14**) in $CDCl_3$

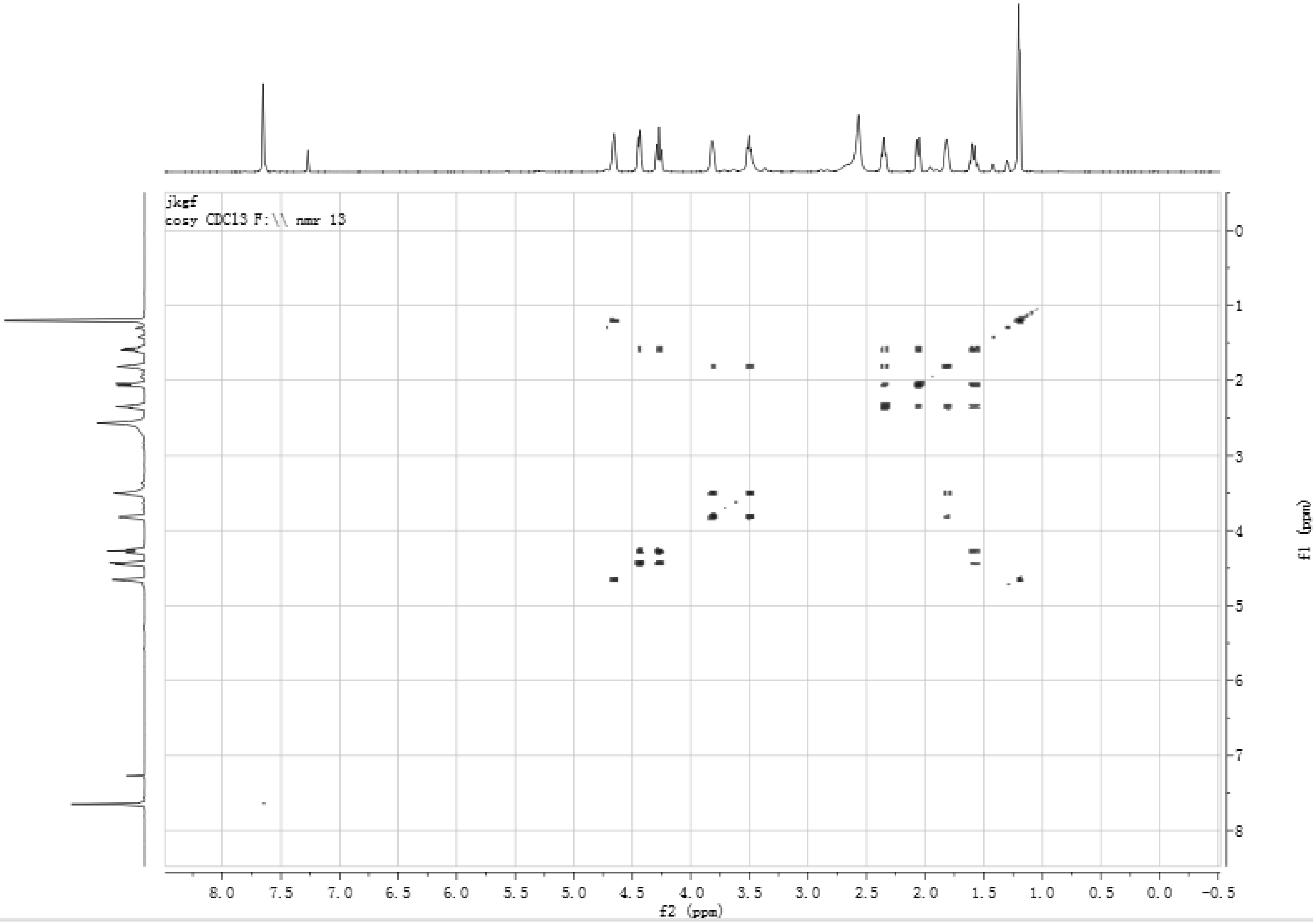

The ROESY spectrum of dihydroepinaucledal (**14**) in $CDCl_3$

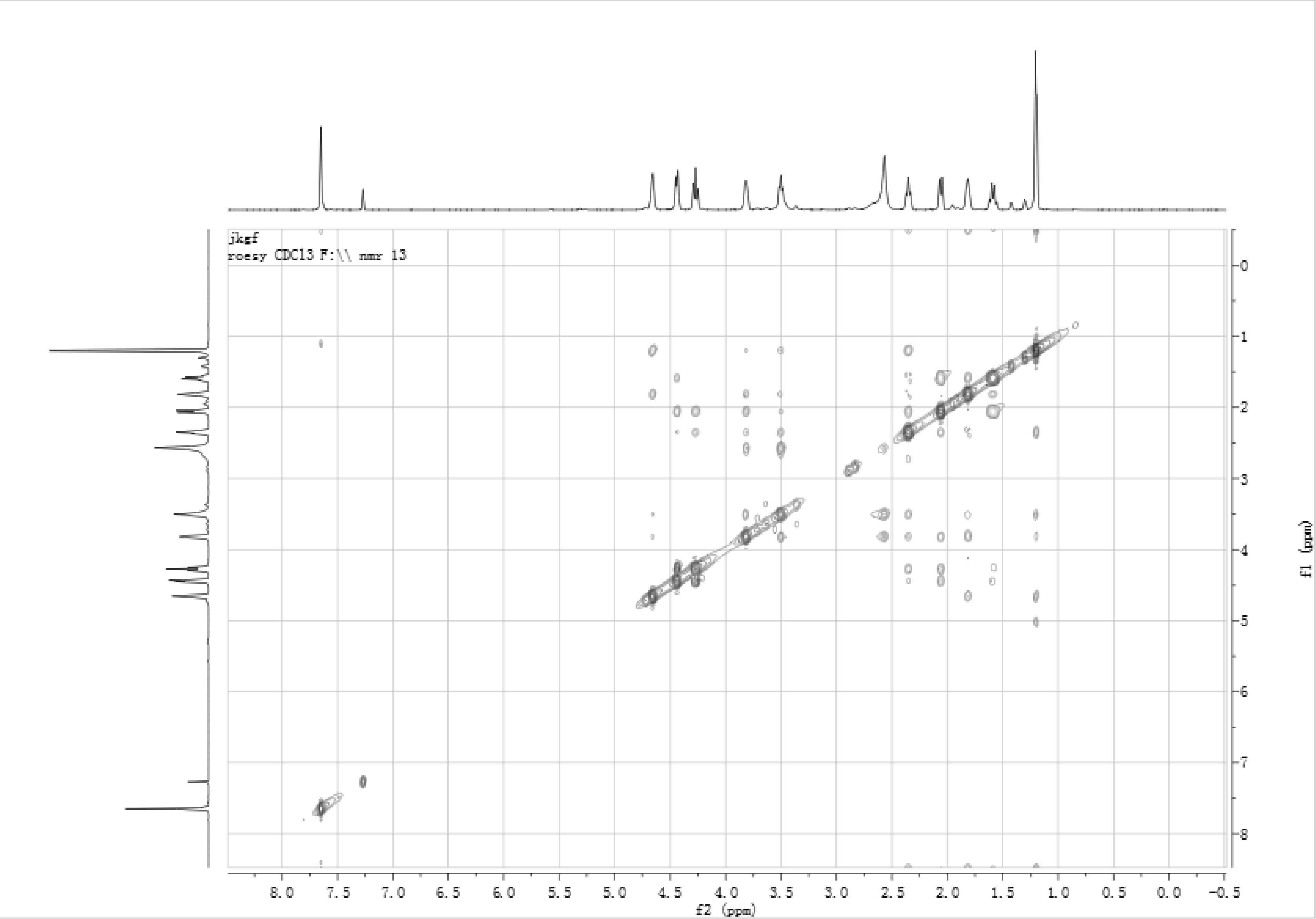

Supplement: Supplementary file 1 — Supplementary material, approximately 21.5 MB. [file 13659_2013_59_MOESM1_ESM.pdf]
